# Proceedings of the 4th Biennial Conference of the Society for Implementation Research Collaboration (SIRC) 2017: implementation mechanisms: what makes implementation work and why? part 2: Seattle, WA, USA. September 7-9, 2017

**DOI:** 10.1186/s13012-018-0715-z

**Published:** 2018-03-20

## A1 How policy contexts disable implementation

### Bianca Albers, Marg Stott, Robyn Mildon

#### Centre for Evidence and Implementation, Melbourne, Australia

##### **Correspondence:** Bianca Albers (bianca.albers@cei.org.au)

**Background**

In 2016, the Department for Family and Community Services in New South Wales, Australia selected Multisystemic Therapy - Emerging Adults (MST-EA) as a potentially suitable intervention for clients in a leaving care program with high and complex support needs emerging from challenging behaviour, mental health problems, involvement with the criminal justice system, intellectual disabilities, and alcohol and other drug use.

MST-EA was originally developed in the U.S. for young people aged 17 - 21 with a serious mental health condition and involvement in the justice system [1]. The program is an adaptation of standard MST [2] and had not been tested with a population with intellectual disabilities before. In the Australian MST-EA trial, its potential to be effective for people aged 16 - 26 with a mild to moderate disability and at high risk for poor outcomes was explored.

The first year of MST-EA implementation took place in a complex policy environment that was dominated by one of the most comprehensive social reforms in Australia – the introduction of the National Disability Insurance Scheme (NDIS). Its national roll-out began in July 2016. The NDIS follows a market-style system where government funding will no longer go directly to disability service providers, but instead to the client, who can choose the providers they want. This reform created substantial barriers to the implementation of MST-EA in New South Wales.

**Materials and Methods**

Based on the Consolidated Framework for Implementation Research [3], a semi-structured questionnaire was developed for use with 15 key stakeholders to the MST-EA Implementation. It was administered with clinicians, managers, partner organisations, consultants and program developers to explore the perceived barriers that contributed most substantially to the lack of success in adapting, transferring and implementing this evidence- based program to the Australian context.

**Results**

Data are currently being collected. Data collection will finish in May, and data analysis commence in June. Data will undergo thematic analysis guided by the Consolidated Framework for Implementation Research (CFIR). Of particular interest will be to understand in what way respondents suggest addressing the challenges that were perceived as substantial barriers to MST-EA adaptation, transport and implementation.

**Conclusions**

Too few examples of challenged implementation projects are being documented, analysed and utilised for learning. Our understanding of complex policy contexts and how to manage them during implementation requires further development. The Australian MST-EA trial mirrors an implementation experience that is shared by many other projects initiated by government or non-government organisations and providers. It should be used to inform future implementation practice and decision-making.

**References**

1. Davis M, Sheidow AJ, McCart MR. Reducing recidivism and symptoms in emerging adults with serious mental health conditions and justice system involvement. J Behav Health Serv Res. 2015;42(2):172-90.

2. Henggeler SW, Schoenwald SK, Borduin CM, Rowland MD, Cunningham PB. Multisystemic therapy for antisocial behavior in children and adolescents. New York, NY: Guilford Press; 2009.

3. Damschroder LJ, Aron DC, Keith RE, Kirsh SR, Alexander JA, Lowery JC. Fostering implementation of health services research findings into practice: a consolidated framework for advancing implementation science. Implement Sci. 2009;4(1):50.

## A2 Bringing the “Bookmobile” model to mental health: Use of mobile therapists to extend access to cognitive processing therapy in Eastern Congo

### Debra Kaysen^1^ (dkaysen@uw.edu), Alice Mudekereza^2^, Ivan Molton^3^, Cass Clemmer^2^, Judith Bass^4^

#### ^1^University of Washington, Department of Psychiatry and Behavioral Sciences, Seattle, WA, USA; ^2^IMA World Health, 1730 M Street, NW, Suite 1100, Washington, DC, USA; ^3^University of Washington, Department of Rehabilitation Medicine, Seattle, WA, USA; ^4^Johns Hopkins University, Bloomberg School of Public Health, Baltimore, MD, USA

##### **Correspondence:** Debra Kaysen (dkaysen@uw.edu)

**Background**

There is high need for mental health services for victims of sexual violence in eastern Democratic Republic of Congo (DRC). However, there is also a tremendous shortage of traditionally trained mental health professionals to provide this care, and very little infrastructure to support conventional mental health services. Cognitive Processing Therapy has been adapted to be delivered by psychosocial assistants in the Democratic Republic of Congo (DRC) and found effective in a randomized clinical trial in reducing PTSD and depression and improving overall functioning [1]. The current program examined the addition of CPT to an existing comprehensive services program, as well as utilizing mobile therapy to expand access to care and to better leverage a small number of trained providers. The existing 7-year Ushindi program provides medical, psycho-social, legal, and economic assistance to survivors of sexual violence in the DRC. Ushindi mental health services consists of active-listening therapy provided in villages by laypersons. The current program was designed to expand this model by providing CPT in three new districts as an addition to the existing program. Given limited numbers of providers trained in CPT, placing them in each village was not a feasible solution. Moreover, lack of transportation and insecurity reduced the feasibility of survivors travelling 1-2 days to receive CPT at a centrally-located setting. Such obstacles would cause missed opportunities for care and a high rate of dropout where CPT was to be provided. Ushindi approached this dilemma by implementing mobile therapy; utilizing motorbikes to transport CPT providers to provide treatment in remote villages.

**Materials and Methods**

Thirteen Congolese psychologists or psychology technicians were trained and provided with expert consultation over a 10 month period. By the end of February 2017, a total of 277 survivors had been identified and enrolled in CPT treatment, with the majority receiving mobile CPT services via providers on motorbikes. The project had anticipated a dropout rate as high as 50% if clients were expected to travel to district headquarters for counseling.

**Results**

Since inception the dropout rate has been less than 5% using mobile CPT outreach services. Although data collection is still underway, currently 142 patients have completed CPT and an additional 135 are enrolled in treatment.

**Conclusions**

Results support the use of mobile therapy as a means of implementation of an evidence-based treatment in low-resource settings to extend reach.

**Reference**

1. Bass JK, Annan J, Murray SM, Kaysen D, Griffiths S, Cetinoglu T, Wachter K, Murray LK, Bolton PA. Controlled trial of psychotherapy for Congolese survivors of sexual violence. N Engl J Med. 2013; 368:2182-91.

## A3 Supervising EBT: What content do workplace-based supervisors cover and what techniques do they use?

### Shannon Dorsey^1^, Michael D. Pullmann^2^, Suzanne E. U. Kerns^2,3^, Esther Deblinger^4^, Leah Lucid^1^, Julie Harrison^1^, Kelly Thompson^1^, Lucy Berliner^5^

#### ^1^Department of Psychology, University of Washington. Seattle, WA, USA; ^2^Department of Psychiatry and Behavioral Sciences, University of Washington, Seattle, WA, USA; ^3^Graduate School of Social Work, University of Denver, Denver, CO, USA; ^4^Rowan University, School of Osteopathic Medicine, Stratford, NJ, USA; ^5^Harborview Center for Sexual Assault and Traumatic Stress, Seattle, WA, USA

##### **Correspondence:** Shannon Dorsey (dorsey2@uw.edu)

**Background**

Workplace-based clinical supervision in public mental health is an underutilized resource for supporting evidence- based treatments (EBTs) [1], despite the fact that supervisors may offer a cost-effective way to support clinician fidelity to EBT. Very little, however, is known about the content and techniques used by workplace-based supervisors [2]; particularly in the context of EBT implementation [3].

**Materials and Methods**

Workplace-based supervisors in children’s public mental health settings audio recorded supervision sessions over the course of one year, when supervising the EBT. Data come from objective coding of these audio files (completed and analyzed). Participants were 28 supervisors, and their 98 clinician-supervisees. All supervisors and clinicians were trained in the EBT of focus (TF-CBT) as part of a Washington State-funded EBT initiative. The coding measure captured extensiveness (1-7 rating) of 27 supervision domains, which included 14 content areas (e.g., exposure, homework assignment/ review, caregiver challenges) and 13 supervision techniques (e.g., providing clinical suggestions, behavioral rehearsal, modeling, review of suggestions). Coder reliability was excellent (ICC = .87).

**Results**

Content areas that occurred in more than 50% of the supervision sessions were exposure (81%), treatment engagement (92%), trauma history (78%), coping skills (76%), caregiver challenges that impacted treatment (62%), use of art/play in treatment delivery (64%), assessment (54%) and psychoeducation (60%). Techniques that occurred in more than 50% of the sessions were information gathering (97%), teaching (93%), providing clinical suggestions (86%), and fidelity/adherence check (64%). Techniques occurring in 25% or fewer sessions were role play/behavioral rehearsal (16%), progress note review (6%), review of actual practice (5%), assigns additional training/learning (5%), and reviews suggestions/ training (5%). Most content and techniques occurred at low intensity. Only two content items occurred at high intensity in any sessions—case management (27%) and exposure (17%). Only two techniques occurred at high intensity in any sessions—supportive listening (29%) and provides clinical suggestions (12%). Other than teaching (8%), information gathering (6%), and fidelity or adherence checklist (5%), all other techniques occurred at high intensity in 1% or fewer of the coded supervision sessions.

**Conclusions**

These findings suggest that workplace-based clinical supervisors are indeed covering EBT content in supervision; but potentially at a lower intensity than may be needed to fully support clinician fidelity. Supervisors were less likely to use more “active” supervision techniques that are common in efficacy trials (role play, modeling, review recommendations), and when used, were used at low intensity.

**Acknowledgements**

NIMH-funded; MH095749 (Dorsey, PI); Washington State Department of Behavioral Health and Recovery.

**References**

1. Schoenwald SK, Mehta TG, Frazier SL, Shernoff ES. Clinical supervision in effectiveness and implementation research. Clin Psychol Sci Pract. 2013;20(1):44-59. doi:10.1111/cpsp.12022.

2. Accurso EC, Taylor RM, Garland AF. Evidence-based practices addressed in community-based children’s mental health clinical supervision. Train Educ Prof Psychol. 2011;5(2):88-96. doi:10.1037/a0023537.

3. Dorsey S, Pullmann MD, Deblinger E, Berliner L, Kerns SE, Thompson K, et al. Improving practice in community-based settings: a randomized trial of supervision – study protocol. Implement Sci. 2013;8:89. doi:10.1186/1748-5908-8-89.

## A4 Mechanisms of intermediary organizations to facilitate successful implementation: Case studies

### Robert Franks^1^, Heather Bullock^2^

#### ^1^Judge Baker Children’s Center/Harvard Medical School, Roxbury Crossing, MA, USA; ^2^McMaster University, Hamilton, Ontario, Canada

##### **Correspondence:** Robert Franks (rfranks@jbcc.harvard.edu)

**Background**

Intermediary organizations work at multiple levels with defined roles and functions to facilitate the successful implementation of best practices [1 -3]. Previous descriptive research has identified core functions of intermediaries and suggested that these functions may change over time and be responsive to local environmental and contextual factors [2,3]. These functions include acting as a purveyor of best practices, providing consultation and technical assistance, quality improvement, research and evaluation, developing best practice models, policies and systems and promoting public awareness and education [2]. Building upon this past research, our study aims to further describe the tools and mechanisms utilized by intermediaries when engaged in these identified roles and functions and further, to identify corresponding competencies and capacities necessary to be successful.

**Materials and Methods**

Using the identified descriptive model of intermediaries as a conceptual frame, we will interview intermediaries about the competencies, tools, mechanisms, and contextual adaptations utilized in the seven identified intermediary roles and functions. The interviews will be conducted in June 2017 at the Global Implementation Conference and organizations will be selected by snow-ball sampling at the conference by identifying organizations or programs that self-identify as an intermediary using the definition we provide. Using a semi-structured tool we have developed, we will conduct a minimum of six interviews with intermediaries working in diverse settings for qualitative analysis. The GIC is expected to have a wide range of participants from around the world, helping to ensure a robust sample of intermediary organizations.

**Results**

Following a qualitative analysis, we will present the major themes and results of our interviews as case examples, which will further describe in a more in depth manner the specific mechanisms being used by intermediaries in various contexts. Further, we will identify key competencies, capacities and adaptations the participants identify as necessary to provide their intermediary functions.

**Conclusions**

By better understanding the mechanisms used by intermediaries, how these mechanisms are responsive to the local needs and contextual factors, and what competencies and capacities are necessary to perform core intermediary functions, we will further articulate a model for developing and establishing successful intermediaries in various settings. By promoting and supporting intermediaries we can further facilitate successful implementation of best practices with good outcomes.

**References**

1. Franks RP., Bory CT. Strategies for developing intermediary organizations: considerations for practice. Fam Soc. 2017;98(1):27-34.

2. Franks RP, Bory CT. Who supports the successful implementation and sustainability of evidence- based practices? Defining and understanding the roles of intermediary and purveyor organizations. New Dir Child Adolesc Dev. 2015;149;41-56.

3. Franks RP. Role of the intermediary organization in promoting and disseminating mental health best practices for children and youth: The Connecticut Center for Effective Practice. Emot Behav Disord Youth. 2010;10(4):87-93.

## A5 Brief theoretically-informed pre-implementation intervention to enhance teachers’ implementation intentions and behaviors: a double-blind experiment

### Clayton Cook^1^, Aaron Lyon^2^, Yanchen Zhang^1^

#### ^1^Department of Psychology, University of Minnesota, Minneapolis, MN, USA; ^2^Department of Psychiatry and Behavioral Science, University of Washington, Seattle, WA, USA

##### **Correspondence:** Clayton Cook (crcook@umn.edu)

**Background**

High quality training and follow-up support are necessary but insufficient implementation strategies to successfully transfer evidence-based practices (EBPs) into everyday service settings [1, 2]. Even when provided with proper with training and follow-up consultation, providers adopt and deliver EBPs unevenly, resulting in weak implementation (e.g., fidelity and reach) and lackluster service recipient outcomes [2,3]. What is needed are implementation strategies that target specific malleable factors that explains why particular providers fail to deliver an EBP with adequate fidelity after receiving proper training and follow-up support [4]. Social psychological research suggests that providers’ behavioral intentions and mindsets are malleable constructs that impact motivation to engage in behavior change [5, 6]. The purpose of this study was to develop and experimentally test the effects of a theoretically-informed pre-implementation intervention designed to increase teachers’ implementation intentions and behaviors with regard to the delivery of evidence-based behavior classroom management practices in a school setting.

**Materials and Methods**

Forty-three teachers were recruited from two urban elementary schools. A double-blind randomized design was used in which teachers were randomly assigned either the intervention or attention control condition. Teachers in both conditions were provided with high quality training and follow-up consultative support. The intervention condition consisted of a brief pre-implementation intervention that integrated three applied social psychological strategies: growth mindset, saying-is-believing, and commitment and consistency. These strategies were packaged into a 1.5-hour professional interactive professional development session. The attention control condition consisted of teachers meeting for the same amount of time with their administrators to identify and problem-solve barriers to current classroom management practices. Teachers in both conditions participated in these activities two days prior to receiving high quality training in evidence-based classroom management practices. Measures included impact of theoretical mechanisms of change (i.e., implementation intentions and growth mindset), as well implementation (intervention fidelity) and student (classroom behavior) outcomes.

**Results**

Findings from repeated measures ANOVAs revealed teachers in the intervention condition demonstrated significantly greater changes in implementation intentions (d = .67), intervention fidelity (d = .54), and student outcomes (d = .45). Mediational analysis revealed that implementation intentions and growth mindset partially mediated the relationship between intervention condition and fidelity.

**Conclusions**

Findings highlight the importance of theoretically-informed pre-implementation interventions that target precise mechanisms of change (intentions and growth mindset) to promote teacher intervention fidelity in the context of proper training and follow-up consultation. This presentation will also discuss other efforts underway to develop and test pre-implementation intervention that target malleable individual-level factors.

**References**

1. Dart EH, Cook CR, Collins TA, Gresham FM, Chenier JS. Test driving interventions to increase treatment integrity and student outcomes. School Psych Rev. 2012;41:467.

2. Herschell AD, Kolko DJ, Baumann BL, Davis AC. The role of therapist training in the implementation of psycho social treatments: a review and critique with recommendations. Clin Psychol Rev. 2010;30:448-66.

3. Beidas RS, Edmunds JM, Marcus SC, Kendall PC. Training and consultation to promote implementation of an empirically supported treatment: a randomized trial. Psychiatr Serv. 2012;63:660-65.

4. Powell BJ, Beidas RS, Lewis CC, Aarons GA, McMillen JC, Proctor EK, Mandell DS. Methods to improve the selection and tailoring of implementation strategies. J Behav Health Serv Res. 2017;34:1-10.

5. Ajzen I, Manstead AS. Changing health-related behaviors: an approach based on the theory of planned behavior. In: Hewston M, de Wit JBF, van den Bos K, Schut H, Stroebe M. The scope of social psychology: theory and applications. New York: Psychology Press; 2007. p. 43-63.

6. Cialdini RB, Goldstein NJ. Social influence: compliance and conformity. Annu Rev Psychol. 2014;55:591-621.

## A5-1 Skills for developing and maintaining community-partnerships for implementation research in children’s behavioral health: Implications for research infrastructure and training of early career investigators

### Geetha Gopalan^1^, Alicia Bunger^2^, Byron Powell^3^

#### ^1^School of Social Work, University of Maryland-Baltimore, Baltimore, MD, USA; ^2^College of Social Work, Ohio State University, Columbus, OH, USA; ^3^Department of Health Policy and Management, Gillings School of Global Public Health, University of North Carolina at Chapel Hill, Chapel Hill, NC, USA

##### **Correspondence:** Geetha Gopalan (ggopalan@ssw.umaryland.edu)

**Background**

Children and youth often receive substandard mental health and child welfare services [1 - 4]. Evidence-based treatments (EBTs) are underutilized, and when they are adopted, problems with implementation can diminish their impact [5]. Thus, the National Institutes of Health (NIH) and the Institute of Medicine (IOM) have prioritized efforts to advance implementation science [6, 7]. These efforts will require that researchers partner closely with a wide range of community stakeholders to improve outcomes for children, youth, and families [8]. The purpose of this paper is to identify skills for developing and maintaining community partnerships within the context of implementation research in child welfare services.

**Materials and Methods**

Two case studies are presented, showcasing efforts of early-career investigators to partner with child welfare systems to improve the quality of behavioral health services for children, youth, and families. Case #1 focuses on a National Institute of Mental Health (NIMH)-funded exploratory/ developmental study which utilizes task-shifting strategies to implement the 4Rs and 2Ss Strengthening Families Program (4R2S) [9], originally provided by advanced mental health practitioners to reduce child disruptive behavior difficulties, so that it can be delivered by child welfare caseworkers providing placement prevention services. Case #2 involves a Children’s Bureau-funded demonstration where behavioral health screening, assessment, and referral practices are implemented within a public child welfare agency.

**Results**

Cross-cutting issues include managing stakeholder relationships, navigating regulatory constraints and human subjects review board procedures, adapting to delays and plan changes, attending to organizational culture and climate, and securing additional resources. Case studies highlight the ways in which early-career investigators are supported by the NIMH-funded Implementation Research Institute [10] to conduct community-engaged research. Moreover, recommendations are identified to enhance training and research infrastructures supporting early-career investigators who aim to partner with community stakeholders.

**Conclusions**

Strong partnerships with community stakeholders have potential to advance implementation research but can be challenging to develop and maintain. Experiences of two early career investigators provide insight into the difficulties and opportunities when working within child welfare systems to promote use of effective child behavioral health interventions.

**References**

1. Garland AF, Brookman-Frazee L, Hurlburt MS, Accurso EC, Zoffness RJ, Haine-Schlagel R, Ganger W. Mental health care for children with disruptive behavior problems: a view inside therapists’ offices. Psychiatr Serv. 2010;61:788-95.

2. Kohl PL, Schurer J, Bellamy JL. The state of parent training: program offerings and empirical support. Fam Soc. 2009;90:247-54. https://doi.org/10.1606/1044-3894.3894.

3. Raghavan R, Inoue M, Ettner SL, Hamilton BH. A preliminary analysis of the receipt of mental health services consistent with national standards among children in the child welfare system. Am J Public Health. 2010; 100:742-9. https://doi.org/10.2105/AJPH.2008.151472.

4. Zima BT, Hurlburt MS, Knapp P, Ladd H, Tang L, Duan N, et al. Quality of publicly-funded outpatient specialty mental health care for common childhood psychiatric disorders in California. J Am Acad Child Adolesc Psychiatry. 2005;44:130-44.

5. Durlak JA, DuPre EP. Implementation matters: a review of research on the influence of implementation on program outcomes and the factors affecting implementation. Am J Community Psychol. 2010;41:327-50. https://doi.org/10.1007/s10464-008-9165-0.

6. Institute of Medicine. Psychosocial interventions for mental and substance use disorders: A framework for establishing evidence-based standards. Washington, D. C.: The National Academies Press. 2015.

7. National Institutes of Health. Dissemination and implementation research in health (R01). 2016. Bethesda, Maryland: National Institutes of Health. Retrieved from http://grants.nih.gov/grants/guide/pa-files/PAR-16-238.html

8. Chambers DA, Azrin ST. Partnership: a fundamental component of dissemination and implementation research. Psychiatr Servi. 2013;64(16):509-11. https://doi.org/10.1176/appi. ps.201300032

9. Gopalan G. Feasibility of improving child behavioral health using task-shifting to implement the 4Rs and 2Ss program for strengthening families in child welfare. Pilot Feasibility Stud. 2016;2:21. doi:10.1186/s40814-016-0062-2

10. Proctor EK, Landsverk J, Baumann AA, Mittman BS, Aarons GA, Brownson RC, et al. The implementation research institute: training mental health implementation researchers in the United States. Implement Sci. 2013;8:105.

## A6 Differential cultural adaptation designs: A relevant methodological approach to empirically test the differential implementation feasibility and efficacy of cultural adapted interventions

### Gabriela Lopez-Zeron, J. Ruben Parra-Cardona, Cris Sullivan, Deborah Bybee

#### Michigan State University, East Lansing, MI, USA

##### **Correspondence:** Gabriela Lopez-Zeron (lopezga3@msu.edu)

**Background**

The cultural adaptation of evidence-based parenting interventions constitutes a promising alternative to reduce mental health disparities in the US. Implementation scholars have also emphasized the need to integrate implementation science and cultural adaptation studies. In this study, we aimed to examine whether a culturally- enhanced adapted parenting intervention with culture-specific sessions, had a significantly higher effect on feasibility and efficacy outcomes, compared to a culturally adapted intervention focused exclusively on parenting components.

**Materials and Methods**

This NIMH-funded investigation compared and contrasted the impact of two differentially culturally adapted versions of the evidence-based parenting intervention known as Parent Management Training, the Oregon Model (PMTOTM). Participants were allocated to one of three conditions: (a) a culturally adapted version of PMTO (only included PMTO core components), (b) a culturally-enhanced version of PMTO (core PMTO components and culturally-focused themes were included in this intervention), and (c) a wait-list control condition. Measurements were implemented at baseline (T1), treatment completion (T2) and 6-month follow up (T3). Initial efficacy of the adapted interventions was examined by analyzing quantitative outcome data from 190 parents. A multilevel modeling approach was utilized to analyze parenting (i.e., quality of parenting skills) and child outcomes (i.e., children’s externalizing and internalizing behaviors).

**Results**

Findings indicate high implementation feasibility of both interventions, with an overall 86% retention rate of families, including 84% of fathers. Multilevel modeling findings indicated contrasting findings with regards to initial efficacy. Specifically, whereas parents in both adapted interventions showed statistically significant improvements on their quality of parenting skills when compared to parents in the wait-list control condition, only mothers in the culturally- enhanced intervention had statistically significant improvements on children’s internalizing symptoms when compared to the two alternative intervention conditions. Similarly, only fathers allocated to the culturally-enhanced intervention had statistically significant reductions on children internalizing and externalizing symptomatology when compared to the original adapted intervention and the wait-list control condition.

**Conclusions**

Data illustrate the benefits of implementing differential cultural adaptation designs. Furthermore, contrasting findings according to level of adaptation indicates possibilities for relevant lines of research focused on integrating cultural adaptation and implementation science.

## A7 Capacity building in LMIC through adapting implementation frameworks and adopting EBPs

### Jacquie Brown (jacquie.brown@familiesfoundation.net)

#### Families Foundation, Hilversum, The Netherlands

**Background**

In partnering to implement evidence-based positive parenting programmes in a number of sub-Saharan Africa countries it has become evident that there is limited capacity and knowledge to utilise implementation science and that many international development research projects are not sustained.

Working directly with local organisations, and INGOs Families Foundation has developed a capacity building model that adapts current implementation frameworks, processes and strategies to support effective adoption of EBPs.

**Materials and Methods**

Integrating implementation science frameworks, strategies and tools Families Foundation has developed a partnership-based model of technical assistance to facilitate capacity building in five spheres: parenting, workforce skills, community planning, system networking, and monitoring and evaluation. Through virtual and in-person consultation and facilitation local organisations are supported to implement Triple P and other evidence-based practices and programmes.

**Results**

Three initiatives are at different stages of progress. These initiatives, in Kenya, South Africa and Rwanda show how a comprehensive framework with intentional flexibility supports the use and value of implementation in different contexts. The evaluation process includes developing capacity for data collection, both quantitative and qualitative. Discussion with partners includes capacity building for independent, ongoing monitoring and evaluation.

Evaluation reports are written in partnership with the implementing organisations. Results are available through these reports.

**Conclusions**

Using an implementation, capacity building model in partnership; with implementing organisations in sub-Saharan Africa can improve service delivery and sustainability as well as contribute to contextualizing and making available EBPs developed in high income countries.

## A8 Key CFIR factors in the implementation of interventions for the prevention and control of typhoid fever in low and middle income countries

### Melanie Barwick^1,2,3,4^, Raluca Barac^1,2^, Michelle Gaffey^1^, Daina Als^1^, Amruta Radhakrishnan^1^, Zulfiqar Bhutta^1^

#### ^1^Centre for Global Child Health, SickKids Hospital, Toronto, Ontario, Canada; ^2^Research Institute, SickKids Hospital, Toronto, Ontario, Canada; ^3^Department of Psychiatry, University of Toronto, Toronto, Ontario, Canada; ^4^Dalla Lana School of Public Health, University of Toronto, Toronto, Ontario, Canada

##### **Correspondence:** Melanie Barwick (melanie.barwick@sickkids.ca)

**Background**

Typhoid is a major cause of morbidity in low and middle income countries. Past research has focused on monitoring typhoid rates with little attention to how typhoid interventions had been implemented. We address this gap by examining implementation of typhoid interventions in Nigeria, Chile, Pakistan, India, Bangladesh, Vietnam, and Thailand. The study used the Consolidated Framework for Implementation Research (CFIR) to identify which factors were most strongly associated with perceived implementation success.

**Materials and Methods**

Participants included 30 public health experts in the 7 countries. Data were collected by CFIR Questionnaire. Thirty- seven constructs were measured on a scale from 1(not important) to 5 (very important) to gauge the perceived importance of each construct relative to implementation success. Given the small sample size, descriptive statistics are provided to highlight highest rate CFIR domains and constructs for each country.

**Results**

The average ratings for the 5 CFIR domains centered around and above the middle point of the scale. The same two or three constructs were rated consistently high in each of the seven countries. INTERVENTION CHARACTERISTICS: 1) evidence strength and quality, 2) relative advantage, and 3) adaptability; OUTER SETTING: 1) patient needs and resources and 2) external policy and incentives; INNER SETTING: 1) organizational incentives and rewards and 2) available resources; STAFF CHARACTERISTICS: 1) knowledge and beliefs about the intervention and 2) self-efficacy; PROCESS: 1) planning, 2) engaging, 3) formally appointed implementation leaders, and 4) reflecting and evaluating.

**Conclusions**

Identifying factors associated with implementation success has implications for advancing implementation knowledge and for improving implementation practice in global health and beyond. For instance, factors emerging as most important can be manipulated in implementation planning to improve outcomes. In addition, comparisons across settings (health, mental health, global health, education) can highlight the factors that are most robust, and set us on a path toward more effective implementation and better outcomes. There are high similarities between the present study data and CFIR studies in other contexts (health, education, mental health), highlighting the more robust factors that could lead to refinements of the CFIR model and/or support implementation in practice.

## A9 Development and testing of a brief EBP implementation intentions scale using Rasch analysis

### Joanna C. Moullin^1,2^, Mark G. Ehrhart^3^, Elisa M. Torres^1,2^, Gregory A. Aarons^1,2^

#### ^1^University of California San Diego, Department of Psychiatry, La Jolla, CA, USA; ^2^Child and Adolescent Services Research Center, University of California San Diego, La Jolla, CA, USA; ^3^San Diego State University, Department of Psychiatry, San Diego, CA, USA

##### **Correspondence:** Joanna C. Moullin (jcmoullin@gmail.com)

**Background**

Differentiating the mechanisms of implementation is not simple. Numerous factors, distributed across the levels of context in which implementation is to occur, will influence implementation processes and outcomes. Implementation research should be conducted using implementation models which hypothesize the direction and influence of such contextual factors. For example, EBP intentions are hypothesized to mediate the relationship between EBP attitudes and implementation participation. A requirement for investigating such a hypothesis is to have tools to measure the model’s parameters. While a recent measure to assess intentions to implement EBPs in general was developed [1], there appears no measure of implementation intentions for a specific EBP. Such a measure could then be tailored for other EBPs.

The Rasch model is a member of a family of models and techniques referred to as Item Response Theory. The Rasch model for measure development and testing is rare in Implementation Science, despite being increasingly used in education and health services research. In contrast, a number of implementation measures have been developed and tested using factor analysis and the Classical Test Theory standards of reliability and validity [2, 3]. This study aimed to develop and assess one implementation measure, a provider level measure of implementation intentions, using the Rasch measurement model.

**Materials and Methods**

Nine items were developed to assess intentions to implement an EBP, in this case motivational interviewing. Items were administered to 106 substance use disorder treatment (SUDT) providers across 20 SUDT programs within 4 agencies in California, USA. Rasch analysis [4] was conducted using RUMM2030 software to assess the items and their overall fit to the Rasch model, the response scale used, individual item fit, differential item functioning (DIF), and person separation.

**Results**

Rasch analysis supported the viability of the scale as a measure of implementation intentions. The scale was reduced from 9 items to 3 items, following a step-wise process to increase the feasibility and acceptability of the scale, while maintaining suitable psychometric properties. The three-item unidimensional scale showed good person separation (PSI = .802, interpreted in a similar way to Cronbach’s alpha), no disordering of the thresholds, and no evidence of uniform or non-uniform DIF.

**Conclusions**

The EBP implementation intentions scale appears to be a sound measure. Further assessment of convergent and divergent validity are proposed. The study indicates the usefulness of the Rasch method of analysis for testing the psychometric properties of implementation measures.

**References**

1. Williams NJ. Assessing mental health clinicians’ intentions to adopt evidence-based treatments: reliability and validity testing of the evidence-based treatment intentions scale. Implement Sci. 2016;11(1):60.

2. Nunnally JC. Psychometric theory. New York, NY: McGraw-Hill Publishing Company; 1978.

3. Cronbach LJ. Coefficient alpha and the internal structure of tests. Psychometrika. 1951;16(8):297-334.

4. Rasch G. Probabilistic models for some intelligence and attainment tests. Chicago, IL: University of Chicago; 1960.

## A10 Advancing the pragmatic measures construct

### Cameo Stanick^1^, Byron Powell^2^, Heather Halko^3^, Caitlin Dorsey^4^, Bryan Weiner^5^, Cara Lewis^4^

#### ^1^Hathaway-Sycamores Child and Family Services, Pasadena, CA, USA; ^2^University of North Carolina Chapel Hill, Chapel Hill, NC, 27599, USA; ^3^University of Montana, Missoula, MT 59812, USA; ^4^Kaiser Permanente Washington Health Research Institute, Seattle, WA 98101, USA; ^5^Department of Global Health and Department of Health Services, University of Washington, Seattle, WA 98195, USA

##### **Correspondence:** Cameo Stanick (cameo.stanick@gmail.com)

**Background**

There is a need for valid and reliable measures of implementation-related constructs; however, practitioners are unlikely to use these measures if they are not pragmatic. Glasgow and Riley suggest that pragmatic measures are important to stakeholders, of low burden for respondents and staff, ‘actionable,’ and sensitive to change. These criteria have considerable face validity, but were not informed by stakeholders or a systematic integration of the literature. The aim of this study was to develop a literature and stakeholder-driven operationalization of the pragmatic measurement construct for use in implementation science and related fields.

**Materials and Methods**

To accomplish this, we conducted 1) a systematic review, and 2) semi-structured interviews (*n*=7), 3) a concept mapping process (*n*=24), and 4) a two-round Delphi process with stakeholders (n=26) with experience in behavioral health and implementation research and practice.

**Results**

The systematic review and semi-structured interviews were conducted to generate a preliminary list of criteria for the pragmatic measurement construct (e.g., low cost, brief), and yielded 47 items after duplicates were removed. Concept mapping was conducted to produce conceptually distinct clusters of the pragmatic measurement criteria, and to yield item and cluster-specific ratings of their clarity and importance. The 47 criteria were meaningfully grouped into four distinct categories: 1) useful (e.g., “informs decision making”), 2) compatible (e.g., “the output of routine activities”), 3) easy (e.g., “brief”), and 4) acceptable (e.g., “offers relative advantage”). Average ratings of clarity and importance for each criterion were used to trim the list prior to the initiation of the multi-round Delphi process, which was intended to further refine the set of criteria and obtain stakeholder consensus on their clarity and importance. The two-round Delphi resulted in obtaining consensus on all but one item; although, qualitative comments provided during the Delphi process supported consensus.

**Conclusions**

The final set will be used to develop quantifiable pragmatic rating criteria that can be used to assess measures in implementation research and practice.

## A11 Psychometric assessment of three newly developed implementation outcome measures

### Bryan Weiner^1^, Caitlin Dorsey^2^, Heather Halko^3^, Cameo Stanick^4^, Byron Powell^5^, Cara Lewis^2^

#### ^1^Department of Global Health and Department of Health Services, University of Washington, Seattle, WA, USA; ^2^Kaiser Permanente Washington Health Research Institute, Seattle, WA, USA; ^3^University of Montana, Missoula, MT, USA; ^4^Hathaway-Sycamores Child and Family Services, Pasadena, CA, USA; ^5^University of North Carolina Chapel Hill, Chapel Hill, NC, USA

##### **Correspondence:** Bryan Weiner (bjweiner@uw.edu)

**Background**

Implementation outcome (IO) measures are essential for monitoring and evaluating the success of implementation efforts and comparing the effectiveness of implementation strategies. However, measures lack conceptual clarity and have questionable reliability and validity. We developed and psychometrically assessed 3 new IO measures: acceptability, appropriateness, and feasibility.

**Materials and Methods**

First, 36 implementation scientists and 27 mental health professionals assigned 31 items to the constructs, rating their confidence in assignments. We used the Wilcoxon one-sample signed rank test to assess substantive and discriminant content validity. Exploratory and confirmatory factor analysis (EFA and CFA) and Cronbach ɑ assessed the validity of our conceptual model. Next, 326 mental health counselors read one of six randomly assigned vignettes. Participants used 15 items to rate therapist’s perceptions of the acceptability, appropriateness, and feasibility of adopting an EBP. We used CFA and Cronbach ɑ to refine the scales, assess structural validity, and assess reliability. Analysis of variance (ANOVA) assessed known-groups validity. Finally, we randomly assigned half of the counselors to receive either the same vignette or the opposite vignette, and re-rate the IOs. Pearson correlation coefficients assessed test-retest reliability and linear regression assessed sensitivity to change.

**Results**

All but 5 items exhibited substantive and discriminant content validity. A trimmed CFA with 5 items per construct exhibited good model fit (CFI = 0.98, RMSEA= 0.08) and high factor loadings (0.79 to 0.94). The ɑ’s for 5-item scales were between .87-.89. Scale refinement based on measure-specific CFAs and Chronbach ɑ’s using vignette data produced 4-item scales (0.85 to 0.91). A 3-factor CFA exhibited good fit (CFI = 0.96, RMSEA = 0.08) and high factor loadings (0.75 to 0.89), indicating structural validity. ANOVA showed significant main effects, indicating known-groups validity. Test-retest reliability coefficients ranged from 0.73 to 0.88. Regression analysis indicated each measure was sensitive to change in both directions.

**Conclusions**

The 3 new measures demonstrate promising psychometric properties.

## A12 A systems approach towards the identification of implementation success

### Arno Parolini^1^, Wei Wu Tan^1^, Aron Shlonsky^1,2^

#### ^1^The University of Melbourne, Melbourne, Victoria, Australia; ^2^The University of Toronto, Toronto, Ontario, Canada

##### **Correspondence:** Arno Parolini (arno.parolini@unimelb.edu.au)

**Background**

The effectiveness of interventions and the effectiveness of implementation are usually treated as separate areas of investigation while causal links between the two are not made explicit in analytical models [1]. Some authors emphasise, however, that successful implementation in complex settings can only be measured as a cohesive construct that takes into account client outcomes, system outcomes and implementation outcomes [2]. This requires an approach that embeds interventions and their implementation within the system that is providing the service, including service providers, practitioners and clients. In such a systems model, the effects of individual implementation components and strategies can be causally linked to measures of effectiveness and potential barriers such as low fidelity can be directly expressed.

**Materials and Methods**

We develop a hypothetical population based on existing research in the fields of implementation science and child welfare. The aim is to simulate system behaviours using realistic population distributions and then investigate the mechanisms of interest using methods of causal inference. In the simulated system, interventions are introduced based on implementation frameworks [3, 4] to emphasise the link between implementation and intervention effectiveness. In particular, the model includes a series of decisions at various levels (e.g., organisation, practitioner and client) that directly affect implementation and consequently clients’ outcomes. We will use non-experimental methods to identify the effects of interest under a variety of assumptions regarding data availability and implementation components. The relationships of implementation strategies with system outcomes, implementation outcomes and clients’ outcomes are hereby of particular interest.

**Results**

We demonstrate how theoretical causal models can be used in combination with statistical methods and observational data to investigate implementation and intervention effectiveness in a systems approach. We illustrate that non-experimental quantitative methods can be used for identifying the effect of implementation strategies on implementation, systems and effectiveness outcomes when evidence-based interventions are implemented in complex practice environments or randomised controlled trials are not an option.

**Conclusions**

Embedding existing evidence into a systems model is a crucial step to advance implementation research. This process should be guided by an integration of potential sources of knowledge, including qualitative and quantitative evidence. Our findings accentuate the importance of collecting high quality data as part of routine service delivery, including data related to implementation factors. The approach presented here, when integrated with routine data collection, can be used to improve intervention outcomes at different levels of the system.

**References**

1. Curran GM, Bauer M, Mittman B, Pyne JM, Stetler C. Effectiveness-implementation Hybrid Designs: Combining elements of clinical effectiveness and implementation research to enhance public health impact. Med Care. 2012;50(3):217-26.

2. Proctor E, Silmere H, Raghavan R, Hovmand P, Aarons G, Bunger A, et al. Outcomes for implementation research: conceptual distinctions, measurement challenges, and research agenda. Adm Policy Ment Health. 2011;38(2):65-76.

3. Aarons GA, Hurlburt M, McCue Horwitz S. Advancing a conceptual model of evidence-based practice implementation in public service sectors. Adm Policy Ment Health. 2011;38:4-23.

4. Damschroder LJ, Aron DA, Keith RE, Alexander JA, Lowery JC. Fostering implementation of health services research findings into practice: a consolidated framework for advancing implementation science. Impl Sci. 2009;4:50

## A13 A case-oriented, qualitative approach to understanding predictors of prevention program sustainment

### Brittany Rhoades Cooper, Angie Funaiole, Louise Parker, Laura Hill

#### Prevention Science, Washington State University, Pullman, WA, USA

##### **Correspondence:** Brittany Rhoades Cooper (brittany.cooper@wsu.edu)

**Background**

For prevention efforts to effectively scale-up within public systems of care, we need a clear understanding of the multifaceted nature of program sustainment. Program sustainment is generally defined as the continued delivery of program activities in order to achieve continued impact, and is viewed as the final stage of effective implementation. This mixed-method study explores the community, organizational, and program factors associated with sustainment in a sample of Strengthening Families Programs (SFP) implemented under natural conditions as part of a 15-year dissemination effort in Washington State.

**Materials and Methods**

Fifty-nine SFP coordinators completed the Program Sustainability Assessment Tool (PSAT) [1] and reported sustainment level in an online survey. Twenty of these coordinators also participated in semi-structured interviews. The coding manual includes constructs from the PSAT and the Consolidated Framework for Implementation Research [2]. The qualitative analysis strategy is modeled after Damschroder & Lowery [3]: (1) a double- consensus, case-analysis approach, (2) valence coding of each identified construct, and (3) matrix analysis to identify patterns, and compare and contrast sites within and across sustainment levels (high, medium, and low). This presentation will focus on the development of the integrated coding manual and the additional insight gained from the qualitative analysis of factors associated with successful sustainment.

**Results**

Results from the quantitative analysis showed that a supportive internal and external climate for the program (environmental support), in combination with strong internal support and resources needed to effectively manage the program (organizational capacity) were conditions consistently present in those sites with high levels of reported sustainment. These results will be compared with results from the qualitative analysis currently underway. Thus far, data obtained from six interviews (two interviews at each level of sustainment) indicate that positive beliefs about the program are not sufficient. It also suggests that while organizational capacity and partnerships positively contribute to sustainment, intervention cost and external policy and incentives appear to negatively influence sustainment. The coding process will be completed for six additional interviews and the full results will be presented at the conference.

**Conclusions**

Few sustainment studies capture the multiple, intersecting factors associated with effective, long-term implementation in real-world conditions. This study addresses that gap by using a mixed methods approach to uncover the combinations of factors that distinguish between sites with high and low sustainment success. This information is critical to supporting program scale-up and ultimately improving public health.

**References**

1. Luke DA, Calhoun A, Robichaux CB, Elliott MB, Moreland-Russell S. The Program Sustainability Assessment Tool: a new instrument for public health programs. Prev Chronic Dis. 2014;11(3):e12. doi:10.5888/pcd11.130184.

2. Damschroder LJ, Aron DC, Keith RE, Kirsh SR, Alexander J, Lowery JC. Fostering implementation of health services research findings into practice: a consolidated framework for advancing implementation science. Implement Sci. 2009;4(50):40-55. doi:10.1186/1748-5908-4-50.

3. Damschroder LJ, Lowery JC. Evaluation of a large-scale weight management program using the consolidated framework for implementation research (CFIR). Implement Sci. 2013;8(1):51. doi:10.1186/1748-5908-8-51.

## A14 Sustainability of prevention programs and initiatives: A community building framework

### Suzanne Spear^1^, Lawrence A. Palinkas^2^, Sapna Mendon^2^, Juan Villamar^3^, C. Hendricks Brown^3^

#### ^1^Department of Health Sciences, California State University Northridge, Northridge, CA, USA; ^2^Department of Children, Youth and Families, Suzanne Dworak-Peck School of Social Work, University of Southern California, Los Angeles, CA, USA; ^3^Center for Prevention Implementation Methodology (Ce-PIM) for Drug Abuse and HIV, Department of Psychiatry and Behavioral Sciences, Feinberg School of Medicine, Northwestern University, Chicago, IL, USA

##### **Correspondence:** Suzanne Spear (suzanne.spear@csun.edu)

**Background**

Implementation science has typically focused on the sustainability of evidence-based practices within organizational settings like health clinics and schools. This study explored the meaning of sustainability in the context of prevention programs designed to impact substance abuse and mental health conditions at the community or population health level. The goals of population-based programs in communities may not align with the traditional view of sustainability as the long-term continuation of a pre-determined evidence-based practice in organizational settings. SAMHSA prevention programs commonly center on coalition building as a central strategy to empower community groups to identify local needs, make decisions about which strategies are appropriate, and evaluate those strategies to determine their value. Understanding the meaning of sustainability from the perspectives of practitioners working with community health programs is important if we are to design methods and tools for measuring sustainability.

**Materials and Methods**

We interviewed 45 representatives of 10 grantees within 4 SAMHSA programs (Strategic Prevention Framework– State Initiative Grants, Sober Truth on Preventing Underage Drinking [STOP-Act], Garrett Lee Smith Suicide Prevention Program, and Prevention Practices in Schools). Data collection consisted of a semi-structured interview to identify experiences with implementation and sustainment barriers and facilitators; free list exercise to elicit practitioners’ conceptions of the words “sustainability or sustainment” and what it will take to sustain their programs; and a checklist of Consolidated Framework for Implementation Research (CFIR) elements to identify which are important for sustainability. The current analysis is based on the semi-structured interviews and free lists.

**Results**

Sustainability was defined by practitioners as the continued use of an evidence-based practice (e.g., The Good Behavior Game), continued use of an evidence-based process (e.g., Strategic Planning Framework) and maintenance of coalitions and community partnerships. When asked what practitioners wished to sustain, a majority mentioned their partnerships, funding, capacity building, and evaluation. Many of the indicators of sustainability described by practitioners (e.g., community partnerships, infrastructure development, ongoing training, and funding), were also perceived to be essential requirements of sustainability. In other words, the predictors of sustainability in the context of community prevention programs are also viewed as outcomes of sustainability efforts. The context of population-based approaches to prevention contrasts with the organizational contexts described in most sustainability research in that community needs and strategies are assumed to change, sustaining the same practice over time is not necessarily a goal, strategies need to be evaluated for relevance and efficacy, and community partnerships and capacity play a central role in designing, implementing and sustaining programs.

**Conclusions**

Sustainability has different meanings depending on the context of the grant program, which can focus community efforts on developing community capacity or implementation of a single evidence-based practice. Based on the themes from the qualitative research, we are developing a model of sustainability of prevention programs that is informed by the community building framework used in public health. A community building framework places community groups, coalitions, and/or networks at the center of practice and emphasizes the importance of community capacity as well as sustainability of specific practices. Key features of the model include longstanding coalitions or provider networks, capacity (e.g., resources, training, and materials), leadership, ongoing evaluation of community needs and approaches, and integration of interventions and processes as a part of routine practice.

## A15 Identifying necessary and sufficient conditions for sustainment of evidence-based substance abuse and mental health programs

### Sapna J. Mendon^1^, Lawrence A. Palinkas^1^, Suzanne Spear^2^, Juan Villamar^3^, C. Hendricks Brown^3^

#### ^1^Department of Children, Youth and Families, Suzanne Dworak-Peck School of Social Work, University of Southern California, Los Angeles, CA, USA; ^2^Department of Health Sciences, California State University Northridge, Northridge, CA, USA; ^3^Center for Prevention Implementation Methodology (Ce-PIM) for Drug Abuse and HIV, Department of Psychiatry and Behavioral Sciences, Feinberg School of Medicine, Northwestern University, Chicago, IL, USA

##### **Correspondence:** Sapna J. Mendon (smendon@usc.edu)

**Background**

Major advances in prevention research have led to the development of numerous community-based programs that target substance abuse, mental health problems, and suicide. While previous studies have established facilitators and barriers of implementation, most have focused on adoption, and have neglected to consider factors and processes associated with sustainment [1]. The current study aimed to identify what factors are important to sustainment based on ratings of characteristics from the Consolidated Framework for Implementation Research (CFIR) and supplemental qualitative data. Specifically, we identified which conditions are necessary (conditions that must almost always be present for an outcome to occur) and which conditions are sufficient (outcome will almost always occur when these conditions are present) to sustainment.

**Materials and Methods**

Representatives from 10 grantees within 4 SAMHSA programs were interviewed to understand factors and processes of sustainment. Data collection consisted of three parts: a semi-structured interview to capture experiences with implementation and sustainment, a free list exercise, and a checklist of elements from CFIR. We used Qualitative Comparative Analysis (QCA), a set theory approach, to identify necessary and sufficient conditions across the 10 grantees. Using Boolean algebra, QCA allows us to describe causal conditions and outcomes in the context of relationships within given sets of conditions [2].

**Results**

All but 2 characteristics were rated as being important to program sustainment by more than 50% of participants. Notably, the highest rated CFIR elements were: needs and resources of the communities being served (97.4%); program champions (94.9%); assessment of progress made towards sustainment (94.7%); access to knowledge and information about the program (92.3%) and knowledge and beliefs about the program (91.4%). Least important elements were pressures to implement from other states, tribes and communities (21.1%) and organizational incentives and rewards for implementing program (45.9%). Correlational and multivariate regression analyses identified which of the 18 characteristics rated as important to sustainment by 76-100% were associated with program elements grantees sought to have sustained. These findings then informed which characteristics should be included in a QCA to determine which sets of these conditions are necessary and sufficient for sustainment.

**Conclusions**

Unique approaches to analyzing a hybrid of qualitative-quantitative data allow researchers to further expand our knowledge about implementation outcomes. In particular, QCA advances our application of a widely used framework, and enables us to understand the relationships of CFIR domains and characteristics in the context of sustainment.

**References**

1. Palinkas LA, Spear S., Mendon SJ, Villamar J, Valente T, Chou CP, et al. Measuring sustainment of prevention programs and initiatives: a study protocol. Implement Sci. 2016;11(1):95.

2. Ragin CC. The comparative method: moving beyond qualitative and quantitative strategies. Berkeley/ Los Angeles: University of California Press; 1987.

## A16 Development of a system for measuring sustainment of prevention programs and initiatives

### Lawrence A. Palinkas^1^, Suzanne Spear^2^, Sapna J. Mendon^1,^ Juan Villamar^3^, C. Hendricks Brown^3^

#### ^1^Department of Children, Youth and Families, Suzanne Dworak-Peck School of Social Work, University of Southern California, Los Angeles, CA, USA; ^2^Department of Health Sciences, California State University Northridge, Northridge, CA, USA; ^3^Center for Prevention Implementation Methodology (Ce-PIM) for Drug Abuse and HIV, Department of Psychiatry and Behavioral Sciences, Feinberg School of Medicine, Northwestern University, Chicago, IL, USA

##### **Correspondence:** Lawrence A. Palinkas (palinkas@usc.edu)

**Background**

Sustainment of prevention efforts directed at substance use and mental health problems is one of the greatest, yet least understood challenges of implementation science. A large knowledge gap exists regarding the meaning of the term “sustainment” and what factors predict or measure sustainment of effective prevention programs and support systems [1].

**Materials and Methods**

We interviewed 45 representatives of 10 grantees within 4 SAMHSA programs (Strategic Prevention Framework– State Initiative Grants, Sober Truth on Preventing Underage Drinking [STOP-Act], Garrett Lee Smith Suicide Prevention Program, and Prevention Practices in Schools). Data collection consisted of a semi-structured interview to identify experiences with implementation and sustainment barriers and facilitators; free list exercise to elicit participant conceptions of the word “sustainment” and what it will take to sustain their programs; and a checklist of Consolidated Framework for Implementation Research (CFIR) elements to identify which are important for sustainment. Lists of sustainment indicators and requirements were then compiled from each data set and compared with one another to see which items appeared on more than one list.

**Results**

Four sustainment elements were identified by all 3 data sets (ongoing coalitions, collaborations, and networks, infrastructure and capacity to support sustainment; ongoing evaluation of performance and outcomes, and availability of funding and resources) and 5 elements were identified by two of three data sets (community need for program, community buy-in and support, supportive leadership, presence of a champion, and evidence of positive outcomes. All but 2 of the CFIR domain elements were endorsed as important to sustainment by 50% or more of participants; however, not all of the CFIR elements were identified in the other data sources. The final SMS consists of 38 items, including sustainment indicators (n=3); funding and financial support (n=6); responsiveness to community needs and values (n=6); coalitions partnerships and networks (*n*=8); infrastructure and capacity to support sustainment (n=9); leadership (*n*=4); monitoring and evaluation (*n*=1); and program outcomes (*n*=1). There is some overlap between these items and one or more SAMHSA grantee reporting systems.

**Conclusions**

Although sustainment is considered the final phase of implementation, not all features of successful implementation as identified by the CFIR are considered relevant to predicting sustainment. Moreover, the overlap between indicators, requirements and capacity for and indicators and requirements of sustainment raise questions as to the nature of the construct (i.e., whether sustainment is part of the process or an outcome of implementation) and how it should be measured.

**Reference**

Palinkas LA., Spear SE, Mendon SJ, Villamar J, Valente T, Chou CP., et al. Measuring sustainment of prevention programs and initiatives: a study protocol. Implement Sci. 2016:11(1):95.

## A17 Determinants affecting delivery of early specialised vocational rehabilitation to people with traumatic brain injury in the National Health Service (NHS)

### Jain Holmes^1^, Kate Radford^1^, Pip Logan^1^, Jose Antonio Merchán-Baeza^2^, Julie Phillips^1^

#### ^1^University of Nottingham, Division of Rehabilitation and Ageing, School of Medicine, Nottingham, United Kingdom; ^2^University of Málaga, Department of Physiotherapy, Málaga, Spain

##### **Correspondence:** Jain Holmes (jain.holmes@nottingham.ac.uk)

**Background**

Findings from healthcare studies do not always translate into improved patient outcomes because of implementation difficulties. Distinguishing effectiveness and factors affecting the delivery of complex interventions is critical to evaluation and clinical implementation. An Early Specialist Traumatic brain injury Vocational Rehabilitation (ESTVR) was delivered in a multi-centre feasibility randomised controlled trial (HTA FRESH 11/66/02). It was not known whether occupational therapists (OTs), trained to deliver the intervention, would do so with fidelity and which factors might affect implementation in three English NHS major trauma centres.

**Materials and Methods**

A mixed methods design was used to examine whether ESTVR was delivered as intended and what affected implementation. A logic model was developed depicting the core ESTVR process and essential resources, a benchmark was derived from an existing study [1]. Tools measuring intervention fidelity were developed according to the Conceptual Framework for Implementation Fidelity (CFIF) and data triangulated with clinical and mentoring records then compared to the logic model and benchmark to describe fidelity and factors affecting fidelity. Implementation factors, informed by the CFIF and Consolidated Framework for Implementation Research (CFIR) were explored in interviews with 4 OTs, 15 trial participants, 6 employers and 13 NHS staff.

**Results**

Analysis of 38 clinical records (one per participant), 699 content proformas, and 12 fidelity checklists indicated while there was variation, fidelity to ESTVR logic model and the benchmark. Interviews revealed similar implementation factors across sites. Factors positively influencing fidelity; the OT’s community rehabilitation experience, expert mentoring and tailoring ESTVR to participants’ needs. Barriers included a lack of access to NHS systems, no backfill and limited support from managers. Factors that helped and hindered delivery were communication with study participants, whether the intervention was seen as acceptable, the changing needs of participants and interagency working. Determinants were mapped to all domains in CFIR and CFIF with few gaps.

**Conclusions**

Using two implementation research frameworks helped to measure fidelity and understand determinants that affected delivery. These were widespread and involved individual and provider organisation issues. Data from multiple sources identified factors likely to affect intervention fidelity in a definitive trial and clinical implementation in the NHS.

**Reference**

1. Radford K, Phillips J, Drummond A, Sach T, Walker M, Tyerman A, Haboubi N, Jones T. Return to work after traumatic brain injury: a cohort comparison study and feasibility economic analysis. Brain Inj. 2013;27(5):507-20.

## A18 Mediation analysis of the efficacy of a training and technical assistance implementation strategy on intention to implement a couple-based HIV/STI prevention intervention

### Timothy Hunt (th2258@columbia.edu)

#### Columbia University, New York, NY, USA

**Background**

The aim of this study was to examine the effectiveness and exposure of an implementation strategy, which included a 4-day in-class training with two follow-up technical assistance calls, on mediating factors hypothesized to be positively associated with staff’s intention to use a five-session, couples-based HIV and other sexually transmitted prevention intervention.

**Materials and Methods**

The Consolidated Framework for Implementation Research (CFIR) guided the study aims and analysis of the direct effect of exposure to the implementation strategy and 3 factors hypothesized to mediate the implementation strategies’ effect on intention to implement a couples-based intervention. Individual staff characteristics and an organizational process variable informed by Social Cognitive Theory (SCT), the Diffusion of Innovation Theory, and Theory of Planned Action were examined. Two hundred and fifty-three staff, predominantly African American and Latina, from 80 organizations, were recruited from HIV service agencies, clinics and community-based organization from New York City and other regions of New York State. They were randomized by agency to either a multimedia condition or a traditional paper-based version of the couples-based intervention and received the implementation strategy 4-day, in-class training followed by a technical assistance phone call at 3 and 6-months.

**Results**

We found that greater exposure to the implementation strategy in days and contacts was significantly associated with an increase in staff’s intention to implement the intervention at six months. While a statistically significant effect of the implementation strategy dose on the mediators examined was not detected, the implementer’s experience of these mediators defined as self-efficacy for couples-based implementation, positive perception of the intervention’s characteristics and the perceived availability of an organizational intervention Champion was found to be significantly associated with the outcome variable intention to implement, and also was found to reduce the dosage effect of the implementation strategy on intention. Of note, the dosage effect on intention was found to diminish at the 12 month follow-up period suggesting the importance of timely support and planning prior to and post implementation strategies to increase utilization of an innovation.

**Conclusions**

Since we observed that staff perception of their self-efficacy, positive perception of the intervention and availability of an intervention champion was significantly associated with intention further research is needed to inform the effect of training and technical assistance on these factors in the causal pathway toward implementation beyond dosage effect. Comparative analysis may be considered for future study using an analytic approach and interpretation not as reliant on p-values.

## A19 The relationship between several staff-reported mechanism of change measures and an independently rated measure of implementation integrity

### Bryan Garner^1^, David Kaiser^1^, Mike Bradshaw^1^, Liz Ball^1^, Alyssa Wolfe^1^, Jay Ford^2^, Mark Zehner^2^, Heather Gotham^3^, Traci Rieckmann^4^, Michael Chaple^5^, Kate Speck^6^, Denna Vandersloot^7^, Mat Roosa^8^, Steve Martino^9^

#### ^1^RTI International, Seattle, WA, USA; ^2^University of Wisconsin – Madison, Madison, WI, USA; ^3^University of Missouri - Kansas City, Kansas City, MO, USA; ^4^Oregon Health & Sciences University, Portland, OR, USA; ^5^NDRI, Inc, New York, NY, USA; ^6^University of Nebraska – Lincoln, Lincoln, NE, USA; ^7^Vandersloot Consulting, Portland, OR, USA; ^8^Roosa Consulting, Syracuse, NY, USA; ^9^Yale University, New Haven, CT, USA

##### **Correspondence:** Bryan Garner (bgarner@rti.org)

**Background**

The prevalence of substance use disorder among individuals living with HIV/AIDS is estimated to be 48%. Unfortunately, despite high levels of comorbid substance use and HIV/AIDS, integration of substance use and HIV/AIDS services is limited. In 2014, the National Institute on Drug Abuse (NIDA; R01-DA038146) funded the Substance Abuse Treatment to HIV Care (SAT2HIV) Project, which is a Type 2 Effectiveness-Implementation Hybrid Trial experimentally testing (a) the effectiveness of a motivational interviewing-based brief intervention (BI) for substance use and (b) the effectiveness of an organizational-level implementation strategy. The objective of the current work is to present several constructs/measures that have been hypothesized to serve as mechanisms of change for implementation strategies and to present findings regarding their relationship with an independently measured measure of implementation integrity.

**Materials and Methods**

Data for the current presentation was limited to study participants that had been randomized to receive the SAT2HIV Project’s motivational interviewing-based BI for substance use. The analytic sample included 214 client participants living with comorbid HIV/AIDS and substance use, which were clustered within 35 staff that were clustered within 21 AIDS service organizations. Implementation integrity served as the dependent measure of interest and was conceptualized as a continuous measure that represents the extent to which the brief intervention session was delivered to client participants with both adherence and competence. Multilevel regression analyses were used to examine the relationship between implementation integrity and the following three constructs/ measures: (1) implementation climate, (2) implementation readiness, and (3) leadership engagement.

**Results**

Implementation integrity was related to implementation climate (coefficient alpha = .76; β = .20, p = .027) and leadership engagement (coefficient alpha = .94; β = .18, p = .039). The relationship between implementation readiness (coefficient alpha = .94) and implementation integrity, however, was close to zero (β = -.003, p = .98).

**Conclusions**

The current results provide support for implementation climate and leadership engagement as promising constructs/measures for understanding why and how implementation strategies work to improve implementation outcomes. Future research is needed to explore the extent to which implementation climate and leadership engagement mediate the relationship between implementation strategy condition assignment and implementation integrity. Those analyses, which will require the full sample of organizations, will be conducted after completion of the SAT2HIV Project’s third and final cohort of AIDS service organizations, which is scheduled for completion in January 2018.

## A20 Necessary and sufficient implementation strategies: A qualitative comparative analysis of strategies to increase evidence-based Hepatitis C treatment in the Veterans Administration

### Vera Yakovchenko^1^, Shari Rogal^2^, Rachel Gonzalez^3^, Angela Park^4^, Timothy R. Morgan^3^, Matthew J. Chinman^5^

#### ^1^Center for Healthcare Organization and Implementation Research, Edith Norse Rogers Memorial VA Hospital, Bedford, MA, USA; ^2^Center for Health Equity Research and Promotion, VA Pittsburgh Healthcare System; Department of Surgery, University of Pittsburgh; Division of Gastroenterology, Hepatology, and Nutrition, University of Pittsburgh, Pittsburgh, PA, USA; ^3^Gastroenterology Section, VA Long Beach Healthcare System, Long Beach, CA, USA; ^4^New England Veterans Engineering Resource Center, VA Boston Healthcare System, Boston, MA, USA; ^5^Center for Health Equity Research and Promotion, VA Pittsburgh Healthcare System; RAND Corporation, Pittsburgh, PA, USA

##### **Correspondence:** Vera Yakovchenko (vera.yakovchenko@va.gov)

**Background**

The U.S. Department of Veterans Affairs (VA) is the largest hepatitis C (HCV) provider in the nation. The VA supports the use of the new evidence-based HCV treatments, which are all-oral, interferon-free regimens. The VA also supports a national HCV Innovation Team Learning Collaborative to facilitate HCV treatment using teams of providers and stakeholders. To promote the uptake of HCV treatment, individuals VA hospitals have conducted a range of the 73 implementation strategies as defined in the Expert Recommendations for Implementing Change (ERIC) study [1]. Prior analyses found that a number of strategies were associated with treatment starts and the aim of this evaluation was to assess which implementation strategies might be necessary and/or sufficient to increase HCV treatment initiation.

**Materials and Methods**

We conducted an online survey with HCVLC members and HCV clinicians at each VA hospital (N=130) to examine use of the 73 ERIC strategies. We then used fuzzy set qualitative comparative analysis (fs/QCA) to examine how different combinations of strategies might be necessary and/or sufficient to increase HCV treatment initiations at these VA hospitals. To identify specific strategies of greatest interest we conducted descriptive and nonparametric bivariate and multivariate analyses on the respondents (N=80).

**Results**

Traditional statistical approaches demonstrate the number of HCV treatment starts was positively correlated with the total number of strategies endorsed (r=0.43, p<0.001). Of the 73 ERIC implementation strategies, 28 were significantly associated with treatment starts and 26 (2 were removed due to low endorsement) were included as conditions in the fs/QCA. The number of possible combinations is 80^26, therefore reduction of conditions is needed. Preliminary results suggest several strategies of importance: developing resource sharing agreements, having an expert in HCV care meet with providers to educate them, providing ongoing HCV training, varying information delivery methods, partnering with a university to share ideas, and making efforts to identify early adopters to learn from their experiences.

**Conclusions**

Specific strategies were previously associated with HCV treatment starts at VA hospitals but this analysis will allow us to define the necessary and sufficient combinations of strategies that increase treatment starts. These regression-analytic and configurational comparative methods were used as complements to investigate implicational and covariational hypotheses regarding HCV treatment and implementation strategies used, respectively. Continued fs/QCA iterations are underway to identify necessary and/or sufficient strategies and/or combinations of strategies.

**Reference**

1. Powell BJ, Waltz TJ, Chinman MJ, Damschroder LJ, Smith JL, Matthieu MM, Proctor EK, Kirchner JE. A refined compilation of implementation strategies: results from the Expert Recommendations for Implementing Change (ERIC) project. Implement Sci. 2015;10:21.

## A21 Mapping implementation strategies in complex interventions: A protocol for process evaluation

### Alexis Huynh^1^, Erin Finley^2,3^, Melissa Farmer^1^, Bevanne Bean-Mayberry^1,4^, Alison Hamilton^1,4^

#### ^**1**^HSR&D Center for the Study of Healthcare Innovation, Implementation & Policy, VA Greater Los Angeles Health System, Los Angeles, CA, USA; ^2^South Texas Veterans Health Care System, San Antonio, TX, USA; ^3^UT Health Science Center, San Antonio, TX, USA; ^3^David Geffen School of Medicine, University of California Los Angeles, Los Angeles, CA, USA

##### **Correspondence:** Alexis Huynh (alexis.huynh@va.gov)

**Background**

Greater specification of implementation strategies is an important challenge for implementation science, but there is little guidance for evaluating complex interventions that incorporate multiple strategies within and across project phases. To strengthen VA women’s health organizational capacity for innovation in patient-centered care, the EMPOWER QUERI developed three implementation projects addressing women’s high-priority health needs. All projects use Replicating Effective Programs (REP) to guide evaluation across four phases: pre-conditions, pre- implementation, implementation, and maintenance and evolution [1]. The Cardiovascular (CV) Toolkit project entails multi-site implementation of a patient- and provider-facing toolkit designed to reduce CV risk by increasing women’s engagement in appropriate services. Our current objective is to describe a protocol for identifying strategies used in real time as part of the CV Toolkit project and specifying their key components (e.g., actors, dose, etc.) in accordance with recommendations by Proctor, et al. [2-3]. We also propose an innovative approach to longitudinal analysis that allows evaluation of the impact of overlapping or sequenced implementation strategies on adoption of and fidelity to the intervention, across multiple sites.

**Materials and Methods**

To characterize and map the implementation strategies, we applied Proctor et al.’s (2013) rubric, constructing a matrix in which we specified each implementation strategy, its conceptual group [4], and the corresponding REP phase(s) in which it occurs. For each strategy, we also specified the actors involved, actions undertaken, action targets, “dose” of the implementation strategy and anticipated outcome addressed.

**Results**

Most implementation strategies that involved developing stakeholder interrelationships and training and educating stakeholders were introduced during the pre-conditions and pre-implementation phases. Strategies introduced in the maintenance and evolution phase emphasized communication, re-examination, and audit and feedback. Some strategies appeared to serve multiple purposes in facilitating evaluation, intervention, and/or implementation activities. The mapping of implementation strategies, in addition to its value for producing valid and reliable process evaluation data, informs longitudinal analyses and supports development of an implementation playbook for scale-up and spread.

**Conclusions**

We update recent guidance on specification of implementation strategies by considering the implications for multi-strategy frameworks such as REP, and propose a novel approach for evaluating the impact of implementation packages integrating multiple strategies that vary in sequence or use across study phases and/ or sites. In operationalizing and specifying the contexts of the implementation strategies used in each phase of implementation, we seek to advance understanding of how implementation strategies – individually and in combination – function to support effective practice change.

**References**

1. Sogolow ED, Poll LSKLS, Neumann MS. Strengthening HIV prevention: application of a research-to-practice framework. AIDS Educ Prev. 2000;12:21-32.

2. Proctor EK, Powell BJ, McMillen JC. Implementation strategies: recommendations for specifying and reporting. Implement Sci. 2013;8(1):139.

3. Proctor EK, Silmere H, Raghavan R, Hovmand P, Aarons G, Bunger A, et al*.* Outcomes for implementation research: conceptual distinctions, measurement challenges, and research agenda. Adm Policy Ment Health Ment Health Serv Res. 2011;38:65-76.

4. Waltz TJ, Powell BJ, Matthieu MM, Damschroder LJ, Chinman MJ, Smith JL, et al*.* Use of concept mapping to characterize relationships among implementation strategies and assess their feasibility and importance: results from the Expert Recommendations for Implementing Change (ERIC) study. Implement Sci. 2015; 10(1):109.

## A22 A qualitative comparative analysis study of strategies for the successful implementation of cancer survivorship care plans in practice

### Sarah Birken^1^, Sara Jacobs^2^, Jamiyla Bolton^1^, Alecia Clary^1^, Miriam Tardif-Douglin^2^, Shampa Bernstein^2^, M. Alexis Kirk^1,2^

#### ^**1**^The University of North Carolina at Chapel Hill, Chapel Hill, NC, USA; ^2^RTI International, Seattle, WA, USA

##### **Correspondence:** Sarah Birken (birken@unc.edu)

**Background**

Care for the 15 million cancer survivors in the US is often poor, contributing to poor health outcomes [1-4]. Care and outcomes improve when survivors and follow-up care providers receive survivorship care plans (SCPs) – written documents containing information regarding cancer diagnosis, treatment, surveillance plans, and health promotion [5-7]. Yet SCP implementation is poor: Cancer care providers often do not develop SCPs; when they do, they frequently omit guideline-recommended content [8] and do not deliver SCPs to survivors or follow-up care providers [9]. Closing the implementation gap requires identifying strategies that high-performing cancer programs use to promote SCP implementation.

**Materials and Methods**

To date, we have used qualitative comparative analysis (QCA), which combines within-case analysis and logic- based cross-case analysis, to assess the relationship between characteristics (e.g., program type, staffing) of US cancer programs participating in the Quality Oncology Practice Initiative (QOPI), a national cancer care quality improvement initiative and SCP implementation (i.e., SCP development and delivery) (n=40). We also conducted qualitative interviews with cancer care providers in a subset of QOPI programs that performed particularly high (n=13 participants in 8 programs) or low (n=6 participants in 5 programs; as a counterfactual) with respect to SCP implementation; to analyze these data, we used template analysis, which allows for the identification of a priori and emergent themes [10].

**Results**

QCA found that high performers tended to be academic programs with social workers supporting SCP implementation or standalone oncology-only programs with staff trained in quality improvement; however, program characteristics predicted only 20-40% of the pathways to SCP implementation. Template analysis suggested that, relative to low-performers, high-performers integrated SCPs into electronic health records, saving time in developing SCPs. High-performers also had physicians who actively engaged in SCP implementation and leaders (e.g., CEOs) who valued SCPs, regularly communicated with middle managers and frontline employees (e.g., in weekly meetings), and enacted suggestions for promoting SCP implementation from middle managers and frontline employees.

**Conclusions**

QCA results based on program characteristics alone are insufficient to predict SCP implementation. Prediction may improve in pending QCA analyses, which incorporate SCP implementation determinants identified in qualitative interviews (i.e., electronic health record integration, physician engagement, leadership support). Future research is needed to understand how high-performers created conditions that facilitated SCP implementation.

**References**

1. Siegel R, DeSantis C, Virgo K, Stein K, Mariotto A, Smith T, et al. Cancer treatment and survivorship statistics. CA Cancer J Clin. 2012;62(4):220-41.

2. Cheung WY, Neville BA, Cameron DB, Cook EF, Earle CC. Comparisons of patient and physician expectations for cancer survivorship care. J Clin Oncol. 2009;27(15):2489-95.

3. Nicolaije KA, Husson O, Ezendam NP, Vos MC, Kruitwagen RF, Lybeert ML, van de Poll-Franse LV. Endometrial cancer survivors are unsatisfied with received information about diagnosis, treatment and follow-up: a study from the population-based PROFILES registry. Patient Educ Couns. 2012;88(3):427-35.

4. Mallinger JB, Griggs JJ, Shields CG. Patient-centered care and breast cancer survivors’ satisfaction with information. Patient Educ Couns. 2005;57(3):342-9.

5. Chrischilles EA, McDowell BD, Rubenstein L, Charlton M, Pendergast J, Juarez GY, Arora NK. Survivorship care planning and its influence on long-term patient-reported outcomes among colorectal and lung cancer survivors: the CanCORS disease-free survivor follow-up study. J Cancer Surviv. 2014;9(2):269-78.

6. Hewitt ME, Greenfield S, Stovall E. From Cancer Patient to Cancer Survivor: Lost in Transition. National Academies Press: Washington, D.C.; 2006

7. Rechis R, Beckjord EB, Nutt S. Potential benefits of treatment summaries for survivors’ health and information needs: results from a LIVESTRONG survey. J Oncol Pract. 2014; 10(1):75-8.

8. Salz T, Oeffinger KC, McCabe MS, Layne TM, Bach PB. Survivorship care plans in research and practice. CA Cancer J Clin. 2012;62(2):101-17.

9. Birken SA, Deal AM, Mayer DK, Weiner BJ. Determinants of survivorship care plan use in US cancer programs. J Cancer Educ. 2014;29(4):720-7.

10. King N, Symon G, Cassell C. Qualitative methods and analysis in organizational research: a practical guide. 12^**th**^ ed. Thousand Oaks, CA: Sage Publications Ltd; 1998. p.118-34.

## A23 Combining theories, process models, and frameworks to guide implementation

### Sobia Khan^1^, Shusmita Rashid^1^, Julia Moore^1^, Melissa Courvoisier^1^, Sharon Straus^1,2^

#### ^**1**^Li Ka Shing Knowledge Institute, St. Michael’s Hospital, Toronto, Ontario, Canada; ^2^University of Toronto, Toronto, Ontario, Canada

##### **Correspondence:** Sobia Khan (RashidS@smh.ca)

**Background**

Over 60 implementation theories, models, and frameworks (TMFs) exist; however, there is little direction on how to apply these in a manner that meaningfully addresses the complexity of implementation. Our aim is to present a combination of TMFs, informed by implementation science, which can be used to guide real world implementation practice.

**Materials and Methods**

We identified TMFs for three linked, but distinct phases of implementation: 1) developing an intervention; 2) implementation, evaluation, and sustainability; and 3) spread/scale up. For each phase, we selected: a process model to outline implementation steps, a theory to describe mechanisms of change or the underlying program theory, and frameworks that describe factors affecting implementation and provide guidance on how to operationalize each implementation step [1]. Whenever possible, we used TMFs in which the content is based on a literature synthesis or constitutes a meta-TMF.

**Results**

We combined three process models, two theories, and seven frameworks to describe and operationalize critical implementation steps. For phase 1 (developing a program ) we selected the Knowledge-To-Action process model [2] to outline implementation steps such as conducting a barriers and facilitators assessment and selecting and operationalizing implementation strategies, used behaviour change theories (e.g., Capability, Opportunity, Motivation – Behaviour [3]), and chose frameworks (e.g., Theoretical Domains Framework [4]), and evidence for implementation strategies. For phase 2 (implementation, evaluation, and sustainability) we selected the Quality Implementation Framework [5] as our process model. We used frameworks (e.g., the Consolidated Framework for Implementation Research [6] and Interactive Systems Framework for Dissemination and Implementation [7]), to consider the context and determine roles for program implementation. We used the Ecological Framework [8] and RE-AIM [9] to evaluate implementation; and the Sustainability planning model [10] and the Dynamic Sustainability Framework [11] to inform sustainability planning. For phase 3, (spread/scale) we selected the Framework for Going to Full Scale [12] as our process model, Theory of Diffusion as the theory, and ExpandNet as the framework. We will provide an overview and visual representation of how the theories, models, and frameworks can be used to develop, implement, evaluate, sustain, and spread/scale programs.

**Conclusions**

Our method can be used by implementation researchers and practitioners to identify and combine selected TMFs pragmatically in real-world contexts. This method can be applied using TMFs of the implementer’s choosing, and can be applied across multiple implementation settings at the micro, meso, and macro levels.

**References**

1. Nilsen P. Making sense of implementation theories, models and frameworks. Implement Sci. 2015;10:(53). doi:10.1186/s13012-015-0242-0.

2. Graham ID, Logan J, Harrison MB, Straus SE, Tetroe J, Caswell W, et al. Lost in knowledge translation: time for a map? J Contin Educ Health Prof. 2006;26(1):13-24. doi:10.1002/chp.47.

3. Michie S, van Stralen MM, West R. The behaviour change wheel: A new method for characterising and design- ing behaviour change interventions. Implement Sci. 2011;6(1):1-12. doi: 10.1186/1748-5908-6-42.

4. Cane J, O’Connor D, Michie S. Validation of the theoretical domains framework for use in behaviour change and implementation research. Implement Sci. 2012;7:37. doi: 10.1186/1748-5908-7-37.

3. Meyers DC, Durlak JA, Wandersman, Al. The Quality Implementation Framework: A Synthesis of Critical Steps in the Implementation Process. Am J Community Psychol. 2012;50:462-480. doi: 10.1007/s10464-012-9522-x.

4. Damschroder LJ, Aron, DC, Keith RE, Kirsh SR, Alexander JA, Lowery JC. Fostering implementation into practice: a consolidated framework for advancing implementation science. Implement Sci. 2009;4:50. doi:10.1186/1748-5908-4-50.

5. Wandersman A, Duffy J, Flashpohler P, et. al. Bridging the gap between prevention research and practice: The Interactive Systems Framework for dissemination and implementation. Am J Community Psychol. 2008; 41(3-4):171-181. doi: 10.1007/s10464-008-9174-z.

6. Durlak JA, DuPre EP. Implementation matters: A review of research on the influence of implementation on program outcomes and the factors affecting implementation. Am J Community Psychol. 2008;41(3-4):327-350. doi: 10.1007/s10464-008-9165-0.

7. Glasgow RE, Vogt TM, Boles SM. Evaluating the public health impact of health promotion interventions: the RE-AIM framework. Am J Public Health. 1999;89(9):1322-7.

8. Johnson K, Hays C, Center H, Daley C. Building capacity and sustainable prevention innovations: a sustain- ability planning model. Eval Program Planning. 2004;27:135-149. doi: 10.1016/j.evalprogplan.2004.01.002.

9. Chambers DA, Glasgow RE, Stange KC. The dynamic sustainability framework: addressing the paradox of sustainment amid ongoing change. Implement Sci. 2013;8(117):1-11. doi: 10.1186/1748-5908-8-117.

10. Barker PM, Reid A, Schall MW. A framework for scaling up health interventions: lessons from large-scale improvement initiatives in Africa. Implement Sci. 2016;11(1):12. doi: 10.1186/s13012-016-0374-x.

11. Rogers, Elliot M. New Product Adoption and Diffusion. J Consum Res Research. 1976;2(4):290-301. https://academic.oup.com/jcr/article-abstract/2/4/290/1820436/New-Product-Adoption-and-Diffusion. Accessed March 2, 2017.

12. Nine steps for developing a scaling up strategy. World Health Organization. www.who.int/ reproductivehealth/publications/strategic_approach/9789241500319/en/.2010. Accessed March 1, 2017.

## A24 Importance and feasibility of a revised compilation of implementation strategies to support education sector behavioral health

### Aaron Lyon^1^, Clayton Cook^2^, Jill Locke^1^, Chayna Davis^1^, Byron Powell^3^, Thomas Waltz^4^

#### ^1^University of Washington, Seattle, WA, USA; ^2^University of Minnesota, Minneapolis, MN, USA; ^3^University of North Carolina at Chapel Hill, Chapel Hill, NC, USA; ^4^Eastern Michigan University, Ypsilanti, MI USA

##### **Correspondence:** Aaron Lyon (lyona@uw.edu)

**Background**

The Expert Recommendations for Implementing Change (ERIC) project’s compilation of implementation strategies in healthcare [1-3] has provided a much needed common language for implementation practitioners and researchers, and allowed for better specified evaluations of implementation interventions [4]. Unfortunately, no comparable effort has occurred to support implementation of a broader range of student support programs in schools. Given that the education sector has a number of unique implementation challenges (e.g., timelines, personnel, policies) [5, 6], strategies designed to support clinical practice in more traditional healthcare settings may require adaptation for use in schools.

**Materials and Methods**

ERIC strategies were systematically adapted via the following steps: (1) Review of existing strategies and revision of language, terms, and constructs for schools; (2) Refinement of definitions and generation of education sector examples; (3) Removal of a small number of strategies determined to be inappropriate for school-based implementation; (4) Addition of novel, contextually appropriate implementation strategies; (5) Review of the updated compilation by ERIC developers ensure conceptual consistency; (6) Further revision by school experts; and (7) Re-review by ERIC developers and finalization. Following, the strategies were presented via an online survey to a large sample (n = ~200) of school-based behavioral health consultants across the state of California, who rated the importance and feasibility of each strategy.

**Results**

The adaptation process produced (1) a revised compilation of school-focused implementation strategies (n = 75), (2) information about the school context that prompted revision, and (3) a catalog of the types of changes that were made. Among other revisions, implementation strategies focused on financial incentives were de-emphasized for the school setting, while new strategies (e.g., “pruning” competing initiatives) were added. In keeping with the work of the ERIC authors [3], results from the online survey were compiled and strategies simultaneously evaluated along importance and feasibility dimensions.

**Conclusions**

This study suggests substantial transportability of the ERIC implementation strategies to schools, but underscores critical ways that contextual appropriateness can be optimized. Results from the survey of behavioral health consultants will be compared to those from Waltz et al. [3] to determine whether the relative importance or feasibility of each strategy varied in the current context and sample. Building on these findings, the presentation will articulate an implementation strategy research agenda for schools that explores mechanisms of action for specific strategies [7] and evaluates strategy variations based on their application to different levels of prevention and intervention programming within schools (ranging from universal prevention to indicated clinical services).

**References**

1. Powell BJ, McMillen JC, Proctor EK, Carpenter CR, Griffey RT, Bunger AC, et al. A compilation of strategies for implementing clinical innovations in health and mental health. Med Care Res Rev, 2012;69(2):123-57.

2. Powell BJ, Waltz TJ, Chinman MJ, Damschroder LJ, Smith JL, Matthieu MM, Proctor EK, Kirchner JE. A refined compilation of implementation strategies: results from the Expert Recommendations for Implementing Change (ERIC) project. Implement Sci. 2015;10:21.

3. Waltz TJ, Powell BJ, Matthieu MM, Damschroder LJ, Chinman MJ, Smith JL, et al.. (2015). Use of concept mapping to characterize relationships among implementation strategies and assess their feasibility and importance: results from the Expert Recommendations for Implementing Change (ERIC) study. Implement Sci. 2015;10(1):109.

4. Proctor EK, Powell BJ, McMillen JC. Implementation strategies: recommendations for specifying and reporting. Implement Sci. 2013;8(1):139.

5. Forman SG, Shapiro ES, Codding RS, Gonzales JE, Reddy LA, Rosenfield SA, et al. Implementation science and school psychology. Sch Psych Q. 2013;28(2):77.

6. Owens JS, Lyon AR, Brandt NE, Warner CM, Nadeem E, Spiel C, Wagner M. Implementation science in school mental health: key constructs in a developing research agenda. Sch Ment Health. 2012;6(2):99-111.

7. Lewis C, Boyd M, Beidas R, Lyon A, Chambers D, Aarons G, Mittman, B. A research agenda for mechanistic dissemination and implementation research. Presented at the 8th Annual Conference on the Science of Dissemination and Implementation, Bethesda, MD. 2015.

## A25 Qualitative research in Implementation Science (QUALRIS): Strong methods for strong science

### Alison Hamilton^1^, Deborah Cohen^2^, Benjamin Crabtree^3^, Laura Damschroder^4^, Jennifer Leeman^5^, Deborah Padgett^6^, Lawrence Palinkas^7^, Borsika Rabin^8^, Heather Schacht Reisinger^9^, Suzanne Heurtin-Roberts ^10^

#### ^1^U.S. Department of Veterans Affairs & University of California, Los Angeles, Los Angeles, CA, USA; ^2^Oregon Health & Science University, Portland, OR, USA; ^3^Rutgers University, New Brunswick, NJ, USA; ^4^U.S. Department of Veterans Affairs, Washington DC, USA; ^5^University of North Carolina at Chapel Hill, Chapel Hill, NC, USA; ^6^New York University, New York, NY, USA; ^7^University of Southern California, Los Angeles, CA, USA; ^8^University of San Diego, San Diego, CA, USA; ^9^U.S. Department of Veterans Affairs & University of Iowa, Iowa City, IA, USA; ^10^National Cancer Institute, Bethesda, MD, USA

##### **Correspondence:** Alison Hamilton (alisonh@ucla.edu)

**Background**

Qualitative methods are vitally important to and widely employed in implementation science (IS), usually in tandem with quantitative methods. However, inadequate attention has been given to the specific demands of qualitative methods in the context of IS. Limited guidance is available in the field as to what rigorous qualitative approaches might be most productively used, for which research questions and settings. This threatens the scientific integrity and practical utility of IS as it develops. To remedy this, the Qualitative Research in Implementation Science (QUALRIS) project was launched.

**Materials and Methods**

Since June 2015 a group of ten leaders in IS, qualitative research, or both was convened by the National Cancer Institute’s Implementation Science Team to develop guidance for using qualitative methods in IS, and to recommend future efforts to improve rigor and utility. The QUALRIS group interacts via teleconference, email, and as an NCI online learning community (https://researchtoreality.cancer.gov). Consulting best practices literature in qualitative methods, and members’ own extensive experience, the group determined focal areas to examine through an iterative consensus process, and drafted pertinent guidelines.

**Results**

The group agreed that IS presents qualitative methods with particular challenges, including conceptual rigor, time constraints, complexity of implementation and intervention, multiple implementation strategies, limited engagement in practice settings, dynamic, changeable practice settings, little control of research environment, sustainability, and scale-up and spread.

The QUALRIS group drafted guidance in the following domains: 1) employing qualitative methods relevant to research questions and conceptual models rather than “default” methods; 2) increased attention to procedures designed to achieve qualitative standards of trustworthiness, and documentation of adherence to those procedures; 3) rationales for format and content of interview and focus group guides, with attention to conceptual underpinnings; 4) documentation and explanation of data analysis logic and procedures; 5) improved presentation of qualitative findings in IS publications. Increased qualitative expertise on research teams and increased training in qualitative methods for IS researchers is recommended.

**Conclusions**

QUALRIS guidance and recommendations offer a resource for consistent, rigorous standards for using qualitative methods in IS. As such, this effort can strengthen the scientific integrity and utility of implementation science.

## A26 Outcomes and sustainability of a medication reconciliation electronic health record tool implemented within an infectious disease clinic

### Travis Lovejoy^1^, Scott Ragland^2^, Kathleen Adams^2^, Victoria Church^2^, Stephanie Tallett^2^, Mimi Ferraro^2^, Anthony Sayers^2^, Patricia Holahan^3^, Blake Lesselroth^2^

#### ^**1**^Center to Improve Veteran Involvement in Care, VA Portland Health Care System, Portland, OR, USA; ^2^Northwest Innovation Center, VA Portland Health Care System, Portland, OR, USA; ^3^Stevens Institute of Technology, Hoboken, NJ, USA

##### **Correspondence:** Travis Lovejoy (travis.lovejoy@va.gov)

**Background**

Inaccurate information about patients’ current medication regimens can lead to prescribing errors that result in serious adverse events. Medication reconciliation (MR) is a process by which clinicians document all current prescribed and over-the-counter medications, list medication allergies and issues, and adjust prescriptions accordingly. Ideally, these activities are performed at each patient visit. However, significant barriers to implementation of MR in ambulatory care settings include lack of a standardized process used across the care team, focus on medications that directly pertain to a patient’s immediate issues or chronic conditions to the detriment of evaluating the other medications taken by the patient, suboptimal workflow, and fragmented layout of information in the electronic health record used in the MR process. The Automated History Intake Device (APHID) is an evidence-based informatics tool that addresses implementation barriers by gathering and centralizing information needed to perform MR in the electronic medical record system of the Veterans Health Administration (VHA). We report findings from an implementation of APHID into the Infectious Disease (ID) Clinic of one VHA health care system.

**Materials and Methods**

The APHID implementation strategy was guided by the Consolidated Framework for Implementation Research and the Effective Technology Use Model. The strategy included initial education to clinic staff (physicians, nurses, and medical support assistants), workflow redesign, initial “at-the-elbows” support, and periodic audit and feedback on achievement of performance goals. Patient-level data were obtained from the VHA electronic medical record.

**Results**

Prior to implementation of APHID, the ID Clinic fell well below target goals of 80% for patient encounters at which MR is performed (20%), medication discrepancies resolved (25%), and an MR after-visit summary provided to patients (27%). At the conclusion of the 7-month implementation, MR had been performed for 95% of patient encounters, medication discrepancies had been resolved for 62% of patients, and 98% received an MR after-visit summary. Three-month post-implementation follow-up data indicate improvements in MR within the ID Clinic were maintained following the removal of the external implementation team.

**Conclusions**

We successfully implemented APHID and modified clinic workflow to support implementation using a multifaceted implementation strategy that included education, facilitation, audit and feedback. We further describe resources the implementation team has provided to clinic management to promote sustainability by allowing the clinic to perform periodic audit and feedback so clinic practices and workflow can be adjusted should MR metrics fall below the target goal of 80%.

## A27 The collaborative development of an electronic health record registry tool to support implementation of collaborative care for perinatal depression

### Ian Bennett^1,2^, Rachel Gold^3^, Amy Bauer^2^, Mindy Vredevoogd^2^, Marla Dearing^4^, Mary Middendorf^4^, Perry Foley^4^

#### ^1^Department of Family Medicine, University of Washington, Seattle, WA, USA; ^2^Department of Psychiatry and Behavioral Sciences, University of Washington, Seattle, WA, USA; ^3^Kaiser Permanente NW Center for Health Research, Portland, OR, USA; ^4^OCHIN Inc., Portland, OR, USA

##### **Correspondence:** Ian Bennett (ibennett@uw.edu)

**Background**

Collaborative Care is a team-based strategy for identifying and managing depression in primary care [1,2]. Though evidence from dozens of clinical trials support its effectiveness, it has not been widely implemented in real-world settings, and multi-site implementation efforts resulted in wide variation in site level clinical outcomes [3]. One obstacle to implementation of this complex care transformation intervention is the lack of a care registry tool within the electronic health record (EHR) designed to support the work of care managers on the collaborative care team. EHR registry tools designed to support clinical interventions are often developed with minimal input from care team members who have experience with the relevant intervention. This undermines such tools’ effectiveness at supporting implementation of clinical innovations.

**Materials and Methods**

As part of an implementation trial of collaborative care for perinatal depression in 20 federally qualified health centers (FQHCs; trials.gov NCT02976025), we developed a registry tool in partnership with primary care teams, and built this tool into the EHR (Epic©) shared by our study sites.

**Results**

Key elements from an existing free-standing (non-integrated into an EHR) care management system developed over the last two decades to support collaborative care (Care Management Tracking System; CMTS) were identified for transfer into this tool including a dashboard organized to support the care processes. A team of clinicians with long-standing experience in the collaborative care model (including those experienced with using both the CMTS and Epic EHR in collaborative care for perinatal depression), developers from the CMTS system, and Epic developers, worked in an iterative manner to create workflows, prototypes, and final build of this integrated registry tool. Associated training for use of this tool was also developed in this process.

**Conclusions**

A patient registry was successfully developed and deployed within an EHR to support Collaborative Care for perinatal depression. Research is needed to assess the registry’s utility and usability in this setting and well as the impact on implementation of collaborative care.

**References**

1. Katon W, Unützer J. Collaborative care models for depression - time to move from evidence to practice. Arch Intern Med. 2006;166(21):2304-6.

2. Katon W, Unützer J, Wells K, Jones L. Collaborative depression care: history, evolution and ways to enhance dissemination and sustainability. Gen Hosp Psychiatry. 2010;32(5):456-64.

3. Solberg LI, Crain AL, Jaeckels N, Ohnsorg KA, Margolis KL, Beck A, et al. The DIAMOND initiative: implementing collaborative care for depression in 75 primary care clinics. Implement Sci. 2013;8:135.

## A28 Can an electronic health record (EHR) promote implementation quality and fidelity in children’s behavioral health? Results of a randomized study

### Eric Bruns^1^, Alyssa Hook^1^, Isabella Esposito^1^, Elizabeth Parker^1^, April Sather^1^, Kelly Hyde^2^

#### ^1^University of Washington, Seattle, WA, USA; ^2^Fidelity EHR, Santa Fe, NM, USA

##### **Correspondence:** Eric Bruns (ebruns@uw.edu)

**Background**

Electronic health records (EHR) and better coordination of care have both been identified as health care priorities. However, only about 30% of behavioral health providers have implemented EHR. Moreover, few studies address the question of how EHR adoption may affect implementation of common factors of research-based care.

**Materials and Methods**

We developed an EHR that aligns with core elements and implementation steps of the research-based wraparound process for youth with complex behavioral health needs and their families [1,2]. Wraparound facilitators working in two provider organizations were randomized to use the EHR (n=18) or paper-based services as usual (SAU; n=13). Variables included (1) ratings of EHR usability and acceptability; (2) service outcomes; (3) facilitator job satisfaction and attitudes toward standardized assessment; and (4) short term (4-month) outcomes.

**Results**

Facilitators’ ratings on the System Acceptability & Appropriateness Scale (SAAS) were high, but usability scores were in the “marginal” range on the System Usability Scale (SUS). EHR facilitators showed significant increases in use of standardized assessment data in treatment planning. Wraparound Fidelity Index (WFI) scores were significantly higher for one subscale (Teamwork) for the EHR group.

**Conclusions**

The current study facilitated continued improvement of this wraparound-specific EHR, and found support for some hypothesized short-term service outcomes. Further research is needed that employs a refined version of the software, more robust EHR implementation support, and longer follow-up.

**References**

1. Bruns EJ, Pullmann MD, Sather A, Brinson RD, Ramey M. Effectiveness of Wrap around versus case management for children and adolescents: results of a randomized study. Adm Policy Ment Health. 2014; 42(3):309-22.

2. Bruns EJ, Hyde KL, Sather A, Hook A, Hensley S, Lyon AR. Applying user input to the design and testing of an electronic behavioral health information system for wraparound care coordination. Adm Policy Ment Health. 2016;43(3):350-68.

## A29 Using direct observation to guide implementation facilitation

### Bo Kim^1,2^, Christopher J Miller^1,2^, Mark S Bauer^1,2^, A Rani Elwy^1,3^

#### ^**1**^VA Health Services Research & Development Center for Healthcare Organization and Implementation Research, Washington DC, USA; ^2^Harvard Medical School, Boston, MA, USA; ^3^Boston University School of Public Health, Boston, MA, USA

##### **Correspondence:** Bo Kim (bo.kim@va.gov)

**Background**

Implementation facilitation is being increasingly employed as a strategy to enhance the use of evidence-based approaches in health care delivery [1]. However, there are limited established methods for thoroughly collecting data on ongoing facilitation experiences and systematically feeding them back to facilitators to help prospectively shape their facilitation activities. To address this methodological gap, we developed and piloted a method for collection and feedback of data based on direct observation [2,3] of facilitation activities.

**Materials and Methods**

We developed this direct observation method for facilitation within the context of a multi-site stepped-wedge controlled trial to implement interdisciplinary team-based behavioral health care at Department of Veterans Affairs (VA) medical centers [4]. We designed observations that would provide insight into elements of implementation as outlined in the Integrated Promoting Action on Research Implementation in Health Services (i-PARIHS) framework. Three external facilitators (EFs) on the study team each worked with the internal facilitator (IF) at three sites (N=9 sites), the site’s interdisciplinary team of providers, and additional stakeholders including facility leaders.

**Results**

Direct observation of facilitation focused on three domains – Site Characteristics, Implementation Status, and Resource Utilization, aligning to i-PARIHS elements and providing actionable feedback to facilitators for each site. Four observation parameters of Observer, Subject, Mode, and Timing were specified for each domain. For Site Characteristics, the EFs gathered information into a pre-implementation assessment document, which was shared with the IFs, provider teams, and stakeholders to collaboratively plan for subsequent implementation.

For Implementation Status, the EF and IF held weekly phone calls to discuss the team’s observed progress and plan for upcoming implementation steps, keeping record of their discussions in a shared coordination document. For Resource Utilization, the EFs compared their activity logs across the sites, noting trends and anomalies that enabled estimation of facilitation resources that would be needed for team-based behavioral health care to be implemented at all VA facilities.

**Conclusions**

Direct observation of facilitation allowed systematic and replicable collection and regular feedback of data on vocalized perceptions/interactions, nonverbal behavior/appearances, care setting/space, team/clinical processes, and utilization of facilitation resources. This method and its associated tools (including conversation guide, templated documents, and activity logs) can help steer facilitation activities toward implementation that fits local and changing contexts both within and outside the realms of behavioral health and VA. Direct observation methods can also be considered more generally for formative evaluation to assess and provide feedback on implementation strategies beyond facilitation.

**References**

1. Kirchner JE, Ritchie MJ, Pitcock JA, Parker LE, Curran GM, Fortney JC. Outcomes of a partnered facilitation strategy to implement primary care-mental health. J Gen Intern Med. 2014; 29(Suppl 4):904-12.

2. Leslie M, Paradis E, Gropper MA, Reeves S, Kitto S. Applying ethnography to the study of context in healthcare quality and safety. BMJ Qual Saf. 2014; 23:99-105.

3. Neuwirth EB, Bellows J, Jackson AH, Price PM. How Kaiser Permanente uses video ethnography of patients for quality improvement, such as in shaping better care transitions. Health Aff. 2012;31:1244-50.

4. Bauer MS, Miller C, Kim B, Lew R, Weaver K, Coldwell C, et al. Partnering with health system operations leadership to develop a controlled implementation trial. Implement Sci. 2016;11:22.

## A30 Providers as co-reviewers in fidelity assessments of Assertive Community Treatment (ACT) teams: Establishing feasibility and acceptability

### Maria Monroe-DeVita^1^, Lorna Moser^2^, Sarah Kopelovich^1^, Roselyn Peterson^1^, Stacy Smith^2^, MacKenzie Hughes^1^

#### ^1^University of Washington School of Medicine, Seattle, WA, USA; ^2^University of North Carolina Institute for Best Practices, Chapel Hill, NC, USA

##### **Correspondence:** Maria Monroe-DeVita (mmdv@uw.edu)

**Background**

Fidelity assessment is an important mechanism featured within various implementation frameworks [1]. It has been shown to predict better clinical outcomes [2,3], and can be a useful quality improvement tool [4]. In spite of these benefits, many gold-standard approaches (e.g., rating audio-taped sessions) can be costly and burdensome. The authors present a more practical, feasible approach to fidelity assessment of Assertive Community Treatment (ACT) programs, utilizing ACT providers as co-reviewers.

**Materials and Methods**

The authors are piloting a provider co-reviewer process to fidelity reviews of 91 ACT teams in two states. One university/state representative (N=10) serves as lead reviewer, joined by one ACT provider co-reviewer (N=41). Fidelity assessments are conducted onsite with each ACT team over a two-day period. After the assessment, each reviewer independently rates team fidelity across the 47-item Tool for Measurement of ACT (TMACT [5]); reviewers then develop final consensus ratings. Surveys on the feasibility and acceptability of this approach are conducted with provider co-reviewers and reviewed ACT teams. Correlations between team co-reviewer participation and their respective team’s fidelity will be further examined.

**Results**

Preliminary results suggest that ACT teams with provider co-reviewers on their team score significantly higher on the TMACT (R=.61, p<.001). Results will also be reported on provider experiences of conducting fidelity reviews, including the extent to which serving as a co-reviewer provided an opportunity to better learn ACT. Results will further report on teams’ experiences of having another ACT team provider conduct a fidelity review of their team.

**Conclusions**

We hypothesize that enlisting ACT providers as co-reviewers is a feasible and acceptable approach to conducting ACT fidelity reviews. This process may yield more hands-on opportunities for learning and improving fidelity within co-reviewers’ own teams. Enlisting providers as co-reviewers in fidelity reviews could be a promising approach to fidelity assessment of other team-based evidence-based practices. Future studies should focus on cost- effectiveness of provider-based fidelity review processes.

**References**

1. Tabak RG, Khoong EC, Chambers DA, Brownson RC. Bridging research and practice: models for dissemination and implementation research. Am J Prev Med. 2012;43(3):337-50.

2. Cuddeback GS, Morrissey JP, Domino ME, Monroe-DeVita M, Teague GB, Moser LL. Fidelity to recovery- oriented ACT practices and consumer outcomes. Psychiatr Serv. 2013;64(4):318-23.

3. McGuire AB, White DA, Bartholomew T, Flanagan ME, McGrew JH, Rollins AL, Mueser KT, Salyers MP. The relationship between provider competence, content exposure, and consumer outcomes in illness management and recovery programs. Adm Policy Ment Health. 2016;44(1):81-91.

4. Monroe-DeVita M, Teague GB, Moser LL. The TMACT: a new tool for measuring fidelity to assertive community treatment. J Am Psychiatr Nurses Assoc. 2011;17(1):1729.

5. Monroe-DeVita M, Moser LL, Teague GB. The tool for measurement of assertive community treatment (TMACT). In: McGovern M, McHugo G, Drake Bond G, & Merrens M, eds. Implementing evidence-based practices in behavioral health. Center City, MN: Hazelden; 2013.

## A31 Process evaluation of the Transform-Us! program to promote children’s physical activity and reduce sedentary behaviour

### Harriet Koorts^1^, Anna Timperio^1^, Gavin Abbott^1^, Lauren Arundell^1^, Nicky Ridgers^1^, Ester Cerin^2^, Helen Brown^1^, Robin Daly^1^, David Dunstan^3^, Kylie Ball^1^, David Crawford^1^, Claire Hume^4^, Mai Chinapaw^5^, Lauren Sheppard^6^, Marj Moodie^6^, Kylie Hesketh^1^, Jo Salmon^1^

#### ^1^Institute for Physical Activity and Nutrition (IPAN), School of Exercise and Nutrition Sciences, Deakin University, Melbourne, VIC, Australia; ^2^Institute for Health & Ageing, Australian Catholic University, Melbourne, VIC, Australia; ^3^Baker IDI Heart and Diabetes Institute, Melbourne, VIC, Australia; ^4^University of Adelaide, Adelaide, South Australia, Australia; ^5^Department of Public and Occupational Health and the EMGO Institute for Health and Care Research, VU University Medical Center, Amsterdam, The Netherlands; ^6^Deakin Health Economics, School of Health and Social Development, Deakin University, Melbourne, VIC, Australia

##### **Correspondence:** Harriet Koorts (h.koorts@deakin.edu.au)

**Background**

Transform-Us! is a school-based intervention to increase physical activity and reduce sedentary behaviour among primary school children. The efficacy of Transform-Us! was tested in a cluster randomised controlled trial (RCT) among 20 primary schools. The aims of this study were to evaluate program reach, dose, fidelity, appropriateness, satisfaction and sustainability, and the association between implementation level and outcomes.

**Materials and Methods**

A mixed method post-hoc design was adopted based on UK Medical Research Council (MRC) recommendations. Surveys of teachers, parents and children at baseline, 18-months, 30-months and 2.5 years post baseline assessed process evaluation indicators. Children wore GT3X ActiGraph accelerometers for 7 days to determine physical activity and sedentary behaviour. Teachers were grouped by levels of implementation based on the proportion of the entire intervention delivered: (i) ‘Low’ (<33%); (ii) ‘Moderate’ (>33%< 67%); and (iii) ‘High’ (>67%). Implementation data was pooled across intervention groups. Linear and logistic regression analyses examined between group differences in implementation, and the association between implementation level and child physical activity and sedentary behaviour outcomes. Qualitative survey data were analysed thematically to examine implementation barriers and facilitators.

**Results**

Among intervention recipients, 52% (n=85) of teachers, 29% (n=331) of parents and 92% (n=407) of children (58% girls; mean age [SD]: 8.2 [0.47 years]) completed baseline evaluation surveys. At T3, teachers delivered on average 70% of the key messages, 65% set active/standing homework, 30% reported delivering >1 standing lesson p/day and 56% delivered active breaks. The majority of teachers (96%) made sports equipment available and used sports equipment in class (81%). Fidelity and dose of key messages and active/standing homework reduced over time. Fidelity to standing lessons, active breaks and sports equipment use increased. Teachers (48%) reported moderate levels of implementation at T3, and low levels of implementation at T4 (46%). Implementation level and child physical activity and sedentary behaviour outcomes were not associated. Qualitative themes identified integration of the program into existing practices, children’s enjoyment and teachers’ awareness of program benefits facilitated delivery and sustainability.

**Conclusions**

This study has demonstrated changes to intervention dose and fidelity over time, and the importance of senior school leadership and effective integration of interventions for improved delivery and sustainability. Strategies to maximise participant response rates and enhance quantifying implementation would improve our understanding of the association between implementation and outcomes. Findings have informed the recently funded scale up of Transform-Us! across Victoria, Australia.

## A32 Systematic multi-method documentation of adaptations in five health system interventions

### Borsika Rabin^1,2,3^, Marina McCreight^2^, Russell Glasgow^2,3^

#### ^1^Department of Family Medicine and Public Health, School of Medicine, University of California San Diego, La Jolla, CA, USA; ^2^Veteran Administration Eastern Colorado Health Care System, Denver, CO, USA; ^3^Adult and Child Consortium for Health Outcomes Research and Delivery Science, School of Medicine, University of Colorado, Aurora, CO, USA

##### **Correspondence:** Borsika Rabin (borsika.a.rabin@gmail.com)

**Background**

Many health systems and implementation science studies have demonstrated the importance of tailoring interventions to the local context to improve fit. By considering local culture, resources, characteristics and preferences, interventions have a better chance to succeed and are more likely to lead to improved outcomes. Hence, there is a growing need for the systematic, parsimonious, and pragmatic documentation of changes or adaptations that happen during the implementation of interventions in various settings. There are currently few instruments and examples of successful adaptation measurement in the field.

**Materials and Methods**

We will present five case studies, four conducted in the context of the Veteran Administrations and one in an academically affiliated health care delivery system, University of California Davies. We will use an overarching framework to assess adaptations.

**Results**

The five case studies are diverse in terms of the conditions addressed, implementation strategies and interventions. They include a nurse coordinator-based transition of care intervention, a data and training driven multimodal pain management project, a cardiovascular patient-reported outcomes project using data sharing and facilitation, and a pharmacist-based chronic care management project. For all five case studies, we used an overarching modified adaptation framework to document changes made to the intervention and implementation strategy compared to that originally proposed. The modified adaptation framework was developed using the framework developed by Stirman and colleagues and was expanded by concepts from the RE-AIM framework. The instrument addresses the intuitive domains of Who, How, When, What, and Why to classify and organize adaptations. For each case study, we will discuss how the modified framework was operationalized, the multiple methods used to collect data and what approaches were utilized to analyze the data. These methods include real time tracking systems, periodic structured interviews at key times during the intervention, and direct observation. Some of these methods are designed to produce rapid information that can inform other assessments in an iterative fashion. We will also provide examples of various categories of adaptations.

**Conclusions**

We will report the utility and helpfulness of these assessments and the overriding adaptations model across the various projects and content areas. Finally, we will make recommendations for the systematic documentation of adaptations in future studies and make our assessment materials available to other researchers.

## A33 Adapting early implementation efforts to local contexts: Development of a transdiagnostic intervention for common adolescent mental health difficulties in Indian schools

### Maya Boustani^1^, Daniel Michelson^2^, Rachana Parikh^3,4^, Aneeha Singh^3^, Resham Gellatly^1^, Bruce Chorpita^1^, Christopher Fairburn^5^, Vikram Patel^3,4,6^

#### ^1^University of California Los Angeles, Los Angeles, CA, USA; ^2^London School of Hygiene and Tropical Medicine, Bloomsbury, London, United Kingdom; ^3^Public Health Foundation of India, Delhi NCR, Gurugram, Haryana, India; ^4^Public Health Foundation of India & Sangath, Delhi NCR, Gurugram, Haryana, India; ^5^Oxford University, Oxford, United Kingdom; ^6^Harvard University, Cambridge, MA, USA

##### **Correspondence:** Maya Boustani (mbous006@fiu.edu)

**Background**

India is home to 20% of the world’s 1.2 billion adolescents, where many are exposed to risk factors for mental disorders. Reaching Indian youth in schools provides a natural opportunity to increase access to services in a non-stigmatizing context. Yet, insufficient resources, lack of a trained workforce and mental health stigma are considerable barriers to successful implementation of mental health care [1]. The current project - “PRIDE” (PRemIum for aDolescents) - aims to address this treatment gap, by developing and testing a scalable transdiagnostic psychological intervention for adolescents.

**Materials and methods**

Initially, the project aimed to develop a single step transdiagnostic treatment for adolescents. However, treatment design evolved significantly in response to (1) expert feedback, (2) qualitative interviews with local stakeholders, including adolescents (n = 124), teachers (n = 65), and mental health staff (n = 22); and (3) unexpected implementation challenges.

**Results**

Implementation challenges, in particular, were numerous and evident from early field testing in nine schools in Delhi (n = 623 student referrals) and Goa (n = 291 student referrals). Administrative concerns such as securing permissions from schools and using translation services, led to significant delays. Demand for services due to academic stress was much higher than anticipated, and led to the creation of an additional universal service. Additional concerns such as widespread literacy problems, affecting usability of a printed workbook; poor access to smartphones and internet, limiting feasibility of digital delivery options; and resistance to deploying female counselors in all-male schools further informed the development and implementation of the program. The program was otherwise well received by school officials, and acceptable to teachers and students - as evidenced by large referral volumes.

**Conclusions**

As a result, the single-step treatment is now a multi-step, comprehensive program with the following architecture:1) universal classroom based group for all youth; 2) guided problem-solving self-help for youth who need additional support after the group (delivered via a printed workbook); 3) face-to-face counseling with a lay counselor for those with symptoms of anxiety, depression, trauma or conduct; 4) referral to a specialist for more severe cases. This project illustrates how community partnerships in underserved global mental health settings inform and impact real- world implementation efforts. Implications for further program development and evaluation are considered.

**Reference**

1. Barker G. Adolescents, social support and help-seeking behaviour: an international review and programme consultation with recommendations for action. WHO Discussion Papers on Adolescence. 2007;978(92):4.

## A34 Stakeholder perspectives on inner- and outer-context factors impacting the implementation of evidence-based strategies to reduce LGBTQ youth suicide

### Cathleen Willging^1^, Amy Green^2^, Mary Ramos^3^, Daniel Shattuck^1^, Lara Gunderson^1^

#### ^1^Pacific Institute for Research and Evaluation, Calverton, MD, USA; ^2^University of California, San Diego, La Jolla, CA, USA; ^3^University of New Mexico, Albuquerque, NM, USA

##### **Correspondence:** Cathleen Willging (cwillging@pire.org)

**Background**

Reducing youth suicide in the United States (U.S.) is a national public health priority, and lesbian, gay, bisexual, transgender, and queer or questioning (LGBTQ) youth are at elevated risk. The Centers for Disease Control and Prevention (CDC) endorses six evidence-based (EB) strategies that center on meeting the needs of LGBTQ youth in schools; however, fewer than 7.6% of U.S. schools implement all of them [1]. Our intervention model builds on the four-phase Exploration, Preparation, Implementation, and Sustainment (EPIS) model [2] and the Dynamic Adaptation Process [3] to implement EB strategies in U.S. high schools.

**Materials and Methods**

As part of a mixed-methods cluster randomized intervention design, implementation readiness interviews were conducted with at least two stakeholders at both intervention (n=18) and control schools (n=18). Interview guides consisted of open-ended questions to examine implementation issues at the system, provider, and student levels, focusing on attitudes toward, access to, and availability of school and community supports for LGBTQ youth, school policies and practices, and organizational factors believed to influence use of the EB strategies. Transcripts were imported into NVivo 11 for iterative coding and thematic analysis.

**Results**

Coding points to ten overarching themes pertaining to factors that affect the preparedness of schools to implement EB strategies to support LGBTQ youth. Outer-context factors include: ^1^socially-conservative community orientations; ^2^lack of local resources; and ^3^district/school policies and practices. Inner-context factors include: ^4^knowledge of and exposure to LGBTQ issues among school staff; ^5^training deficits among school staff; ^6^prevalence of neutrality discourses suggesting that LGBTQ students should not be singled out for “special treatment;” ^7^student attitudes and support; ^8^de facto safe spaces; ^9^health education curricula; and ^10^pragmatic considerations. For pragmatic considerations, participants indicated that efforts to change school climate can be influenced by employee turnover, excessive staff workload and time constraints, the sense that a school already has sufficient supports in place for LGBTQ students and, in some cases, the belief among fellow staff that there are no LGBTQ students attending schools who warrant support/ interventions.

**Conclusions**

These interviews highlight multiple inner- and outer-context factors impacting the ability of schools to implement EB strategies to support LGBTQ youth. This data will be presented to and used by Implementation Resource Teams at participating schools during the Preparation phase to determine: (a) adaptations needed in the school context and its workforce to ensure uptake; and (b) how to accomplish such adaptations.

**References**

1. Demissie Z, Brener ND, McManus T, Shanklin SL, Hawkins J, Kann L. School health profiles 2014: characteris tics of health programs among secondary schools. Atlanta, GA: Centers for Disease Control and Prevention; 2015. https://www.cdc.gov/healthyyouth/data/profiles/pdf/2014/2014_profiles_report.pdf. Accessed 14 March 2017.

2. Aarons GA, Hurlburt M, Horwitz SM. Advancing a conceptual model of evidence-based practice implementation in public service sectors. Admn Policy Ment Health. 2011;38(1):4-23.

3. Aarons GA, Green AE, Palinkas LA, Self-Brown S, Whitaker DJ, Lutzker JR, et al. Dynamic adaptation process to implement an evidence-based child maltreatment intervention. Implement Sci. 2012; 7(1):32.

## A35 Characterizing implementation mechanisms in community effectiveness trials: Applying the EPIS Framework to two large-scale autism trials

### Lauren Brookman-Frazee^1^, Aubyn Stahmer^2^

#### ^1^University of California, San Diego, La Jolla, CA, USA; ^2^University of California, Davis, Davis, CA, USA

##### **Correspondence:** Lauren Brookman-Frazee (lbrookman@ucsd.edu)

**Background**

The two public service systems particularly important for serving school-age children with ASD are education and mental health. Our research groups have used community-partnered approaches to adapt and test behavioral evidence-based interventions (EBI) for autism in these service systems. AIM HI (“An Individualized Mental Health Intervention for ASD” refers to a package of EBI strategies designed to reduce challenging behaviors in children served in mental health service settings. CPRT (“Classroom Pivotal Response Teaching” refers to an EBI adapted for use in classroom settings to target social, communication, and academic skills. AIM HI and CPRT share common methods for developing, adapting, and testing interventions in the community. The purpose of this study is to undertake an in-depth examination of EBI implementation factors using the EPIS framework.

**Materials and Methods**

An independent researcher conducted 9 semi-structured interviews with the intervention developers and experts from both studies across the duration of the projects to gather first-hand accounts of the implementation process. Two focus groups were conducted with research teams’ trainers responsible for providing ongoing training to community providers (MH therapists, school teachers). A focus group guide was structured to gather trainers’ perspectives on barriers and facilitators to provider use of the EBIs and sustainment. Transcripts were analyzed in an iterative process using the “coding, consensus, co-occurrence and comparison” methodology rooted in grounded theory.

**Results**

Many outer and inner context, and intervention factors influenced implementation for both service settings differentially across phases. The preparation/adoption phase was most influenced by the identified factors across all influences and contexts, while influences in the implementation and sustainment phases were more specified. Specific influences including leadership, program, provider and client/student factors will be described across the preparation, implementation and sustainment phases.

**Conclusions**

EBI implementation and sustainment is a complex process involving interactions between intervention developers, and community stakeholders including system, organizations, and providers. The use of the EPIS framework helps to identify and organize both outer and inner context factors that may impact implementation across the phases of the process. AIM HI and CPRT research shares common methods for developing, adapting, and testing interventions and reports similar themes in implementation processes and outcomes, providing a unique opportunity for a cross-service setting comparison of innovative implementation interventions.

## A36 Organizational climate and leadership in implementation of evidence-based interventions for ASD

### Nicole Stadnick, Colby Chlebowski, Lauren Brookman-Frazee

#### University of California, San Diego, La Jolla, CA, USA

##### **Correspondence:** Nicole Stadnick (nstadnic@ucsd.edu)

**Background**

Implementation frameworks highlight the significance of organizational climate and program leadership in promoting the adoption, implementation, and sustainment of evidence-based practice (EBP). This study examined the association between organizational-level climate and leadership characteristics and therapist training outcomes of an EBP implementation effort in children’s mental health (MH) services using data from a large-scale randomized community effectiveness trial of AIM HI (“An Individualized Mental Health Intervention for ASD”).

**Materials and Methods**

AIM HI is a clinical intervention and training protocol to reduce challenging behaviors in children with ASD for delivery by community MH therapists. AIM HI was developed through a community-academic partnership with county MH leaders, therapists and caregivers. Training in AIM HI consisted of an introductory workshop followed by in-person consultations and delivery of AIM HI for 6 months. Participants included 126 MH therapists (85% Female; 35% Hispanic), employed in 16 MH programs in San Diego or Los Angeles County, who participated in the training condition of the effectiveness trial. Therapist report on the Implementation Climate Scale and Implementation Leadership Scale, program type (clinic; school; both), and county were included as predictors. The following training outcomes were examined: 1) Training Engagement (number of completed consultations) and 2) Therapist report of protocol delivery (number of AIM HI protocol steps completed).

**Results**

Two multilevel (therapists nested within programs) models were specified to predict each training outcome. Results indicated there were county differences in training engagement (B = 1.96, p <.05). Rewards for EBP use had a marginal negative association with both training engagement (B = -.46, p = .05) and training completion (B = -.69, p =.05). Finally, there was a positive trend towards educational support for EBPs and training engagement (B =.92, p=.06). Program type and therapist perceptions of leadership qualities were not predictive of training engagement or completion.

**Conclusions**

Findings suggest that aspects of implementation climate were associated with therapist training completion and engagement, although not always in facilitative directions. Specifically, implementation climates in which tangible or fiscal rewards for EBP use are provided may be unnecessary but offering opportunities for EBP education and training may facilitate therapist training engagement. County differences in training engagement may be explained by these implementation climate findings. Results have implications for selection of key elements of implementation climate to evaluate or modify to maximize therapist training engagement and completion.

## A37 Mapping leadership structures in special education programs to tailor leadership intervention

### Jessica Suhrheinrich^1^, Kelsey Dickson^2^

#### ^1^San Diego State University, San Diego, CA, USA; ^2^University of California, San Diego, La Jolla, CA, USA

##### **Correspondence:** Jessica Suhrheinrich (jsuhrheinrich@mail.sdsu.edu)

**Background**

Although evidence-based practices (EBPs) for children with autism spectrum disorder (ASD) exist, current methods for selecting, implementing and sustaining these practices in community school settings are not effective. Teachers use practices with and without research support about equally with children of varied disabilities and there is very limited evidence to indicate barriers and facilitators to implementation in this unique context. Leadership across organizational levels is indicated as an important factor in acquiring and using evidence, which suggests need for further exploration of the leadership structure within school-based services for ASD and how it can serve to facilitate a context that supports implementation and use of EBPs for ASD.

**Materials and Methods**

To explore the leadership structure within school-based services for ASD and the effect on implementation processes, a 63 item School Leadership Survey was developed. The survey included the Implementation Leadership Scale [1] demographics, and questions regarding specific roles and responsibilities of personnel across leadership levels across stages of implementation. A subset of the questions were analyzed for this presentation.

**Results**

First-level leaders (e.g., program specialists, school psychologists, etc.) working in school-based programs participated in the School Leadership Survey (n=214). Preliminary analyses indicate 80% of participants report leaders within their districts are at least moderately involved in addressing factors impacting the implementation of EBPs (e.g., developing a plan, removing obstacles). Further, involvement varies as a function of district size, with decreased personnel involvement in larger districts. Across district sizes, mid-level leaders or specialists are most actively involved in providing training in new interventions whereas both mid and high level leaders are most actively involved in deciding how schools will implement new educational interventions.

**Conclusions**

This preliminary analysis provide early understanding of leadership influence on implementation of ASD services in schools. Identification of key leadership factors that influence successful implementation and sustainment of EBP will impact the quality of educational programming for students with ASD. Future analyses will integrate qualitative measures (focus groups) and will explore relationships between organizational characteristics (size, rural/urban location, student demographics of school district) and participants’ ratings of implementation leadership practices.

**Reference**

1. Aarons GA, Ehrhart MG, Farahnak LR. The implementation leadership scale (ILS): development of a brief measure of unit level implementation leadership. Implement Sci. 2014;9:45.

## A38 Testing multi-level implementation model for translating evidence-based interventions for ASD (TEAMS): Methods and interventions

### Aubyn Stahmer^1^, Lauren Brookman-Frazee^2^

#### ^**1**^University of California, Davis, Davis, CA, USA; ^**2**^University of California, San Diego, La Jolla, CA, USA

##### **Correspondence:** Aubyn Stahmer (astahmer@ucdavis.edu)

**Background**

Data from AIM HI and CPRT studies support the effectiveness of ASD EBI for improving child outcomes only when providers complete training and deliver interventions with fidelity. Unfortunately, adoption and provider training outcomes, considered key implementation outcomes are variable. These findings are especially concerning given the link between fidelity and child outcomes. Testing methods of improving implementation outcomes is key to ensuring positive child-level outcomes when EBI are implemented in routine care. Based on the data from the independent effectiveness studies indicating that provider attitudes and implementation leadership are promising targets of implementation interventions our groups are now initiating two, coordinated studies testing the effectiveness of the “Translating Evidence-based Interventions (EBI) for ASD: Multi-Level Implementation Strategy” (TEAMS) model (R01MH111950 and R01MH111981).

**Materials and Methods**

These studies use a randomized implementation/effectiveness Hybrid, Type 3, trial to test the TEAMS model with the AIM HI in publicly-funded mental health services and CPRT intervention in education settings. A dismantling design will be used to understand the effectiveness of TEAMS and the mechanisms of change (Leadership Training & Provider Engagement Strategies) across settings and participants. We will randomize 37 mental health programs and 37 school districts to one of 4 treatment conditions (usual training (UT); UT + leadership training; UT + provider engagement; all 3 elements). We anticipate enrolling 600 providers and children over 4 years. Implementation out- comes including provider training completion, fidelity and child behavior change will be examined.

**Results**

We will present relevant results from our initial trials indicating variable provider fidelity outcomes. Approximately 16% of providers in both groups did not complete training and 27% did not meet fidelity of implementation criteria. Providers in programs/districts with stronger leadership support, and provides with better attitudes toward EBI were more likely to have higher fidelity and sustainment. By the time of the conference will present initial enrollment data and initial response to the leadership intervention for TEAMS.

**Conclusions**

Implementation support is needed to facilitate access to quality care. ASD interventions are typically complex, require decision making based on the significant heterogeneity of the condition and must be integrated with other strategies. Therefore, examining multi-level implementation interventions has the potential to further increase the impact of implementing ASD EBI in community settings by increasing the effectiveness of provider uptake of EBI, thereby improving child outcomes.

## A39 Political party, ideology, and variations in research dissemination preferences and research use practices among US state legislators

### Jonathan Purtle^1^, Elizabeth Dodson^2^, Ross Brownson^2^

#### ^1^Drexel University Dornsife School of Public Health, Philadelphia, PA, USA; ^2^Washington University in St. Louis, Brown School of Social Work, St. Louis, MO, USA

##### **Correspondence:** Jonathan Purtle (jpp46@drexel.edu)

**Background**

State legislators (i.e., elected state policymakers) influence the context of health service delivery through the budgetary and regulatory decisions they make. These decisions can become evidence-informed through research dissemination strategies that are tailored to legislators’ individual characteristics. Political party and ideology are known to influence legislators’ policy decisions, but little is known about whether these characteristics should be considered in the design of legislator-focused dissemination strategies. The study aims were to determine if and how research dissemination preferences and research use practices differ between US state legislators with different political party affiliations and varying social and fiscal ideologies.

**Materials and Methods**

A telephone-based, cross-sectional survey of 862 state legislators (response rate 50.4%) was conducted in 2012. Research dissemination preferences and research use practices were measured using 31 Likert scale items that have been previously validated with state legislators [1]. Social and fiscal ideologies were assessed on Likert scales and political party information was publicly available. Non-parametric tests examined differences in research dissemination preferences and research use practices between Democrats and Republicans, Spearman correlation coefficients were produced to examine differences by social and fiscal ideology, and multiple linear regression analyses were conducted to control for other legislator characteristics.

**Results**

Compared to Republicans, Democrats assigned a higher priority rating to eight-of-twelve features of disseminated research—such as research being presented in a concise way (p=.001) and delivered by someone the legislator knows (p=.004). Republicans and Democrats did not, however, significantly differ in their research use practices or level or trust in research from different sources. The more ideologically conservative a legislator was the more they trusted research from industry, their constituents, and other legislators. This positive correlation was the strongest for industry (social ideology score: r=.334, p<.001; fiscal ideology score: r=.287; p<.001). Conversely, the more conservative a legislator was the less they trusted research from government agencies (social ideology score: r=.-394, p<.001; fiscal ideology score: r=.-357, p<.001) and universities (social ideology score: r=.-290, p<.001; fiscal ideology score: r=.-289, p<.001).

**Conclusions**

Compared to Republicans, Democrat legislators have somewhat different and slightly stronger preferences for disseminated research. Independent of political party affiliation, legislators’ trust in research from difference sources varied significantly according to their social and fiscal ideologies. Political party affiliation and ideology are characteristics that should be considered in the design of legislator-focused dissemination strategies.

**Reference**

1. Bogenschneider K, Johnson K. Policymakers’ use of social science research. J Marriage Fam. 2013;75(2):263-75.

## A40 Adapting the stages of implementation completion measure for health policy interventions

### Jennifer Leeman^1^, Allison Myers^2^, Kathleen Knocke^1^, Mian Wang^1^

#### ^1^University of North Carolina, Chapel Hill, NC, USA; ^2^Counter Tools, Carrboro, NC, USA

##### **Correspondence:** Jennifer Leeman (jleeman@email.unc.edu)

**Background**

A central limitation of research on policy implementation strategies is the focus on policy enactment as the sole measure of effectiveness. In many cases, policy enactment is uncertain, may take years to achieve, and results from multiple factors, thereby limiting efforts to attribute enactment to the effects of implementation strategies. Measuring intermediate outcomes overcomes these challenges by providing interim markers of community partnerships’ progress on the road to policy enactment and by providing ongoing feedback to motivate, strengthen, and sustain partnerships throughout the policy change process. Measuring intermediate outcomes also advances understanding of the mechanisms through which policy implementation strategies have their effects, understanding critical to optimizing strategies’ effectiveness. We created and piloted a Policy Change Process Completion (PCPC) measure of the intermediate outcomes (mechanisms) through which policy implementation strategies affect policy enactment. The PCPC is modeled on the Stages of Implementation Completion (SIC) measure, which documents completion of activities within each stage of an implementation process. We studied policy change processes within the context of implementation strategies to promote policy to counter tobacco marketing in retail environments.

**Materials and Methods**

Applying approach used to develop SIC, we identified activities required to complete each policy change process via literature review and in-depth interviews with public health and other professionals (*n*=30) working on tobacco retail policy in one mid-western state. We translated activities into a structured interview guide and pilot tested it with tobacco control partnerships (n=30) in one southeastern state at 6-months (n=26, 86.7% response) and 12-months (n=30, 100% response) following receipt of implementation strategies. This summer, we will administer PCPC to 90 additional partnerships and assess reliability and validity of measure’s five constructs.

**Results**

The measure assesses 24 activities within five core policy change processes: (1) engage partners, (2) document local problem, (3) formulate evidence-informed solution, (4) raise awareness of problem and solution, and (5) persuade decision makers to enact new policy. In the pilot test, we achieved 95% interrater-reliability for agreement about task completion and proportion of activities completed within each stage varied across partnerships. We also will report findings from analysis of constructs.

**Conclusions**

Additional research is planned to further assess validity and reliability and whether higher completion scores predict policy enactment. The measure has potential to identify gaps in performance and tailor policy implementation strategies and utility is evaluating implementation strategy effectiveness not only for tobacco retail policy but also for other health policies.

## A41 Breaking down evidence-based practices for state policy: Using a common elements approach in progress note documentation as an indicator of adherence

### Sarah Walker^1^, Georganna Sedlar^1^, Jessica Leith^1^, Lucy Berliner^1^, Paul Davis^2^, Eric Trupin^1^

#### ^**1**^University of Washington, Seattle, WA, USA; ^2^Washington State Department of Social and Health Services, Arlington, WA, USA

##### **Correspondence:** Sarah Walker (secwalkr@uw.edu)

**Background**

In 2012, the Washington State legislature directed the children-focused divisions of the Department of Social and Health Services to “substantively” increase their respective investment in research and evidence-based practices. The legislation simultaneously directed two state research entities to develop an inventory of practices that would be eligible for counting. This list contained both name brand programs as well as categories of effective approaches identified through meta-analysis - for example, Cognitive Behavior Therapy for Anxious Children. Subsequently, the Evidence-Based Practice Institute (EBPI) was asked by the state to develop guidelines for reporting evidence-based practices within children’s mental health Medicaid services. In order to balance rigor with flexibility, the EBPI proposed guidelines that specify appropriate training, consultation and progress note documentation that allow providers to report an EBP even in the absence of active consultation as long as notes conform to “essential” and “allowable” elements of a treatment category. The elements were derived from the distillation and matching model [1], meta-analytic studies and reviews of dismantling studies. The following study examined the accuracy of EBP reporting prior to implementation of the guides and baseline attitudes towards evidence-based practices among providers in one children’s mental health service agency.

**Materials and Methods**

Evidence-based practice data from state administrative records was obtained for the year prior to the release of the first version of the guides. This included reviewing more than 40,000 reported encounters for children’s mental health, Medicaid services in Washington from April 2015 through March 2016 and calculating rates of evidence-based practices for all eligible encounters. A companion, qualitative study assessed the baseline perceptions of evidence-based practices in one mental health agency receiving targeted technical assistance for using the Reporting Guides.

**Results**

Analysis of the accuracy of evidence-based practice reporting found error rates among regional healthcare authorities ranging from 9-83% based on definitions adopted in the guides for eligible encounters and programs. Qualitative analyses of baseline attitudes towards evidence-based practices suggested providers were primarily interested in practices that clearly fit with the needs of their clientele, required relatively low training burden, and/or strengthened connections with respected experts.

**Conclusions**

Requiring providers to self-report evidence-based practices using modifiers in billing codes is feasible but will requires clear and specific definitions for providers concerning 1) which encounters are eligible for EBP reporting; 2) when a clinical activity in session is eligible for reporting. Further, analysis at the state level will required clear instructions for which encounters are eligible to include in calculation of rates. Without such direction, administrative data is likely to misrepresent the true penetration of EBPs in public mental health services.

**Reference**

1. Chorpita BF, Daleiden EL. Mapping evidence-based treatments for children and adolescents: application of the distillation and matching model to 615 treatments from 322 randomized trials. J Consult Clin Psychol. 2009;77(3);566-79

## A42 Why is external facilitation effective as an implementation strategy? Evidence for tailoring to the learning capacity of primary care practices

### Michael Parchman, Clarissa Hsu, Katie Coleman

#### Kaiser Permanente Washington Health Research Institute, Seattle, WA, USA

##### **Correspondence:** Michael Parchman (parchman.m@ghc.org)

**Background**

External Facilitation as an implementation strategy is “a process of interactive problem solving and support that occurs in a context of a recognized need for improvement and a supportive interpersonal relationship” [1]. One mechanism proposed for why it is effective is the ability to tailor the support to the absorptive or learning capacity of the clinical setting, that is the practice’s ability to recognize the value of new knowledge, assimilate it, and apply it to patient care [2]. Here we examine evidence of tailoring of support by facilitators to match the absorptive capacity of primary care practices for the purpose of building their quality improvement (QI) ability.

**Materials and Methods**

Healthy Hearts Northwest (H2N) is trial to expand QI capacity within smaller primary care practices across Washington, Oregon and Idaho. Practice facilitators support 10-20 practices with quarterly in-person visits and phone calls in between. During an initial visit, the facilitator meets with the practice team to develop consensus responses to 20 questions about current QI capacity. This Quality Improvement Capacity Assessment (QICA) survey assesses QI capacity in 7 domains: embedding clinical evidence, using data, establishing a QI process, population management, defining team roles, self-management support, and community resources. Facilitators also document number and type of topics discussed after each contact with the practice. Here we examined the association between baseline QICA scores and the number and type of topics discussed during and subsequent to the initial visit. We also draw on the notes generated by the facilitators after every encounter with a practice and focus group data from facilitators.

**Results**

209 practices are enrolled. The mean QICA score was 6.52 (SD 1.45, range 3.3 to 10.8) Total topics discussed ranged from zero to 26 with a mean of 5.39 (SD 5.08) A greater number of topics were discussed in practices with higher total QICA scores. (9.0 versus 7.9, *p* < 0.01) The number of health information technology topics discussed was correlated with practice capacity to use data; the number of QI topics discussed was correlated with capacity regarding team roles and functions. Comments from facilitators provide further insight into how QICA results were used in tailoring their work support.

**Conclusions**

Tailoring support to the absorptive/learning capacity of an individual practice provides additional insight into why practice facilitation as an implementation support mechanism is effective within the primary care setting.

**References**

1. Powell BJ, Waltz TJ, Chinman MJ, Damschroder LJ, Smith JL, Matthieu MM, Proctor EK, Kirchner JE. A refined compilation of implementation strategies: results from the Expert Recommendations for Implementing Change (ERIC) project. Implement Sci. 2015;10:21.

2. Berta W, Cranley L, Dearing JW, Doghety EJ, Squires JE, Estabrooks CA. Why (we think) facilitation works: insights from organizational learning theory. Implement Sci. 2015;10:141.

## A43 Time and cost of “extreme” implementation facilitation to address challenging clinical contexts

### Mona J. Ritchie^1,2^, Chuan-Fen Liu^3,4^, James C. Townsend^5^, Jeffery A. Pitcock^1^, JoAnn E. Kirchner^1,2^

#### ^1^VA Quality Enhancement Research Initiative (QUERI) Program for Team-Based Behavioral Health, Department of Veterans Affairs, Washington DC, USA; ^2^Department of Psychiatry, University of Arkansas for Medical Sciences, Little Rock, AR, USA; ^3^Center of Innovation for Veteran-Centered and Value-Driven Care, VA Puget Sound Health Care System, Seattle, WA, USA; ^4^Department of Health Services, University of Washington, Seattle, WA, USA; ^5^Center for Mental Healthcare and Outcomes Research, Central Arkansas Veterans Healthcare System, Little Rock, AR, USA

##### **Correspondence:** Mona J. Ritchie (Mona.Ritchie@va.gov)

**Background**

Implementation facilitation (IF) has shown promise for fostering uptake of evidence-based innovations [1]. However, we know little about the organizational costs of utilizing IF, particularly in clinical sites with very challenging contexts. We applied an “extreme” external and internal facilitation strategy that supported successful implementation of primary care-mental health integration at 8 challenged primary care clinics in two VA networks [2]. The strategy included the transfer of IF knowledge and skills to the clinical organization to support future implementation efforts. We examine the level of facilitator and clinical personnel effort and variation in time and cost across these networks.

**Materials and Methods**

We followed one expert external facilitator (EF) and two internal regional facilitators (IRFs). Facilitators engaged and involved over 350 VA personnel at all organizational levels in implementation efforts. We documented facilitators’ and clinical personnel time, personnel information, and types of IF activities across participating sites using a structured spreadsheet collected from facilitators on a weekly basis. We obtained travel costs from project records and salary information from publicly available web portals. We conducted descriptive analysis of time data and estimated the organizational cost of applying IF.

**Results**

The EF devoted 441 hours across all 8 clinics over 28 months working similar amounts of time in each network, although time varied across clinics. Travel accounted for more of the EF’s time than any other activity. Time devoted to IF by the two IRFs varied in total amount (1,963 hours versus 1,442 hours). Preparation and planning accounted for IRFs’ time more than any other activity (39.18% and 36.47%). Clinical personnel across all organizational levels participated in IF activities. A higher number of personnel participated in IF activities in one of the networks. Although the cost of providing IF was similar in both networks ($198,303 versus $198,416), total organizational costs were higher in the network with higher clinical personnel participation ($320,068 versus $297,744). IRFs in this study operationalized IF in different ways due to both organizational context and IRF style resulting in variations in both time and cost.

**Conclusions**

Although extreme IF in challenged healthcare settings can improve evidence-based program implementation and build capacity for future implementation efforts, it requires substantial organizational investments that may vary by site and implementation effort. Given the organizational costs, it is important to identify sites that are likely to benefit from IF, tailor IF intensity to local needs, and apply very intensive strategies for only the most challenging contexts.

**References**

1. Baskerville NB, Liddy C, Hogg W. Systematic review and meta-analysis of practice facilitation within primary care settings. Ann Fam Med. 2012;10:63-74.

2. Kirchner JE, Ritchie MJ, Pitcock JA, Parker LE, Curran GM, Fortney JC. Outcomes of a partnered facilitation strategy to implement primary care-mental health. J Gen Intern Med. 2014;29(Suppl 4):904-12. doi:10.1007/ s11606-014-3027-2

## A44 Using external and internal facilitation as implementation strategies for improving delivery of a collaborative care model in 45 community-based practices: Evidence from the ADEPT study

### Shawna N. Smith^1,3,4^, Julia Kyle^1^, Mark Bauer^5,6^, Daniel Eisenberg^7^, Michelle Barbaresso^1^, Celeste Liebrecht^1^, Katherine Prenovost^1^, Amy M. Kilbourne^1,2^

#### ^1^University of Michigan, Department of Psychiatry, Ann Arbor, MI, USA; ^2^VA Center for Clinical Management Research, Fairfax, VA, USA; ^3^University of Michigan, Institute for Social Research, Ann Arbor, MI, USA; ^4^University of Michigan, Department of Internal Medicine, Ann Arbor, MI, USA; ^5^Harvard University, Department of Psychiatry, Cambridge, MA, USA; ^6^VA Center for Healthcare Organization and Implementation Research, Washington DC, USA; ^7^University of Michigan, Department of Healthcare Management and Policy, Ann Arbor, MI, USA

##### **Correspondence:** Shawna N. Smith (shawnana@umich.edu)

**Background**

While evidence-based collaborative care models (CCMs) can improve mental and physical health outcomes in patients with mood disorders, barriers in resources, leadership support, and payment models can stymie implementation in community-based practices. Facilitation is an implementation strategy wherein change agents aid implementation efforts through guided problem solving. External facilitators (EF) can be partnered with Internal Facilitators (EF+IF), or site-native advocates, to address barriers and improve uptake. However, few studies have attempted widespread facilitation in community-based practices, or examined content or effectiveness of EF and EF+IF.

**Materials and Methods**

45 community-based practices were randomized to receive either EF (k=22) or EF+IF (k=23) after initially failing to provide a CCM to 10+ patients under minimal implementation support. EFs logged all site interactions, categorizing mode, duration, and content. Bivariate analyses and multivariable negative binomial models examine differences in EF interaction length and content, improvement in CCM delivery to patients, and effect of study arm and facilitation time and content on CCM delivery.

**Results**

EFs logged 1,037 interactions across all sites between January 2015 and October 2016, with a median interaction time of 36 minutes per site-month (IQR: 25-43). 64% of interactions were done via email and 34% via phone. EF site interactions (N=564) involved site administrators (31%), supervisors (29%) and providers (20%); EF+IF site interactions (N=473) were predominantly with the IF. EF and EF+IF mean interaction times did not differ (t=0.47, p=0.64), but content did. EF interactions focused more on education (EF: 51%; EF+IF: 45%) while EF+IF interactions focused on strategy development (EF: 7%; EF+IF: 16%) and reinforcement (EF: 23%; EF+IF: 35%). After 6 months, EF sites averaged 7.9 patients receiving the CCM (up from 4.25) and EF+IF sites averaged 5.5 patients (up from 2.5). In multivariable models, however, study arm (B=0.55, 95% CI=-0.76, 1.87), total interaction time (b=0.04; 95% CI=-0.01, 0.09) and strategic time (B=-0.17; 95% CI=-0.40, 0.04) were not significant predictors of improvement in uptake.

**Conclusions**

ADEPT successfully used facilitation strategies at 45 community-based practices to improve delivery of a CCM to patients with mood disorders. Both EF and EF+IF improved CCM delivery in community based practices. Data on EF interactions provide insight into the mechanisms of facilitation on implementation success. Specifically, EF activity becomes more strategic when augmented with IF. Although neither total EF time nor strategic time was associated with increased CCM delivery to patients, future work will explore longer-term comparative effectiveness on uptake and patient mental health outcomes.

## A45 A systems-contextual evaluation of implementation of an evidence-based parenting intervention within a public health framework

### Suzanne Kerns^1,2^, Cathea Carey^2^, Jessica Leith^2^, Victor Rodriguez^3^, Sebrena Chambers^3^, Scott Waller^4^

#### ^**1**^University of Denver, Denver, CO, USA; ^2^University of Washington, Seattle, WA, USA; ^3^Tacoma Pierce County Public Health, Tacoma, WA, USA; ^4^Washington State Division of Behavioral Health and Recovery, Olympia, WA, USA

##### **Correspondence:** Suzanne Kerns (suzanne.kerns@du.edu)

**Background**

The Triple P Positive Parenting Program (Triple P) is a population based approach designed to enhance parenting skills and reduce child maltreatment [1, 2]. This intervention has demonstrated population-level impacts on child welfare indicators and a small randomized trial of pediatric residents who used Triple P with families in a primary care context found beneficial effects for families [3]. A prior study examined the systems-contextual factors associated with timely implementation of Triple P within three rural communities in Washington State [4]. The present study examines the replicability of prior findings to a different implementation context and expands upon prior evaluations by examining the utility of social network analyses in understanding implementation impacts. We emphasize the impact of this initiative on primary care physicians integrating parenting interventions into practice.

**Materials and Methods**

94 individuals participated in the Triple P trainings. Forty-four individuals (47%) responded to baseline and 6-month follow-up questionnaires. These individuals reported on their overall use of the Triple P model, their attitudes towards EBPs in general, their perceptions of the acceptability and feasibility of Triple P, network communication and collaboration and the availability of referral networks and other supports for implementation. Facilitators and barriers to implementation were collected qualitatively. For the social network analysis, representatives from 13 different agencies responded to baseline and follow-up questionnaires focused on the extent to which they share referrals and resources with other participating agencies and perceptions of collaborative capacity.

**Results**

Practitioners were highly satisfied with Triple P and the training was effective in increasing practitioner self-efficacy in delivery of the parenting intervention. Over half of the trained practitioners went on to deliver the intervention. Home visitors and behavioral health practitioners were more likely to use the program compared with primary care physicians and those in non-traditional settings (e.g., librarians). A network analysis revealed that the initiative had a positive impact in creating additional referral pathways within the Tacoma area.

**Conclusions**

Overall, evaluation of this initiative revealed improvements in individual factors associated with implementation, compared with a similar project in rural communities. However, absolute levels of implementation were not substantially improved. Recommendations include exploring additional ways to support implementation within primary care settings and continuing to trouble shoot with agencies who have struggled to fully embed Triple P within their suite of services.

**References**

1. Prinz RJ, Sanders MR, Shapiro CJ, Whitaker DJ, Lutzker JR. Population-based prevention of child maltreatment: The US Triple P system population trial. Prev Sci. 2009;10(1):1-2.

2. Sanders MR. Triple P-Positive Parenting Program: Towards an empirically validated multilevel parenting and family support strategy for the prevention of behavior and emotional problems in children. Clin Child Fam Psychol Rev. 1999;2(2):71-90.

3. McCormick E, Kerns SE, McPhillips H, Wright J, Christakis DA, Rivara FP. Training pediatric residents to provide parent education: a randomized controlled trial. Acad Pediatr. 2014;14(4):353-60.

4. Kerns SEU, McCormick E, Negrete A, Carey C, Haaland W, Waller S. Walking the talk: factors associated with practitioners’ initial use of an evidence-based parenting intervention following training (under review).

## A46 Testing the Getting To Outcomes implementation support strategy to facilitate the use of an evidence-based practice in VA homeless programs

### Matthew Chinman^1^, Sharon McCarthy^2^, Gordon Hannah^2^, Thomas Byrne^3^, David Smelson^3^

#### ^**1**^RAND Corporation, Santa Monica, CA, USA; ^2^Pittsburgh VA Medical Center, Pittsburgh, PA, USA; ^3^VA National Center on Homelessness Among Veterans, Washington DC, USA

##### **Correspondence:** Matthew Chinman (chinman@rand.org)

**Background**

Evidence-based treatment for co-occurring mental health and substance abuse disorders is needed within Veteran housing programs, but has been challenging to implement. This study tests the impact of an implementation strategy called Getting To Outcomes (GTO) on how well teams in Housing and Urban Development - Veterans Affairs Supportive Housing programs (HUD-VASH) incorporate a treatment called Maintaining Independence and Sobriety Through Systems Integration, Outreach and Networking - Veterans Edition (MISSION-Vet).

**Materials and Methods**

Over two years, this Hybrid Type III, cluster-randomized controlled trial assessed the impact of GTO over and above MISSION-Vet Implementation as Usual (IU) with case managers and peer specialists across three HUD- VASH teams (GTO n=35; IU n=22). Within each team, existing sub-teams (case managers and Veterans) were the clusters randomly assigned. Both conditions received standard MISSION-Vet training and manuals. The GTO group received an implementation manual, training, technical assistance, and data feedback. The trial assessed MISSION-Vet services, implementation barriers and facilitators (via semi-structured interview), and Veteran outcomes rated by case managers.

**Results**

Zero IU case managers initiated MISSION-Vet; 68% in the GTO group did, with 81 Veterans. 7% of Veterans in the GTO group received at least one MISSION-Vet session. Veteran substance abuse, housing, and mental health outcomes did not differ between the GTO and a matched comparison group from control sub-teams. The number of case manager contacts with Veterans was significantly higher among Veterans in the GTO group (B = 2.30, p = .04). Veterans in the GTO group who received higher intensity MISSION-Vet services had less alcohol and drug use, inpatient hospitalization and emergency department use approaching statistical significance. Most case managers interviewed appreciated the MISSION-Vet materials and felt that GTO supported the use of MISSION- Vet. They also reported several significant implementation barriers including the time required for MISSION-Vet, lack of leadership support, and competing priorities.

**Conclusions**

Despite numerous challenges, GTO was able to support the launch of a new evidence-based, co-occurring disorders treatment in a VA housing program. While multiple implementation facilitators are always needed for successful execution of a complex evidence-based program like MISSION-Vet, the GTO implementation strategy could be a model for launching such practices in VA.

**Reference**

1. Chinman M, McCarthy S, Hannah G, Byrne TH, Smelson DA. Using Getting To Outcomes to facilitate the use of an evidence-based practice in VA homeless programs: a cluster randomized trial of an implementation support strategy. Implement Sci. 2017;12:34. doi 10.1186/s13012-017-0565-0

## A47 Systems consultation: A novel implementation strategy for adopting clinical guidelines for opioid prescribing in primary care

### Andrew Quanbeck (arquanbe@wisc.edu)

#### University of Wisconsin-Madison, Madison, WI, USA

**Background**

This NIH-funded research reports on the feasibility, acceptability, and effectiveness of an innovative implementation strategy named “systems consultation” aimed at improving adherence to clinical guidelines for opioid prescribing in primary care (1R34 DA036720-01A1). While clinical guidelines for opioid prescribing have been developed, they have not been widely implemented, even as opioid abuse reaches epidemic levels. We tested a novel implementation strategy consisting of 3 key innovations. First: We engaged a multi-disciplinary panel of world-class experts in medicine, implementation science, and systems engineering in a systematic decision-making technique designed to produce a checklist-based implementation guide. Second: we trained and deployed physician peer consultants to work with clinics to in implementing the streamlined guidelines. Third, we supplied clinics with evidence-based implementation tools from systems engineering (e.g., flowcharting, Plan-Do-Study-Act change cycles) that were used to modify workflows and facilitate adoption of the streamlined guidelines.

**Materials and Methods**

The study compares 4 intervention clinics to 4 control clinics in a randomized matched-pairs design. Each systems consultant aided clinics on implementing the guidelines during a 6-month intervention comprised of monthly site visits and videoconferences. The mixed-methods evaluation employs the RE-AIM (Reach, Effectiveness, Adoption, Implementation, Maintenance) framework. Quantitative outcomes are reported using difference-in-differences analysis. Qualitative methods primarily included ethnographic field techniques.

**Results**

Feasibility. Seven clinics were approached to recruit 4 intervention clinics (3 clinics declined, citing “lack of time”). Each clinic designated a project team consisting of 6-8 staff members, each with at least 1 primary care physician and 1 RN, MA/LPN, and administrative staff member. Attendance at intervention meetings was 88%. Acceptability. More than 80% of staff respondents agreed/strongly agreed with the statements: “I am more familiar with guidelines for safe opioid prescribing” and “My clinic’s workflow for opioid prescribing is easier.” Effectiveness. Urine drug screening rates among intervention clinics increased 3.6% per month over six months; control clinics increased by 0.1% (p=0.01). Mental health screening rates among intervention clinics increased 6.4% per month; control clinics increased by 3.8% (p=0.02). Qualitatively, intervention clinics reported that chronic pain was now treated using approaches similar to those employed for other chronic conditions that are hallmarks of effective primary care, including hypertension and diabetes.

**Conclusions**

The systems consultation implementation strategy demonstrated feasibility, acceptability, and effectiveness in a study of 8 primary care clinics. This multi-disciplinary strategy holds potential to mitigate the prevalence of opioid addiction and ultimately may help to improve implementation of clinical guidelines across healthcare.

## A48 Practice based implementation network: Facilitating psychological health clinical practice change

### Kate McGraw^1^, Robert Ciulla^2^

#### ^1^Deployment Health Clinical Center, Defense Centers of Excellence for Psychological Health and Traumatic Brain Injury, Arlington County, VA, USA; ^2^Mobile Health Program, T2, Defense Centers of Excellence for Psychological Health and Traumatic Brain Injury, Arlington County, VA, USA

##### **Correspondence:** Kate McGraw (adam.k.civ.walsh@mail.mil)

**Background**

According to an Institute of Medicine (2014) report, two decades may pass before psychological health research findings become part of routine clinical practice [1]. In 2012 the Department of Defense (DoD) in conjunction with the Department of Veterans Affairs (VA) began to develop a Practice Based Implementation (PBI) Network to more rapidly translate psychological health research findings into clinical practice by facilitating practice change. The PBI Network is based on the implementation science model Promoting Action on Research in Health Service to: (1) accomplish effective implementation of psychological health evidence-based practices (EBPs); (2) pilot practice change initiatives at military behavioral health clinics; (3) identify the impact of implementation barriers and solutions; and (4) inform military Services and stakeholders on effective processes to implement the practice change prior to dissemination throughout the DoD and VA [2-7].

**Materials and Methods**

The PBI Network presents EBPs to clinicians through trainings that respect clinic culture and context while providing continuous support and facilitation to pilot sites. By engaging in these overarching objectives and actions, the Network also increases provider knowledge and accountability, promotes coordination and information sharing, and potentially reduces costs by testing implementation initiatives prior to broader dissemination throughout the enterprise. Additionally, the PBI Network has an online website that serves as both a repository and resource to support practice changes, allowing DoD and VA providers to share resources, materials and lessons learned.

**Results**

The DoD PBI Network has 14 sites across DoD, and from 2013 to 2016 piloted the implementation of outcomes monitoring of Posttraumatic Stress Disorder treatment, and Substance Abuse Brief Intervention and Referral to Treatment (SBIRT) for alcohol misuse screening.

**Conclusions**

PBI Network demonstrated more rapid translation of research into clinical practice, as well as sustained practice change post-facilitation. In 2017, the next PBI Network pilot will facilitate clinician use of technology in clinical care, in partnership with the VA National Center for PTSD, and the National Center for Telehealth and Technology. This presentation will discuss the implementation and sustainment of the PBI Network, demonstrate the processes and science that have served as the PBI Network foundation, and explore the newest project which is underway. Attendees will learn how to promote psychological practice change within the clinical setting.

**References**

1. Institute of Medicine. Treatment for posttraumatic stress disorder in military and veteran populations: final assessment. Washington, DC: The National Academies Press; 2014.

2. Department of Veterans Health Administration, Health Services Research & Development, Quality Enhancement Research Initiative. Implementation Guide. Washington, DC; 2013. http://www.queri.research.va.gov/implementation/ImplementationGuide.pdf.

3. Department of Veterans Health Administration. Health Services Research & Development Service. Retrieved from: http://www.queri.research.va.gov/implementation/ImplementationGuide.pdf.

4. Helfrich CD, Li YF, Sharp ND, Sales AE. Organizational readiness to change assessment (ORCA): development of an instrument based on the Promoting Action on Research in Health Services (PARIHS) framework. Implement Sci. 2009;4(38):38.

5. McHugh RK, Barlow DH. The dissemination and implementation of evidence-based psychological treatments: a review of current efforts. Am Psychol. 2010;5(2):73-84.

6. Ruzek JI, Karlin BE, Zeiss A. Implementation of evidence-based psychological treatments in the Veterans Health Administration. In McHugh RK, Barlow DH (Eds.), Dissemination of evidence-based psychological treatments. New York, NY: Oxford University Press; 2012. p. 78-96.

7. Stetler CB, Damschroder LJ, Helfrich CD, Hagedorn HJ. A guide for applying a revised version of the PARIHS framework for implementation. Implement Sci. 2011;6(1):99.

8. Stetler C, Mittman B, Francis J. Overview of the VA quality enhancement research Initiative (QUERI) and QUERI theme articles: QUERI series. Implement Sci. 2008;3(8):8-18.

## A49 A public health approach: Translation and implementation of non-clinical practices for suicide prevention in the military

### Adam Walsh, Laura Neely

#### Defense Suicide Prevention Office, Arlington, VA, USA

##### **Correspondence:** Adam Walsh (adam.k.walsh.civ@mail.mil)

**Background**

The Defense Suicide Prevention Office (DSPO) translation and implementation of public health initiatives are based in the Institute of Medicine (IOM) model.

**Materials and Methods**

The process starts with a 360° review of the evidence and gap analysis including the best available research evidence as well as contextual and experiential evidence.

**Results**

From this, strategies on the prevention end of spectrum (universal, indicated, selected) are identified as foci for DSPO efforts to reduce suicide risk in military public health settings including with peers, leadership, clergy, etc. This presentation will describe this translation and implementation model and illustrate it with two large suicide prevention initiatives: the use of social media to predict suicide risk and a means safety campaign.

**Conclusions**

These initiatives show breadth of D&I possible in the public health arena.

## A50 Developing and evaluating a system for consensus decision-making for prioritizing evidence-based practices for dissemination and implementation in very large systems: A collaboration with the Department of Defense for suicide prevention

### Kate Comtois^1^, Gregory Brown^2^, Andria Pierson^1^, & Sara Landes^3^

#### ^1^University of Washington, Department of Psychiatry and Behavioral Sciences, Seattle, WA, USA; ^2^University of Pennsylvania, Philadelphia, PA, USA; ^3^University of Arkansas for Medical Sciences, Little Rock, AR, USA

##### **Correspondence:** Kate Comtois (comtois@uw.edu)

**Background**

There are multiple evidence-based treatments available to reduce suicide risk for the military. The decision about which of these treatments to implement in massive organizations such as the Department of Defense (DoD) is a daunting task and there is a pressing need to develop a systematic protocol to assist in this effort. Recently, the Military Suicide Research Consortium (MSRC) constituted a Dissemination and Implementation (D&I) Core to promote the dissemination of the practical, evidence-based suicide prevention practices (EBPs) that have resulted from MSRC-funded research.

**Materials and Methods**

This mission is accomplished by: 1. Establishing a D&I Readiness Working Group to foster synergy between MRSC researchers, D&I scientists, Defense Suicide Prevention Office, Defense Center for Excellence, Military Operational Medicine Research Program, and other military settings. 2. Facilitating consensus evaluation of where and how MSRC EBPs will be disseminated or implemented by the DoD and other military settings.

**Results**

To date, the D&I Readiness Working Group has developed and adopted a protocol and template for summarizing and evaluating an intervention’s potential for implementation. Readiness for implementation is evaluated by providing descriptive information organized by a combination of the RE-AIM and Implementation Outcome Frameworks in the following domains: Reach, Evidence for the Clinical Intervention (using a standardized rating of the quality of evidence for suicide and other outcomes), Adoption (i.e., Acceptability, Adoption, Appropriateness), Implementation (Fidelity, Feasibility), Maintenance (i.e., Sustainability, Cost). The D&I Readiness Working Group reviews this information using standardized rating scales and identifies specific military office, agency or setting that has the potential for implementing each intervention.

**Conclusions**

Preliminary evidence on the feasibility and acceptability of this protocol as well as its impact on dissemination of these EBPs within the military will be presented.

## A51 Development of a provider network survey to operationalize and measure a network weaving implementation strategy

### A. Rani Elwy^1,2^, Bo Kim^1,3^, Dorothy Plumb^4,5^, Shihwe Wang^4^, Allen Gifford^1^, Steven Asch^2,4^, Jill Bormann^6^, Brian Mittman^7,8,9^, Thomas Valente^10^, Lawrence Palinkas^11^

#### ^1^Center for Healthcare Organization and Implementation Research, VA Boston Healthcare System, Boston, MA, USA; ^2^Boston University School of Public Health, Department of Health Law, Policy and Management, Boston, MA, USA; ^3^Harvard Medical School, Department of Psychiatry, Boston, MA, USA; ^4^Center for Healthcare Organization and Implementation Research, Edith Nourse Rogers Memorial Veterans Hospital, Bedford, MA, USA; ^5^Boston Medical Center, Department of Family Medicine, Boston, MA, USA; ^6^VA San Diego Healthcare System, San Diego, CA, USA; ^7^VA Greater Los Angeles, Los Angeles, CA, USA; ^8^Kaiser Permanente Southern California, Pasadena, CA, USA; ^9^University of California Los Angeles, Los Angeles, CA, USA; ^10^University of Southern California, School of Medicine, Los Angeles, CA, USA; ^11^University of Southern California, School of Social Work, Los Angeles, CA, USA

##### **Correspondence:** A. Rani Elwy (rani.elwy@va.gov)

**Background**

Promoting network weaving is defined as identifying and building on existing high-quality working relationships within an organization to promote information sharing, collaborative problem-solving, and shared goals related to an implementation [1]. We used a Hybrid 1 mixed methods process evaluation [2] within the context of a Veterans Affairs (VA) RCT to create a network weaving measure.

**Materials and Methods**

We conducted an online social network survey of VA mental health providers eligible to refer to the ongoing RCT, and semi-structured interviews with a subset of these responders. Providers nominated up to 10 colleagues in response to three network survey questions: 1) Which colleagues do you speak to regularly at work? (Q2), 2) Which colleagues’ opinions on new clinical treatments do you rely on the most? (Q3), and 3) Which colleagues do you go to when you need help managing a complex clinical situation at work? (Q4). Each network was analyzed using logistic regression analysis to understand the influence of six centrality measures (indegree, outdegree, incloseness, outcloseness, betweenness, eigenvector) on providers’ self-reported referral behavior [3,4]. We also explored how providers learn about evidence-based practices (EBP) and who they speak to about this. Transcripts were coded using a grounded thematic analysis approach, derived from grounded theory [5] using a constant comparison process. We began with line by line coding, and then individual codes were discussed until consensus was reached. Once all codes were identified and defined, we collapsed codes into overall themes.

**Results**

Web-based surveys were sent to 129 mental health providers, and 69 (53%) were completed. In all three networks, high indegree centrality (number of individuals designating participant) significantly predicted providers’ referral behavior: in Q2 (OR=1.25, 95% CI 1.00, 1.60), Q3 (OR=1.37, 95% CI 1.10, 1.84) and Q4 (OR=1.27, 95% CI 1.03, 1.59). Indegree centrality was highly correlated with both eigenvector centrality and betweenness centrality, indicating that providers who are connected to other highly connected individuals are most likely to serve as bridges between provider subgroups, or cliques. Twenty-five providers (36%) agreed to participate in semi-structured interviews. Twelve interviews were sufficient to reach data saturation. Interviews emphasized beliefs in providers’ own clinical judgments, the idealism of EBPs, and the need to deliberately manufacture time to discuss important clinical issues with colleagues.

**Conclusions**

Opportunities for in-person contact between providers with high degree centrality and those without may promote network weaving, which can be measured by this short, online survey.

**References**

1. Powell BJ, Waltz TJ, Chinman MJ, Damschroder LJ, Smith JL, Matthieu MM, Proctor EK, Kirchner JE. A refined compilation of implementation strategies: results from the Expert Recommendations for Implementing Change (ERIC) project. Implement Sci. 2015;10:21.

2. Curran GM, Bauer M, Mittman B, Pyne JM, Stetler C. Effectiveness-implementation hybrid designs: combining elements of clinical effectiveness and implementation research to enhance public health impact. Med Care. 2012;50(3):217-26.

3. Freeman L. Centrality in social networks: conceptual clarification. Soc Networks 1979;1:215-39.

4. Bonacich P. Technique for analyzing overlapping memberships. Sociol Method. 1972;4:176-85.

5. Charmaz, K. Constructing grounded theory. Sage: Thousand Oaks, CA; 2014.

## A52 Exploring network interventions as a mechanism for measurement based care implementation

### Elena Navarro^1,2^, Cara Lewis^1^,^2^, Alicia Bunger^3^

#### ^1^Department of Psychological and Brain Sciences, Indiana University, Bloomington, IN USA; ^2^Kaiser Permanente Washington Health Research Institute, Seattle, WA, USA; ^3^College of Social Work, Ohio State University, Columbus, OH, USA

##### **Correspondence:** Elena Navarro (elnavarr@indiana.edu)

**Background**

Measurement-based care (MBC) is an evidence-based practice that involves the use of symptom measurement to monitor client progress and inform care [1]. Most community mental health clinicians do not utilize MBC despite its demonstrated effectiveness [2-3]. Social networks might support MBC implementation by diffusing information, social support, and social influence, all of which bear upon clinicians’ attitudes and behaviors [4-6]. There is growing interest in the use of network interventions, such as opinion leaders and implementation teams, as strategies for targeting these key mechanisms to implement new practices. Network interventions target influential individuals that span the network to help identify community needs and barriers to change, develop strategies to facilitate change, and accelerate the adoption of innovations [7]. Few studies have examined how these network interventions influence existing social networks within agencies to appreciate their effect on implementation [8-9]. Two aims guide this study: (1) To determine the differential influence of advice, professional, and personal networks on MBC implementation; and (2) To investigate how implementation teams change existing networks to influence clinician implementation of and fidelity to MBC.

**Materials and Methods**

Within an RCT comparing tailored versus standardized approaches for implementing MBC, clinicians (N=140) across 12 community mental health clinics completed measures assessing demographics, social networks, attitudes about MBC, and use of MBC at baseline and 5 months. From this data, opinion leaders and champions were identified and invited to join implementation teams at the tailored condition sites. Social network analysis was used to determine the influence of each network on MBC implementation and assess how the introduction of implementation teams changed existing networks to promote MBC fidelity. MBC fidelity information was collected via a combination of self-report and objective data from the electronic health record.

**Results**

Preliminary results of 10 sites found that clinicians’ MBC use increased after 5 months of active implementation. However, network structures changed minimally. The influence of the advice, professional, and personal networks on clinicians’ change in MBC use will be further examined using exponential random graph modeling suitable for longitudinal network analysis. Further analyses (using contagion models and ego-networks) will examine how implementation teams rewire existing network structures to influence MBC adoption and how exposure to others using MBC influences clinicians’ adoption of and fidelity to MBC.

**Conclusions**

Determining how network interventions influence social networks and clinicians’ MBC use will clarify implementation mechanisms and inform the use of strategies to increase implementation success.

**References**

1. Scott KS, Lewis CC. Using measurement-based care to enhance any treatment. Cogn Behav Practice. 2015;22(1):49-59.

2. Hatfield DR, Ogles BM. Why some clinicians use outcome measures and others do not. Adm Policy Ment Health. 2007;34(3):283-91.

3. de Jong K, de Goede M: Why do some therapists not deal with outcome monitoring feedback? A feasibility study on the effect of regulatory focus and person-organization fit on attitude and outcome. Psychother Res.2015;25(6):661-8.

4. Fujimoto K, Valente TW, Pentz MA: Network structural influences on the adoption of evidence-based preventions in communities. J Community Psychol. 2009;37(7):830-45.

5. Valente TW: Network interventions. Science. 2012;337(6090):49-53.

6. Palinkas LA, Holloway IW, Rice E, Fuentes D, Wu Q, Chamberlain P: Social networks and implementation of evidence-based practices in public youth-serving systems: A mixed methods study. Implement Sci. 2011;6(113):1-11.

7. Valente TW, Palinkas LA, Czaja S, Chu KH, Brown CH: Social network analysis for program implementation. PLoS ONE. 2015;10(6):1-18.

8. Valente TW, Pumpuang P: Identifying opinion leaders to promote behavior change. Health Educ Behav. 2007;34:881-96.

9. Bunger AC, Hanson RF, Doogan NJ, Powell BJ, Cao Y, Dunn J: Can learning collaboratives support implementation by rewiring professional networks? Adm Policy Ment Health. 2014;43(1):79-92.

## A53 Mixed methods evaluation of the implementation of routine depression screening followed by suicide risk assessment in 3 primary care clinics

### Julie E. Richards^1^, Amy K. Lee^1^, Gwen T. Lapham^1^, Emily C. Williams^2^, Carol E. Achtmeyer^1^, Rebecca L. Parrish^1^, Ryan M. Caldeiro^1^, Evette J. Ludman^1^, Bradley Steinfeld^1^, Katharine A. Bradley^1^

#### ^**1**^Kaiser Permanente Washington Health Research Institute, Seattle, WA, USA; ^2^VA Puget Sound, Seattle, WA, USA

##### **Correspondence:** Julie E. Richards (richards.je@ghc.org)

**Background**

The US Preventive Services Task Force recommends depression, but not suicide, screening in primary care (PC). However, as health systems implement behavioral health integration (BHI), many PC practices are screening for depression with instruments that include suicidality. We describe BHI implementation strategies; and use mixed methods to evaluate screening reach and implementation barriers and facilitators in PC following BHI.

**Materials and Methods**

Between 3/2015 and 3/2016, 3 pilot PC clinics in a large Pacific Northwest health system implemented BHI. Standard BHI work for Medical Assistants (MAs) included routine screening using the 2 item Patient Health Questionnaire [PHQ-2]. Patients with positive screens (≥2 on either PHQ-2 item) were asked to complete the remainder of the PHQ-9 depression screen. Those with frequent suicidal ideation (PHQ-9 question #9 score 2-3) were asked to complete the Columbia-Suicide Severity Rating Scale [C-SSRS].

Implementation strategies included: partnership between clinical leaders and researchers, local clinical champions, 1-day workshop for frontline staff to design workflow, rapid testing and revision, automated EHR prompts, staff training, frequent performance feedback, ongoing support from practice coaches for 6 months, and regular PDCA meetings to address quality gaps.

Quantitative analyses describe the proportion of patients screened during standard BHI work. Qualitative analyses identified barriers and facilitators to implementation using notes for weekly formative evaluation meetings with practice coaches.

**Results**

Among adult PC patients visiting the clinics during implementation, 74% completed the PHQ-2 (22,081 of 29,857). Among positive screens (n 3,597), 82% (n 2,553) were assessed for depression, including suicidal ideation, with the PHQ-9. Of those, 67% (n 1,700) had moderate to severe current depressive symptoms (PHQ-9≥10) and 11% (n 275) had frequent suicidal ideation. Of those with ideation, 228 (83%) of those were assessed for suicide risk by C-SSRS (mean 2.3, SD 1.6, range 0-6).

Key facilitators included: ownership of the screening process by MAs, perceived value of the assessments by clinicians, PC social workers trained to support BHI, and positive stories of identifying suicidal patients who were being seen for unrelated issues (e.g. wart removal). A key barrier was lack of EHR tools to prompt use of the C-SSRS for patients with suicidal ideation.

**Conclusions**

After BHI implementation, a large proportion of patients were screened for depression followed by severity assessment, and assessed for suicide risk after report of ideation. Formative evaluation results, including positive stories, are being used to refine and spread the implementation strategy in the health care system.

## A54 Automated reporting on therapist’s fidelity to motivational interviewing: Technology overview and pilot feasibility results

### David Atkins (datkins@uw.edu)

#### University of Washington, Seattle, WA, USA

**Background**

Monitoring fidelity – or the quality with which a treatment is being provided – is an ongoing challenge in the implementation of behavioral interventions such as psychotherapy [1]. The research-based methodology of using human evaluators is not practical in real-world settings, and thus, psychotherapy training and ongoing clinical services happen without any objective feedback on treatment quality. Technology advancements in processing and modeling spoken language (e.g., automated speech recognition, natural language processing) provide the necessary tools for a computational solution to automated fidelity ratings with behavioral treatments. The current presentation will introduce technology – the counselor observer ratings expert for MI (CORE-MI) – to provide automated feedback on therapist fidelity to motivational interviewing, and will report results from a pilot study in which the system provided automated feedback to MI therapists based on standardized patient sessions.

**Materials and Methods**

The CORE-MI system was developed and tested using 1,825 MI sessions, wherein 356 sessions had detailed fidelity coding to train algorithms to identify specific MI fidelity codes [2,3]. Algorithms used the words spoken and paralinguistic features (e.g., vocally-encoded arousal) to predict fidelity codes. Incorporating user-centered design methodology, a web-based interactive reporting tool allows therapists to review their MI fidelity ratings relative to clinical standards and review the session transcript, as well as vocally-encoded arousal of therapist and patient throughout the session [4]. To pilot test the system, 10 experienced and 10 novice MI clinicians participated in 10 minute sessions with a standardized patient. In a follow-up meeting, they were provided with an automated report of their session and were interviewed about their experiences and perceived accuracy of the report.

**Results**

The CORE-MI tool demonstrated basic feasibility, in which the computational processing steps worked without error on all sessions. The majority of therapists: 1) found the automated feedback “representative of my clinical performance in the recorded session” (85%; 17/20), 2) were highly satisfied with the report format and content (100%; 19/19), and 3) “would use the [tool] in my clinical practice” (89%; 17/19). Preliminary analyses of computer- generated fidelity codes suggest that the system can discriminate novice from expert MI clinicians.

**Conclusions**

Spoken language technologies provide methodologies to enable automated feedback on behavioral interventions, removing a significant barrier to the successful implementation of such treatments. A current study implements the CORE-MI system within an active training clinic to evaluate its impact on therapist performance and patient outcomes.

**References**

1. Proctor E, Silmere H, Raghavan R, Hovmand P, Aarons G, Bunger A, Griffey R, Hensley M. Outcomes for implementation research: conceptual distinctions, measurement challenges, and research agenda. Adm Policy Ment Health. 2011;38(2):65-76. doi:10.1007/ s10488-010-0319-7.

2. Xiao B, Huang C, Imel ZE, Atkins DC, Georgiou P, Narayanan SS. A technology prototype system for rating therapist empathy from audio recordings in addiction counseling. Peer J Comput Sci. 2016;2:e59 doi:10.7717/ peerj-cs.59

3. Can D, Marín RA, Georgiou PG, Imel ZE, Atkins DC, Narayanan SS. “It sounds like...”: A natural language processing approach to detecting counselor reflections in motivational interviewing. J Couns Psychol. 2016;63(3):343-50. doi:10.1037/cou0000111

4. Gibson J, Gray G, Hirsch T, Imel Z, Narayanan S, Atkins D. Developing an automated report card for addiction counseling: the Counselor Observer Ratings Expert for MI (CORE-MI).http://mentalhealth.media.mit.edu/wpcontent/uploads/sites/46/2016/04/COREMI_ positionpaper_cameraready.pdf. Accessed March 15, 2017.

## A55 Computer-facilitated 5A’s for tobacco addiction: Using technology to promote implementation and fidelity

### Jason Satterfield^1^, Steve Gregorich^1^, Nicholas Alvarado^1^, Ricardo Munoz^2^, Maya Vijayaraghavan^1^

#### ^1^Department of Medicine, University of California San Francisco, San Francisco, CA, USA; ^2^Department of Psychiatry, University of California San Francisco, San Francisco, CA, USA

##### **Correspondence:** Jason Satterfield (Jason.Satterfield@ucsf.edu)

**Background**

Clinical practice guidelines recommend that primary care providers (PCPs) deliver the 5A’s (ask, advise, assess, assist, and arrange) at every clinical encounter for the treatment of tobacco use disorders [1]. Unfortunately, while most clinicians “ask” and “advise,” adherence to the more powerful “assist” and “arrange” steps remains low [2]. Innovative service delivery models are needed to improve 5A’s fidelity and adherence.

**Materials and Methods**

PCPs from 3 diverse, adult PC clinics were randomized into the CF5A’s condition or to usual care. Patients who smoke were recruited in PC waiting rooms and assigned to the condition of their provider. Intervention patients completed the 5A’s computer intervention and two tailored clinical summaries were generated – one for the PCP and one for the patient. Control patients completed an eligibility survey and consent document only. Within 72 hours of the PC appointment, patients completed a post-visit, telephone survey about their receipt of the 5A’s during the PC encounter. Patients could participate up to 3 times within the one year study period [3].

**Results**

272 PCPs were randomized (n=137 intervention; n=135 usual care) and saw n=961 patients for a total of n=1,340 visits. N=1,011 post-visit surveys were completed (75.4% response rate). Using logistic regression and GEE models to control for clustering, significant main effects were found for the intervention group on Ask, Advise, Assess, and Assist. (Arrange was not included.) Intervention patients were also more likely to receive all 5A’s compared to controls but only for their first participating visit. Adjusted odds ratios ranged from 1.57 (Ask) to 3.43 (Assist). Main effects were also found for clinic site with an HIV clinic having lower odds of delivering the 5A’s compared to general primary care.

**Conclusions**

A computer-facilitated 5A’s delivery model was effective in improving the fidelity of the 5A’s received by adult PC patients. Effectiveness was attenuated by clinic site and affected by the number of clinic visits to the same provider with earlier visits showing stronger results. While this relatively low cost, time saving intervention has great potential for this and other service delivery, future studies should help identify ways to promote and sustain technology implementation and integration with clinic flow [4].

**References**

1. Fiore MC, Jaén CR, Baker TB, Bailey WC, Benowitz NL, Curry SJ, et al. Treating tobacco use and dependence: 2008 update U.S. Public Health Service Clinical Practice Guideline executive summary. Respir Care. 2008;53(9):1217-22.

2. Park ER, Gareen IF, Japuntich S, Lennes I, Hyland K, DeMello S, Sicks JD, Rigotti NA. Primary care provider-delivered smoking cessation interventions and smoking cessation among participants in the National Lung Screening Trial. JAMA Intern Med. 2015;175(9):1509-16. doi:10.1001/ jamainternmed.2015.2391

3. Kalkhoran S, Appelle NA, Napoles AM, Munoz RF, Lum PJ, Alvarado N, Gregorich SE, Satterfield JM. Beyond the ask and advise: implementation of a computer tablet intervention to enhance provider adherence to the 5As for smoking cessation. J Subst Abuse Treat. 2016;60:91-100. doi. 10.1016/j.jsat.2015.05.009

4. Holden, R., Karsh, B. The Technology Acceptance Model: Its past and its future in health care. J Biomed Inform. 43(1):159-72. doi:10.1016/j.jbi.2009.07.002

## A56 Implementation pilot study results: Social determinants of health data tools in community health centers

### Rachel Gold^1,2^, Erika Cottrell^2,3^, Arwen Bunce^2^, Celine Hollombe^2^, Katie Dambrun^1^, Mary Middendorf^1^, Edward Mossman^2^, Stuart Cowburn^2^, Maria Zambrano^6^, Gerardo Melgar^5^, Peter Mahr^4^

#### ^1^OCHIN, Inc., Portland, OR, USA; ^2^Kaiser Permanente NW Center for Health Research, Portland, OR, USA; ^3^Oregon Health & Science University, Portland, OR, USA; ^4^Multnomah County Health Department, Portland, OR, USA; ^5^Cowlitz Family Health Center, Longview, WA, USA; ^6^La Clinica del Valle Family Health Care Center, Medford, OR, USA

##### **Correspondence:** Rachel Gold (rachel.gold@kpchr.org)

**Background**

Addressing patients’ SDH may be as important to their health as addressing their medical needs. Implementing the systematic collection of SDH data in electronic health records (EHRs) could augment care in ‘safety net’ CHCs, whose socioeconomically vulnerable patients have worse health outcomes than the general population. Little is known about how to implement standardized SDH data collection, using EHR-based SDH data tools, in standard CHC workflows.

**Materials and Methods**

We conducted a ten-month, CHC stakeholder-driven tool design process. Per stakeholder input on SDH screening tool content and format, our ‘SDH data tools’ harnessed commonly-used EHR functions (e.g., data entry flowsheets, order preference lists). After iteratively revising the tools, we activated them in three pilot CHCs in June 2016. Rates of tool adoption (SDH data collection, and SDH-related referrals) in the year post-tool implementation were tracked using EHR data. Qualitative data were collected via on-site observation and interviews with care team members involved in adopting the SDH data tools (e.g., RN care managers, MAs, referral coordinators, Community Health Workers), email communication with pilot clinic stakeholders (e.g., clinic managers, lead provider, medical director, quality manager), and attendance at webinars / group discussions where the SDH tools were discussed.

**Results**

The SDH data tools were designed to enable collection and summarization of patient-reported SDH data, and to help care teams follow up on identified SDH needs. Adoption approaches varied, as the pilot clinics targeted different populations for SDH screening. Clinic A: Every new patient seen by a single provider. Clinic B: Patients with Hepatitis C or high-risk diabetes and those aged >=65. Clinic C: New patients / patients at annual sliding scale fee authorization renewal. As of February 23, 2017, 732 distinct patients were screened for SDH needs; 72% of screened patients reported financial resource strain, 31%-38% reported housing insecurity, 55%-59% reported food insecurity, 20%-29% reported exposure to violence, 42%-53% reported lack of adequate physical activity, 62%-75% reported social connections/isolation, and 59%-73% reported stress. Of the patients screened, 234 (32%) received a referral associated with SDH. Results from the full year of follow-up will be presented at the conference.

**Conclusion**

Implementing SDH data collection in busy, resource-constrained CHCs is feasible. It involved optimizing the data tools, taking adaptive approaches to targeted populations, and minimizing the workflow changes and burdens involved in tool adoption.

## A57 Successes and challenges in delivering consultation in evidence-based psychotherapies

### David Riggs (driggs@deploymentpsych.org)

#### Uniformed Services University of the Health Sciences, Center for Deployment Psychology, Bethesda, MD, USA

**Background**

There has been an increased emphasis on providing evidence-based psychotherapies (EBPs) in military and Veteran Affairs (VA) healthcare environments to treat PTSD and other conditions. Research shows that successful implementation of EBPs with fidelity involves not only training, but also post-workshop consultation [1,2]. The Center for Deployment Psychology (CDP) supports training and implementation of EBPs within the DoD and has been successful in disseminating EBP workshops via in-person and online formats. Despite these successful dissemination efforts, CDP has struggled to gain participation in post-workshop consultation for these EBPs.

**Materials and Methods**

This talk will outline many of the efforts CDP has undertaken in the past several years to address this gap in implementation. Multiple models and strategies for increasing participation in consultation have been pursued, including making consultation available at no cost over the phone and via online platforms, attempting to replicate the VA model by soliciting supervisor buy-in so that providers will be able to access consultation, piloting a program offering certification in EBP proficiency, pairing a workshop with follow-on refresher consultation sessions, and finally placing full-time staff at selected military sites to provide on-site consultation. CDP conducts periodic surveys as part of its program evaluation efforts, which ask about reasons for low levels of consultation usage.

**Results**

CDPs workshops receive very positive ratings in satisfaction and participants routinely show strong knowledge gains in post-training assessments. Participants also strongly endorse the need for consultation while at these training events. Unfortunately, the models that have been attempted have only partially met the need for bridging the gap, with the majority of participants not receiving consultation. Based on the results from multiple programs and projects, CDP has documented many of the challenges inherent in getting providers to access consultation and later use these EBPs. These barriers to implementation include systemic and individual factors and are outlined in a Lessons Learned Manual that CDP has created.

**Conclusions**

CDP continues to explore innovative methods to implement and disseminate EBP training and encourage participants to engage in post-workshop consultation. Noting that a key reason for lack of consultation lies in systemic barriers within the Military Health System, CDP has sought to address these challenges by creating a clinic optimization toolkit. This toolkit contains products tailor made to mitigate or eliminate barriers to receiving consultation and ultimately aims to improve utilization of EBPs throughout the DoD.

**References**

1**.** Department of Veterans Affairs. Local implementation of evidence-based psychotherapies for mental and behavioral health conditions. VHA Handbook. 2012 http://www.va.gov/vhapublications/ ViewPublication.asp?pub_ID=2801

2. Foa EB, Gillihan SB, Bryant RA. Challenges and successes in dissemination of evidence-based treatments for posttraumatic stress: lessons learned from Prolonged Exposure Therapy for PTSD. Psychol Sci Public Interest 2013;14(2):65-111.

## A58 A comparison of two learning collaborative strategies to support newly trained clinicians in delivering cognitive processing therapy

### Shannon Wiltsey-Stirman^1^, Matthew Beristianos^1^, Norman Shields^2^, Kera Mallard^1^, Tasoula Masina^2^, Rachel Haine- Schalgel^3^, Christopher Miller^4^, Michael Suvak^5^, Clara Johnson^1^, Patricia K. Carreño^1^, Candice Monson^6^

#### ^1^National Center for PTSD and Stanford University, Menlo Park, CA, USA; ^2^Divisional Psychologist Occupational Health and Safety, Royal Canadian Mounted Police, Ottawa, Ontario, Canada; ^3^Ryerson University, Toronto, Ontario, Canada; ^4^San Diego State University, San Diego, CA, USA; ^5^VA Boston Center for Healthcare, Organization, and Implementation Research, Boston, MA, USA; ^6^Suffolk University, Boston, MA, USA

##### **Correspondence:** Shannon Wiltsey-Stirman (sws1@stanford.edu)

**Background**

Numerous research studies have demonstrated that short-term cognitive-behavioral psychotherapies, such as Cognitive Processing Therapy (CPT), lead to substantial and sustained improvements in PTSD symptoms [1-3]. There has been little research to identify the most effective strategies for training providers, or for providing long- term support to facilitate ongoing, high quality use of evidence-based psychotherapies (EBPs) in routine care settings. Whether the focus of implementation efforts should be fidelity to EBPs or adaptation of either the EBP or the setting to facilitate EBP use has not been determined [4,5].

**Materials and Methods**

In this study, clinicians (n=40) who attended a workshop were randomized into either a twelve-month Continuous Quality Improvement-oriented Learning Collaborative (CQI) or a Fidelity-oriented Learning Collaborative (FID) to learn to deliver CPT. Patient (n=66) symptoms were assessed via weekly self-reported PTSD inventories and periodic assessment of other symptoms and functioning. Clinicians uploaded recordings of every CPT session and completed reports of their CPT use and adaptation every month. At the end of the twelve-month learning collaborative phase, the two conditions were compared using the longitudinal data on engagement and dropout at the clinician and client level, clinical outcomes, clinician fidelity, and adaptation of CPT.

**Results**

Preliminary data suggests that patients whose therapists participated in the CQI condition may have experienced greater symptom change, although both groups improved. CQI therapists reported more adaptations to CPT that were fidelity-consistent, but groups did not differ on self-reported fidelity-inconsistent adaptations. Analyses with all data from the twelve-month consultation phase will be presented, and patterns of observer-rated fidelity and adaptation in the two conditions will also be examined.

**Conclusions**

These results suggest that continuous quality improvement activities may result in improved patient outcomes. Potential mechanisms will be explored and discussed.

**References**

1. Resick PA, Nishith P, Weaver TL, Astin MC, Feuer CA. A comparison of cognitive- processing therapy with pro- longed exposure and a waiting condition for the treatment of chronic posttraumatic stress disorder in female rape victims. J Consult Clin Psychol. 2002;70(4):867-79.

2. Resick PA, Galovski TE, Uhlmansiek MOB, Scher CD, Clum GA, Young-Xu Y. A randomized clinical trial to dis- mantle components of cognitive processing therapy for posttraumatic stress disorder in female victims of inter- personal violence. J Consult Clin Psychol. 2008;76(2):243-58.

3. Resick PA, Williams LF, Suvak M, Monson CM, Gradus JL. Long-term outcomes of cognitive–behavioral treatments for posttraumatic stress disorder among female rape survivors. J Consult Clin Psychol. 2012; 80(2):201-10.

4. Chambers DA, Glasgow RE, Stange KC. The dynamic sustainability framework: addressing the paradox of sustainment amid ongoing change. Implement Sci. 2013;8(1):117.

5. Stirman SW, Kimberly J, Cook N, Calloway A, Castro F, Charns M. The sustainability of new programs and innovations: a review of the empirical literature and recommendations for future research. Implement Sci. 2012; *7*(1):17.

## A59 Creating system change: A state-initiated rollout of the R3 supervisor-targeted practice change model

### Lisa Saldana^1^, Patricia Chamberlain^1^, Jason Chapman^1^

#### ^**1**^Oregon Social Learning Center, Eugene, OR, USA

##### **Correspondence:** Lisa Saldana (lisas@oslc.org)

**Background**

Of the many empirically supported behavioral health interventions shown to improve outcomes for high-risk children and families, few are assimilated into public service systems enough to have a measurable public health impact.

R^3^ came by request of a large child welfare system (CWS) to train their workforce in the use of evidence-based principles in each interaction with families. R^3^ draws from three areas of reinforcement shown to produce positive outcomes: Reinforcement of (1) effort, (2) relationships and roles, and (3) small steps toward goal achievement. The aim is to bolster positive progress toward completing CWS treatment plans and to improve system-level outcomes related to permanency and stability. A supervisor targeted implementation strategy maximizes the potential reach across the system, while working under the real-world limitations of training and coaching capacity.

**Materials and Method**

An ongoing state-initiated rollout of R^3^ provides a real-world research opportunity. Observation-based, rapid, fidelity monitoring and feedback facilitates the potential for efficient system-wide behavior change.

Supervisors meet monthly with their caseworkers for group supervision, uploading a video of each to a secure fidelity monitoring web-based system for review by an R^3^ expert coach. Coaches provide monthly observational monitoring and fidelity rating, written feedback, and virtual consultation coaching. Supervisors are coached to use R^3^ in their interactions with caseworkers and to support the caseworkers to use R^3^ with their families.

Following a Cascading Full Transfer model [1], supervisors are encouraged toward certification. Necessary milestones include participation in a minimum of: 12 months of coaching, with 80% session upload for fidelity rating and attendance; 80% attendance of caseworkers for group supervision each month; and 3 consecutive months of acceptable fidelity ratings.

**Results**

Four cohorts of CWS staff including regional leadership, supervisors, and caseworkers were trained in R^3^ between September 2015 and February 2016 (n = 355). Over the first year, all 4 regions developed some supervisors who achieved certification, with numbers continuing to grow consistently over time (current range: 23-60% of supervisors). Outcomes will include change over time in fidelity ratings from baseline to current performance. Preliminary outcomes will be provided linking fidelity to system level outcomes such as permanency.

**Conclusions**

R^3^ was developed to improve interactions between families and the CWS. Outcomes suggest the potential to successfully train and sustain R^3^ in a real-world CWS. Infusing evidence-based strategies into the CWS, raises the potential for quality and timely service plans to be achieved ultimately leading to improved individual and system level outcomes.

**Reference**

1. Chamberlain P, Feldman SW, Wulczyn, F, Saldana L, Forgatch, M. Implementation and evaluation of linked parenting models in a large urban child welfare system. Child Abuse Negl. 2016;53*:*27-39.

## A60 Interactions between organizational and clinician constructs as predictors of therapist use of evidence-based and non-evidence-based practices

### Emily M. Becker-Haimes^1^, Nathaniel Williams^2^, Zuleyha Cidav^1^, Ronnie Rubin^3^, & Rinad S. Beidas^1^

#### ^1^Center for Mental Health Policy and Services Research, University of Pennsylvania, Philadelphia, PA, USA; ^2^School of Social Work, Boise State University, Boise, ID, USA; ^3^Department of Behavioral Health and Intellectual disAbility Services, Philadelphia, PA, USA

##### **Correspondence:** Emily M. Becker-Haimes (embecker@upenn.edu)

**Background**

Theoretical implementation models posit factors impacting evidence-based practice (EBP) use at multiple levels (e.g., clinician, organizational) [1-3] and both clinician and organizational predictors of EBP use have been identified [4-6]. However, theoretical models provide little guidance for hypothesizing interactive effects across these multilevel contexts. Empirical work examining interactions between organizational and clinician constructs to predict use of EBP and non-EBP techniques in the context of EBP implementation may help supplement existing theory.

**Materials and Methods**

We examined interactions between clinician and organizational characteristics as predictors of clinician use of cognitive-behavioral (CBT) and psychodynamic therapy techniques in a large sample of community mental health clinicians (N=247, M age = 38.74, SD = 11.9, 77.7% female). Clinician characteristics included attitudes toward EBP [7], years of clinical experience, theoretical orientation, and participation in an EBP implementation initiative. Organizational constructs included general organizational proficiency [8] and multiple dimensions of more specific measurement of implementation climate [9]. A series of mixed-effects interaction models examined whether the relationship between clinician variables and CBT use was moderated by organizational variables; models also examined predictors of psychodynamic use. Main effects were interpreted in the absence of an interactive effect.

**Results**

With respect to predictors of CBT use, there was a significant interaction between clinician attitudes about the appeal of EBP and organizational proficiency (p < .01); higher ratings of EBPs as appealing predicted greater CBT use in the context of high proficiency cultures (b = .16, p =.07, but not within low proficiency cultures (b = -.11, p =.14). Greater openness to EBPs and more years of experience were significant main effects of CBT use. When predicting use of psychodynamic techniques, there was a significant interaction between years of experience and the implementation climate reward subscale. More years of experience was associated with greater use of psychodynamic techniques in low reward for EBP climates (b = .02, p = .008) but was not related to psychodynamic technique use in high reward for EBP climates (b = .005, p = .45).

**Conclusions**

Results suggest that examining interactions between organizational and clinician variables in the context of implementation can provide more nuanced insight into predictors of both EBP and non-EBP use. Findings suggest that it may be possible to optimize the likelihood of implementation success by leveraging synergistic relationships between clinician and organizational variables. Implications for theoretical implementation models will be discussed.

**References**

1. Damschroder LJ, Aron DC, Keith RE, Kirsh SR, Alexander JA, Lowery JC. Fostering implementation of health services research findings into practice: A consolidated framework for advancing implementation science. Implement Sci. 2009;4(1):1-15

2. Raghavan R, Bright CL, Shadoin AL Toward a policy ecology of implementation of evidence-based practices in public mental health settings. Implement Sci. 2008;3(1):1-26.

3. Wandersman A, Duffy J, Flaspohler P, et al. Bridging the gap between prevention research and practice: the interactive systems framework for dissemination and implementation. Am J Community Psychol. 2008;41(3-4):171-81.

4. Aarons GA, Sommerfeld DH, Walrath-Greene CM. Evidence-based practice implementation: the impact of public versus private sector organization type on organizational support, provider attitudes, and adoption of evidence-based practice. Implement Sci. 2009;4(1):1.

5. Brookman-Frazee L, Haine RA, Baker-Ericzén M, Zoffness R, Garland AF. Factors associated with use of evidence-based practice strategies in usual care youth psychotherapy. Adm Policy Ment Health. 2010; 37(3):254 69.

6. Beidas RS, Marcus S, Aarons GA, Hoagwood KE, Schoenwald S, Evans AC, et al. Individual and organizational factors related to community clinicians’ use of therapy techniques in a large public mental health system. JAMA Pediatr. 2015;169(4):374-82.

7. Aarons GA. Mental health provider attitudes toward adoption of evidence-based practice: The Evidence- Based Practice Attitude Scale (EBPAS). Ment Health Serv Res. 2004;6(2):61-74.

8. Glisson C, Landsverk J, Schoenwald S, Kelleher K, Hoagwood KE, Mayberg S, et al. Assessing the organizational social context (OSC) of mental health services: Implications for research and practice. Adm Policy Ment Health. 2008;35:98-113.

9. Ehrhart, MG, Aarons GA, Farahnak LR. Assessing the organizational context for EBP implementation: the development and validity testing of the Implementation Climate Scale (ICS). Implement Sci. 2014;9(1):1157.

## A61 Unintended consequences of evidence-based treatment policy reform

### Alayna L. Park^1^, Katherine H. Tsai^2^, Karen Guan^1^, Richard Border^3^, & Bruce F. Chorpita^1^

#### ^1^University of California, Los Angeles, Los Angeles, CA, USA; ^2^Five Acres – The Boys’ and Girls’ Aid Society of Los Angeles, Los Angeles, CA, USA; ^3^University of Colorado Boulder, Boulder, CO, USA

##### **Correspondence:** Alayna L. Park (alaynapark@ucla.edu)

**Background**

In 2009, the Los Angeles County Department of Mental Health (LACDMH) Prevention and Early Intervention (PEI) Plan was approved, which offered fiscal incentives for the delivery of 32 evidence-based treatments (EBTs) and free trainings in 6 EBTs. Although the LACDMH PEI Plan represents an impressive accomplishment in terms of promoting the widespread adoption of EBTs, it remains unclear whether such efforts are achieving the desired public health impact—particularly considering that most EBTs have not been developed for or tested with the diverse youth who are often seen in the community [1].

**Materials and Methods**

We examined (a) the applicability of the EBTs offered by the PEI Plan to youth accessing LACDMH services, and (b) the delivery of EBTs in the context of the LACDMH PEI Plan initiative. Data were gathered from 60 youth, aged 5-15, receiving treatment for anxiety, depressive, traumatic stress, or disruptive behavior concerns under the PEI Plan and their 21 providers [2]. Providers were trained in an average of 2.55 EBTs. Information about the interventions [3] that youth were receiving was coded as EBTs, off-label EBTs (i.e., EBTs designed to treat a different presenting problem or age group), EBT practices (i.e., practices commonly featured in EBTs that were not specific to a particular EBT protocol), and unstructured treatment (i.e., practices that were not informed by the EBT literature).

**Results**

Sixty-three percent of youth in our sample matched the presenting problem and age that at least one EBT from the PEI Plan was designed to treat; 54% of youth could be covered by at least one EBT in which the PEI Plan offered free training; 40% of youth could be covered by at least one EBT in which their provider was trained. Of the 60 sampled youth, 17 received an EBT as their primary intervention (10 of these youth received an EBT used off- label), 29 youth received EBT practices, and 14 youth received unstructured treatment.

**Conclusions**

Results suggest that policies promoting dissemination of EBTs do not necessarily equate to EBT use and that the expectation for EBTs to be applied as a unified treatment package to every client may be unrealistic given the limitations of the evidence base. These findings emphasize the need to contemplate the promotion of effective psychotherapy options other than EBTs (e.g., modular approaches to therapy), and highlight the importance of considering the service sample and provider workforce when making policy decisions about mental health treatments.

**References**

1. Southam-Gerow, MA, Chorpita, BF, Miller, LM, Gleacher, AA. Are children with anxiety disorders privately referred to a university clinic like those referred from the public mental health system? Adm Policy Ment Health. 2008;35(3):168-80.

2. Chorpita BF, Daleiden EL, Park AL, Ward AM, Levy MC, Cromley T, et al. Child STEPs in California: a cluster randomized effectiveness trial comparing modular treatment with community implemented treatment for youth with anxiety, depression, conduct problems, or traumatic stress. J Consult Clin Psychol. 2016;85(1):13-25.

3. Ward, AM, Regan, J, Chorpita, Starace N, Rodriguez A, Okamura K, et al. Tracking evidence based practice with youth: Validity of the MATCH and Standard Manual Consultation Records. J Clin Child Adolesc Psychol. 2013;42(1):44-55.

## A62 Coordinated knowledge systems: Enhancing the use of evidence in clinical decision making

### Kimberly D. Becker^1^, Alayna L. Park^2^, & Bruce F. Chorpita^2^

#### ^1^University of Maryland, Baltimore, MD, USA; ^2^University of California, Los Angeles CA, USA

##### **Correspondence:** Kimberly D. Becker (beckerkd@mailbox.sc.edu)

**Background**

Service organizations frequently fail to make optimal use of evidence, resulting in inefficiencies in mental health care and reduced quality of life for children and families. Consistent with the Knowledge to Action Process [1] that emphasizes action-oriented evidence, we examined whether a Coordinated Knowledge System (CKS) that organizes and coordinates the relevant research evidence, the individuals who use that evidence, and the workflow and decisions in which those individuals operate could produce greater use of evidence relative to a traditional resource (i.e., practice guidelines) that separates evidence delivery from the planning and action that follow.

**Materials and Methods**

We evaluated the effect of a CKS on use of evidence in the context of engaging students in school mental health services within the Los Angeles Unified School District (LAUSD), a site that sought our assistance due to a trend of poor engagement in these services.

Participants were supervisors (n = 4) and school mental health clinicians (n = 16). Each supervisor, along with their four supervisees, was randomly assigned to either the CKS or the Traditional Resource (TR) condition. Individuals in the CKS condition received training in how to use four tools as part of a coordinated action sequence: (1) a screener to detect low youth/caregiver engagement, (2) a worksheet to structure collaborative reflection about engagement and predispose the use of evidence in decision making and clinical practice, (3) written guides that describe how to do different engagement procedures, and (4) a measurement feedback tool.

Individuals assigned to the TR condition received training in a traditional evidence resource (i.e., written practice guidelines for addressing poor engagement). Supervisors and supervisees used their respective materials with two cases that demonstrated risk for engagement problems. One supervision session was recorded for each case. A detailed coding system was applied to each session to examine the use of evidence.

**Results**

During supervision, the CKS group spent more turns discussing the nature of the engagement problem as well as making plans to improve engagement. Discussion was more thorough in the CKS group relative to the TR group. CKS participants reported that the materials were easy to use, provided structure to their supervision sessions, provided them with new ideas, and supported their clinical decision making.

**Conclusions**

This research provides a model for improving the translation of knowledge to mental health care and offers insights into how Coordinated Knowledge Systems can fit into existing service system infrastructure.

**Reference**

1. Graham ID, Logan J, Harrison MB, Straus SE, Tetroe J, Caswell W, Robinson N. Lost in translation: time for a map? J Contin Educ Health Prof. 2006;26:13-24.

## A63 What comprises ‘organizational context’ in implementation research? A systematic integrative review

### Shelly-Anne Li^1^, Melanie Barwick^2^, Lianne Jeffs^1,3^, Bonnie Stevens^1,2^

#### ^1^Faculty of Nursing, University of Toronto, Toronto, Ontario, Canada; ^2^Child and Youth Mental Health Research Unit, The Hospital for Sick Children, Toronto, Ontario, Canada; ^3^Li Ka Shing Knowledge Institute, St. Michael’s Hospital, Toronto, Ontario, Canada

##### **Correspondence:** Shelly-Anne Li (shellyanne.li@mail.utoronto.ca)

**Background**

Although organizational context is recognized as a key consideration for implementing evidence-based practices (EBPs) in healthcare services, there is a lack of conceptual clarity on this construct. Definitional inconsistencies among implementation researchers may impede the identification of important organizational contextual factors (i.e., leadership, culture, resources) that facilitate or hinder EBP implementation, resulting in suboptimal implementation outcomes within healthcare organizations. This integrative review summarizes the empirical literature on the influence of organizational context factors on implementing research evidence in healthcare settings.

**Materials and Methods**

We identified published literature that described, explained, measured, or explored organizational context during the implementation process for EBPs. Systematic searches for peer-reviewed empirical studies were performed in Cochrane databases, CINAHL, MEDLINE, EMBASE, and PsycINFO. Two reviewers independently and concurrently screened the titles and abstracts for study inclusion. Quality appraisal of the studies was performed using the Mixed Methods Appraisal Tool.

**Results**

The search yielded 692 citations. Following a review of titles and abstracts, 50 relevant articles were identified, retrieved in full-text and reviewed for eligibility. Twelve peer-reviewed journal articles were included. Half (n=6) of the included studies were guided by an implementation framework. Authors of included studies identified over 20 different factors as related to organizational context. Among these 20, only four organizational contextual factors were consistently measured/explored (identified in ≥50% of included studies) including: resources, leadership, communication and networks, and culture. These factors map on to the constructs of the Inner Setting domain of the Consolidated Framework for Implementation Research (CFIR).

**Conclusions**

The authors of the included studies reported on multiple factors, suggesting a lack of consensus for the operational definition of organizational context. It is noted that constructs related to the CFIR’s Inner Setting domain were common. These results provide initial indication that organizational context is an important consideration in implementation of evidence in healthcare settings but further work is needed to refine its definition.

## A64 Bringing evidence-based interventions into the schools: The impact of organizational factors on implementation success

### Hannah Frank^1^, Lisa Saldana^2^, Philip Kendall^1^, Holle Schaper^2^

#### ^**1**^Temple University, Philadelphia, PA, USA; ^2^Oregon Social Learning Center, Eugene, OR, USA

##### **Correspondence:** Hannah Frank (Hannah.frank@temple.edu)

**Background**

Children with mental health problems often do not receive mental health services, and when they do, it is most often through their schools [1]. Many barriers exist to the successful implementation of evidence-based practices (EBPs) in schools, including the need for organizational support in the implementation process [2]. The present study aims to examine organizational factors that relate to implementation outcomes for a computer-assisted cognitive behavioral therapy intervention (Camp Cope-A-Lot) designed to treat anxious youth.

**Materials and methods**

The present study includes 20 elementary schools from the United States (n=7) and Canada (n=13). These schools were involved in a dissemination and implementation study that examined the sustainability of Camp Cope-A-Lot in schools as delivered by school providers. Teachers from each school (N=86) completed the Organizational Social Context measure (OSC [3]) prior to program implementation. Study staff completed the Stages of Implementation Completion (SIC [4]), a measure that assesses the duration and proportion of activities completed across three phases of implementation (pre-implementation, implementation, and sustainability), for each school.

**Results**

Comparisons between United States (domestic) and Canadian (international) sites indicated that OSC proficiency (i.e., competency in the intervention and responsiveness to the needs of students) was significantly higher for domestic sites, t(18)=2.74, p=.01. The duration of pre-implementation activities was also significantly longer for domestic than for international sites, t(18) = 5.12, p < .001. There were no significant differences between domestic and international sites on pre-implementation proportion (all sites completed all activities), implementation duration, or implementation proportion. A hierarchical linear regression predicting pre-implementation SIC duration indicated that site location (domestic versus international) was a significant predictor, and OSC proficiency trended toward significance (p =.07). Pre-implementation duration was the only significant predictor of implementation duration, such that a longer duration during pre-implementation predicted a shorter duration in the implementation phase. There were no significant predictors of proportion scores.

**Conclusions**

These findings suggest that there is a relationship between proficiency, site location, and speed of implementation. Specifically, domestic schools took longer to complete pre-implementation activities and had higher proficiency scores. Schools that spent longer in the pre-implementation phase spent less time in the implementation phase. These results suggest that spending sufficient time preparing for implementation and establishing proficiency may allow schools to proceed more quickly through the implementation phase. Consistent with previous research, organizational factors appear to play an important role in the implementation of EBPs in schools.

**References**

1. Stephan SH, Weist M, Kataoka S, Adelsheim S, Mills C. Transformation of children’s mental health services: the role of school mental health. Psychiatr Serv. 2007;58:1330-8.

2. Gottfredson DC, Gottfredson GD. Quality of school-based prevention programs: results from a National Survey. J Res Crime Delinq. 2002;39:3-35.

3. Glisson C, Landsverk J, Schoenwald S, et al. Assessing the Organizational Social Context (OSC) of mental health services: implications for research and practice. Adm Policy Ment Health. 2008;35:98-113.

4. Saldana L. The stages of implementation completion for evidence-based practice: protocol for a mixed methods study. Implement Sci. 2014;9:43.

## A65 Organizational-level factors that predict implementation of an autism evidence-based intervention in public schools

### Jill Locke^1^, Cristine Oh^2^, Rinad Beidas^3^, Steven Marcus^3^, Aaron Lyon^1^, Gregory Aarons^4^, Aubyn Stahmer^5^, Shannon, Dorsey^1^, David Mandell^3^

#### ^**1**^University of Washington, Seattle, WA, USA; ^2^University of Pittsburgh, Pittsburgh, PA, USA; ^3^University of Pennsylvania, Philadelphia, PA, USA; ^4^University of California, San Diego, La Jolla, CA, USA; ^5^University of California, Davis, CA, USA

##### **Correspondence:** Jill Locke (jill.locke@gmail.com)

**Background**

The purpose of this study was to examine organizational characteristics associated with the implementation of an evidence-based intervention (EBI) for children with autism spectrum disorder (ASD) in public schools. Although many interventions for children with ASD have shown efficacy in university-based research settings, few have been effectively implemented and sustained in schools, the primary setting in which children with ASD receive services. Organizational characteristics have been shown to predict the implementation of EBIs for the prevention and treatment of other problems in schools, and may play a role in the successful use of autism EBIs in schools; however, these factors have not been systematically studied within this context.

**Materials and methods**

Participants included 37 principals, 50 teachers and 75 classroom staff from 37 under-resourced public schools in Philadelphia, PA. Independent observers rated teachers’ implementation of several EBIs in which the teachers had been trained using a fidelity checklist. Participants completed ratings of organizational characteristics (i.e., organizational culture, organizational climate, implementation climate, and leadership).

**Results**

Preliminary descriptive analyses indicate that: 1) ratings of implementation climate were similar across principals, teachers, and other staff; and 2) ratings of leadership were highest among principals followed by teachers and other staff. A linear regression with random effects for classroom and school (to account for classrooms nested within schools) will be conducted to examine individual associations between each organizational-level factor (i.e., organizational culture, implementation climate, and leadership) and each component of fidelity.

**Conclusions**

The results of this study will provide an in-depth understanding of organizational factors that influence the successful implementation of EBIs for children with ASD in under-resourced public schools. These data will help identify implementation intervention targets that will facilitate the development of strategies to help schools overcome barriers to implementation and ultimately improve the outcomes of children with ASD.

## A66 Effects of training and organizational factors on staff turnover in a large-scale implementation initiative

### Laurel Brabson, Amy Herschell

#### West Virginia University, Morgantown, West Virginia, USA

##### **Correspondence:** Laurel Brabson (labrabson@mix.wvu.edu)

**Background**

PCIT Across PA is a large-scale, NIMH funded (R01 MH095750) implementation trial with the goals of: 1) implementing Parent-Child Interaction Therapy (PCIT) across the state of Pennsylvania, and 2) investigating the effectiveness of three different training models in promoting clinician use of PCIT. Staff turnover rates are notoriously high within the field of behavioral health, which can be especially problematic in implementation efforts when the adoption and sustainability of a new intervention is contingent upon a stable workforce. The current study seeks to understand individual-level (e.g., salary, education level) and organizational-level factors (organizational culture and climate) that influence staff turnover within large-scale implementation initiatives. Given the focus on training methods, the current study also seeks to understand the effect of different training methods on clinician turnover.

**Materials and Methods**

Participants (n=102 clinicians, n=54 supervisors, n=50 administrators) were randomized to one of three training conditions. Information about turnover was collected at 6-months (mid-training), 12-months (post-training), 24-months (1-year follow up), and was supplemented by research staff recording details about turnover when they learned of a staff member leaving an agency.

**Results**

Data collection was recently completed; data is currently being cleaned, preliminary analyses have been complete, and primary analyses will be completed soon. Given the nested structure of the data, Hierarchical Linear Modeling (HLM) will be used to understand the influence of training condition, individual factors, and organizational factors on staff turnover. Preliminary analyses suggest that training condition may impact supervisor and administrator turnover, while organizational factors may impact clinician turnover.

**Conclusions**

High rates of staff turnover are common in most treatment settings within the behavioral health field and are problematic for agencies and for clients. Results of the current study will help to identify predictors of staff turnover within implementation initiatives. Given that training is one of the most critical factors in the early implementation stages, the focus on training methods within the current study will help to uncover any possible protective effects of specific training methods on staff turnover, which will ultimately improve the sustainability of the intervention.

## A67 National implementation of a suicide prevention outreach program in the Department of Veterans Affairs: Perspectives from an operational partner

### Aaron Eagan (aaron.eagan@va.gov)

#### Department of Veterans Affairs, Office of Suicide Prevention, Washington DC, USA

**Background**

The Department of Veterans Affairs (VA) has identified suicide prevention as a top priority. To improve prevention, it is critical to identify patients at risk as early as possible and before suicide related events occur. To that end, VA developed and validated a predictive model that uses medical record data to identify veterans at risk [1]. For those identified as high risk, VA’s Office of Suicide Prevention is implementing a national suicide prevention outreach program entitled Recovery Engagement and Coordination for Health – Veterans Enhanced Treatment (REACH VET). This program includes identification, re-evaluation of care, and care enhancements as appropriate.

**Materials and Methods**

Implementation strategies used in the initial roll out of REACH VET included policy memos, identification of a coordinator at every VA medical center, creation of a web-based dashboard to provide names, web-based training of coordinators, creation of support materials, and technical assistance. The dashboard allowed for tracking of coordinator and provider actions.

**Results**

Initial roll out of REACH VET was impacted by a number of factors at the national level, including national leadership priorities, changes in resources available, and political factors. Initial implementation of the program varied across facilities, with some sites implementing fully and others needing more assistance to implement.

**Conclusions**

Given that some facilities need more assistance to implement REACH VET, additional implementation support through a virtual external facilitation strategy is being offered [2,3]. The operational partner’s perspective on this national implementation and evaluation will be presented.

**References**

1. McCarthy JF, Bossarte RM, Katz IR, Thompson C, Kemp J, Hannemann CM, et al. Predictive Modeling and Concentration of the Risk of Suicide: Implications for Preventive Interventions in the US Department of Veterans Affairs. Am J Public Health. 2015;e1-8.

2. Stetler CB, Legro MW, Rycroft-Malone J, Bowman C, Curran G, Guihan M, et al. Role of “external facilitation” in implementation of research findings: a qualitative evaluation of facilitation experiences in the Veterans Health Administration. Implement Sci. 2006;1:23.

3. Ritchie MJ, Dollar KM, Kearney LK, Kirchner JE. Responding to needs of clinical operations partners: transfer- ring implementation facilitation knowledge and skills. Psychiatr Serv. 2014;65:141-3.

## A68 Randomized program evaluation of national implementation of a suicide prevention outreach program in the Department of Veterans Affairs: Initial outcomes and experiences in partnered research

### Sara J. Landes^1,2,3^ (sara.landes@va.gov)

#### ^1^VA Quality Enhancement Research Initiative (QUERI) Program for Team-Based Behavioral Health, Department of Veterans Affairs, Little Rock, AR, USA; ^2^Department of Psychiatry, University of Arkansas for Medical Sciences, Little Rock, AR, USA; ^3^VISN 16 South Central MIRECC, Little Rock, AR, USA

**Background**

Facilitation is an evidence-based implementation strategy to support sites that have difficulty implementing innovative programs [1, 2]. Facilitation is a multi-faceted “process of interactive problem solving and support that occurs in the context of a recognized need for improvement and a supportive interpersonal relationship [3].” Virtual external facilitation has been used nationally in the Department of Veterans Affairs (VA) to implement a low complexity intervention [1]. VA’s Office of Suicide Prevention is using virtual external facilitation with a new suicide prevention outreach program entitled REACH VET, a moderately complex innovation targeting a high-risk clinical population.

**Materials and Methods**

A randomized program evaluation is being conducted using a stepped wedge design. Regional networks opting to participate in facilitation will be randomized to when they receive facilitation. Up to four medical centers in each region that are struggling to implement REACH VET will receive facilitation. Facilitation will include an in-person site visit and six months of ongoing virtual support. Implementation fidelity will be measured through completion of coordinator and provider tasks on a web-based dashboard. Facilitator activity will be recorded via a time tracking log and weekly debrief interviews.

**Results**

Initial results will be presented on the implementation of REACH VET nationally, including facilities not receiving facilitation. Initial results of sites receiving facilitation will be presented, along with data on the time and types of activities occurring during facilitation.

**Conclusions**

Virtual external facilitation is an implementation strategy that can be helpful in assisting facilities struggling to implement a new intervention. The virtual component is especially helpful in a nationwide healthcare system with limited resources for travel. We will discuss the evaluation team’s experience working with an operational partner to plan a national program evaluation.

**References**

1. Kilbourne AM, Abraham KM, Goodrich DE, Bowersox NW, Almirall D, Lai Z, et al. Cluster randomized adaptive implementation trial comparing a standard versus enhanced implementation intervention to improve uptake of an effective re-engagement program for patients with serious mental illness. Implement Sci. 2013;8:1-14.

2. Kirchner JE, Ritchie MJ, Pitcock JA, Parker LE, Curran GM, Fortney JC. Outcomes of a partnered facilitation strategy to implement primary care–mental health. J Gen Intern Med. 2014;29:904-12.

3. Powell BJ, Waltz TJ, Chinman MJ, Damschroder LJ, Smith JL, Matthieu MM, Proctor EK, Kirchner JE. A refined compilation of implementation strategies: results from the Expert Recommendations for Implementing Change (ERIC) project. Implement Sci. 2015;10:21.

## A69 Virtual external facilitation to support implementation of a suicide prevention outreach program in the Department of Veterans Affairs: Facilitation activities and a facilitator’s experience

### Kaily Cannizzaro (kaily.cannizzaro@va.gov)

#### VISN 19 Rocky Mountain MIRECC, Denver, CO, USA

**Background**

Facilitation has been defined as a multi-faceted “process of interactive problem solving and support that occurs in the context of a recognized need for improvement and a supportive interpersonal relationship [1].” Facilitation includes a variety of other strategies and activities, such as provider education, performance monitoring and feedback, stakeholder engagement, facilitating marketing, and formative evaluation. Effective facilitators adapt to each site’s particular circumstances and select from a broad range of strategies. As such, it can often be difficult to define what takes place during facilitation. The Department of Veterans Affairs (VA) Office of Suicide Prevention is using virtual external facilitation with a new suicide prevention outreach program entitled REACH VET.

**Materials and Methods**

The facilitator team includes one psychologist and two social workers, all with clinical expertise in suicide prevention. Facilitators attended a one-day interactive training that included a virtual trainer to demonstrate how to conduce virtual external facilitation. Facilitators received virtual mentoring as needed following training. In a randomized program evaluation with a stepped wedge design, facilitators will provide virtual external facilitation to 28 sites over a 4-year period. Facilitators are keeping detailed time and activity logs and participating in regular qualitative debriefing interviews that include use of a key implementation event template.

**Results**

Initial results will be presented on the types of activities used for sites receiving facilitation, as well as the time spent. Qualitative data will be presented to elaborate on time and activity logs. One facilitator will present on how activities were chosen for each site.

**Conclusions**

Virtual external facilitation is an implementation strategy that can be tailored to sites in need of implementation support. The facilitator’s perspective on this national implementation and evaluation will be presented.

**Acknowledgments**

This project was funded by the Department of Veterans Affairs (VA) Health Services Research & Development (HSR&D) Service Directed Research (SDR). The results described are based on data analyzed by the authors and do not represent the views of the VA, Veterans Health Administration (VHA), or the United States Government.

**Reference**

1. Powell BJ, Waltz TJ, Chinman MJ, Damschroder LJ, Smith JL, Matthieu MM, Proctor EK, Kirchner JE. A refined compilation of implementation strategies: results from the Expert Recommendations for Implementing Change (ERIC) project. Implement Sci. 2015;10:21.

## A70 Does implementation of evidence-based recommendations for classroom management impact teacher stress?

### Rachel R. Ouellette, Stacy L. Frazier

#### Florida International University, Miami, FL, USA

##### **Correspondence:** Rachel R. Ouellette (rouel001@fiu.edu)

**Background**

Significant time and resources have been invested in bringing evidence-based practice (EBP) for classroom management to schools. Most studies examine teacher adherence to recommendations and their impact on youth outcomes or measure changes in teacher attitudes, knowledge, or skills. Less is understood about the impact of EBP adoption and implementation on teachers’ well-being. Introduction of a new EBP can require extensive training, bring organizational-level changes, and create competing demands in the classroom, potentially introducing increased burden and stress for the teacher. Conversely, perceived improvements in classroom functioning and student engagement may reduce stress and increase teacher efficacy. A growing body of literature in other settings support such positive influences of EBP implementation, revealing decreased emotional exhaustion among providers trained in a new EBP [1].

**Materials and Methods**

Data for the current study comes from a three-year randomized trial examining a school- and home-based mental health service model called Links to Learning [2]. General education teachers (n=71) in Kindergarten to 4th grade classrooms received training and support on four evidence-based classroom recommendations. Teachers reported adherence on monthly checklists as well as their work-related efficacy and stress at the beginning and end of the school year.

**Results**

Overall, positive associations were found between one of the four evidence-based recommendations (Class-wide Peer Tutoring) and teacher reports of quality of work-life. No association was found between reported stress levels and the remaining three recommendations, including the Good Behavior Game, Daily Reported Cards, and Good News Notes. Teacher reported self-efficacy did not appear to mediate this relationship.

**Conclusions**

Previous research in mental health settings has shown that decreasing emotional exhaustion and stress among providers can in turn decrease turnover and increase job productivity. While there is a rich literature in school mental health services examining the transport of EBPs to schools, comparatively little is known about the impact of these efforts on teacher stress. A large and robust literature on the effects of teacher stress and burnout suggests this is something we need to pay more attention to. These findings indicate that certain recommendations may have varying effects on teacher stress.

**References**

1. Aarons GA, Fettes DL, Flores LE, Jr., Sommerfeld DH. Evidence-based practice implementation and staff emotional exhaustion in children’s services. Behav Res Ther. 2009;47(11):954-60.

2. Atkins MS, Shernoff ES, Frazier SL, Schoenwald SK, Cappella E, Marinez-Lora A, et al. Redesigning community mental health services for urban children: Supporting schooling to promote mental health. J Consult Clin Psychol. 2015;83(5):839-52.

## A71 Stuck at the beginning: How the absence of a change mechanism can influence implementation

### Teresa Damush (tdamush@iupui.edu)

#### Veterans Health Administration, HSRD PRIS-M QUERI Center, Indiana University, Indianapolis, IN, USA

**Background**

Goal-setting and ongoing feedback about progress toward those goals is considered a cornerstone strategy for patient behavior change programs [1]. More recently this behavior change strategy has been applied to provider behavior change [2,3]. The use of shared goals for a team of clinical providers with feedback on performance is an implementation strategy that falls into the Inner Setting domain of the Consolidated Framework for Implementation Research (CFIR) [4]. To explore potential mechanisms that explain how this strategy may influence implementation of quality improvement in acute stroke care, we evaluated the use of Goals and Feedback among 11 large, acute health care facilities in the National VHA system.

**Materials and Methods**

A group of 152 clinical staff and management personnel involved in acute stroke care at 11 facilities were interviewed each year across 3 years for a total of 312 interviews. Because acute stroke care spanned roles and services, respondents replied to questions on multidisciplinary collaboration and communication practices.

Audio-recorded interviews were transcribed and coded by a trained team through weekly meetings. Data analysis consisted of qualitative thematic coding and systematic team-based assignment of scores for specific CFIR constructs and level of group organization to improve stroke care for each of the 33 one-year intervals. Using the Group Organization [GO] Score [5], the study team scored each of the facilities on their level of group organization for improving acute stroke care as “advanced,” “intermediate” or “beginning” for each of three one-year intervals covered by the study.

**Results**

The absence of team-based reflecting and evaluating directly connected with a “beginning” level of group organization for improving stroke care during that same interval. Ten of the 12 intervals scored at the beginning level all lacked positive scores for goals and feedback and reflecting & evaluating. Conversely, four of the five intervals that scored “advanced” in group organization scored positively for goals and feedback had established shared goals with a regular feedback process to reflect and evaluate performance to pinpoint subsequent improvement opportunities.

**Conclusions**

The Goals and Feedback implementation strategy may be useful as a mechanism for implementing change in an organization by a group of individuals who are committed and may align their individual activities to obtain this shared goal. The use of feedback on a regular interval with benchmarks may pinpoint opportunities for process improvement to obtain set goals.

**References**

1. Bandura A. Social foundations of thought and action: a social cognitive theory. Englewood Cliffs, NJ: Prentice- Hall; 1986.

2. Powell B, Waltz TJ, Chinman MJ et al, A refined compilation of implementation strategies: results from the Expert Recommendations for Implementing Change (ERIC) project. Implementation Science, 2015;10:21

3. Michie S, Richardson M, Johnston M. The behavior change technique taxonomy (v1) of 93 hierarchically clustered techniques: building an intervention consensus for the reporting of behavior change interventions. Ann Behav Med. 2013;46(1):81-95. doi: 10.1007/s12160-013-9486-6.

4. Damschroder LJ & Lowery JC. Evaluation of a large-scale weight management program using the consolidated framework for implementation research (CFIR). Implement Sci. 2013;8:51.

5. Miech E, Damush T. Applying the Consolidated Framework for Implementation Research constructs directly to qualitative data: the power of implementation science in action. Proceedings of the 3rd Biennial Conference of the Society for Implementation Research Collaboration (SIRC) 2015: advancing efficient methodologies through community partnerships and team science. Implement Sci. 2016;11(Suppl 1):85.

## A72 Stressed out: Examining work-stress reduction as mechanism for improved implementation

### Madeline Larson^1^, Clayton Cook^1^, Aria Fiat^1^, Aaron Lyon^2^

#### ^**1**^University of Minnesota, Minneapolis, MN, USA; ^2^University of Washington, Seattle, WA, USA

##### **Correspondence:** Madeline Larson (lars5424@umn.edu)

**Background**

Addressing implementation difficulties after active implementation has begun can be critical to the successful use of evidence-based practices (EBPs) [1]. Identifying malleable tailoring variables that explain why a particular provider is failing to deliver an innovation with adequate fidelity can inform more precise implementation strategies. One factor that may impact EBP implementation is stress related to work-overload. While studies have shown that work-related stress can impact provider buy-in and intentions to implement [2,3], no studies to date have examined the functional association between work-related stress and EBP implementation. The purpose of this study was to examine experimentally the impact of stress reduction via wellness coaching on the fidelity of evidence-based behavior management practices in a school setting.

**Materials and Methods**

Four teachers identified with high ratings of work-related stress and low implementation fidelity participated in the study. An intervention fidelity rubric was developed using established guidelines [4] and gathered daily. Work-related stress was assessed weekly using the subjective units of distress scales, adapted to address work stress specifically [5]. Following baseline data collection, participating teachers received wellbeing coaching [6]. At the outset of coaching, teachers selected wellbeing-promoting practices from a menu of possible supports (i.e., values clarification, mindfulness, gratitude, emotion management, therapeutic lifestyle choices, and social connections). Coaching was then tailored based on chosen wellbeing practices and level of reported work-related stress. During coaching sessions, counselors used a variety of coaching practices (e.g., motivational interviewing) to facilitate reflection and evoke change talk. To evaluate the impact of wellbeing coaching on teachers’ stress reduction and improved EBP fidelity, a single-case concurrent multiple baseline design (MBD) across participants was utilized.

**Results**

Visual analysis of the MBDs revealed a functional relation between the introduction of the wellbeing coaching, reductions in all four teachers’ stress ratings, and improvement in intervention fidelity. Together, findings suggested a causal relationship between stress reduction and intervention fidelity, such that decreases in teachers’ stress corresponded to improvements in the delivery of the classroom-based EBPs with fidelity.

**Conclusions**

Findings highlight the relationship between work-related stress and EBP implementation, with decreased stress serving as potential a mechanism by which intervention fidelity can be improved. Those supporting providers to implement EBPs during active implementation may attend to work-related stress in order to enhance implementation and ultimately improve outcomes for service recipients. Building on these findings, the presentation will focus on ways researchers and practitioners can utilize stress reduction as a mechanism of action for precision implementation interventions.

**References**

1. Novins DK, Green AE, Legha RK, Aarons, GA. Dissemination and implementation of evidence-based practices for child and adolescent mental health: a systematic review. J Am Acad Child Adolesc Psychiatry. 2013;*52*(10):1009-25.

2. Margolis J, Nagel L. Education reform and the role of administrators in mediating teacher stress. TEQ. 2006;*33*(4):143-59.

3. Ross SW., Romer N, Horner RH. Teacher well-being and the implementation of school-wide positive behavior interventions and supports. J Posit Behav Interv. 2012;14(2):118-28.

4. Sanetti LMH, Kratochwill R. Treatment integrity assessment in the schools: An evaluation of the treatment integrity planning protocol. Psych Schol Q. 2009;24(1):24-35. doi: 10.1037/a0015431.

5. Wolpe J. The practice of behavior therapy. New York, NY: Pergamon Press; 1969.

6. Cook CR, Miller FG, Fiat A, Renshaw T, Frye M, Joseph G, Decano P. Promoting secondary teachers’ wellbeing and intentions to implement evidence-based practices: randomized evaluation of the achiever resilience curriculum. Psychol Sch. 2017;54(1):13-28. doi 10.1002/pits.21980

## A73 When implementation can’t wait: Focusing on the impact of context

### Suzanne Kerns^1,2^, Michael Pullmann^1^, Barb Putnam^3^, Paul Davis^4^, Jacqueline Uomoto^1^, Jedediah Jacobson^1^, Barbara Lucenko^5^, Lucy Berliner^6^

#### ^1^University of Washington, Seattle, WA, USA; ^2^University of Denver, Denver, CO, USA; ^3^Washington State DSHS Children’s Administration, Kent, WA, USA; ^4^Washington State DSHS Division of Behavioral Health and Recovery, Olympia, WA, USA; ^5^Washington State DSHS Research and Data Analysis Division, Olympia, WA, USA; ^6^University of Washington Medicine Harborview Center for Sexual Assault and Traumatic Stress, Seattle, WA, USA

##### **Correspondence:** Suzanne Kerns (Suzanne.Kerns@du.edu)

**Background**

A hospitable, supportive implementation context is a critical component of implementation success. However, policy and grant-funded initiatives often mandate new approaches regardless of implementation environment. This presentation explores the impact of a complex systems- and practice-level project to embed trauma symptom screening within existing screening protocols, and link screening to targeted case planning for children and youth in foster care in Washington State. Funded by the Administration for Children and Families, this project is a collaborative partnership between the University of Washington, Children’s Administration (CA), and Division of Behavioral Health and Recovery. Readiness activities were initiated, including stakeholder meetings and pre-training workshops. However, time constraints and funding expectations necessitated proceeding with implementation despite variable readiness. We explore the implications of this common situation.

**Materials and Methods**

There were three different implementation contexts: 1) An existing high-functioning screening infrastructure supported the dissemination of a new trauma screening tool at entry into foster care, 2) A system to conduct mental health screening with youth 6 months after entry into care was a newly developed innovation that had no existing infrastructure, but was within the control of principal investigators; and 3) Supporting case-level connections between child welfare and mental health and required complex cross-system effort outside of the control of principal investigators. Approximately 200 social workers, 100 mental health professionals, and 20 screening staff periodically participated in surveys and focus groups over three years. Questions included satisfaction with the implementation approach and subsequent changes to the screening procedure, level and usefulness of collaboration and communication between mental health and child welfare agencies, training satisfaction, and social worker measures of the use of screens to drive case planning and mental health referral. System-wide outcomes were evaluated by comparing rates of children and youth receiving screening and subsequent mental health services before and after intervention implementation.

**Results**

Various support approaches were provided to those administering the screening tool. While there was mixed satisfaction at initial implementation, three years’ post-implementation compliance to the screening procedure was high. Implementation success was also high for training efforts and development of the ongoing screening program. Yet, there was very little progress made in enhancing the service array and improving case-level communication and collaboration across systems.

**Conclusions**

Current implementation science frameworks need to account for flexible implementation environments and consider differential impacts of implementation support efforts within varying environments. “Cross-silo” work requires enhanced and potentially specialized implementation support.

## A74 Transforming a plan into real practice change: The role and influence of child welfare supervisors in implementation

### Alicia C. Bunger^1^, Sarah A. Birken^2^, Jill A. Hoffman^3^, Mimi Choy-Brown^4^, Christy Kranich^1^, Byron J. Powell^2^

#### ^1^Ohio State University, Columbus, OH, USA; ^2^Department of Health Policy and Management, Gillings School of Global Public Health, University of North Carolina at Chapel Hill, Chapel Hill, NC, USA; ^3^School of Social Work, Portland State University, Portland, OR, USA; ^4^Silver School of Social Work, New York University, New York, NY, USA

##### **Correspondence:** Alicia C. Bunger (bunger.5@osu.edu)

**Background**

Child welfare supervisors play an essential role in implementation by disseminating, synthesizing, and justifying implementation details as well as translating top management’s project plans to front-line workers [1]. Through these roles, supervisors shape the climate for implementation – i.e., the degree to which innovations are expected, supported, and rewarded [2]. Although executive leadership’s influence on climate has been examined, the role of supervisors proximal to the front-lines has received less attention. This study illustrates child welfare supervisors’ implementation roles and explores their influence on climate.

**Materials and Methods**

A sequenced behavioral health screening and assessment intervention was implemented within a county-based child welfare agency. We conducted six focus groups with supervisors and front-line workers from implementing work-units six months post-implementation (*n*=51) and one year later (*n*=40) (12 groups total). Participants were asked about implementation barriers and facilitators. We audio-recorded, transcribed, and analyzed focus groups using an open coding process during which the importance of supervisors’ roles emerged as a major theme. We further analyzed this code using concepts and definitions related to middle managers’ roles and implementation climate.

**Results**

Supervisors filled four roles that target implementation climate. First, supervisors disseminated information about the screening and assessment tools proactively and in response to worker questions, which reinforced formal trainings. Second, supervisors synthesized information and supported workers’ application of screening and assessment procedures during supervision, and by troubleshooting complex cases. Third, supervisors justified implementation by explaining the role of trauma on behavioral health problems and children’s outcomes using tailored messaging based on staff’s perceived commitment, experience, and knowledge. These three roles support workers’ knowledge and innovation use. Fourth, supervisors translated top managements’ project plans into action by monitoring workers’ use of the screenings and assessments, issuing reminders, advocating for resources, reinforcing standards for practice change, and praising workers, which conveyed expectations and rewards for innovation use.

**Conclusions**

Child welfare supervisors support workers’ as they learn and apply innovations, reinforce expectations, and reward their performance, thus linking top management with the front-lines. Through these roles supervisors shape implementation climate, and, in turn, implementation effectiveness, and children’s outcomes. Results suggest that implementation climate may mediate supervisors’ influence on implementation effectiveness.

**References**

1. Birken SA, Lee S-YD, Weiner BJ. Uncovering middle managers’ role in healthcare innovation implementation. Implement Sci. 2012;7(1):28. doi:10.1186/1748-5908-7-28.

2. Weiner BJ, Belden CM, Bergmire DM, Johnston M. The meaning and measurement of implementation climate. Implement Sci. 2011;6(1):78. doi:10.1186/1748-5908-6-78.

## A75 Clinician and agency factors associated with implementation outcomes in learning collaboratives

### Jason Lang^1^, Christian Connell^2^, Kyle Barrette^1^

#### ^1^Child Health and Development Institute, Farmington, CT, USA; ^2^Yale School of Medicine, New Haven, CT, USA

##### **Correspondence:** Jason Lang (jalang@uchc.edu)

**Background**

Significant federal and state efforts have promoted implementation of evidence-based treatments (EBTs) for children with behavioral health concerns. However, availability of EBTs remains very limited in community settings, and may even be decreasing [1,2]. Learning Collaboratives (LCs) are a promising approach that has been used for disseminating EBTs with initial evidence of success [3,4]. However, Learning Collaboratives still often result in widely variable adoption rates, and little is known about how clinician- and organizational level factors change during implementation and are associated with implementation outcomes in Learning Collaboratives.

**Materials and Methods**

Data are presented from 98 clinicians from 13 agencies who completed pre- and post- implementation surveys as part of training in Trauma-Focused Cognitive Behavioral Therapy (TF-CBT) through Learning Collaboratives intended to provide TF-CBT to children in the child welfare system. Clinicians reported on prior training experience, theoretical orientation, commitment to TF-CBT, perceptions of organizational commitment and support, and measures of attitudes towards EBTs (Evidence-Based Practice Attitude Scale [EBPAS]), trauma-informed care (Trauma Systems Readiness Tool [TSRT] and Trauma Informed System of Care Instrument [TISCI]), and collaboration with child welfare (Levels of Collaboration Scale [LOC], Interagency Collaboration Activities Scale [IACAS]). During and following implementation, clinicians report data about children served with EBTs in a statewide administrative data system, including number served, dose/fidelity, satisfaction, and standardized clinical outcome measures.

**Results**

Initial results show that clinicians participating in a Learning Collaborative reported significant improvements in exposure to trauma-related content (TSRT) and self-reported agency policy and individual and agency-level practice related to trauma (TISCI), and for most aspects of collaboration (LOC and IACAS). Commitment to TF-CBT was unchanged, likely due to high baseline ratings. No changes in attitudes about EBTs were observed (EBPAS), and there was a trend effect towards decreases in positive attitudes about EBTs. Analysis of implementation and outcomes data from 797 children receiving TF-CBT are under way. Analysis will examine the effects of prior clinician training and experience as well as baseline and change scores on staff knowledge, attitudes, and perceptions of leadership and organizational support on implementation and child outcomes, including use of TF-CBT, fidelity, and clinical outcomes.

**Conclusions**

Clinicians participating in a Learning Collaborative demonstrated significant improvements in trauma knowledge, individual and agency practice, policy, and collaboration, but not in attitudes about EBTs. Clinicians provided TF-CBT to at least 797 children. Clinician-reported commitment to the EBT was associated with greater implementation number of youth served. Individual trauma practice was associated with more completed cases and self-reported fidelity. Agency policy was negatively associated with cases completed. The findings suggest that assessing commitment to a specific EBT may be more helpful than assessing attitudes about EBTs broadly, and that clinicians’ reports of agency practices and policies may not be effective predictors of implementation.

**References**

1. Bruns EJ, Kerns SE, Pullmann MD, Hensley SW, Lutterman T, Hoagwood KE. Research, data, and evidence-based treatment use in state behavioral health systems, 2001 to 2012. Psychiatr Serv. 2015;67(5):496-503.

2. Kazak AE, Hoagwood K, Weisz JR, Hood K, Kratochwill TR, Vargas LA, & Banez GA. A meta-systems approach to evidence-based practice for children and adolescents. Am Psychol. 2010;65(2):85-97. doi:10.1037/a0017784

3. Ebert L, Amaya-Jackson L, Markiewicz JM, Kisiel C, Fairbank JA. Use of the breakthrough series collaborative to support broad and sustained use of evidence-based trauma treatment for children in community practice settings. Adm Policy Ment Health. 2012;39(3):187-99. doi: 10.1007/s10488-011-0347-y.

4. Lang, JM, Franks, RP, Epstein, C, Stover, C, Oliver, JA. Statewide dissemination of an evidence-based practice using Breakthrough Series Collaboratives. Children and Youth Services Review, 2015;55:201-9.

## A76 Choosing implementation strategies to address local contextual barriers

### Laura Damschroder^1^, Thomas Waltz^2^, Brenton Abadie^2^, Byron J. Powell^3^

#### ^**1**^VA Center for Clinical Management Research, Fairfax, VA, USA; ^2^Psychology Department, Eastern Michigan University, Ypsilanti, MI, USA; ^3^Department of Health Policy and Management, Gillings School of Global Public Health, University of North Carolina at Chapel Hill, Chapel Hill, NC, USA

##### **Correspondence:** Laura Damschroder (Laura.Damschroder@va.gov)

**Background**

A top priority for implementation researchers is to provide guidance for tailoring implementation strategies to local contexts when implementing evidence-based innovations. The Consolidated Framework for Implementation Research (CFIR) is comprised of 39 constructs believed to influence implementation. It has been used across the world to assess local contexts including identifying potential barriers to implementation. However, the CFIR does not specify what strategies to use to mitigate identified barriers. The Expert Recommendations for Implementing Change (ERIC) implementation strategy compilation includes 73 strategies but it does not specify which strategy to use in which contexts. The aim of this project was to elicit recommendations from experts about which ERIC strategies would best address each CFIR barrier.

**Materials and Methods**

Participants were recruited from an international list of 435 implementation researchers and practitioners. Willing participants were randomly assigned a contextual barrier based on the CFIR and asked to select and rank up to 7 ERIC strategies they believed would best address that barrier. The barriers were presented in random order and participants were able to decide how many CFIR constructs they wished to address.

**Results**

Of 169 participants, 85% self-identified as being an implementation expert, 66% were outside VA, and 17% were from outside the US. At least 20 participants selected ERIC strategies for each of the 39 CFIR constructs, 21 of which had strategies that a majority endorsed. The strategy most often recommended, Identify and Prepare Champions, was endorsed by a majority of respondents for 5 different barriers. Of the 2847 possible combinations of 73 ERIC strategies and 39 CFIR constructs, at least one respondent endorsed at least one strategy for 1832 of those combinations (64%). ERIC strategies within the Develop Stakeholder Interrelationships thematic cluster (n=13 strategies) together, had among the highest endorsement for all CFIR barriers and received majority endorsement 7 barriers. The three top reasons strategies were chosen were: relevance, feasibility, and potential impact.

**Conclusions**

Participating experts had wide divergence in recommended strategies across the CFIR barriers. However, a majority of participants did endorse a total of 33 ERIC strategies that each addresses one or more of 21 CFIR barriers (1-3 strategies per barrier). All CFIR barriers have at least four ERIC strategies that were endorsed by at least 25% of participants. Based on these results, a high-level algorithm has been developed to help guide users to select strategies with the highest degree of endorsement based on contextual barriers.

**References**

1. Damschroder LJ, Aron D, Keith R, Kirsh S, Alexander J, Lowery J. Fostering implementation of health services research findings into practice: a consolidated framework for advancing implementation science. Implement Sci. 2009;4(1):50.

2. Powell BJ, Waltz TJ, Chinman MJ, Damschroder LJ, Smith JL, Matthieu MM, Proctor EK, Kirchner JE. A refined compilation of implementation strategies: results from the Expert Recommendations for Implementing Change (ERIC) project. Implement Sci. 2015;10:21.

3. Waltz TJ, Powell BJ, Matthieu MM, Damschroder LJ, Chinman MJ, Smith JL, Proctor EK, Kirchner JE. Use of concept mapping to characterize relationships among implementation strategies and assess their feasibility and importance: results from the Expert Recommendations for Implementing Change (ERIC) study. Implement Sci. 2015;10:109.

## A77 Developing implementation strategies with stakeholders to promote firearm safety as a suicide prevention strategy in pediatric primary care

### Rinad S. Beidas^1^, Brittany Courtney Benjamin Wolk^1^, Shari Jager-Hyman^1^, Steven C. Marcus ^2^, Brian K. Ahmedani ^3,^ John E. Zeber ^4^, Joel A. Fein^5,^ Gregory K. Brown ^1^, Adina Lieberman^1^

#### ^1^Department of Psychiatry, University of Pennsylvania Perelman School of Medicine, Philadelphia, PA, USA; ^2^School of Social Policy and Practice, University of Pennsylvania, Philadelphia, PA, USA; ^3^Henry Ford Health System, Center for Health Policy & Health Services Research and Behavioral Health Services, One Ford Place, Detroit, MI, USA; ^4^Center for Applied Health Research, Baylor Scott & White Health, jointly with Central Texas Veterans Health Care System, Temple, TX, USA; ^5^Division of Emergency Medicine, The Children’s Hospital of Philadelphia, Department of Pediatrics, University of Pennsylvania Perelman School of Medicine, Philadelphia, PA, USA

##### **Correspondence:** Rinad S. Beidas (rbeidas@upenn.edu)

**Background**

The promotion of safe firearm practices, or firearms means restriction, is a promising but infrequently used suicide prevention strategy in the United States. Safety Check is an evidence-based practice for improving parental firearm safety behavior in pediatric primary care [1]. However, providers rarely discuss firearm safety during visits, suggesting the need to better understand barriers and facilitators to promoting this approach [2,3] This study, Adolescent Suicide Prevention In Routine clinical Encounters (ASPIRE), aims to engender a better understanding of how to implement the three firearm components of Safety Check as a suicide prevention strategy in pediatric primary care.

**Materials and Methods**

The NIMH-funded Mental Health Research Network (MHRN), a consortium of 13 healthcare systems across the United States, affords a unique opportunity to better understand how to implement a firearm safety intervention in pediatric primary care from a system-level perspective. As part of Project ASPIRE, we are collaboratively developing implementation strategies in partnership with MHRN stakeholders. First, we surveyed leadership of 82 primary care practices (i.e., practices serving children, adolescents, and young adults) within two MHRN systems to understand acceptability and use of the three firearm components of Safety Check (i.e., screening, brief counseling around firearm safety, provision of firearm locks). Then, in collaboration with MHRN stakeholders, we will use intervention mapping [4] and the Consolidated Framework for Implementation Research [5] to systematically develop and evaluate a multi-level menu of implementation strategies for promoting firearm safety as a suicide prevention strategy in pediatric primary care.

**Results**

Responses from surveys have been received from 40 physician leaders across the 2 systems (70% response rate) and 100 primary care physicians (49% response rate). Physician leaders generally endorsed that the Safety Check is acceptable from their perspective, but that it would not be acceptable to the doctors in their site. Primary care physicians endorsed that the Safety Check is acceptable from their perspective, but that it would not be acceptable to their patients and their parents. Both sets of participants endorsed that the components of the Safety Check are rarely used. Qualitative interviews with nine stakeholder groups are ongoing.

**Conclusions**

This study will provide important insights into acceptability and current use of evidence-based practices for safe firearm practices in pediatric primary care for suicide prevention. We will also outline our approach to collaboratively developing implementation strategies with stakeholders across two large systems using a systematic and mixed- methods approach.

**References**

1. Barkin SL, Finch SA, Ip EH, Scheindlin B, Craig JA, Steffes J, Weiley V, Slora E, Altman D, Wasserman RC. Is office-based counseling about media use, timeouts, and firearm storage effective? results from a cluster-randomized, controlled trial. Pediatrics. 2008;122(1):e15-25.

2. Barber CW, Miller MJ. Reducing a suicidal person’s access to lethal means of suicide: a research agenda. Am J Prev Med. 2014;47(3 Suppl 2):028.

3. Glenn CR, Franklin JC, Nock MK. Evidence-based psychosocial treatments for self-injurious thoughts and behaviors in youth. J Clin Child Adolesc Psychol. 2015;44(1):1-29.

4. Bartholomew LK, Parcel GS, Kok G, Gottlieb NH, Fernandez ME. Planning health promotion programs: an intervention mapping approach. San Francisco, CA: Jossey-Bass; 2016.

5. Damschroder LJ, Aron DC, Keith RE, Kirsh SR, Alexander JA, Lowery JC. Fostering implementation of health services research findings into practice: a consolidated framework for advancing implementation science. Implement Sci. 2009;4(1):50.

## A78 Tailored implementation approaches using mixed methods and implementation teams

### Cara C. Lewis^1,2,3^ (lewis.cc@ghc.org)

#### ^1^Kaiser Permanente Washington Health Research Institute, Seattle, WA USA; ^2^Department of Brain and Psychological Sciences, Indiana University, Bloomington, IN, USA; ^3^Department of Psychiatry and Behavioral Sciences, University of Washington, Seattle, WA, USA

**Background**

There is some evidence that tailored implementation approaches outperform standardized implementation, but no studies, to our knowledge, focus on implementation in behavioral health where interventions tend to be more complex [1]. Moreover, the extant literature lacks transparency in the details surrounding the method used to prospectively identify determinants of practice, their influence on implementation strategy selection, and processes for supporting the implementation. This talk will put forth two related methods for tailoring implementation strategies to the contextual determinants of practice in behavioral health settings.

**Materials and Methods**

The first study is a dynamic cluster randomized trial in which tailored versus standardized approaches to implementing measurement based care for depressed adults are compared across 12 clinics in the nation’s largest not-for-profit behavioral health service provider [2]. The standardized approach included “best practices” of implementation including expert-led training with active learning, consultation, a guideline, clinical decision support, and electronic health record enhancements. The tailored approach used rapid ethnography and mixed methods needs assessment procedures to prospectively identify determinants of practice guided by an established implementation model [3]. Stakeholders were invited to join an implementation team that met monthly to select and employ strategies that were tailored to the identified determinants and informed by penetration and fidelity data reports.

**Results**

Results will be presented with respect to differences in measurement based care fidelity between conditions. The second study employed a similar prospective, mixed methods, model-based tailoring approach to implementing Cognitive Behavioral Therapy (CBT) in youth residential centers [4]. In this study, two sites (one secure and one non-secure) created implementation teams who engaged in conjoint analysis to prospectively generate a tailored blueprint that outlined implementation strategies to be employed across three phases: pre-implementation, implementation, and sustainment. Results regarding reduction in barriers to implementation, provider knowledge and self-reported skill in using CBT will be presented as evidence for this approach’s effectiveness.

**Conclusions**

This presentation will reveal a pragmatic approach to tailoring implementation to determinants of practice for use in behavioral health settings and beyond.

**References**

1. Baker R, Camosso-Stefinovic J, Gillies C, Shaw EJ, Cheater F, Flottorp S, et al. Tailored interventions to address determinants of practice. Cochrane Database Syst Rev. 2015;(4):CD05470

2. Lewis CC, Scott K, Marti CN, Marriott BR, Kroenke K, Putz JW, Mendel P, Rutkowski D. Implementing mea- surement-based care (iMBC) for depression in community mental health: a dynamic cluster randomized trial study protocol. Implement Sci. 2015;10(1):127.

3. Mendel P, Meredith LS, Schoenbaum M, Sherbourne CD, Wells KB. Interventions in organizational and community context: a framework for building evidence on dissemination and implementation in health services research. Adm Policy Ment Health. 2008;35(1-2):21-37.

4. Lewis C, Darnell D, Kerns S, Monroe-DeVita M, Landes SJ, Lyon AR, et al. Proceedings of the 3rd Biennial Conference of the Society for Implementation Research Collaboration (SIRC) 2015: advancing efficient methodologies through community partnerships and team science. Implement Sci. 2016;11(1):85.

## A79 The Collaborative Organizational Approach to Selecting and Tailoring Implementation Strategies (COAST-IS)

### Byron J. Powell^1^, Gregory A. Aarons^2^, Lisa Amaya-Jackson^3^, Amber Haley^1^, Bryan J. Weiner^4^

#### ^1^Department of Health Policy and Management, Gillings School of Global Public Health, University of North Carolina at Chapel Hill, Chapel Hill, NC, USA; ^2^Department of Psychiatry, School of Medicine, University of California, San Diego, La Jolla, CA, USA; ^3^Department of Psychiatry & Behavioral Sciences, Duke University School of Medicine, Durham, NC, USA; ^4^Department of Global Health and Department of Health Services, University of Washington, Seattle, WA, USA

##### **Correspondence:** Byron J. Powell (bjpowell@unc.edu)

**Background**

Implementing and sustaining Trauma-Focused Cognitive Behavioral Therapy and other evidence-based programs with fidelity may require that multiple implementation strategies be selected and tailored to address multilevel, context-specific determinants (barriers and facilitators). Ideally, the selection and tailoring of implementation strategies would be guided by theory, evidence, and input from relevant stakeholders; however, methods to guide the selection and tailoring of strategies are not well-developed. The purpose of this study is to partner with the North Carolina Child Treatment Program and the National Child Traumatic Stress Network to develop and pilot the Collaborative Organizational Approach to Selecting and Tailoring Implementation Strategies (COAST-IS).

**Materials and Methods**

The COAST-IS intervention will involve coaching organizational leaders and therapists to use Intervention Mapping to select and tailor strategies. Intervention Mapping is a multistep process that is inherently ecological and incorporates theory, evidence, and stakeholder perspectives to ensure that intervention components effectively address key determinants of change. After collaboratively developing COAST-IS in Year 1, we will conduct a randomized pilot trial of the intervention within a North Carolina Child Treatment Program learning collaborative, randomly assigning eight organizations to the learning collaborative-only condition or the learning collaborative plus COAST-IS condition.

**Results**

The study results will focus on: 1) the acceptability, appropriateness, feasibility, and utility of COAST-IS; 2) organizational stakeholders’ fidelity to the core elements of Intervention Mapping; and 3) the feasibility of recruitment, randomization, retention, and data collection procedures. Findings will inform the refinement of the COAST-IS intervention and study procedures in preparation for a larger effectiveness trial.

**Conclusions**

This work is significant because it will yield a systematic method that integrates theory, evidence, and stakeholder perspectives to improve the effectiveness and precision of implementation strategies. Ultimately, COAST-IS may have the potential to improve implementation and sustainment of a wide-range of evidence-based practices in mental health and other health sectors.

## A80 Getting back to primary care after a non-VA hospitalization: Provider, staff, and patient perspectives of transitional care for veterans

### Roman Ayele^1,2^, Marina McCreight^1^, Emily Lawrence^1^, Kelty Fehling^1^, Russell Glasgow^4^, Borsika Rabin^3,4^, Robert Burke^1,2^, Catherine Battaglia^1,2^

#### ^1^Department of Veterans Affairs, Eastern Colorado Health Care System, Denver, CO, USA; ^2^University of Colorado, Anschutz Medical Campus, Aurora, CO, USA; ^3^Department of Family Medicine and Public Health, School of Medicine, University of California San Diego, La Jolla, CA, USA; ^4^Department of Family Medicine, School of Medicine, University of Colorado, Aurora, CO, USA

##### **Correspondence:** Roman Ayele (roman.ayele@va.gov)

**Background**

Health systems are challenged by the complex process of transitioning patients back to primary care following an out-of-system hospitalization. Poor transitions potentially result in medical complications, patient confusion and dissatisfaction, unnecessary costs, and hospital readmissions. In 2015, approximately 2500 Veterans from the Department of Veterans Affairs (VA) Eastern Colorado Healthcare System were hospitalized in non-VA hospitals making this an important care coordination issue. We used Lean Six Sigma (LSS) approach to identify current state of transitional care along with Practical, Robust Implementation and Sustainability Model (PRISM) framework, which informed the intervention design and implementation process of this quality improvement initiative.

**Materials and Methods**

Guided by LSS method of Define, Measure, Analyze, Improve, Control, we conducted 70 semi-structured interviews with VA and non-VA providers, staff, administrators, and Veterans. LSS tools such as value stream mapping, process mapping, and fishbone diagram were utilized during the pre-implementation current process assessment. To address the complexity of the current process, we performed root cause analysis of the perceived issues as discussed by interviewees and barriers to effective transitions. These assessments were further enriched by the PRISM implementation framework to inform assessment of the current transition process, plan and implement the intervention and de-adopt identified low-value practices as well as system failures.

**Results**

We identified four barriers: 1) Untimely identification and notification of admissions; 2) Non-standardized process for obtaining non-VA prescriptions at the VA pharmacy; 3) Untimely follow-up care with primary care team; and 4) Delay in medical record transfer. To address these care coordination issues, we implemented a nurse coordinator role to manage post-discharge care needs and educate stakeholders about VA processes. System changes were made to facilitate timely notification and medical record transfer. A Care Card given to Veterans upon enrollment in the program contains information that optimizes care coordination. We will use repeated improvement cycles to test the effectiveness and sustainability of the intervention.

**Conclusions**

The lack of a standardized transition process and weak coordination between non-VA inpatient and VA primary care pose a major challenge to better health outcomes. LSS provides tools to inform elements of the PRISM implementation framework that helped design an intervention and implementation strategy. Lessons learned from integrating these two frameworks will guide the use of actionable data to improve patient health outcomes, reduce unnecessary costs and enhance sustainability.

## A81 Can workshop training change community clinicians’ practice? A non-randomized comparison of two workshop lengths for training clinicians in the DBT prolonged exposure protocol for PTSD

### Melanie Harned, Sara Schmidt

#### University of Washington, Seattle, WA, USA

##### **Correspondence:** Melanie Harned (mharned@uw.edu)

**Background**

The dissemination of evidence-based psychotherapies (EBPs) into routine practice requires clinicians who are already working in community settings to be trained to deliver these treatments. The current gold standard of training in EBPs includes a workshop and expert supervision [1]. However, few clinicians have access to EBP experts for supervision and, when available, these services are both costly and time-intensive. As a result, brief continuing education (CE) workshops remain a common method of training community clinicians in EBPs. Despite their widespread use, relatively little research has examined the effectiveness of these types of workshops in changing community clinicians’ practice.

**Materials and Methods**

This observational study evaluated the extent to which a 2-day versus a 4-day workshop in the Dialectical Behavior Therapy Prolonged Exposure (DBT PE) protocol [2] increased adoption, reach, and competence among community clinicians, as well as which clinicians were most likely to change their practice following training. Participants were 254 clinicians from diverse practice settings who were recruited from five CE workshops (2-day: n = 134, 4-day: n =120). Surveys were administered at pre-training, post-training, and 3 and 6 months after training.

**Results**

In the six months after training, the rate of adoption of DBT PE was significantly higher among clinicians attending the 4-day workshop (66.3%) than the 2-day workshop (38.8%; p < .001). Among adopters, clinicians attending the 4-day workshop used DBT PE with significantly more clients (M = 2.8, SD = 2.2) than those attending the 2-day workshop (M = 1.8, SD = 1.5; p < .04). On average, adopters reported ‘often’ to ‘always’ using optimal exposure procedures during DBT PE and this did not differ between workshops. However, the use of suboptimal exposure procedures was significantly more common among clinicians attending the 2-day workshop (‘moderately’) than those attending the 4-day workshop (‘rarely’; p=.01). After adjusting for baseline differences between groups, attending the 2-day workshop and having greater concerns about client worsening predicted suboptimal use of exposure. In contrast, clinicians reporting greater comfort using imaginal and in vivo exposure to treat PTSD at post-training had higher rates of adoption, reach, and optimal use of exposure irrespective of training condition.

**Conclusions**

Brief workshops of varying lengths that emphasize active learning methods can change clinician behavior. However, longer workshops with greater opportunities for active learning may be more effective in reducing suboptimal delivery of EBPs after training.

**References**

1. Beidas RS, Kendall PC. Training therapists in evidence-based practice: a critical review of studies from a systems-contextual perspective. Clin Psychol Sci Prac. 2010;17:1-30.

2. Harned MS, Korslund KE, Linehan MM. A pilot randomized controlled trial of Dialectical Behavior Therapy with and without the Dialectical Behavior Therapy Prolonged Exposure protocol for suicidal and self-injuring women with borderline personality disorder and PTSD. Behav Res Ther. 2014;55:7-17.

## A82 Economic impact of psychology trainees in integrated behavioral health: Implications for pediatric primary care providers

### Alex Dopp, Allison Smith, Aubrey Dueweke, Ana Bridges

#### Department of Psychological Science, University of Arkansas, Fayetteville, AR, USA

##### **Correspondence:** Alex Dopp (dopp@uark.edu)

**Background**

Increasingly, child psychologists are expected to provide services in primary care clinics, integrate with medical teams, and treat youth in a more holistic manner [1,2]. Training future child psychologists in primary care settings will require collaborations between universities and primary care clinics, but many clinics may be unsure of the economic sustainability of hosting student trainees. We investigated whether the demonstrated economic benefits of integrated behavioral health care to providers and patients [3] generalize to services provided by trainees.

**Materials and Methods**

Using a combination of data sources, our study examines the economics of a psychological clerkship within a primary care setting at a Federally Qualified Health Center. Specifically, we will use data drawn from electronic medical records for 40,326 pediatric patient visits completed in the 2015-16 training year, focusing on N = 554 patients (3.69% of all pediatric patients) with at least one behavioral health visit. We will use these data, in combination with documentation files from trainees and clinic-wide annual reports, to compare the costs and benefits of psychology trainees versus full-time behavioral health consultants.

**Results**

To date, we have calculated the annual cost of one psychology trainee ($27,875; based on stipend, tuition, and supervision) versus behavioral health consultant ($62,650; based on salary and benefits), resulting in incremental savings of $3,450 per trainee after accounting for the fact that trainees work half-time. Planned data analyses will compare those incremental cost savings to the incremental benefits, in clinical (i.e., symptom reduction) and economic (i.e., medical cost offset) domains, of the pediatric services provided by psychology trainees versus behavioral health consultants over the training year.

**Conclusions**

Our findings will be of great interest to primary care clinics and graduate programs who wish to establish partnerships for training the next generation of child psychologists in integrated behavioral health care. In particular, our results will inform decisions about sustainability of such training by demonstrating an expected return on investment.

**References**

1. Stancin T, Perrin EC, Ramirez L. Pediatric psychology and primary care. In Roberts M, Steele R, eds. Handbook of pediatric psychology. 4th ed. New York: Guilford Press; 2009. p. 630-48.

2. Hoffses KW, Ramirez LY, Berdan L, Tunick R, Honaker SM, Meadows TJ, et al. Building competency: professional skills for pediatric psychologists in integrated primary care settings. J Pediatr Psychol. 2016; 41(10):1144-60.

3. Blount A, Schoenbaum M, Kathol R, Rollman BL, Thomas M, O’Donohue W, Peek CJ. The economics of behavioral health services in medical settings: a summary of the evidence. Prof Psychol Res Pr, 2007; 38(3):290-7.

## A83 Understanding implementation mechanisms for an evidence-based depression care management program (PEARLS): Ten years of applying implementation science to reach underserved elders

### Lesley Steinman^1^, Mark Snowden^2^

#### ^**1**^University of Washington Health Promotion Research Center, Seattle, WA, USA; ^2^University of Washington Department of Psychiatry and Behavioral Sciences, Seattle, WA, USA

##### **Correspondence:** Lesley Steinman (lesles@uw.edu)

**Background**

The Program to Encourage Active, Rewarding Lives (PEARLS) was developed fifteen years ago when local social service agencies approached our Prevention Research Center to create a more accessible model for screening and treating minor depression in frail, homebound older adults. PEARLS is a brief, home-based collaborative care program that trains existing agency providers to teach older adults problem-solving and behavioral activation tools. The initial RCT [1] found that PEARLS significantly improved depression in low-income elders living with multiple chronic conditions. For ten years since, our research center has collaborated with community-based social service organizations and mental health agencies that reach underserved older adults to better understand how PEARLS is disseminated and implemented.

**Materials and Methods**

This presentation will describe lessons learned from implementation research and technical assistance activities to date using the following established implementation science frameworks and models: Powell and colleagues [2] implementation strategies, the Consolidated Framework for Implementation Research (CFIR) [3] and Normalisation Process Theory (NPT) [4,5] to understand facilitators and barriers to implementation outcomes, and Proctor et al.’s [6] taxonomy of implementation, service and client outcomes and RE-AIM [7] to evaluate the success of PEARLS implementation. Data include transcripts from interviews and focus groups, notes from technical assistance call discussions, self-report data from fidelity instruments, and PEARLS process and outcome data from PEARLS participants, providers, administrators, and referrers. We analyzed the data using descriptive statistics and content and thematic analysis.

**Results**

Key implementation strategies include accessing new funding, capturing and sharing local knowledge, centralizing technical assistance, changing service sites, conducting educational meetings, educational outreach visits, and ongoing training, developing academic partnerships, implementation tools, and educational materials, involve patients/consumers and obtain and use their feedback, making training dynamic, organize clinician implementation meetings, promote adaptability, provide clinical supervision, local technical assistance, and ongoing consultation, purposefully re-examine the implementation, revise professional roles, tailor implementation strategies, and using train-the-trainer strategies. Successful PEARLS implementation has been influenced by mechanisms of ‘coherence’ (sense-making work), ‘cognitive participation ‘(engagement work), ‘collective action’ (operational work) and ‘reflexive monitoring’ (feedback and quality improvement work). Main implementation outcomes include acceptability, appropriateness, costs, feasibility, and fidelity, patient-centered service outcomes, and participant satisfaction, function and other benefits in addition to improvements in depression.

**Conclusions**

This study begins to summarize what works with implementing PEARLS and identifies key gaps for further study through our community-academic partnership (e.g., utilizing existing implementation measures for implementation determinants and outcomes and moving beyond understanding what works to predicting what works for implementation).

## A84 Contextual tailoring of empirically-supported behavior therapies to augment combination antiretroviral therapy adherence: Perspectives from the HIV care community

### Bryan Hartzler^1^, Julia Dombrowski^2^, Dennis Donovan^1,3^

#### ^1^Alcohol & Drug Abuse Institute, University of Washington, Seattle, WA, USA; ^2^Division of Allergy and Infectious Disease, University of Washington, Seattle, WA, USA; ^3^Psychiatry and Behavioral Sciences, University of Washington, Seattle, WA, USA

##### **Correspondence:** Bryan Hartzler (hartzb@uw.edu)

**Background**

Substance use disorders (SUDs) are prevalent among HIV+ Americans [1], and challenge their adherence to combination antiretroviral therapy (cART). Multiple randomized controlled trials demonstrate efficacy in augmenting cART adherence among SUD patients for each of three behavior therapies: Cognitive-Behavioral Therapy (CBT) [2], Contingency Management (CM) [3], and Motivational Interviewing (MI) [4]. To inform broad dissemination efforts, community HIV care perspectives about the contextual compatibility of these behavior therapies may guide their tailored implementation.

**Materials and Methods**

In a mixed-method study, multilevel setting data were gathered from an executive, staff members, and patients during a full-day site visit to each of four regional health settings that principally offer HIV care services. Purposeful recruitment achieved an aim of setting diversity, with inclusion of a hospital-based HIV primary care clinic, urban outreach clinic, health center-affiliate virology clinic, and large group private practice. During the site visit, an executive initially participated in an ethnographic interview wherein organizational data were gathered and prospect of behavior therapy feasibility and clinical effectiveness was rated. After a brief facilities tour, a pair of respective focus groups were conducted with interested staff members (n=32) and patients (n=44) during which each of the three noted behavior therapies were discussed with eventual setting-therapy compatibility ratings elicited via live polls. Rating data were analyzed via generalized linear models, and focus group audio-recordings were subjected to a phenomenological narrative analysis by a multidisciplinary investigative pairing.

**Results**

Findings indicate: 1) cautious enthusiasm for these behavior therapies among setting executives that balanced strong perceived effectiveness with their SUD patients and moderate perceived feasibility due to setting-specific barriers; 2) much greater perceived setting-therapy compatibility for MI relative to CBT and CM among staff, albeit with some between-site variance in magnitude of their differential perceptions; 3) greater perceived setting- therapy compatibility for MI relative to CBT and CM among patients, albeit with substantial between-site variance in magnitude of their differential perceptions; 4) focal themes among staff valuing adaptability and patient- centeredness as therapy attributes as well as preservation of setting integrity; and 5) focal patient themes of preference for therapies that build intrinsic motivation, support patient autonomy, and maintain fairness among patients.

**Conclusions**

Collective findings of this community-participatory research effort highlight MI as a candidate behavior therapy for large-scale dissemination to HIV care settings to improve cART adherence among SUD patients. These community care perspectives also identify salient therapy attributes around which tailored implementation may focus.

**References**

1. Hartzler B, Dombrowski JC, Crane HM, Eron JJ, Geng EH, Christopher Mathews W, et al. Prevalence and predictors of substance use disorders among HIV care enrollees in the United States. AIDS and Behavior. 2017;21(4):1138-48.

2. Beck AT, Wright FD, Newman CF, Liese BS. Cognitive therapy of substance abuse. New York, NY: Guilford Press; 1993.

3. Higgins ST, Silverman K, Heil SH. Contingency management in substance abuse treatment. New York, NY: Guilford; 2008.

4. Miller WR, Rollnick S. Motivational Interviewing: helping people change. New York, NY: Guilford; 2013.

## A85 ‘We are the champions! Now what?’ Identifying mechanisms for long-term sustainability of evidence-based practices through an EBP champion program

### Casey Meinster, Amanda Gentz, Cameo Stanick

#### Hathaway-Sycamores Child and Family Services, Pasadena, CA, USA

##### **Correspondence:** Casey Meinster (caseymeinster@hathaway-sycamores.org)

**Background**

Research has identified barriers and facilitators to the sustainability of evidence-based practices (EBP) [1,2]. One mechanism for sustainability is the involvement of EBP ‘champions’ - individuals who are organizational thought leaders and influential on staff attitudes and decisions. Once champions are identified, a number of factors may serve as mechanisms for sustained EBP use. The current study describes the qualitative assessment of EBP champions on the mechanisms of sustained EBP use within a large, community-based youth mental health organization.

**Materials and Methods**

Eight individuals meeting established criteria were identified as possible EBP ‘Leads’ and recruited into the program, supporting 5 EBP protocols. These individuals completed an assessment twice within one year and 8 domains hypothesized as mechanisms of EBP sustainability were assessed: resources, change readiness, leadership engagement, staff-EBP compatibility, system dynamics (e.g., communication and collaboration within the organization), EBP protocol features, time and competing demands, and training. Champions were asked to indicate if items were barriers, facilitators, or both, and to provide comments regarding their choices.

**Results**

Across both assessment time points, features of all 8 mechanisms were identified as both positively and negatively impacting sustainability. At least 25% of champions indicated that each mechanism was a sustainability facilitator. ‘Change readiness’ and ‘leadership’ were identified as barriers to sustainability across both assessment time points. Utilizing the Consolidated Framework for Implementation Research (CFIR) coding guide, qualitative data were coded for themes to clarify the quantitative ratings [3]. For instance, the knowledge and beliefs about the innovation (CFIR subdomain) among staff were identified by EBP champions as relevant to sustaining the program, as staff were reportedly “open to learning the components and want to do it well.” Outer setting constructs, such as external policy and incentives, were reportedly negative influences on the ‘time and competing demands’ mechanism such that changes in funding and funding restrictions limited staff participation with EBPs relative to their caseload match.

**Conclusions**

EBP champions identification represents an important first step in EBP implementation. However, understanding the mechanisms for long-term sustainability through the assessment of EBP champions can close an important feedback loop. It is also possible that the mechanisms detected in the current study are relevant to sustaining the EBP champion program as well, given that champion utilization is dependent on a number of the same factors. Organizations considering implementing an EBP champion program may benefit from assessing relevant mechanisms a priori to support implementation.

**References**

1. Fixsen DL, Naoom SF, Blasé KA, Friedman RM, Wallace F. Implementation research: a synthesis of the literature. Tampa, FL: University of South Florida. 2005.

2. Blasinsky M, Goldman HH, Unutzer, J. Project IMPACT: A report on barriers and facilitators to sustainability. Adm Policy Ment Health Ment Health Serv Res. 2006;7:18.

3. Damschroder LJ, Aron DC, Keith RE, Kirsh SR, Alexander JA, Lowery JC: Fostering implementation of health services research findings into practice: a consolidated framework for advancing implementation science. Implement Sci. 2009;4:50.

## A86 Evaluating the impact of a tailored middle-manager-level facilitation intervention to improve implementation of evidence-based practices in community mental health

### Prerna Martin^1^, Rosemary Meza^1^, Lucy Berliner^2^, Sarah Birken^3^, Shannon Dorsey^1^

#### ^1^Department of Psychology, University of Washington, Seattle, WA, USA; ^2^Haborview Center for Sexual Assault and Traumatic Stress, Seattle, WA, USA; ^3^Department of Health Policy and Management, University of North Carolina Chapel Hill, Chapel Hill, NC, USA

##### **Correspondence:** Prerna Martin (prmartin@uw.edu)

**Background**

Research suggests that middle managers (e.g., supervisors) influence the implementation of innovations. The middle manager role theory suggests that supervisors do this through four roles: providing information about the innovation, making it relevant, providing necessary implementation tools, and encouraging consistent and effective innovation use [1]. These roles are hypothesized to positively impact implementation climate (proposed mechanism), which, in turn, influences implementation effectiveness. While some support exists for this model in implementing healthcare innovations [2,3], it has not been applied to a mental health context.

**Material and Methods**

The aims of this pilot study are to 1) evaluate the impact of a middle-manager-focused facilitation intervention in improving implementation climate, clinician implementation engagement, and quality of treatment delivery in the context of a state-funded CBT implementation initiative, and 2) examine acceptability and feasibility of the facilitation intervention. Supervisors from 17 public mental health agencies in WA State were randomized to receive either the facilitation intervention (N=12; supervising 29 clinicians) or control (N=12; supervising 34 clinicians). Intervention supervisors participated in 4 support calls before, during, and after implementation. Supervisors developed tailored work plans to fulfill the four middle manager roles to support clinicians’ CBT implementation. Clinician engagement in CBT delivery (e.g., use of online implementation tools, number of CBT cases during training) was measured through Toolkit, an online case tracking and training tool.

**Results**

Preliminary analyses of clinician engagement indicate that clinicians supervised by supervisors who received the facilitation intervention were more actively engaged in Toolkit during the first 50 days following training (M = 5.28, SD = 3.06) compared to controls (M = 3.21, SD = 3.13) t(60) = -2.62, p = .01. Clinicians in the intervention group also had a greater number of training cases (M = 5.03, SD = 4.44) than controls (M = 2.79, SD = 1.78), t(35.7) = -2.53, p = .02. Preliminary analyses also indicate that the facilitation intervention was acceptable (M=3.95) and feasible (M=3.83) to supervisors (N=4; range 1 [Not at all] to 5 [Extremely]). At study completion, we will examine these and other engagement indicators, quality of CBT delivery (e.g., measurement use, session-by-session component delivery) and change in implementation climate (proposed mechanism) across conditions.

**Conclusions**

The brief, tailored facilitation intervention appears acceptable and feasible to community supervisors and has a potentially beneficial impact on clinician engagement. Results will inform the development of an RCT examining the impact of middle manager roles on implementation climate and effectiveness.

**References**

1. Birken SA, Lee SY, Weiner BJ. Uncovering middle managers’ role in healthcare innovation implementation. Implement Sci*.* 2012;7(1):28.

2. Birken SA, Lee SY, Weiner BJ, Chin MH, Schaefer CT. Improving the effectiveness of health care innovation implementation: middle managers as change agents. Med Care Res Rev. 2013;70(1):29-45.

3. Birken SA, DiMartino LD, Kirk MA, Lee SY, McClelland M, Albert NM. Elaborating on theory with middle managers’ experience implementing healthcare innovations in practice. Implement Sci*.* 2016;11(1):2.

## A87 The effect of message board correspondence on therapist fidelity and adaptation in cognitive processing therapy for PTSD

### Clara Johnson^1^, Kera Mallard^1^, Patricia Carreño^1^, Matthew Beristianos^1^, Tasoula Masina^3^, Norman Shields^3^, Candice Monson^2^, Shannon Wiltsey-Stirman^1^

#### ^1^National Center for Posttraumatic Stress Disorder (PTSD), Veterans Affairs Palo Alto Health Care System & Stanford University, Menlo Park, CA, USA; ^2^Divisional Psychologist Occupational Health and Safety, Royal Canadian Mounted Police, Ottawa, Ontario, Canada; ^3^Ryerson University, Toronto, Ontario, Canada

##### **Correspondence:** Clara Johnson (Clara.Johnson@va.gov)

**Background**

Understanding the types of strategies that are necessary to support implementation and fidelity to evidence-based treatments (EBT) is essential to moving the field of implementation science forward. While learning collaboratives are being used more frequently to support implementation, there is little information available on their effectiveness, and on what aspects of learning collaboratives are essential [1]. Using a web-based learning collaborative (LC) format informed by the Institute for Healthcare Improvement’s Breakthrough Series Collaborative model [2], this study analyzed message board participation and content to examine how both clinicians and facilitators used this tool to improve therapist and patient outcomes.

**Materials and Methods**

A sample of 40 newly trained clinicians delivered Cognitive Processing Therapy (CPT), an EBT for Posttraumatic Stress Disorder (PTSD) to patients with a clinician-diagnosed PTSD. These clinicians were randomly assigned to one of two learning collaborative formats: fidelity-oriented (FID) or continuous quality improvement (CQI). Both conditions received consultation and support for delivering the CPT protocol; however, the CQI consultation leaders primarily used a plan-do-study act cycle (PDSA) to address barriers to using CPT protocol. Clinicians and facilitators in both conditions used an online message board correspondence tool to post important resources, questions, CPT worksheets and updated PDSA’s for the CQI condition.

**Results**

Preliminary results indicate that time spent engaging in correspondence (M =47.08 minutes; SD = 116.35) was associated at a trend-level with more fidelity consistent adaptation (t=1.75, ß=.33, p<.1;) and with higher confidence in delivering CPT (t=2.15, ß=.47, p<.05). We plan to run more detailed coding on message board content and relationships to therapist and patient outcomes.

**Conclusions**

The preliminary results point to a relationship between time spent corresponding and therapist outcomes; however, we expect to find more detailed results as we continue to analyze data from the active phase and the follow-up year. We plan to compare the effect of message board correspondence and learning collaborative condition on therapist fidelity and adaptation.

**References**

1. Nadeem E, Olin SS, Hill LC, Hoagwood KE, Horwitz SM. A literature review of learning collaboratives in mental health care: used but untested. Psychiatr Serv. 2014;(65)9:1088-99.

2. The Breakthrough Series: IHI’s collaborative model for achieving breakthrough improvement*.* Boston: Institute for Healthcare Improvement; 2003.

## A88 Implementation of evidence-based supervision in community mental health

### Leah Lucid, Adam M. Kuczynski, Katherine Benjamin, Shannon Dorsey

#### University of Washington, Seattle, WA, USA

##### **Correspondence:** Leah Lucid (llucid@uw.edu)

**Background**

Many efficacious evidence-based treatments (EBTs) have been developed to address child mental health needs. However, efforts to implement EBTs in community settings have often been unsuccessful. To date, implementation efforts have primarily focused on clinician-level training, sometimes with a limited period of outside expert consultation. In publicly funded settings, weekly supervision is “nearly ubiquitous” [1], yet community-based supervision is one of the least studied implementation factors [2]. A supervisor’s expertise in treatment [3] and an organization’s EBT implementation climate—defined as perceptions of the extent to which use of EBTs is rewarded, supported, and expected—may increase clinician treatment fidelity and improve client outcomes [4]. However, it is unclear how supervisor expertise and a supportive climate translate into higher clinician fidelity and better client outcomes. We hypothesize that community-based supervisors vary in their focus on EBTs in supervision, and that implementation climate and individual supervisor factors may predict this variation.

**Materials and Methods**

The present study tested whether supervisor- and organization-level factors predicted evidence-based supervision content in objectively coded audio recordings of Trauma-Focused Cognitive Behavioral Therapy (TF-CBT) supervision. Participants included supervisors (n = 28) and clinicians (n = 70) from 20 community mental health clinics across Washington State participating in an NIH-funded supervision study. Self-report surveys assessed background characteristics, self-efficacy supervising TF-CBT, knowledge of TF-CBT, and EBT implementation climate. Our main outcome was objectively coded supervision coverage of clinically challenging TF-CBT content using an adaptation of the Therapeutic Process Observational Coding System for Child Psychotherapy (Supervision TPOCS) [5]. In this study, we use multilevel modeling to predict how extensively supervisors addressed three underutilized [6] yet important TF-CBT content areas during supervision: parenting skills; trauma narrative (TN) and gradual exposure; and conjoint session preparation.

**Results**

Implementation climate was the strongest predictor of how extensively supervisors covered parenting skills and TN/ exposure. As implementation climate increased, so did parenting skills (b = 0.20, t(25) = 2.23, p = .035) and TN/ exposure (b = 2.28, t(18) = 2.94, p = .009), but not conjoint session preparation (b = 0.07, t(25) = 0.68, p = .501). Supervisor characteristics such as TF-CBT knowledge and supervision self-efficacy also differentially predicted supervisor engagement in these important TF-CBT content areas, but no individual supervisor characteristic explained the supervision content as strongly as implementation climate.

**Conclusions**

Our findings suggest that although individual supervisor factors matter for predicting TF-CBT content coverage in supervision, increasing a clinic’s implementation climate to further support EBTs may be the most critical for improving supervision coverage.

**References**

1. Schoenwald SK, Mehta TG, Frazier SL, Shernoff ES. Clinical supervision in effectiveness and implementation research. Clin Psychol. 2013:20(1):44-59.

2. Accurso EC, Taylor RM, Garland AF. Evidence-based practices addressed in community-based children’s mental health clinical supervision. Train Educ Prof Psychol. 2011;5(2):88.

3. Schoenwald, SK, Sheidow AJ, Chapman JE. Clinical supervision in treatment transport: effects on adherence and outcomes. J Consult Clin Psychol. 2009;77(3):410.

4. Aarons GA, Sawitzky AC. Organizational culture and climate and mental health provider attitudes toward evidence-based practice. Psychol Serv. 2006:3(1):61.

5. McLeod BD, Weisz JR. The therapy process observational coding system for child psychotherapy strategies scale. J Clin Child Adolesc Psychol. 2010;39(3: 436-43.

6. Dorsey S, Pullmann MD, Kerns SEU, Jungbluth N, Meza R, Thompson K, Berliner L. The juggling act of supervision in community mental health: implications for supporting evidence-based treatment*.* 2017;44(6):838-52.

## A89 Tracking implementation strategies in a community mental health implementation initiative

### Meredith Boyd^1^, Byron Powell^2^, Cara Lewis^1,3^

#### ^1^Psychological and Brain Sciences, Indiana University, Bloomington, IN, USA; ^2^Department of Health Policy and Management, Gillings School of Global Public Health, University of North Carolina at Chapel Hill, Chapel Hill, NC, USA; ^3^Kaiser Permanente Washington Health Research Institute, Seattle, WA, USA

##### **Correspondence:** Meredith Boyd (mereboyd@indiana.edu)

**Background**

Implementation experts suggest tailoring implementation strategies to the intended context may enhance implementation outcomes [1]. However, it remains unclear which strategies are best suited to address specific barriers to implementation. While there is also mounting evidence for directly involving key stakeholders in implementation [2], it is unknown which strategies these groups are likely to select in the course of an implementation effort and why, an important step in identifying candidate mechanisms of implementation. The present study addresses these gaps by: 1) comparing implementation strategies utilized by six community mental health clinics working to implement measurement-based care (MBC), specifically use of the Patient Health Questionnaire Nine Items (PHQ-9; [3]), 2) examining the relationship between strategy use and implementation outcomes, and 3) exploring stakeholder justification of strategy use to identify potential mechanisms of implementation.

**Materials and Methods**

A coding form based on Proctor et al.’s implementation strategy reporting guidelines was created to facilitate specification of the strategies used [4]. A trained research assistant coded digitally recorded implementation team (IT) meetings. Strategies were described using language of meeting members, and later coded using standardized language from a published taxonomy of implementation strategies [5]. Concurrently, data was collected via the electronic health record (EHR) regarding clinician use of the PHQ-9.

**Results**

Videos of IT meetings were collected for all clinics with an average of six meetings per clinic. Strategy use was coded for two clinics. Clinic 1 opted to distribute the PHQ-9 to clients in the lobby to increase the likelihood that clinicians would use MBC. However, because no alert was built into the EHR to identify eligible clients for survey administration, 22% of strategies planned or enacted focused identifying clients and distributing surveys. Clinic 2 also decided to distribute surveys to clients in the lobby. The majority of strategies enacted or planned in the first two meetings centered on this aim. Two months into active implementation, the organization’s analytics department added an alert into the EHR that identified clients eligible for survey administration. Subsequent meetings focused on a more diverse range of strategies targeting clinician buy-in for MBC and incorporation of MBC discussion when staffing clinical cases.

**Conclusions**

Preliminary results emphasize the importance of infrastructure in the implementation of MBC. This study will also link detailed reports of strategy use to implementation outcomes, a critical step in establishing evidence for use of specific strategies.

**References**

1. Wensing M, Oxman A, Baker R, Godycki-Cwirko M, Flottorp S, Szecsenyi J, Grimshaw J, Eccles M. Tailored implementation for chronic diseases (TICD): a project protocol. Implement Sci. 2011;6(1):103.

2. Higgins MC, Weiner J, Young L. Implementation teams: a new lever for organizational change. J Organ Behav. 2012;33(3):366-88.

3. Kroenke K, Spitzer RL, Williams JB. The Phq 9. J Gen Intern Med. 2001;16(9):606-13.

4. Proctor EK, Powell BJ, McMillen JC. Implementation strategies: recommendations for specifying and reporting. Implement Sci. 2013;8(1):139.

5. Powell, BJ, Waltz, TJ, Chinman, MJ, Damschroder, LJ, Smith, JL, Matthieu, MM, Proctor, EK, Kirchner, JE. A refined compilation of implementation strategies: results from the Expert Recommendations for Implementing Change (ERIC) project. Implement Sci. 2015;10(1):21.

## A90 Adaptation in dissemination and implementation science

### Ana A. Baumann^1^, Leopoldo J. Cabassa^1^, Shannon Wiltsey-Stirman^2^

#### ^1^George Warren Brown School of Social Work, Washington University in St. Louis, St. Louis, MO, USA; ^2^National Center for PTSD and Stanford University, Menlo Park, CA, USA

##### **Correspondence:** Ana A. Baumann (abaumann@gwbmail.wustl.edu)

**Background**

Despite advances in research methods in the field of dissemination and implementation (D&I), we have not yet been able to answer the decades-old question of what works best for whom under what circumstances [1]. Investigators are still calling for increased action in promoting evidence-based interventions in usual care and for testing interventions and designs to optimize outcomes [2,3]. In light of the diversity of patient populations, providers, and service settings into which interventions are delivered, it is unlikely that the same program, techniques and strategies can be implemented successfully in the exact same way across multiple contexts. Scholars from the fields of implementation science and cultural adaptation warn of the dangers of implementing evidence-based interventions without attending to the fit of the interventions to the context, in particular to the populations that are being served, the different providers who deliver these interventions, and the diversity of service settings who could benefit from these interventions [4,5]. In fact, numerous studies indicate the importance of matching the intervention with the population and context of interest, including attention to race, ethnicity, location, community norms, service settings and organizational characteristics [4,6,7].

**Materials and Methods**

Drawing from the cultural adaptation field and recent advances in D&I science, we propose that scholars should carefully consider evaluating, documenting, and rigorously studying the adaptation process and outcomes.

**Results**

Using Stirman et al’s framework [8] as a starting point, we provide a broader conceptualization of adaptations. Our assumption is that by clearly specifying and evaluating adaptation, we can increase the external validity of the intervention, the implementation strategies, its outcomes, and the implementation process. This is a conceptual presentation where we: (a) outline why D&I science scholars should consider adaptation, (b) describe when to adapt intervention, followed by outlining components scholars should consider adapting, how to adapt components, how to evaluate the impact of adaptation, and (c) provide our recommendations for the D&I science field regarding adaptation of interventions.

**Conclusions**

Consistent with the existing literature, we recommend that adaptations be proactively and iteratively determined, strongly informed by a variety of stakeholders, that efforts be made to carefully describe and document the nature of the adaptations as well as to evaluate their impact on desired service, health, and implementation outcomes.

**References**

1. Paul GL. Strategy of outcome research in psychotherapy. J Consult Psychol. 1967;31:109.

2. National Institutes of Mental Health. The National Institute of Mental Health strategic plan. 2015. https://www.nimh.nih.gov/about/strategic-planning-reports/index.shtml

3. National Research Council and Institute of Medicine. Preventing mental, emotional, and behavioral disorders among young people: progress and possibilities. O'Connell ME, Boat T, Warner KE, editors. Washington DC: The National Academies Press; 2009.

4. Bernal G, Domenech Rodriguez MM. Cultural adaptation in context: psychotherapy as a historical account of adaptations. In Bernal G, Domenech Rodriguez MM, editors. Cultural adaptations: tools for evidence-based practice with diverse populations. Washington DC: American Psychological Society; 2012. p. 3-22.

5. Cabassa LJ, Baumann AA. A two-way street: bridging implementation science and cultural adaptations of mental health treatments. Implement Sci. 2013;8:90.

6. Aarons GA, Miller EA, Green AE, Perrott JA. Bradway R. Adaptation happens: a qualitative case study of implementation of The Incredible Years evidence-based parent training programme in a residential substance abuse treatment. J Child Serv. 2012;4:233-45.

7. Graham PW, Kim MM, Clinton-Sherrod AM, Yaros A, Richmond AN, Jackson M, Corbie-Smith G. What is the role of culture, diversity, and community engagement in transdisciplinary translational science? Transl Behav Med. 2016;6(1):155-24. doi:10.1007/s13142-015-0368-2

8. Stirman SW, Miller CJ, Toder K, Calloway A. Development of a framework and coding system for modifications and adaptations of evidence-based interventions. Implement Sci. 2013*;*8:65.

## A91 Mixed methods model for evaluating shared decision making implementations

### Ann Nguyen^1^, Cynthia LeRouge^1^, Deborah Bowen^1^, Melissa Schiff^2,3^, Megan Rogers^4^, Savitha Sangameswaran^5^, Tao Kwan-Gett^1^

#### ^1^Department of Health Services, School of Public Health, University of Washington, Seattle, WA, USA; ^2^Department of Epidemiology, School of Medicine, University of Washington, Seattle, WA, USA; ^3^Department of Obstetrics and Gynecology, School of Medicine, University of Washington, Seattle, WA, USA; ^4^Northwest Center for Public Health Practice, University of Washington, Seattle, WA, USA; ^5^Department of Biomedical Informatics and Medical Education, School of Medicine, University of Washington, Seattle, WA, USA

##### **Correspondence:** Ann Nguyen (annn4@uw.edu)

**Background**

Shared decision making (SDM) is a process in which patients and their care team work together to make decisions informed by scientific evidence as well as patients’ values and preferences. SDM implementations, however, are complex due to the inherent problems of a causal narrative colluded by multiple changes at multiple levels. The literature also points to significant gaps in SDM measurement. Our objective is to share a mixed methods model for evaluating SDM implementations, identifying the components and challenges for evaluation and how to address them. Our model was developed for the Washington State Health Care Authority (HCA) for implementation of a certified patient decision aid (PDA) to support SDM. Washington is the first state to certify PDAs.

**Materials and Methods**

We are using a mixed methods approach to examine implementation in two parts – process and impact – on a maternal health decision, the type of delivery after prior cesarean. We developed a model based on the CMS SDM evaluation framework created for the Health Care Innovations Awardees [1], which we further extended by integrating three implementation science frameworks: Damschroder (Consolidated Framework of Implementation Research), Greenhalgh (Diffusion of Innovations Model), and Aarons (Evidence-Based Practice Implementation Model) [2-4]. This integrative model guided our measures and study design to include: interviews with HCA, vendors, and pilot sites; direct observation of implementation activities; content analysis of SDM tools and documents; pre- and post-implementation surveys of providers and patients; interviews with providers and patients; direct observation of patient workflow; and review of electronic medical record (EMR) data. We are studying three organizations: HCA, vendors, and pilot sites. The three sites recruited are Washington health systems.

**Results**

SDM implementation requires early and frequent communication between stakeholders, with success more likely when there is physician buy-in, a team-based approach, and vendor-provided training and support. Considerations and challenges to SDM evaluation include: defining the intervention (tool, change of process, documentation in EMR) and capturing the aspects of the SDM tool (content, presentation, interaction, implementation process, workflow, role of certification, end objective, defining measures for SDM, and need for multiple perspectives).

**Conclusions**

A multilevel conceptual framework and mixed methods approaches are required to capture the complexity and heterogeneity of SDM implementations. Interviews and observations capture the narrative of the patient workflow and complement survey and EMR data. Evaluation thus requires design under real-world conditions, which in turn requires an integration of evidence-based approaches.

**References**

1. Deleire T, Wyman J, Haron-feiertag R, Olinger L. Evaluation of the Shared Decision Making (SDM) & medication Management (MM) health care innovation awardees annual report 1. CMS. 2014. Retrieved from https://innovation.cms.gov/Files/reports/HCIA-SDM-FirstEvalRpt.pdf

2. Damschroder LJ, Aron DC, Keith RE, Kirsh SR, Alexander JA, Lowery JC. Fostering implementation of health services research findings into practice: a consolidated framework for advancing implementation science. Implement Sci. 2009;4(1):50.

3. Greenhalgh T, Robert G, Macfarlane F, Bate P, Kyriakidou O. Diffusion of innovations in service organizations: systematic review and recommendations. Milbank Q. 2004;82(4):581-629.

4. Aarons GA, Hurlburt M, Horwitz SM. Advancing a conceptual model of evidence-based practice implementation in public service sectors. Adm Policy Ment Health. 2011;38(1):4-23.

## A92 Tailoring the diabetes prevention program for women veterans: Use of replicating effective programs (REP) to facilitate and evaluate adaptation in VA primary care

### Tannaz Moin^1,2,3^, Bevanne Bean-Mayberry^1,2,3^, Jessica Zuchowski^1,2^, Melissa Farmer^1,2^, Erin Finley^4^, Alison Hamilton^1,2,3^

#### ^1^VA Greater Los Angeles Health System, Los Angeles, CA, USA; ^2^HSR&D Center for the Study of Healthcare Innovation, Implementation & Policy, Los Angeles, CA, USA; ^3^David Geffen School of Medicine at UCLA, Los Angeles, CA, USA; ^4^South Texas Veterans Health Care System and UT Health Science Center, San Antonio, San Antonio, TX, USA

##### **Correspondence:** Tannaz Moin (tmoin@mednet.ucla.edu)

**Background**

Despite increasing calls to tailor evidence-based practices to meet the needs of specific populations or settings, tailoring continues to pose challenges related to adaptation, implementation, and evaluation. The Replicating Effective Programs (REP) framework was developed to facilitate tailoring in low-resource settings and incorporates (a) stakeholder engagement, (b) adaptation of both intervention and implementation strategies, (c) evaluation of implementation and effectiveness outcomes, and (d) planning for sustainability and spread. We conducted a VA QUERI-funded one-year quality improvement project using REP to inform tailoring and implementation of the evidence-based Diabetes Prevention Program (DPP) to meet the needs of women Veterans in women’s VA primary care (PC) settings.

**Materials and Methods**

Based on pre-implementation stakeholder feedback, DPP was tailored in two primary ways: (1) by offering gender- specific groups for women Veterans, who have expressed discomfort with participating in mixed-gender groups; and (2) by offering participants a choice between peer-led in-person or online versions of the intervention. Of 863 women Veterans screened for DPP eligibility, 515 were contacted to provide education and outreach regarding DPP. Patient and implementation outcomes were assessed using contact tracking, patient and provider semi- structured interviews at baseline and six-month follow-up, a patient survey at baseline and follow-up, and monthly reflection forms completed by the PI and project team to document ongoing activities, adaptations, and stakeholder input.

**Results**

Among 281 women Veterans reached by phone, 191 (68%) expressed interest; 48 chose the peer-led (in-person) DPP intervention, 73 chose the online DPP format, and 51 declined participation. Significant patient demand for the program resulted in expansion to serve 120 women rather than the 40 planned. Interviews conducted during early implementation indicated that most women were unaware of their prediabetes status and women appreciated having gender-specific groups and a choice of in-person or online format. Women Veterans reported high satisfaction with DPP content in both formats. Mean weight loss indicated greatest benefit for those attending >4 sessions.

**Conclusions**

Following the REP framework throughout this one-year quality improvement study resulted in delivery of a tailored DPP intervention designed to meet the needs of women Veterans and to be feasible for delivery in VA PC settings. Program satisfaction was high and program reach exceeded expectations. These findings suggest REP has utility in real-world efforts to achieve active implementation of tailored interventions.

## A93 Engaging multilevel stakeholders in an implementation trial of evidence-based quality improvement in VA women’s health primary care

### Alison Hamilton^1^, Julian Brunner^2^, Cindy Cain^2^, Emmeline Chuang^3^, Tana Luger^2^, Ismelda Canelo^4^, Lisa Rubenstein^4^, Elizabeth Yano^4^

#### ^1^Department of Veterans Affairs, Los Angeles, CA, USA; ^2^University of California, Los Angeles, Los Angeles, CA, USA; ^3^University of California, Los Angeles, Fielding School of Public Health, Los Angeles, CA, USA; ^4^VA Greater Los Angeles Healthcare System, Los Angeles, CA, USA

##### **Correspondence:** Alison Hamilton (alisonh@ucla.edu)

**Background**

The Veterans Health Administration (VHA) has undertaken primary care transformation based on patient-centered medical home (PCMH) tenets. VHA PCMH models are designed for the predominantly male Veteran population, and require tailoring to meet women Veterans’ needs. We used evidence-based quality improvement (EBQI), a stakeholder-driven implementation strategy, in a cluster randomized controlled trial across 12 sites (eight EBQI, four control) that are members of a Practice-Based Research Network. EBQI involves engaging multi-level, inter- professional leaders and staff as stakeholders in reviewing evidence and setting QI priorities.

**Materials and Methods**

Four inter-professional regional stakeholder planning meetings were conducted; these meetings engaged stakeholders by providing regional data about gender disparities in Veterans’ care experiences. Subsequent to each meeting, qualitative interviews were conducted with 87 key stakeholders (leaders and staff). Stakeholders were asked to describe QI efforts and the use of data to change aspects of care, including women’s health care. Interview transcripts were summarized and coded using a hybrid deductive/inductive analytic approach.

**Results**

The presentation of regional-level data about gender disparities resulted in heightened awareness and stakeholder buy-in and decision-making related to women’s health-focused QI. Interviews revealed that stakeholders were familiar with QI, with regional and facility leaders aware of interdisciplinary committees and efforts to foster organizational change, including PCMH transformation. These efforts did not typically focus on women’s health, though some informal efforts had been undertaken. Barriers to engaging in QI included lack of communication across clinical service lines, fluidity in staffing, and lack of protected time.

**Conclusions**

Inter-professional, multi-level stakeholders need to be engaged in implementation early, with data and discussion that convey the importance and relevance of a new initiative. Stakeholder perspectives on institutional norms (e.g., gender norms) and readiness for population-specific QI are useful drivers of clinical initiatives designed to transform care for clinical subpopulations.

## A94 Foreseeing the future: Measures’ predictive validity of implementation outcomes

### Kayne Mettert^1^, Caitlin Dorsey^1^, Cara Lewis^1^, Elspeth Nolen^2^, Bryan Weiner^2^

#### ^**1**^Kaiser Permanente Washington Health Research Institute, Seattle, WA, USA; ^2^University of Washington, Seattle, WA, USA

##### **Correspondence:** Kayne Mettert (mettert.k@ghc.org)

**Background**

Relatively new constructs and outcomes of implementation require reliable and valid measurement. Previous research has demonstrated that measures of implementation outcomes are generally substandard or have unknown psychometric properties [6]. Furthermore, while establishing the predictive validity of measures is pivotal to understanding which strategies effectively support the implementation of evidence-based practices [1], recent studies indicate few measures have established predictive validity [1,2,4]. Moreover, previous research has not specified predictive validity as it pertained to one of eight implementation outcomes [7]. Implementation mechanisms cannot be identified until measures’ predictive validity is established [5]. The current study endeavored to address the aforementioned knowledge gaps by 1) assessing the psychometric quality of measures of readiness for implementation as delineated in the Consolidated Framework for Implementation Research (CFIR; [3]) and, 2) establishing the ability of readiness measures to predict specific implementation outcomes.

**Materials and Methods**

We conducted a systematic review to identify measures for assessment. First, we searched PubMed and Embase databases in order to identify literature with CFIR- relevant measures published between 1985-2017. Studies were included if they were written in English, contained quantitative measurement (e.g. survey, questionnaire), they involved an evidence-based innovation, they assessed readiness for implementation, and if they pertained to behavioral health. Once identified, studies were compiled into PDF ‘packets’ and relevant information was extracted for a formalized rating process. Two independent raters applied revised Evidence-Based Assessment criteria [6], which contains standards for internal consistency, structural validity, discriminant validity, convergent validity, known-groups validity, concurrent validity, predictive validity, norms, and responsiveness. The predictive validity rating criterion was modified so that it allowed for characterization of measures’ predictive validity relating to implementation outcomes.

**Results**

Simple statistics (i.e., frequencies) pertaining to the psychometric quality and predictive validity of readiness measures are presented. Preliminary results suggest that most measures of readiness are used only once and that many have substandard reliability and validity. We present high quality measures that are recommended for use, in addition to low quality measures that require further development or overall abandonment. We also highlight measures with established predictive validity and indicate which outcome they predict.

**Conclusions**

Ratings will allow researchers to carefully select valid measures with established predictive, positioning them to pinpoint moderators, mediators, and mechanisms of implementation with confidence.

**References**

1. Chaudoir SR, Dugan AG, Barr CH. Measuring factors affecting implementation of health innovations: a systematic review of structural, organizational, provider, patient, and innovation level measures. Implement Sci. 2013;8:22. doi:10.1186/1748-5908-8-22

2. Chor KH, Wisdom JP, Olin SC, Hoagwood KE, Horwitz SM. Measures for predictors of innovation adoption. Adm Policy Ment Health. 2015;42(5):545-73. doi:10.1007/s10488-014-0551-7

3. Damschroder LJ, Aron DC, Keith RE, Kirsh SR, Alexander JA, Lowery JC. Fostering implementation of health services research findings into practice: a consolidated framework for advancing implementation science. Implement Sci. 2009;4(1):50.

4. Durlak J, DuPre E. Implementation matters: a review of research on the influence of implementation on program outcomes and the factors affecting implementation. Am J Community Psychol. 2008;41(3-4):327-50.

5. Kazdin AE. Mediators and mechanisms of change in psychotherapy research. Annu Rev Clin Psychol. 2007;3:1-27. doi:10.1146/annurev.clinpsy.3.022806.091432

6. Lewis, C. C., Fischer, S., Weiner, B. J., Stanick, C., Kim, M., & Martinez, R. G. Outcomes for implementation science: an enhanced systematic review of instruments using evidence-based rating criteria. Implement Sci. 2015;10:155. doi:10.1186/s13012-015-0342-x

7. Proctor, E., Silmere, H., Raghavan, R., Hovmand, P., Aarons, G., Bunger, A., et al. Outcomes for implementation research: conceptual distinctions, measurement challenges, and research agenda. Adm Policy Ment Health. 2011;38(2):65-76. doi:10.1007/s10488-010-0319-7

## A95 Reducing inappropriate use of inhaled corticosteroids among patients with mild-to-moderate COPD: Baseline survey of providers participating in a de-implementation quality improvement project

### Christian Helfrich^1,2^, Renda Wiener^3,4^, Seppo Rinne^3,4,5^, Edmunds Udris^1^, Colby Lea^1^, Barbara Majerczyk^1^, Laura Feemster^1,6^, David Au^1,6^

#### ^1^Seattle-Denver Center of Innovation for Veteran-Centered & Value-Driven Care, VA Puget Sound Health Care System, Seattle, WA, USA; ^2^Department of Health Services, University of Washington School of Public Health, Seattle, WA, USA; ^3^The Pulmonary Center, Boston University School of Medicine, Boston, MA, USA; ^4^Center for Healthcare Organization & Implementation Research, Bedford VA Medical Center, Bedford, MA, USA; ^5^Pulmonary Critical Care, Yale University, New Haven, CT, USA; ^6^Division of Pulmonary and Critical Care, University of Washington, Seattle, WA, USA

##### **Correspondence:** Christian Helfrich (christian.helfrich@va.gov)

**Background**

Patients with mild to moderate chronic obstructive pulmonary disease (COPD) are commonly prescribed inhaled corticosteroid (ICS), in spite an increased risk of pneumonia and the availability of equally effective, safer long- acting muscarinic agonists (LAMAs) and long-acting beta agonists (LABAs). Overuse of ICS might arise from prescribing providers conflating treatment for COPD and treatment for asthma; lack of awareness of harms from ICS or availability of alternatives. Implementation models suggest workplace climate may play a role, particularly related to support for improving patient care. However, little is known about the prevalence of these views among prescribing providers or their receptiveness to changing prescribing of ICS.

**Materials and Methods**

As part of a quality improvement project on medical overuse, we conducted surveys with primary care providers at 13 primary care clinics affiliated with two VA medical centers between July and August 2016 (Bedford VA Medical Center) and December 2016 and January 2017 (VA Puget Sound Health Care System).

**Results**

Among 134 eligible providers surveyed, 46 responded (34% response rate). Recent prescribing and awareness of guidelines: 64% reported they prescribed an ICS for one or more primary care patients with mild to moderate COPD in the prior month. 46% were unaware that ICS were associated with a higher risk of pneumonia, and 52% were unaware that LAMAs/LABAs are as effective as ICS in reducing breathing exacerbations. 41% reported that they were unlikely to take patients off of an ICS prescription that another provider prescribed.

Workplace climate: 78% reported frequently observing colleagues exhibit a sense of personal responsibility for improving patient care and outcomes, but only 15% reported that they and their colleagues frequently had the necessary resources such as budget, training, or protected time when a change needs to happen to improve patient care. 46% reported that clinical innovation and creativity to improve patient care is rewarded infrequently. 35% screened positive for burnout and 24% reported they would leave their current job if they were able. Intention to change prescribing practices in the next 6 months: 50% reported they would make an effort to make greater use of long acting agents and 52% would make an effort to reduce the use of inhaled corticosteroids.

**Conclusions**

Half of PCPs were unaware of the most recent data on use of ICS for mild-moderate COPD, but when presented with information, are committed to improving their prescribing practices despite many feeling unsupported in their work environment.

## A96 Implementation strategies used by state mental health agencies to promote compliance with federal behavioral health parity law

### Jonathan Purtle^1^, Ben Borchers^1^

#### ^**1**^Drexel University, Dornsife School of Public Health, Philadelphia, PA, USA

##### **Correspondence:** Jonathan Purtle (jpp46@drexel.edu)

**Background**

First implemented in 2010, the federal Mental Health Parity and Addiction Equity Act (MHPAEA) was enacted to eliminate disparities in insurance coverage between behavioral and physical health services. State mental health agencies have been identified as potentially important to MHPHAEA implementation, but little empirical research has examined MHPAEA implementation strategies. More broadly, public policy-focused research is an underdeveloped area in the field of implementation science. The study aims were to: 1) determine the proportion of state mental health agencies involved with MHPAEA implementation between 2010 and 2015, 2) characterize the implementation strategies used by these agencies, and 3) assess the utility of the Expert Recommendation for Implementing Change (ERIC) compilation to state mental health agencies and MHPAEA implementation.

**Materials and Methods**

Data collected through the State Mental Health and Substance Abuse Profiling System surveys were used to assess state mental health agency involvement in MHPAEA implementation in 2010, 2012, and 2015. ERIC category definitions were revised through an iterative process to capture agency responses. Directed content analysis was then used to code open-ended responses about MHPAEA implementation strategies to revised ERIC categories. Univariate statistics were generated to describe the proportion state mental health agencies using each implementation strategy and examine trends in implementation between 2010 and 2015.

**Results**

In 2010, 28 (54.9%) state mental health agencies expected to be involved with MHPAEA implementation, but only 12 (23.5%) were involved in 2012 and only six (11.8%) were involved in 2015. Forty-one implementations strategies were identified that fit within six ERIC categories. Ongoing consultation was the most common implementation strategy, accounting for 24 (58.5%) of strategies reported, followed by local technical assistance, accounting for six (14.6%) strategies. Six ERIC compilation strategies were relevant to the MHPAEA activities reported by state mental health agencies. Minor revisions were made to ERIC definitions across the domains of specificity about the implementation actor (i.e., state mental health agencies), action (i.e., related to MHPAEA implementation), and action target (e.g., providing support to state insurance agencies).

**Conclusions**

State mental health agency involvement with MHPAEA implementation has been limited. When MHPAEA was first implemented in 2010, many agencies expected to provide consultation or technical assistance to assist with implementation. However, few agencies went on to actually perform these activities in 2012 or 2015. Future research should explored barriers and facilitators to these activities. The ERIC compilation has utility as a resource for public policy-focused implementation research.

## A97 Using coaching to implement evidence-based mental health practices in schools: Effectiveness and feasibility evidence from the TRAILS program

### Elizabeth S. Koschmann^1^, James L. Abelson^1^, Shawna N. Smith^1,3,4^, Kate Fitzgerald^1^, Anna Pasternak^1^, Amy M. Kilbourne^1,2^

#### ^1^University of Michigan, Department of Psychiatry, Ann Arbor, MI, USA; ^2^VA Center for Clinical Management Research, Ann Arbor, MI, USA; ^3^University of Michigan, Institute for Social Research, Ann Arbor, MI, USA; ^4^University of Michigan, Department of Internal Medicine, Ann Arbor, MI, USA

##### **Correspondence:** Elizabeth S. Koschmann (shawnana@umich.edu)

**Background**

With 20-30% of school age children affected by mood and anxiety disorders, schools provide an ideal venue for improving access to evidence-based mental health practices (EBPs). In particular, training existing school professionals (SPs) to deliver mental health EBPs in the context of available student support services could substantially improve access. However, EBP training opportunities for SPs are often unaffordable and, more importantly, lack the follow-up supported practice necessary for ensuring effective EBP implementation. Coaching, an implementation strategy that provides in-person, post-training support and live practice with an expert, holds promise for improving the uptake and sustainability of EBPs among SPs across diverse school settings.

**Materials and Methods**

In this pilot hybrid implementation-effectiveness study, we examined the feasibility and effectiveness of a novel coaching-based implementation strategy for integrating common elements of evidence-based Cognitive Behavioral Therapy (CBT) into 24 diverse public school settings. The implementation strategy incorporated didactic training in CBT for SPs (N=53) followed by live coaching from a treatment expert during co-facilitation of CBT skills groups offered to students (n=293) during school hours for 12-16 weeks. Feasibility was evaluated via success in recruiting and coaching SPs, and retaining students in CBT groups. Effectiveness was assessed using mixed-effects models to assess over-time changes in SP confidence delivering CBT, frequency of CBT skill utilization, and perceptions of CBT utility for the school setting, as well as student symptom improvement.

**Results**

Fifty-three SPs from 24 public schools with significant cultural and socioeconomic diversity were recruited to participate in coaching. All 53 SPs participated in training and 49 (92%) completed the full course of coaching. Over the course of the combined training and coaching components, SPs saw significant improvements in CBT confidence (Bsy=1.27; p<0.001), utilization (Bsy=0.86; *p*<0.001), and attitudes towards CBT (Bsy=0.75; *p*<0.001). For student participants, average PHQ-9-measured depression decreased from 10.1 prior to CBT group participation to 7.7 at group end (p<0.001); and GAD-7 measured anxiety declined from 9.1 to 7.1 (p<0.001).

**Conclusions**

Delivery of EBPs in novel settings, including schools, provides a compelling means of increasing access and practice effectiveness, but requires development, deployment, and assessment of novel implementation strategies. Coaching resulted in significant improvement in broadly-defined SP ability to deliver CBT in schools, leading to improved student mental health outcomes. These findings reinforce the value of school-delivered CBT for depression and anxiety and suggest that the coaching implementation strategy is a promising means of diffusing EBPs into a central community setting.

## A98 A public-health approach to integrating a parenting intervention and referral system in primary care

### Cathea Carey^1^, Suzanne Kerns^2^

#### ^**1**^University of Washington, Seattle, WA, USA; ^2^University of Denver, Denver, CO, USA

##### **Correspondence:** Cathea Carey (cmc37@uw.edu)

**Background**

In recent years, the relative importance of mental health in supporting overall health has gained more widespread acceptance in the medical community [1]. In particular, supporting the role of parents has been shown to have far reaching benefits for the entire family and, importantly, great potential in shifting the developmental trajectories associated with adverse childhood experiences [2]. There is a developing literature supporting systematic approaches to providing that support within the context of primary care (PC) [3], though much remains to be learned [4, 5]. Policy- level, organizational-level, and practitioner level considerations are paramount [6]. In this poster, we explicate the primary factors that impacted delivery of a brief parenting intervention and referral system in primary care settings.

**Materials and Methods**

24 primary care physicians (PCPs) received training in the evidence-based Triple P Positive Parenting Program – Brief Primary Care intervention and 21 (88%) became accredited to provide the service. This intervention involves supporting families in one 10-30 minute session when they identify a child behavior problem or parenting need. PCPs use a tailored tip-sheet to come up with a plan to address the concern. A referral system through the public health department was implemented to support PCPs when parenting needs were unable to be addressed within this brief session.

PCPs completed surveys across three time points, baseline (n=24), at training accreditation (n=21), and at 6-months following training (n=10). Attitudes towards EBPs, self-efficacy, preparedness to deliver the intervention, and confidence in parent consultation skills were collected as independent variables. PCP responses about the behavioral health referral process, cross-agency community collaboration, and knowledge of community resources were dependent variables.

**Results**

As a group, PCPs indicated favorable attitudes towards evidence-based psychosocial interventions in general. PCPs showed continued improvement in self-efficacy, preparedness, and confidence in parent consultation skills. At the 6 month follow up, those responding (n=10) indicated that their perceptions of the behavioral health referral process, cross agency communication, and knowledge of community resources decreased over time. At the time of the 6 month follow-up, 30% of PCPs reporting using the intervention. While ratings of the relevance and applicability of the intervention were high, PCPs ran into substantial organizational-level barriers to implementation at the organizational and infrastructural level. Qualitative findings suggest more is needed in adapting the model to clinic setting.

**Conclusions**

Despite favorable intervention and skills ratings provided by PCPs, rates of implementation were low due to organizational-level barriers. These barriers differ substantially across sites, necessitating tailoring of implementation strategies.

**References**

1. Shonkoff J, Boyce W, McEwen B. Neuroscience, molecular biology, and the childhood roots of health disparities. JAMA. 2009;301(21):2252. doi:10.1001/jama.2009.754.

2. Shonkoff J, Garner A, Siegel B, Dobbins M, Earls MF, Garner AS, et al. The lifelong effects of early childhood adversity and toxic stress. Pediatrics. 2011;129(1):e232-46. doi:10.1542/peds.2011-2663.

3. Leslie L, Mehus C, Hawkins J. Boat T, McCabe MA, Barkin S, et al. Primary health care: potential home for family-focused preventive interventions. Am J Prev Med. 2016;51(4):S106-18. doi:10.1016/j.amepre.2016.05.014.

4. McCormick E, Kerns SE, McPhillips H, Wright J, Christakis DA, Rivara FP. Training pediatric residents to provide parent education: a randomized controlled trial. Acad Pediatr*.* 2014;14(4):353-60.

5. Shah R, Kennedy S, Clark M, Bauer S, Schwartz A. Primary care-based interventions to promote positive parenting behaviors: a meta-analysis. Pediatrics. 2016;137(5):e20153393. doi:10.1542/ peds.2015-3393.

6. Kerns S, Negrete A, McCormick E. DBHR Triple P Initiative. 2014. Retrieved from http://theathenaforum.org/ sites/default/files/Triple%20P%20Rural%20Initative%20Final%20Report.pdf

## A99 Barriers in implementing an evidence-based, electronic screening program (eScreening) in three VA clinical care settings

### James Pittman^1^, Niloofar Afari^1,2^, Elizabeth Floto^1^, Laurie Lindamer^1,2^

#### ^1^VA Center of Excellence for Stress and Mental Health, San Diego, CA, USA; ^2^University of California, San Diego, Department of Psychiatry, La Jolla, CA, USA

##### **Correspondence:** James Pittman (James.Pittman@va.gov)

**Background**

The Department of Veterans Affairs (VA) serves 8.76 million Veterans each year, and the number increases by 8-12% annually [1]. The VA estimates that as many as 58% have a diagnosable mental illness [2] and mandates screening for Veterans in order to identify and treat those with mental health symptoms. The eScreening Program [3] is a tablet-based system developed for use in multiple VA settings to aid screening for mental health symptoms with promising results [4].

**Materials and Methods**

We conducted pre- and post- implementation interviews with leaders and frontline staff in primary care, mental health, and transition care management (TCM) programs to identify barriers to implementation of eScreening.

**Results**

Pre-implementation interviews identified three potential barriers to implementation: 1) lack of adequate personnel support; 2) lack of leadership support; and 3) technical challenges with the software. Only the primary care setting was unsuccessful in integrating eScreening as part of normal practice after six months. Results of post- implementation interviews: 1) confirmed pre-implementation concerns that eScreening increased work for staff; 2) suggested that leadership support for eScreening should include holding staff accountable to use it; and 3) disconfirmed problems with the technology as a barrier.

**Conclusions**

Despite increased work associated with the eScreening program and perceived lack of enforced accountability from leadership, eScreening was successfully implemented in two of three VA clinical care settings—mental health and TCM programs. The technology itself posed no barriers in any of the settings. An implementation strategy that accounts for increase staff work burden and includes staff accountability may help in future eScreening implementation efforts in the VA.

**References**

1. Department of Veterans Affairs. Annual benefits report, fiscal year 2013. 2013. http://www.benefits.va.gov/ REPORTS/abr/ABR-IntroAppendix-FY13-09262014.pdf. Accessed February 28, 2017.

2. Epidemiology Program, Post-Deployment Health Group, Office of Public Health, Veterans Health Administration, Department of Veterans Affairs. Analysis of VA health care utilization among Operation Enduring Freedom, Operation Iraqi Freedom, and Operation New Dawn Veterans, from 1st Qtr FY2002 through 4th Qtr Y2014. 2015. Washington, DC. http://www.publichealth.va.gov/ epidemiology/reports/oefoifond/health-care-utilization/. Accessed February 28, 2017

3. Pittman JO, Floto E, Lindamer L, Baker DG, Lohr JB, Afari N. VA eScreening program: technology to improve care for post-9/11 veterans. Psychol Serv. 2017;14(1):23.

4. Elnahal SM, Clancy C, Shulkin D. A framework for disseminating clinical best practices in the VA Health System. JAMA. 2017;317(3):255.

## A100 A scoping review of system-wide implementation of evidence-based practices for youth in public-sector service systems

### Kelsie Okamura^1^, Emily Becker-Haimes^1^, Kelly Zentgraf^1^, Ronnie Rubin^2^, Shawna Weaver^2^, Arthur Evans^2^, Byron Powell^3^, Rinad Beidas^1^

#### ^**1**^University of Pennsylvania, Philadelphia, PA, USA; ^2^City of Philadelphia Department of Behavioral Health and Intellectual disAbility Services, Philadelphia, PA, USA; ^3^Department of Health Policy and Management, Gillings School of Global Public Health, University of North Carolina at Chapel Hill, Chapel Hill, NC, USA

##### **Correspondence:** Kelsie Okamura (kelsieo@upenn.edu)

**Background**

Increasing the incorporation of evidence-based practice (EBP) into youth mental health is an important target for improving services [1,2] However, given the rapid rate at which evidence grows, coupled with estimated 17-year time-lags for incorporation [3], greater effort is needed to ensure youth receive evidence-based services. Implementation science is an effective facilitator of translating research to practice that acknowledges variation in EBP implementation at the system level [4]. However, across service systems, there appears to be many implementation process similarities and lessons to be learned [5,6].

**Materials and Methods**

We aim to synthesize these findings across youth state, county, and city public sector service systems. We intend to conduct a scoping review of the extant literature following established guidelines [7-10]. The six stages include: (1) clearly stating the research question and purpose of the study, (2) identifying relevant studies, (3) refining studies based on specific inclusion and exclusion criteria, (4) organizing and charting the data, (5) summarizing and tabling the results, and (6) seeking out consultation.

**Results**

Study identification were done through key word searches in electronic databases (e.g., Medline), searching reference lists, hand-searching key journals (e.g., Implementation Science), and reaching out to existing networks and organizations (e.g., Dissemination and Implementation Science Special Interest Group). Consultation will be given by implementation science and service system experts. We will identify common approaches to EBP implementation across systems and map them on to existing frameworks such as the taxonomy of implementation strategies proposed by Powell et al.[11], ecological influences on policy (i.e., policy ecology) [12], and phases of implementation (i.e., EPIS [4]).

**Conclusions**

Youth public-sector service systems often move faster than the rate of science and may be driven by factors like political mandates or changes in leadership, and many of these contexts and phases are interconnected within a service system.^4^ For example, do political mandates force systems to jump forward to implementation without considering Exploration or Preparation phases? Findings will have practical applications for policy-makers, system administrators, and researchers in identifying common implementation strategies, methodological approaches to implementation science, and propose reporting guidelines for future studies conducted outside formal research.

**References**

1. Institute of Medicine - Committee on Quality of Health Care in America. Crossing the quality chasm: a new health system for the 21st century. Washington, DC: National Academy Press; 2001.

2. Institute of Medicine. The future of disability in America. Washington, DC: National Academy Press; 2007.

3. Balas EA, Boren SA. Managing clinical knowledge for health care improvement. Yearb Med Inform.2000;1:65-70.

4. Aarons GA., Hurlburt M, Horwitz SM. Advancing a conceptual model of evidence-based practice implementation in public service sectors. Adm Policy Mental Health. 2011;38(1):4-23.

5. Bruns EJ, Hoagwood KE, Hamilton JD. State implementation of evidence-based practice for youths, part I: Responses to the state of the evidence. J Am Acad Child Adolesc Psychiatry. 2008;47(4):369-73.

6. McHugh RK, Barlow DH. The dissemination and implementation of evidence-based psychological treatments: a review of current efforts. Am Psychol. 2010;65(2):73.

7. Arksey H, O’Malley L. Scoping studies: towards a methodological framework. Int J Soc Res Methodol. 2005;8(1):19-32.

8. Moher D, Liberati A, Tetzlaff J, Altman DG; PRISMA Group. Preferred reporting items for systematic reviews and meta-analyses: the PRISMA statement. Ann Intern Med. 2009;151(4):264-9.

9. Levac D, Colquhoun H, O’Brien KK. Scoping studies: advancing the methodology. Implement Sci. 2010; 5(1): 69.

10. Colquhoun HL, et al. Scoping reviews: time for clarity in definition, methods, and reporting. J Clin Epidemiol. 2014;67(12):1291-4.

11. Powell BJ, Beidas RS, Rubin RM, Stewart RE, Wolk CB, Matlin SL, et al. Applying the policy ecology framework to Philadelphia’s behavioral health transformation efforts. Adm Policy Ment Health. 2016;43(6):909-26.

12. Raghavan R, Bright CL, Shadoin AL. Toward a policy ecology of implementation of evidence-based practices in public mental health settings. Implement Sci. 2008;3(1):26.

## A101 Evaluating the fit of the ecological framework for implementation influences in school settings

### Melissa Collier-Meek^1^, Austin Johnson^2^, Lisa Sanetti^3^

#### ^**1**^University of Massachusetts Boston, Boston, MA, USA; ^2^University of California Riverside, Riverside, CA, USA; ^3^University of Connecticut, Storrs, CT, USA

##### **Correspondence:** Melissa Collier-Meek (mel.colliermeek@umb.edu)

**Background**

Teachers struggle to deliver intervention with sufficient treatment fidelity, perhaps due to implementation influences that mediate or moderate treatment fidelity [1]. Potential implementation influences have been conceptualized in ecological frameworks that include the intervention, implementer, organization, and external environmental levels [1,2,3]. Although the ecological framework is a useful organizational tool, data are needed to hone and evaluate this model of implementation influences. To do so, we developed the Assessment of Ecological Implementation Influences (AEII), a measure to evaluate implementation influences across ecological levels.

**Materials and Methods**

To evaluate to what extent responses on the AEII reflect the hypothesized multi-level factor structure, two study phases were completed [4]. First, following initial content validation, 488 teachers completed the AEII and an initial exploratory factor analysis (EFA) was conducted. Second, 216 teachers completed the updated version of the AEII and a confirmatory factor analysis (CFA) and a follow-up EFA were conducted.

**Results**

Results of the initial EFA supported a five-factor solution (i.e., Intervention Influences, Implementation Support, School Context, Collegial Norms, and External Environment). The CFA suggested the model resulted in moderate to low fit. The follow-up EFA suggested that a major source of potential misfit in the CFA model may have resided within an erroneous conceptualization of the External Environment factor.

**Conclusions**

Findings suggest that the ecological model was not well suited to describe teachers’ perceptions of implementation influences. A four-factor model was proposed, but a five-factor model was chosen based upon the results of factor extraction analyses. Collegial Norms emerged as a unique factor, while the External Environment factor failed to fit within respondents’ perceptions. Additional research is needed to model how implementation influences operate on teachers’ treatment fidelity.

**References**

1. Sanetti LM, Kratochwill T. Towards developing a science of treatment integrity: Introduction to the special series. School Psychol Rev. 2009;38:445-59.

2. Durlak J, DuPre E. Implementation matters: a review of research on the influence of implementation on program outcomes and the factors affecting implementation. Am J Comm Psychol. 2008;41(3-4):327-50.

3. Long A, Sanetti LM, Collier-Meek M, Gallucci J, Altschaefl M, Kratochwill T. An exploratory investigation of teachers’ intervention planning and perceived implementation barriers. J Sch Psychol. 2016;55:1-26.

4. McCoach DB, Gable RK, Madura J. Instrument design in the affective domain. 3rd ed. New York: Springer; 2013.

## A102 Teachers’ reported barriers to delivering interventions in schools

### Melissa Collier-Meek^1^, Lisa Sanetti^2^

#### ^**1**^University of Massachusetts Boston, Boston, MA, USA; ^2^University of Connecticut, Storrs, CT, USA

##### **Correspondence:** Melissa Collier-Meek (mel.colliermeek@umb.edu)

**Background**

Teachers are responsible for delivering classroom management and behavior support plans, however, many struggle with implementation [1, 2]. Low treatment fidelity levels may be due to barriers to implementation related to the intervention, implementer, organization, or external level [3]. Teachers’ experience of these barriers within the context of specific interventions has not been evaluated. This exploratory study involves the analysis of barriers reported during Implementation Planning [4] by teachers implementing classroom management or behavior support plans.

**Materials and Methods**

Thirty-three teachers responsible for delivering classroom management or behavior support plans reported barriers during Implementation Planning [4]. Responses were coded for analysis. Barrier codes and associated ecological levels used in previous research [2] were applied in the current study. Thematic analysis was used to develop codes for responses that did not fit into prior barrier codes. Implementation barriers were coded by the first author, with 20% independently completed by a secondary coder with inter-rater agreement of 100%.

**Results**

The 20 teachers who implemented classroom management plans reported 55 barriers (M = 2.75, SD = 1.01), mostly related to Managing Problem Behavior, Remembering to Implement and Competing Responsibilities related to Other Activities. The 13 teachers who implemented behavior support plans reported 31 barriers (M = 2.38, SD = 1.12), mostly related to Competing Responsibilities related to Other Students, Managing Problem Behaviors, and Competing Responsibilities related to Other Activities. Across both interventions, most reported barriers were aligned with the Implementer level.

**Conclusions**

Teachers reported primarily struggling with implementation barriers related to their own role. Most of the frequently reported barriers were not previously identified in the literature [3]. Future research will need to systematically document these implementation barriers and evaluate how implementation barriers operate on treatment fidelity.

**References**

1. Mouzakitis A, Codding R, Tryon G. The effects of self-monitoring and performance feedback on the treatment integrity of behavior intervention plan implementation and generalization. J Posit Behav Interv. 2015;17(4):223-334.

2. Reddy L, Fabiano G, Dudek C, Hsu L. Instructional and behavior management practices implemented by elementary general education teachers. J Sch Psychol. 2013;51(6):683-700.

3. Long A, Sanetti LM, Collier-Meek M, Gallucci J, Altschaefl M, Kratochwill T. An exploratory investigation of teachers’ intervention planning and perceived implementation barriers. J Sch Psychol. 2016;55:1-26.

4. Sanetti LM, Collier-Meek M, Long A, Byron J, Kratochwill T. Increasing teacher treatment integrity of behavior support plans through consultation and implementation planning. J Sch Psychol. 2015;53(3):209-29.

## A103 Effective measurement for implementing school-based CBT: Validation of the Clinical Practices Assessment Measure (CPAM) as a tool for coaching-based implementation efforts

### Katherine Prenovost^1,2^, Shawna Smith^1,3,4^, Jennifer Vichich^1^, Emily Berregaard^1^, Elizabeth Koschmann^1^

#### ^1^Department of Psychiatry, University of Michigan Medical School, Ann Arbor, MI, USA; ^2^VA Ann Arbor Center for Clinical Management Research, Ann Arbor, MI, USA; ^3^University of Michigan, Institute for Social Research, Ann Arbor, MI, USA; ^4^University of Michigan, Department of Internal Medicine, Ann Arbor, MI, USA

##### **Correspondence:** Katherine Prenovost (kprenovo@med.umich.edu)

**Background**

Coaching-based implementation strategies may improve access to evidence-based practices (EBPs) in non- traditional settings by providing training and follow-up support from treatment experts. Schools in particular are an appropriate target for implementation as delivery could significantly improve treatment access. Regrettably, school professionals (SPs) are rarely trained to deliver EBPs. The TRAILS program piloted a coaching implementation strategy incorporating didactic CBT training for SPs followed by expert coaching. Successful implementation and evaluation of TRAILS requires development and use of an instrument to assess dimensions of CBT proficiency among SPs. TRAILS developed a measure for this purpose, the Clinical Practices Assessment Measure (CPAM) that aims to assess three dimensions of SP competency in CBT: Clinical Expertise (CE), Skills Use Frequency (UF), and Perceptions of CBT (P). Results from the first psychometric evaluation of the CPAM are presented.

**Materials and Methods**

The CPAM consists of 40 self-report items and 10 criterion-referenced items measuring responses to two hypothetical case vignettes. Data were collected from a sample of 53 SPs from 24 schools, prior to initial training, thus representing SP competency at baseline. Nine self-report items were dropped due to insufficient variability. Total score on the criterion-referenced vignette items was then regressed on the CPAM subscales and covariates: age, sex, race, degree area, years practicing, theoretical orientation, and prior CBT training.

**Results**

Internal consistency of the 31 items was high (Cronbach α=.996). Exploratory factor analysis indicated there was a general Clinical (CE+UF) factor (N=22 items; eigenvalue=27.7) and a second factor isolating the P items and 1 UF item (N=10 items; eigenvalue=1.8). Factors were highly correlated (r=0.81). Linear regression of the vignette total scores (M=4.7, SD=2.2, range=0-9) on the Clinical factor (M=62.9, SD=19.1, range=23-103) and P scale scores (M=37.5, SD=5.3, range=28-50) revealed that after adjustment, the Perception scale was associated with better vignette scores, ($\widehat{\upbeta}$ =.46, *p* < .01), with an increase of 10 points on P scale reflecting an improvement of 1.8 points on vignette score. The CE+UF scale was not predictive ($\widehat{\upbeta}$ =.007).

**Conclusions**

Understanding how and why the coaching implementation strategy works to improve uptake of EBPs requires development of measures that capture mechanisms of effectiveness. The CPAM measure for evaluating SP response to training and coaching has potential for illustrating these mechanisms. Future work will examine change in the CPAM over the course of training and coaching, as well as further validation and refinement using data from a larger set of SPs.

## A104 Using ecological momentary assessment (EMA) to collect data on the fidelity of implementation of school-wide behavioral supports

### Hao-Jan Luh^1^, Lisa Sanetti^1^, Melissa Collier-Meek^2^

#### ^**1**^University of Connecticut, Storrs, CT, USA; ^2^UMASS-Boston, Boston, MA, USA

##### **Correspondence:** Hao-Jan Luh (hao-jan.luh@uconn.edu)

**Background**

Teacher self-report is an appealing option for treatment fidelity assessment as it is feasible, efficient, and aligned with educational practice of asking for teacher reports. Yet, it is not currently recommended as data indicate teachers overestimate their treatment fidelity [1, 2]. Pilot studies indicate self-report measures with detailed questions, daily recall, and independent completion can result in accurate treatment fidelity data [3,4]. Ecological momentary assessment (EMA) is a form of self-report that (a) reduces recall biases and episodic memory decay; (b) increases ecological validity; (c) allows repeated sampling in real time; (d) increasingly involves using technology; and (e) has been found to be defensible, efficient, and feasible across multiple fields [5].

**Materials and Methods**

We collected data on teachers’ implementation of a school-wide behavioral support intervention. The teacher was provided with an iPod Touch programmed to alert her to complete an EMA self-report. For 15 days, the teacher’s implementation was videotaped and she completed three EMA self-reports per day. Videos were coded for implementation behaviors.

**Results**

Agreement between (a) each EMA self-report and the treatment fidelity methods (observations, permanent product) and (b) one EMA sample for each day and a composite of all EMA samples for each day will be examined through the appropriate correlation coefficients.

**Conclusions**

The proposed research will add to the limited literature base on methods for assessing treatment fidelity in schools.

**References**

1. Cochrane WS, Laux JM. A survey investigating school psychologists’ measurement of treatment integrity in school-based interventions and their beliefs about its importance. Psychol Sch. 2008;45:499-507.

2. Noell GH, Gansle KA. Assuring the form has substance: Treatment plan implementation as the foundation of assessing response to intervention. Assess Eff Interv. 2006;32:32-9.

3. Sanetti LMH, Chafouleas SM, O’Keeffe BV, Kilgus SP. Treatment integrity assessment of a daily report card intervention: a preliminary investigation of two methods and frequencies. Can J Sch Psychol, 2013;28:261-76.

4. Sanetti LMH, Kratochwill TR. An evaluation of the treatment integrity planning protocol and two schedules of treatment integrity self-report: impact on implementation and report accuracy. J Educ Psychol Consult. 2011;21:284-308.

5. Kubiac T, Krog, K. Computerized sampling for experiences and behavior. In Mehl MR, Conner TS, editors. Handbook of research methods for studying daily life. New York, NY: The Guilford Press; 2012. p.124-43.

## A105 Walking the talk: Factors associated with practitioners’ initial use of an evidence-based parenting intervention following training

### Andrea Negrete^1^, Erin McCormick^2^, Cathea Carey^3^, Wren Haaland^2^, Scott Waller^4^, Suzanne E.U. Kerns^5,3^

#### ^**1**^University of Virginia, Charlottesville, VA, USA; ^2^Center for Child Health Behavior and Development, Seattle Children’s Research Institute, Seattle, WA , USA; ^3^University of Washington, Seattle, WA, USA; ^4^Washington State Division of Behavioral Health and Recovery, Olympia, WA, USA; ^5^University of Denver, Denver, CO, USA

##### **Correspondence:** Andrea Negrete (an8ee@virginia.edu)

**Background**

There is an increased push towards adoption of evidence-based practices (EBPs) across child-serving systems. However, training alone does not always lead to EBP uptake and adoption.[1] Prior research suggests an implementation approach that considers the social context such as quality of training, practitioner and client variables, and organizational supports [1,2]. The current study explores systems-contextual implementation factors that predict timely use of the Positive Parenting Program (Triple P), an evidence-based parenting intervention and seeks to document additional impacts of training and barriers to implementation.

**Materials and Methods**

Participants in the current study included 37 providers from three rural communities trained in Triple P. Participants completed a baseline survey reporting on demographics, attitudes towards EBPs, self-efficacy, training satisfaction, perceptions of their referral network, and communication and collaboration among service providers. A six-month follow-up survey was administered on provider use of Triple P since training, generalization of training to other areas of their work and barriers to delivering Triple P. Monthly service delivery reports on Triple P utilization were also collected. Participants represented a diverse number of service delivery systems, agencies, and training backgrounds with 43% from mental health and social services, 32% from healthcare, and 24% from other work settings.

**Results**

Fifty-four percent of respondents reported having used the Triple P intervention with any family in the first six months following training. Analyses using exact logistic regression suggested that practitioner self-efficacy and attitudes toward evidence based practice predicted using Triple P within the first six months. Health care workers were marginally more likely to use Triple P compared to those in other settings (e.g., schools, churches) but no difference was found between health care and mental health. Across users and non-users of Triple P, the vast majority of providers (83%) reported incorporating at least one core component of Triple P training into other aspects of their work. Thematic coding of qualitative responses on barriers to implementation revealed three prominent themes related to financial barriers (organization- and client-level), referrals, and implementation-related barriers. Results of this study have been submitted for publication.

**Conclusions**

The results of this exploratory prospective study suggest that individual-level practitioner factors such as attitudes towards EBPs and self-efficacy were predictive of Triple P utilization after training. Generalizability of training suggests there are other measurable benefits of evidence-based training beyond direct use of the intervention with families. These findings elucidate factors of importance for those interested in supporting EBP implementation.

**References**

1. Beidas RS, Kendall PC. Training therapists in evidence-based practice: a critical review of studies from a systems/contextual perspective. Clin Psychol. 2010;17(1):1-30.

2. Shapiro CJ, Prinz RJ, Sanders MR. Sustaining use of an evidence-based parenting intervention: practitioner perspectives. J Child Fami Stud. 2015;24(6):1615-24.

## A106 A systematic review of barriers and facilitators to implementing trauma-focused interventions for children and youth

### Byron Powell^1^, Sheila Patel^1^, Amber Haley^1^, Colleen Katz^2^, George Ake^3^, Lisa Amaya-Jackson^3^

#### ^1^Department of Health Policy and Management, Gillings School of Global Public Health, University of North Carolina at Chapel Hill, Chapel Hill, NC, USA; ^2^Silberman School of Social Work, Hunter College, New York, NY, USA; ^3^Department of Psychiatry & Behavioral Sciences, Duke University School of Medicine, Durham, NC, USA

##### **Correspondence:** Byron Powell (sheila@unc.edu)

**Background**

Children and youth experience trauma at alarming rates, which can lead to serious mental health problems including PTSD, behavioral problems, depressive symptoms, and anxiety [1,2]. There are number of evidence- based treatments (EBTs) for those who experience emotional or behavioral difficulties related to trauma [3]; however, much like other EBTs, they are underutilized, and when they are adopted, implementation problems limit their effectiveness [4,5]. Improving the integration of trauma-focused interventions will require the identification, development, and testing of implementation strategies that effectively address multilevel implementation determinants (barriers and facilitators). The purpose of this study is to conduct a systematic review of the literature to identify key determinants of implementing trauma-focused interventions for children and youth.

**Materials and Methods**

We will search CINAHL, PubMed, and PsycINFO using terms related to trauma, children and youth, psychosocial interventions, and implementation to identify English-language peer-reviewed journal articles related to the implementation of evidence-based trauma-focused interventions for children and youth (<19 years). Two researchers (SP & AH) will independently review abstracts and articles selected for full-text review, we will document reliability of coding, and any discrepancies will be discussed with the full authorship team until consensus is reached. Qualitative and quantitative data related to determinants of trauma-focused intervention implementation will be abstracted using a structured abstraction form.

**Results**

Results will be synthesized using Aarons and colleagues Exploration, Preparation, Implementation, and Sustainment model [6]. Findings will be used in conjunction with a mixed methods assessment of determinants of implementing Trauma-Focused Cognitive-Behavioral Therapy, and will ultimately be used to inform implementation at the organizational-level within a randomized pilot trial of a systematic approach to selecting and tailoring implementation strategies.

**Conclusions**

This study will contribute to the literature by yielding a comprehensive picture of the determinants of implementing trauma-focused interventions that is grounded in an established conceptual model of implementation in public service settings. Findings will be immediately useful to stakeholders attempting to improve the implementation of trauma-focused interventions, and will be to applied within a National Child Traumatic Stress Network-affiliated study that will develop and pilot a systematic approach to selecting and tailoring implementation strategies.

This study will also model how systematic reviews of qualitative, quantitative, and mixed methods studies of implementation can be used to identify determinants (i.e., mechanisms) of implementation for other interventions and contexts.

**References**

1. Copeland WE, Keeler G, Angold A, Costello EJ. Traumatic events and posttraumatic stress in childhood. Arch Gen Psychiatry. 2007;64(5):577-84.

2. McLaughlin KA, Koenen KC, Hill E, Petukhova M, Sampson NA, Zaslavsky AM, Kessler RC.Trauma exposure and posttraumatic stress disorder in a US national sample of adolescents. J Am Acad Child Adolesc Psychiatry. 2013;52:815-30.

3. Dorsey S, McLaughlin KA, Kerns SEU, Harrison JP, Lambert HK, Briggs EX, et al. Evidence base update for psychosocial treatments for children and adolescents exposed to traumatic events. J Clin Child Adolesc Psychol. 2017;46(3):303-30.

4. Allen B, Johnson JC. Utilization and implementation of Trauma-Focused Cognitive- Behavioral Therapy for the treatment of maltreated children. Child Maltreat. 2012;17(1):80-5. doi:10.1177/1077559511418220

5. Powell BJ, Hausmann-Stabile C, McMillen JC. Mental health clinicians’ experiences of implementing evidence-based treatments. J Evid Based Soc Work. 2013;10(5):396-409.

6. Aarons GA, Hurlburt M, Horwitz SM. Advancing a conceptual model of evidence- based practice implementation in public service sectors. Adm Policy Ment Health. 2011;38:4-23. doi:10.1007/s10488-010-0327-7

7. Baker R, Camosso-Stefinovic J, Gillies C, Shaw EJ, Cheater F, Flottorp S, Robertson N, Wensing M, Fiander M, Eccles MP, Godycki-Cwirko M. Tailored interventions to address determinants of practice. Cochrane Database Syst Rev. 2015;(4):CD05470.

8. Bosch M, van der Weijden T, Wensing M, Grol R. Tailoring quality improvement interventions to identified barriers: A multiple case analysis. J Eval Clin Pract. 2007;13:161-8. doi:10.1111/j.1365-2753.2006.00660.x

## A107 Expert consultation and caseload: Training mechanisms to facilitate clinician skill and implementation

### Carrie Jackson^1^, Amy Herschell^1,2^, Kristen Schaffner^1,2^, Nicholas Turiano^1^, Cheryl McNeil^1^

#### ^**1**^West Virginia University, Morgantown, WV, USA; ^2^University of Pittsburgh Medical Center, Pittsburgh, PA, USA

##### **Correspondence:** Carrie Jackson (cbjackson@mix.wvu.edu)

**Background**

Consultation has been linked to improvements in clinician knowledge, skill, and client outcomes [1]. However, little research has investigated the association between consultation and implementation outcomes (e.g., acceptability, feasibility), and the role of individual clinician characteristics. Given the variability of clinicians participating in trainings on evidence-based treatments, a greater understanding of how these characteristics impact the effectiveness of trainings.

**Materials and Methods**

This study utilized data from a statewide implementation trial of Parent-Child Interaction Therapy, examining the effects of three different training designs on various outcomes. Relevant to the current study, 32 therapists from community agencies participated in a cascading training for PCIT. Following the initial training, therapists attended up to 24 1-hour consultation calls conducted by one of three expert trainers. Expert trainers conducted measures of consultation content and attendance following each consultation call. Simple and multiple linear regression analyses were conducted to predict post-training knowledge, skill, acceptability, and feasibility, as well as to examine clinician variables (e.g., caseload, licensure, years of experience) that moderate these relations.

**Results**

Clinicians attended an average of 17.60 consultation calls, and had an average PCIT training caseload of 3.81 families. Consultation call attendance significantly predicted post-training skill. However, the impact of consultation call attendance on skill was qualified by a significant interaction with PCIT caseload.

**Conclusions**

These results suggest that clinicians who attended a majority of consultation calls and had a high PCIT caseload demonstrated the greatest post-training skill. These results indicate that caseload is important to consider for training guidelines and efforts.

**Reference**

1. Nadeem E, Gleacher A, Beidas RS. Consultation as an implementation strategy for evidence-based practices across multiple contexts: Unpacking the black box. Adm Policy Ment Health. 2013;40(6):439-50. doi:10.1007/s10488-013-0502-8.

## A108 Summarizing implementation data from routine delivery of a parenting intervention across multiple-sites: Using an interactive dashboard to visualize data trends

### W. Alex Mason, Robert Oats

#### Boys Town, National Research Institute for Child and Family Studies, Boys Town, NE, USA

##### **Correspondence:** W. Alex Mason (walter.mason@boystown.org)

**Background**

Providers in service organizations need access to timely, organized implementation data; however, the tasks of collecting, managing, and reporting on such data can be daunting. In particular, without dynamic and intuitive ways of viewing results, implementation data likely will not be used for the improvement of service delivery. This presentation describes an interactive dashboard designed to help providers visualize implementation data trends, and illustrates the tool with routine data collected in a service setting on Common Sense Parenting (CSP).

**Materials and Methods**

CSP is a six-session, classroom-based parenting intervention developed by Boys Town. Content is delivered via structured learning activities including skills instruction, modeling, and practice. Since 2014, Boys Town has collected 151 fidelity observations of 79 trainers by 36 evaluators at 11 sites. The fidelity observation form contains 21 items with 3 subscales: Trainer Skills, Skill Practice Leadership, and Professional Presence. A 5-point rating scale for each item is used to rate adherence and quality. A rating of 3 (meets criteria) is used as a benchmark for minimally successful implementation. Observation data were entered into an Excel spreadsheet and summarized on a “dashboard” that uses a variety of visual aids to help detect trends (e.g., conditional formatting, databars, icon sets, sparklines) and allows users to sort and filter the data by various categories (e.g., month/quarter/year of observation, site, evaluator, trainer).

**Results**

Results indicate that the Skills Practice subscale was below criteria (2.9), while Trainer Skills (3.2) and Professional Presence (3.5) were above criteria. Examining the subscales across sessions indicated only 1 session below criteria for the Trainer Skills subscale, 4 sessions below criteria for Skills Practice, and no sessions below criteria for Professional Presence. Examining individual items across sessions indicated that session 1 had the highest percentage of items below criteria (62%). The items with the highest percentages below criteria across all sessions were related to skills practice (e.g., deliver conceptual feedback (100%), practice documentation (83%), and time management (67%).

**Conclusions**

Efficiently and effectively using implementation data can be challenging for providers. This presentation illustrates a tool that can be used to identify areas of implementation that are below criteria, thereby requiring improvements. In contrast to static charts and tables, this interactive dashboard helps users generate tailored reports that chart meaningful data trends. Although illustrated for CSP, the tool potentially could be modified for other programs and, ultimately, holds promise for helping to ensure quality program implementation.

## A109 Redirecting the infrastructure and intervention efforts of treatment providers as a mechanism for increasing the implementation of evidence-based prevention

### W. Alex Mason, Jasney Cogua-Lopez, Ronald Thompson

#### Boys Town, National Research Institute for Child and Family Studies, Boys Town, NE, USA

##### **Correspondence:** W. Alex Mason (walter.mason@boystown.org)

**Background**

Few evidence-based preventive interventions are implemented at scale, reflecting a science-to-practice gap in prevention science. By contrast, many communities already have well-developed services for treating emotional and behavioral disorders. Helping established treatment providers redirect a portion of their infrastructure and intervention efforts to evidence-based prevention could provide an efficient and cost-effective way to grow the reach and impact of prevention in community settings. We present a framework for working with and within treatment service organizations to redirect their focus to prevention, drawing on Kotter’s eight-step model of organizational change [1] and provide a case study.

**Materials and Methods**

We draw on the eight steps of the framework to illustrate a shift toward implementation of evidence-based prevention at Boys Town, a national service organization with a 100-year history of working with troubled youth and their families. In 2014, Boys Town began implementing a strategic plan to supplement existing treatment offerings with preventive services to reach more children and families in community settings. Organizational characteristics and specific steps taken to implement the strategic initiative are discussed. To illustrate progress, we also report on routine program data (e.g., pretest-posttest, model implementation forms) for close to 900 cases across four different programs that were collected on the dissemination, implementation, and outcomes of preventive services in Nebraska and Nevada in 2016.

**Results**

Based on a strong sense of urgency for change due to external pressures and internal motivations, Boys Town developed a guiding coalition to roll out the strategic initiative for prevention. Steps to empower organizational change included removing barriers (e.g., increasing the diversity of providers) and providing education about prevention concepts and program delivery. Short-term wins were accomplished (e.g., increasing visibility in communities). Geo-mapping data reflected the anticipated growth of preventive services in targeted areas. Implementation quality as reflected in data on organizationally-specified benchmarks varied across programs and sites. Further, pretest-posttest and follow-up results have shown anticipated improvements in parenting and reductions in child problem behaviors.

**Conclusions**

Although there are challenges (e.g., finding ways to sustainably pay for preventive services), the current framework could have relevance for other treatment organizations. Rather than building prevention capacity from the ground up in community settings, helping established treatment organizations adopt a culture of prevention and redirect their efforts holds promise for expanding the dissemination and implementation of evidence-based preventive interventions for public health benefit.

**Reference**

1. Kotter JP. Leading change. Harvard Business Review Press: Boston, MA; 1996.

## A110 Evaluating implementation of adolescent substance use screening in public schools: Perspectives from multiple stakeholders

### Marvin So^1^, Allian Rodriguez^2^

#### ^**1**^Northeastern University Bouvé College of Health Sciences, Boston, MA, USA; ^2^University of Massachusetts Amherst, Amherst, MA, USA

##### **Correspondence:** Marvin So (marvin.so@mail.harvard.edu)

**Background**

Educational systems have increasingly recognized the importance of identifying substance use among adolescents in order to prevent long-term consequences. School nurses are uniquely positioned to screen for substance use, and provide counseling, education, and referrals to address students at-risk. In response to increasing substance misuse and overdose prevalence, Massachusetts passed legislation requiring public schools to engage in substance use prevention and education. This included Screening, Brief Intervention, and Referral to Treatment (SBIRT-in-Schools), a model that has been successfully implemented in non-educational clinical settings [1].

**Materials and Methods**

Pursuant to the legislation, SBIRT-in-Schools was to be scaled-up from nine pilot districts to 200. Thus, identification of key factors critical for successful implementation was warranted. Given heterogeneity in extant implementation measures [2] and the desire to explore implementation processes, we conducted qualitative, semi-structured interviews with diverse stakeholders. We used maximum variation sampling for administrators: district nurse leaders (N=9), substance use prevention coalition directors (N=9), and state training/technical assistance (TA) providers (N=3). Expert sampling was used for screening personnel: guidance counselors (N=7) and school nurses (N=6). Interviews were audio-recorded, transcribed, and analyzed using a general inductive approach [3]. A focus group involving a portion of interview participants served as a member-check to confirm validity of findings (N=11). Fixsen et al.’s [4] conceptualization of implementation stages and implementation drivers served as a framework guiding interpretation [4].

**Results**

Most administrators identified coalition partnerships as critical for the exploration and installation stages, as was eliciting buy-in from parents via town halls and the salience of the overdose epidemic. Screening personnel noted competing mandated screenings and uncooperative teaching staff as dominant challenges for initial and full implementation. Administrators underscored the mismatch between public law and dedicated funding as a barrier to long-term maintenance. Finally, communication strategies were frequently cited across implementation stages, particularly framing the intervention as facilitating access to trusted adults. Formalized systems of support (i.e., training and TA) were deemed less critical than were informal support systems (e.g., debrief meetings).

**Conclusions**

We used findings to develop an implementation toolkit and webinar for districts, as well as an online network facilitating informal support. Identified themes can serve as constructs for quantitative investigations examining associations between implementation and both short-term (e.g., # of children screened and referred) and long-term (e.g., substance use prevalence from youth risk behavior surveys) outcomes. Future research should investigate student/family perceptions in order to minimize opt-outs and optimize likelihood of accessing referrals.

**References**

1. Ryan SA, Martel S, Pantalon M, Martino S, Tetrault J, Thung SF, et al. Screening, brief intervention, and referral to treatment (SBIRT) for alcohol and other drug use among adolescents: evaluation of a pediatric residency curriculum. Subst Abus. 2012;33(3):251-60.

2. Chaudoir SR, Dugan AG, Barr CH. Measuring factors affecting implementation of health innovations: a systematic review of structural, organizational, provider, patient, and innovation level measures. Implement Sci. 2013;8(1):22.

3. Thomas D.R. A general inductive approach for analyzing qualitative evaluation data. Am J Eval. 2012;27(2):237-46.

4. Fixsen DL, Naoom SF, Blase KA, Friedman RM, Wallace F. Implementation research: a synthesis of the literature. Tampa: University of South Florida, Louis de la Parte Florida Mental Health Institute, National Implementation Research Network; 2005.

## A111 Impact of CBT training program on perceived clinician burnout

### Tanya Shtutman^1^, Hollie Granato^1^, Urmi Patel^1^, Jillian Yeargin^1^

#### ^**1**^Harbor-UCLA, University of California, Los Angeles, Harbor City, CA, USA

##### **Correspondence:** Tanya Shtutman (tshtutman@gmail.com)

**Background**

Literature demonstrates that there is high burn out across mental health professionals [1]. The most commonly used definition of burn out comprises three components, including emotional exhaustion, depersonalization, and reduced personal accomplishment [2,3]. Research has demonstrated that healthcare professionals’ burnout impacts performance [4], turn over, physical and emotional health [5], and impaired memory [6].

**Materials and Methods**

This program evaluation examined the pre and post data measures of the Professional Quality of Life Scale (ProQOL-5) from the CBT roll-out, which is an initiative to train all frontline clinicians in the Los Angeles Department of Mental Health (LADMH) on Cognitive Behavior Therapy treatment. The data aimed to answer the following question: Does the CBT training program have a positive impact on burn out? The hypothesis was that the CBT training program will have a negative correlation with burn out and that the mean burnout will be significantly lower after the end of the training compared to before training. Paired t-tests were used to answer this question.

**Results**

A paired-samples t-test was conducted to compare ProQOL-5 scores at the first day of training (pre) and at the booster session (post). There was not a significant difference in the scores for pre (M= 84.18, SD=6.7) and post (M=82.97, SD=7.0); t=1.2, p = .236 The results indicate that there was no significant between pre/post on ProQOL. A paired-samples t-test was also conducted to compare subscales for compassion satisfaction, burnout, and secondary traumatic stress. There was not a significant difference in the scores.

**Conclusions**

The results demonstrated that there is no significance as it relates to burnout between pre and post measures on the ProQOL, including the subtests. However, it is important to note that burnout in general is low for LADMH clinicians. This finding demonstrates that these clinicians are not feeling overextended, depleted, and/or fatigued as it relates to their job. Additionally, based on the results, these clinicians do not report a reduced sense of personal accomplishment. However, the findings suggest that although overall these clinicians reported low burn out, the results also demonstrated low compassion satisfaction. Further conclusions and recommendations for future research are discussed.

**References**

1. Morse G, Salyers MP, Rollins AL, Monroe-DeVita M, Pfahler C. Burnout in mental health services: a review of the problem and its remediation. Adm Policy Ment Health. 2012;39(5):341-52.

2. Maslach, C. Burnout: a multidimensional perspective. In Schaufeli WB, Maslach C. Marek T, editors. Professional burnout: recent developments in theory and research. Washington, DC: Taylor and Francis; 1993. p. 19-32.

3. Maslach C, Jackson SE, Leiter MP. Maslach Burnout Inventory, 3rd ed., Palo Alto, CA: Consulting Psychologists Press; 1996.

4. Taris TW. Is there a relationship between burnout and objective performance? A critical review of 16 studies. Work & Stress. 2006;20(4):316-34.

5. Stalker C, Harvey C, Partnerships for Children and Families Project. Professional burnout: A review of theory, research, and prevention. Waterloo, Canada: Social Work, Wilfrid Laurier University; 2002.

6. Peterson U, Demerouti E, Bergström G, Samuelsson M, Åsberg M, Nygren Å. Burnout and physical and mental health among Swedish healthcare workers. J Adv Nurs. 2008;62(1):84-95.

## A112 Reliability and validity of the Cognitive-Behavioral Therapy for Anxiety in Youth Adherence Scale (CBAY-A) adapted for use with modular treatments

### Stephanie Violante^1^, Chantelle Miller^1^, Lucas Melo^1^, Michael Southam-Gerow^1^, Bryce McLeod^1^, Bruce Chorpita^2^, John Weisz^3^

#### ^**1**^Virginia Commonwealth University, Richmond, VA, USA; ^2^University of California, Los Angeles, Los Angeles CA, USA; ^3^Harvard University, Cambridge, MA, USA

##### **Correspondence:** Stephanie Violante (violantes@vcu.edu)

**Background**

The measurement of adherence, the extent to which the therapy occurred as intended [1], is a key aspect of treatment integrity research and critical for identifying gaps in implementation of evidence-based treatments [2]. The Cognitive–Behavioral Therapy for Anxiety in Youth Adherence Scale (CBAY-A) is an observational measure designed to capture therapist adherence to common practice elements found in individual cognitive-behavioral therapy (ICBT) for youth anxiety [3]. The initial items on the CBAY-A scale showed evidence of reliability and representational validity [3]. There has been a recent shift toward modularized approaches to treatment wherein practice elements are used to treat youth problems such as anxiety, depression, and disruptive behavior problems.

**Materials and Methods**

The CBAY-A was adapted with 10 new items to capture therapist adherence to practice elements for anxiety, depression, and disruptive behavior problems. This study aims to replicate previous reliability and validity findings of the CBAY-A with the adapted measure. The adapted CBAY-A was used to assess therapeutic interventions delivered as part of Standard Manualized Treatment (SMT), Modular MATCH Treatment (MMT), and usual care (UC) in community settings [4]. This study uses a sample of N = 796 recordings from N = 38 youth being treated for anxiety by N = 26 therapists. All sessions were independently rated by two coders.

**Results**

All original CBAY-A model items exhibited the expected full range of scores with a range of at least 5 points. Overall, the new items were observed infrequently; four items exhibited a restricted range and five items were not observed. The 12 original CBAY-A items for anxiety demonstrated a mean ICC of .85 (SD = .05, range .76 to .94) whereas the 10 new items demonstrated a mean ICC of .64 (SD = .27, range .33 to .87). All coded item scores demonstrated convergent validity with corresponding items on an observational measure of cognitive and behavioral interventions (TPOCS-RS) [6], with medium to large correlations ranging from r = .31 to r = .91. Finally, the majority of inter-item correlations are small, ranging from r = .002 to r = .291, supporting overall discriminant validity.

**Conclusions**

Results are supportive of the reliability and validity of the original CBAY-A items for use with our new sample; however, the new adherence items were not coded with enough frequency to adequately assess score reliability and validity.

**References**

1. Perepletchikova F, Kazdin AE. Treatment integrity and therapeutic change: issues and research recommendations. Clin Psychol. 2005;2(4):365-83. doi:10.1093/clipsy.bpi045

2. Hagermoser Sanetti LM, Kratochwill TR. Toward developing a science of treatment integrity: introduction to the special series. School Psych Rev. 2009;38(4):445-59.

3. Southam-Gerow MA, McLeod BD, Arnold CC, Rodriguez A, Cox JR, Reise SP, et al. Initial development of a treatment adherence measure for cognitive–behavioral therapy for child anxiety. Psychol Assess. 2016;28(1):70-80. http://dx.doi.org/10.1037/pas0000141

4. Weisz JR, Chorpita BF, Palinkas LA, Schoenwald SK, Miranda J, Bearman SK, et al. Testing standard and modular designs for psychotherapy treating depression, anxiety, and conduct problems in youth: a randomized effectiveness trial. Arch Gen Psychiatry. 2012;69(3):274-82.

5. Cicchetti CV. Guidelines, criteria and rules of thumb for evaluating normed and standardized assessment instruments in psychology. Psychology Assess. 1994;6:284-90. http://dx.doi.org/10.1037/1040- 3590.6.4.284

6. McLeod, BD, Smith, MM, Southam-Gerow, MA, Weisz, JR, Kendall, PC. Measuring differentiation for implementation research. Psychol Assess. 2015;27:314-25. http://dx.doi.org/10.1037/pas0000037

## A113 Contribution of teacher-child relationships and self-efficacy during BEST in CLASS: A moderated mediation analysis

### Katrina Markowicz^1^, Kristen Granger^2^, Jason Chow^1^, Rachel Kunemund^1^, Jessica Wright^1^, Kevin Sutherland^1^, Maureen Conroy^3^

#### ^**1**^Virginia Commonwealth University, Richmond, VA, USA; ^2^Arizona State University, Tempe, AZ, USA; ^3^University of Florida, Gainesville, FL, USA

##### **Correspondence:** Katrina Markowicz (markowiczk@mymail.vcu.edu)

**Background**

BEST in CLASS (Behavioral, Emotional, & Social Training: Competent Learners Achieving School Success) is a manualized teacher-delivered early childhood intervention with documented success in reducing preschoolers’ problem behaviors [1]. However, less is known about the processes through which BEST in CLASS ameliorates problem behaviors. A focus on process is crucial for contributing knowledge about potential pathways that may result in better outcomes.

**Materials and Methods**

A total of 185 teachers were randomly assigned to either program exposure (BEST in CLASS, n = 92) or business- as-usual (n = 93) conditions. BEST in CLASS teachers participated in one full-day training and in 14-weeks of practice-based coaching. Participating children (n=232 BEST in CLASS; n = 234 business as usual) were identified by teachers at risk for an emotional/behavior disorder. To investigate the mechanisms through which BEST in CLASS reduces child problem behaviors, a moderated multiple mediator model was conducted. Two potential mediators were examined (teacher-child closeness and conflict) and a moderator (teachers’ behavior management efficacy). Measures included teacher reported behavior management efficacy (pretest), teacher-child relationships (posttest), and observations of children’s engagement in problem behaviors (posttest).

**Results**

Children’s problem behaviors decreased from pretest to posttest in program exposure classrooms. The relation between program exposure and problem behaviors was significantly mediated through teacher-child closeness (posttest); BEST in CLASS increased ratings of teacher-child closeness, and teacher-child closeness was negatively associated with children’s problem behaviors. The relation between program exposure and problem behaviors was not significantly mediated through teacher-child conflict (posttest). The relation between program exposure and teacher- child closeness (a path) was significantly moderated by teachers’ behavior management efficacy (pretest). Simple slopes revealed teachers’ behavior management efficacy was negatively associated with teacher-child closeness for BEST in CLASS teachers.

**Conclusions**

BEST in CLASS is an effective prevention program for reducing preschool behavior problems, and its efficacy is, in part, through teacher-child closeness. Moreover, teachers’ initial sense of efficacy may influence program effectiveness; BEST in CLASS had a stronger influence on teacher-child closeness when teachers reported lower levels of behavior management efficacy at pretest. Findings identify mechanisms through which prevention efforts can reduce children’s problem behavior. Understanding these mechanisms can inform program development and subsequent implementation.

**Reference**

1. Conroy MA, Sutherland KS, Algina JJ, Wilson RE, Martinez JR, Whalon KJ. Measuring teacher implementation of the BEST in CLASS intervention program and corollary child outcomes. J Emot Behav Disord. 2015;23:144-55. doi: 10.1177/1063426614532949

## A114 Evaluation of data-driven delivery of implementation supports in education

### Lisa Sanetti^1^, Melissa Collier-Meek^2^, Hao-Jan Luh^1^

#### ^**1**^University of Connecticut, Storrs, CT, USA; ^2^University of Massachusetts-Boston, Boston, MA, USA

##### **Correspondence:** Lisa Sanetti (lisa.sanetti@uconn.edu)

**Background**

Over the past 10 years, multi-tiered systems of support for delivery of academic and behavioral interventions (e.g., Response-to-Intervention and Positive Behavior Interventions and Support) have been widely adopted by schools nationwide. To effectively impact student outcomes, interventions delivered across the tiers must be implemented with adequate fidelity, but rarely are [1]. Numerous implementation strategies are available, but they have different intensities, are appropriate at different stages of implementation, and are designed for different implementation issues. Frameworks to determine what strategy to implement, when, and for whom are lacking. We propose organization of implementation strategies in a multi-tiered implementation support (MTIS) framework that recognizes strategies have different intensities and may be appropriate at different stages of implementation or for different implementation issues [1].

**Materials and Methods**

A multiple baseline design across six elementary school teachers was used to evaluate the impact of implementation strategies delivered through a MTIS framework on teachers’ delivery of classroom management strategies. Direct training was delivered to all implementers and based on responsiveness, increasingly intensive implementation strategies (implementation planning, participant modeling) were provided. Adherence, quality, and student teachers’ implementation of classroom management practices and subsequent student outcomes were measured via direct observation throughout the study.

**Results**

Results suggest (a) all teachers responded to implementation supports, but response magnitude was different across teachers and supports; (b) higher levels of treatment fidelity generally were associated with fewer disruptive behaviors; and (c) duration of strategies increased across tiers.

**Conclusions**

Teachers demonstrated varied levels of implementation fidelity indicating need for differential implementation strategies. Use of tiered supports may allow limited time available for implementation support to be spent with teachers most in need of the support, as opposed to providing standardized supports to all teachers. MTIS may be a promising way to provide practitioners a systematic framework for delivering treatment integrity promotion strategies.

**Reference**

1. Sanetti LMH, Collier-Meek MA. Data-driven delivery of implementation supports in a multi-tiered framework: a pilot study. Psychol Sch. 2015;52:815-28.

## A115 Research community collaboration in observational implementation research: complementary and competing interests in the study of implementation as usual

### Adriana Rodriguez^1^, Anna S. Lau^1^, Lillian Bando^2^, Debbie Innes-Gomberg^3^, Lauren Brookman-Frazee^4^

#### ^**1**^Department of Psychology, University of California, Los Angeles, Los Angeles, CA, USA; ^2^Prevention and Early Intervention Administration Division, Program Support Bureau, Los Angeles County Department of Mental Health, Los Angeles, CA, USA; ^3^Mental Health Services Act Implementation and Outcomes Division, Program Support Bureau, Los Angeles County Department of Mental Health, Los Angeles, CA, USA; ^4^Department of Psychiatry, University of California, San Diego; Child and Adolescent Services Research Center, La Jolla, CA, USA

##### **Correspondence:** Adriana Rodriguez (arodriguez@psych.ucla.edu)

**Background**

Implementation research is dominated by studies of investigator-driven implementation of evidence-based practices (EBPs) in community settings. However, system-driven implementation efforts are an increasingly common context of EBP implementation through policy and fiscal interventions [1]. Research-community partnerships (RCPs) are essential to generating knowledge from these efforts. The purpose of this qualitative study was to describe the process of RCP within a system-driven implementation of multiple EBPs for children and families and to characterize the competing and complementary interests among community partners from a variety of stakeholder types.

**Materials and Methods**

27 interviews were conducted with community stakeholders (system leaders, program managers [PM], therapists) involved in a larger study examining policy change in Los Angeles County which fiscally-mandated the use of selective EBPs. We used the RCP framework as a guiding basis for the codebook, which specifies formation, activities, and sustainability of the RCP. All transcripts were coded using qualitative data analysis software.

**Results**

Findings suggest novel considerations in initial engagement phases of an RCP, given the unique set of potentially competing and complementary interests of different stakeholder groups in Implementation as Usual. Previously identified processes and outcomes of RCPs in earlier models [2] generally applied to the current research context. One exception is that all stakeholders focused more on study benefits to therapist- and system-level implementation outcomes rather than client-level clinical outcomes. Stakeholder motivations and concerns were understandably shaped by the most direct perceived impacts on their work. During the phases of the RCP formation and execution of research activities, additional stakeholder role differences emerged in the perceptions of interpersonal and operational processes. The data also provided clear evidence of the distal outcome of increased capacity for sustained and future research-community collaboration.

**Conclusions**

This study adds to our understanding of the process of engaging multiple stakeholder groups in observational studies of EBP Implementation-as-Usual and begins to elucidate the multiple (and competing) interests in the engagement in, and the outcomes of, observational research. It provides specific direction to implementation and effectiveness researchers on the process of engaging multiple stakeholder groups in the context of system-driven implementation research.

**References**

1. Beidas RS, Adams DR Kratz HE, Jackson K, Berkowitz S, Zinny A, et al. Lessons learned while building a trauma-informed public behavioral health system in the City of Philadelphia. Eval Program Plann. 2016;59:21-32. doi:10.1016/j. evalprogplan.2016.07.004

2. Brookman-Frazee L, Stahmer AC, Lewis K, Feder JD, Reed S. Building a research-community collaborative to improve community care for infants and toddlers at-risk for autism spectrum disorders. J Community Psychol. 2012;40(6):715-34. doi:10.1002/jcop.21501

## A116 Evaluation of a low-cost, web-based, multi-component training for trauma-focused cognitive- behavioral therapy: Short- and long-term training outcomes

### Brigid Marriott, Kristin Hawley, Evelyn Cho, Siena Tugendrajch

#### Department of Psychological Sciences, University of Missouri, Columbia, MO, USA

##### **Correspondence:** Brigid Marriott (bmvv5@mail.missouri.edu)

**Background**

Ongoing training may help close the research-to-practice gap, but effective trainings remain expensive and inaccessible. The current study evaluated a low-cost, multi-component, web-based training for Trauma-Focused Cognitive-Behavioral Therapy (TF-CBT) using a two-arm randomized clinical trial to: 1) evaluate the short- and long-term outcomes and potential public health impact of the training using the Therapist Training Evaluation Outcomes Framework [1] and RE-AIM framework [2], 2) explore clinicians’ perspectives of the training including what was most and least helpful as well as barriers to completing the training, and 3) investigate clinician characteristics as predictors of training completion.

**Materials and Methods**

To address these aims, 163 clinicians from a Practice-Based Research Network were recruited via email and randomized to either an immediate training group (ITG; N=89 assigned) or delayed training group (DTG; N=74 assigned). ITG was offered training immediately, while the DTG waited six months. At 12-months, additional interactive training components were added and offered to both the ITG and DTG, and to additional clinician members (ATG; *N*=33). Clinicians completed web-based pre-training, 6-month, 12-month, and 18-month assessments measuring training completion and satisfaction, knowledge, and use of TF-CBT. A subset of clinicians participated in clinical demonstration interviews assessing fidelity to TF-CBT at 6- (*N*=28), 12- (*N*=8), and 18-months (*N*=7) and in qualitative interviews about the training experience at 6- (*N*=20) and 18-months (*N*=7).

**Results**

Coding of the TF-CBT clinical demonstration interviews and qualitative interviews are underway. Initial findings showed variability in both amount and types of training completed. Some 23% completed no training while over 50% completed at least some training (e.g., viewing online didactics (52.3%), reading manual (51.8%), using toolkit (51.3%)). Clinicians were mostly satisfied with the training components and reported frequently using TF-CBT strategies with their cases. Age (β=.31, p<.01), Behavioral theoretical orientation (β=.20, p=.045), and perceived increased job security by learning an EBP (β=.23, p=.03) significantly predicted amount of training completed.

**Conclusions**

Implications for the use of web-based training to enhance implementation of research supported practices within community mental health care will be discussed.

**References**

1. Decker SE, Jameson MT, Naugle AE. Therapist training in empirically supported treatments: a review of evaluation methods for short-and long-term outcomes. Adm Policy Ment Health. 2011;38:254.

2. Glasgow RE, Vogt TM, Boles SM. Evaluating the public health impact of health promotion interventions: the RE-AIM framework. Am J Public Health. 1999;89:1322-7.

## A117 A comprehensive approach to implementation monitoring of a healthy eating and active living evidence-based intervention with African-American churches

### Heather Brandt^1^, Andrea Gibson^2^, Asa Revels^1^, Venice Haynes^1^, Samira Khan^1^, Marian Botchway^1^, Lisa Davis^1^, Lashonda Williams^2^, James Hebert^1^

#### ^**1**^University of South Carolina, Columbia, SC, USA; ^2^Faith-based African American Communities Empowered for Change, Columbia, SC, USA

##### **Correspondence:** Heather Brandt (hbrandt@sc.edu)

**Background**

Using community-engaged approaches, the purpose of Dissemination and Implementation of a Diet and Activity Community Trial In Churches is to implement an evidence-based diet and physical activity intervention, called Healthy Eating and Active Living in the Spirit (HEALS), which consists of 12 weekly sessions and 9 monthly booster sessions over a 1-year period. Process evaluation involves analyzing how program activities are delivered and the level of quality with which delivery occurs [1,2,3]. Examining implementation processes is critical to optimizing overall impact. The purpose is to describe the comprehensive process being used to conduct process evaluation and implementation monitoring.

**Materials and Methods**

A multi-level approach to monitor HEALS intervention delivery is utilized, including monitoring fidelity, completeness, dose received, reach, recruitment, context, and program modification [1,2,3]. Fidelity is addressed beginning with in-depth training for 18 lay health educator (LHE) mentors who previously delivered the intervention and 91 first- time LHEs representing 28 churches. Mentors and LHEs complete evaluations before and after training, 12-weeks, and 1-year to assess development and retention of key skills, knowledge, and role-specific experiences delivering HEALS. Fidelity checks occur through direct observation to assess performance/quality and to inform technical assistance efforts. Completeness is assessed through weekly forms to describe intervention delivery, identify challenges, and observe. Dose received is assessed by tracking attendance. Reach and recruitment are assessed by tracking number of churches contacted and enrolled and participants recruited, enrolled, and retained. Context is monitored through collecting church-level information on social and physical environment characteristics that may relate to implementation. Program modifications are tracked by personnel. Data review occurs quarterly across type and source throughout the project period.

**Results**

Thus far, fidelity to the intervention has been a challenge due to the delivery format in churches. However, we observed moderate retention of skills and knowledge and acceptable performance across assessment points among mentors and LHEs who deliver the program. Observations have used to inform technical assistance activities, and church information was used to better understand the intervention environment. Refinements to the intervention delivery process were made based on a comprehensive approach to implementation monitoring.

**Conclusion**

Implementing LHE-delivered HEALS intervention establishes a pipeline for sustainability by increasing agency for delivery, and careful monitoring is needed. Results have led to changes to implementation and are used to enhance the dissemination of the intervention. A major challenge has been capacity to utilize fully the products of an extensive and comprehensive approach to process evaluation and implementation monitoring.

**References**

1. Durlak JA, DuPre EP. Implementation matters: a review of research on the influence of implementation on program outcomes and the factors affecting implementation. Am J Community Psychol. 2008 ;41(3-4):327-50.

2. Saunders RP. Implementation monitoring and process evaluation. Los Angeles, CA: Sage Publications; 2015.

3. Patton M. Essential of utilization-focused evaluation. Thousand Oaks, CA: Sage Publications; 2012.

## A118 Improving substance use interventions in HIV care in the public healthcare system: Understanding provider needs

### Nicole Ennis-Whitehead^1^, Natalie Kelso-Chichetto^2^, Robert Cook^2^

#### ^1^Department of Clinical and Health Psychology, University of Florida, Gainesville, FL, USA; ^2^Department of Epidemiology, University of Florida, Gainesville, FL, USA

##### **Correspondence:** Nicole Ennis-Whitehead (nwhitehead@phhp.ufl.edu)

**Background**

Substance use interventions have lagged in moving from the bench (research labs) to the community (real-world settings). Therefore, NIDA and the Office of AIDS Research have prioritized research that promotes the uptake of evidence-based interventions into real-world settings. In order to provide effective patient-centered care, effective interventions need to reach those in need. This delay is a vital concern for those with HIV because substance misuse is a common problem that requires large-scale intervention. Extant literature indicates that 20-50% of those with HIV misuse alcohol, illicit drugs, or prescription medications [1,2]. To encourage the community to practice evidence-based substance use intervention routinely, we must understand the best methods of implementation.

**Materials and Methods:**

In 2016, we recruited 14 HIV providers in Florida who provide HIV care in the Alachua and Hillsborough county health departments and the Jackson Memorial public healthcare system in Miami. Participants completed 30-minute structured qualitative interviews that consisted of 6 open ended questions and probes to assess current provider behavior and perceptions of best practices in the field for addressing substance use, mental health, and routine primary care management. Interviews were audio taped and transcribed. NVivo was used to conduct thematic analysis. Themes identified were further refined through alignment with the Consolidated Framework of Implementation Research (CFIR).

**Results**

Preliminary findings identified relevant inner setting themes and characteristic of individual themes that influence implementation of substance use interventions in public healthcare systems. Inner setting themes identified by most providers included: structural characteristics, networks and communication, organizational culture, and availability of resources. Specifically, providers identified the need to focus on HIV care in the clinic setting, limited time to provide additional care outside of medical treatment due to organizational norms/practices, and preference for networking and communication with substance abuse specialists. Additionally, providers varied on knowledge and beliefs about interventions, self-efficacy (i.e., how effective they can be at eliciting change in patients) and individual stage of change (i.e., their level of interest in incorporating substance use interventions into clinic practice).

**Conclusion**

Providers are fundamental agents of change that need to be incorporated in order to facilitate implementation of substance use interventions in public healthcare systems for HIV-positive patients. Therefore, the next step is to develop an intervention protocol that addresses identified themes.

**References**

1. Durvasula R, Miller TR. Substance abuse treatment in persons with HIV/AIDS: challenges in managing triple diagnosis. Behav Med. 2014;40(2):43-52. doi:10.1080/08964289.2013.866540

2. Merlin J, Westfall A, Raper J, Zinski A, Norton WE, Wilig JH, et al Pain, mood, and substance abuse in HIV: implications for clinic visit utilization, antiretroviral therapy adherence and virologic failure. J Acquir Immue Defic Syndr, 2012;61:164-70.

## A119 Implementation stuck point: The effect of added psychotherapy trainings on a multifaceted academic detailing intervention to improve rural PTSD care

### Nancy C. Bernardy^1^, Macgregor Montano^2^, Kathleen Sherrieb^1^

#### ^**1**^National Center for PTSD, US Department of Veterans Affairs, White River Junction, VT, USA; ^2^Veterans Affairs Medical Center, White River Junction, VT, USA

##### **Correspondence:** Nancy C. Bernardy (macmontano@gmail.com)

**Background**

A gap exists between treatments rendered and treatments recommended by clinical guidelines for posttraumatic stress disorder (PTSD) and insomnia [1-3]. More guideline-discordant prescribing practices have been reported in rural areas and such practices are related to poor outcomes in veterans with PTSD, including overdose and suicide-related behavior [4-6]. Limited access to evidence-based psychotherapies is a common barrier to optimal care [7]. Rural areas are plagued by chronic mental health service and provider shortages [8]. Coupling an academic detailing campaign focused on de-prescribing of harmful medications with the provision of trainings in beneficial alternative behavioral treatments may increase implementation success [9,10]. The authors examine the effect of psychotherapy trainings on an educational outreach intervention to improve care delivered to veterans with PTSD in rural outpatient clinics.

**Materials and Methods**

Department of Veterans Affairs data tools and qualitative provider surveys provide information on treatment trends in veterans with PTSD treated at the White River Junction VA Medical Center (WRJ VA) and seven affiliated outpatient clinics in Vermont and New Hampshire. Individualized academic detailing visits and five psychotherapy trainings were provided with the aim of increasing guideline-concordant care. Surveys focused on the impact of trainings offered in cognitive behavioral therapy for insomnia (CBT-I) for individual and group, brief cognitive behavioral therapy for chronic pain (CBT-CP), present-centered therapy for PTSD (PCT), and Mindfulness-Based Cognitive Behavioral Therapy (MB-CBT). Training effect on practitioner ability, motivation, and opportunity to practice was collected [11]. Concurrent prescribing trends of benzodiazepines, non-benzodiazepine sedative hypnotics, off-label antipsychotics, and prazosin were collected for 3 years (2014 to 2017).

**Results**

Prescribing rates and psychology training effects in eight outpatient clinics will be reported and compared. Clinic and clinician characteristics will be examined to determine how local conditions influence implementation of evidence-based practices. Analysis will yield information on the impact of psychotherapy trainings on an academic detailing intervention to improve the care of rural Veterans with PTSD.

**Conclusions**

Findings may help improve rural PTSD care.

**References**

1. Bernardy NC, Lund BC, Alexander B, Friedman MJ. Prescribing trends in veterans with posttraumatic stress disorder. J Clin Psychiatry. 2012;73(3):297-303.

2. Abrams TE, Lund BC, Bernardy NC, Friedman MJ. Aligning clinical practice to PTSD treatment guidelines: medication prescribing by provider type. Psychiatr Serv. 2013; 64(2):142-8.

3. Rosen, CS, Matthieu, MM, Wiltsey Stirman S, Cook JM, Landes S, Bernardy NC, et al. A review of studies on the system-wide implementation of evidence-based psychotherapies for posttraumatic stress disorder in the Veterans Health Administration. Adm Policy Ment Health. 2016;43(6):957-77.

4. Lund BC, Bernardy NC, Alexander B, Friedman MJ. Declining benzodiazepine use in Veterans with Posttraumatic Stress Disorder. J Clin Psychiatry. 2012;73:292-6.

5. Bernardy NC, Lund BC, Alexander B, Friedman MJ. Increased polysedative use in veterans with posttraumatic stress disorder. Pain Med. 2014;15(7):1083-90.

6. Collett GA, Song K, Jaramillo CA, Potter JS, Finley EP, Pugh MJ. Prevalence of central nervous system poly- pharmacy and associations with overdose and suicide-related behaviors in Iraq and Afghanistan war veterans in VA Care 2010-2011. Drugs Real World Outcomes. 2016;3(1):45-52.

7. Barnett ER, Bernardy NC, Jenkyn AB, Parker LE, Lund BC, Alexander B, Friedman MJ. Prescribing clinicians’ perspectives on evidence-based psychotherapy for posttraumatic stress disorder. Behav Sci. 2014;4(4):410-22.

8. Thomas KC, Ellis AR, Konrad TR, Holzer CE, Morrissey JP. County-level estimates of mental health professional shortage in the United States. Psychiatr Serv. 2009;60(10):1323-8.

9. Prasad V and Ioannidis JPA. Evidence-based de-implementation for contradicted, unproven, and aspiring healthcare practices. Implement Sci. 2014;9:1.

10. O’Brien MA, Rogers S, Jamtvedt G, Oxman AD, Odgaard-Jensen J, Kristoffersen DT, Forsetlund L, Bainbridge D, Freemantle N, Davis D, Haynes RB, Harvey E. Educational outreach visits: effects on professional practice and health care outcomes. Cochrane Database Syst Rev. 2007;(4):CD000409.

11. Rousseau DM, Gunia BC. Evidence-based practice: The psychology of EBP implementation. Annu Rev Psychol 2016;67:667-92.

## A120 Characterizing the use of specific delivery strategies in cognitive behavioral therapy for anxious youth over time and across therapy setting

### Julia Cox, A. Ali, Michael Southam-Gerow, Bryce McLeod

#### Department of Psychology, Virginia Commonwealth University, Richmond, VA, USA

##### **Correspondence:** Julia Cox (coxjr4@vcu.edu)

**Background**

Cognitive-behavioral therapy (CBT) for youth anxiety often features specific skills that therapists teach to clients (e.g., relaxation). As attention to measuring treatment integrity—a multidimensional construct that typically comprises adherence, competence, differentiation, and relationship factors—increases, there is value in separating the content of the intervention from the method of delivery. This is partly because treatment manuals may prescribe different delivery strategies for the same skill. For example, when teaching relaxation, therapists may employ didactic and modeling strategies early in treatment and turn to rehearsal later as the child’s mastery of the skill increases. Therapists’ use of delivery strategies may also depend on their comfort with and training in such approaches (e.g., rehearsal of exposure tasks). Further, therapists’ use of delivery strategies may correlate with other therapy process factors, including use of specific interventions and relational factors.

**Results**

The extent to which clinicians used specific delivery techniques differed between research and practice settings for all items except Self-Disclosure such that clinicians in research settings had significantly higher scores. Collaborative Teaching and Rehearsal were the most extensively used delivery methods across both trials. Delivery items significantly correlated with several CBAY-A items: passive approaches (i.e., Didactic Teaching, Modeling) negatively correlated with exposure-focused items, and active approaches (i.e., Collaborative Teaching, Rehearsal) positively correlated with exposure-focused items. Finally, active approaches were significantly positively correlated with child involvement scores.

**Materials and Methods**

The main goal of this study is to examine how therapists in different treatment settings use specific delivery strategies over time while providing individual CBT for anxious youth. Data were drawn from two randomized controlled trials (RCTs) in which therapists providing individual CBT for youth anxiety, treated a diverse sample of children, aged 8–15: (RCT1) in a university laboratory (n=51; 44% female, 85% white), and (RCT2) in community clinics (n=17; 56% female, 38% white). Therapist adherence using six specific delivery strategies of CBT for youth anxiety—didactic (ICC=0.73), collaborative teaching (ICC=0.69), modeling (ICC=0.74), rehearsal (ICC=0.88), coaching (ICC=0.43), and self-disclosure (ICC=0.71)—throughout treatment was double coded on a 7-point extensiveness scale using the Cognitive-Behavioral Treatment for Anxiety in Youth Adherence Scale. A total of 744 sessions were coded (RCT1=532; RCT2=212). We will model the use of specific treatment delivery strategies over the course of treatment and explore setting-level differences.

**Conclusions**

Findings may help inform future therapy process models and dissemination efforts (e.g., improved training, supervision, consultation).

## A121 Capturing complexity: A structured reflection method for implementation research in complex adaptive systems

### Erin Finley^4^, Alexis Huynh^2^, Melissa Farmer^2^, Bevanne Bean-Mayberry^3^, Tannaz Moin^3^, Sabine Oishi^2^, Jessica Zuchowski^4^, Karen Dyer^4^, Holly Lanham^1^, Luci Leykum^1^, Alison Hamilton^3^

#### ^**1**^South Texas Veterans Health Care System, UT Health Science Center, San Antonio, TX, USA; ^2^VA Greater Los Angeles Health System, HSR&D Center for the Study of Healthcare Innovation, Implementation & Policy, Los Angeles, CA, USA; ^3^VA Greater Los Angeles Health System, HSR&D Center for the Study of Healthcare Innovation, Implementation & Policy, David Geffen School of Medicine at UCLA, Los Angeles, CA, USA; ^4^HSR&D Center for the Study of Healthcare Innovation, Implementation & Policy, Los Angeles, CA, USA

##### **Correspondence:** Erin Finley (finleye@uthscsa.edu)

**Background**

Although complexity science has been argued to illuminate a variety of phenomena essential to successful implementation, including emergence, sensemaking, self-organization, and interdependencies [1], challenges associated with documenting these characteristics of complex adaptive systems (CAS) amid busy clinical care settings [2] remain a significant barrier to understanding their role in implementation. Development of methods to support feasible observation of CAS phenomena becomes ever more important as implementation increasingly integrates multi-strategy approaches occurring across large healthcare systems.

**Materials and Methods**

The VA-funded EMPOWER QUERI is conducting three projects to implement innovative care models in VA women’s health for high-priority health conditions – prediabetes, cardiovascular risk, and mental health – following an adapted version of the Replicating Effective Programs (REP) framework enhanced with complexity theory. Drawing on tenets of rapid qualitative research, we developed an innovative structured reflection method to facilitate observations of CAS phenomena occurring across multiple sites for the three EMPOWER projects. The method was reviewed by a panel of implementation and complexity science experts and clinical staff for content and feasibility and iteratively refined during the initial six months of data collection. Site PIs regularly participate in brief (20-30 minute) telephone interviews at monthly or bi-monthly intervals. Questions inquire about main actors, activities, and challenges, as well as recent changes to the intervention, implementation plan, or local/national context. Interview notes are coded to reflect key project activities and CAS phenomena.

**Results**

Eighteen structured reflections completed during the initial study period indicate this method provides a feasible strategy for documenting pre-implementation and implementation activities and events on a periodic basis without placing undue burden on research or clinical staff. Coded reflections exhibit characteristics of CAS including emergence (e.g., adaptations, unexpected events), sensemaking and self-organization occurring at the level of projects and individual sites, interdependencies (e.g., among staff and stakeholders), and nonlinear impacts (e.g., the outsized role of leadership support). This method offers a user-friendly means to document key processes, events, and CAS phenomena occurring as part of research and implementation.

**Conclusions**

Few methods exist to aid in operationalizing complexity science in implementation research, and those that do often require significant investment and/or burden for staff and participants, reducing their value for use in multi-site implementation studies. This structured reflection method shows potential as a feasible and low-burden approach for documenting CAS phenomena in multi-pronged interventions across multiple sites.

**References**

1. Lanham HJ, Leykum LK, Taylor BS, McCannon CJ, Lindberg C, Lester RT. How complexity science can inform scale-up and spread in health care: Understanding the role of self-organization in variation across local contexts. Soc Sci Med. 2013;93:194–202.

2. Chambers DA, Glasgow RE, Stange KC. The dynamic sustainability framework: addressing the paradox of sustainment amid ongoing change. Implement. Sci. 2013;8:117.

## A122 Collaborative goal-setting intervention for clinical supervision: A mixed-methods pilot of a fidelity intervention

### Alan McGuire^1^, Tom Bartholomew^2^, Mary Blaney-Rychener^3^, Adrienne Anderson^1^, Sarah Bauer^1^, Dominique White^1^, Michelle Salyers^1^

#### ^**1**^Indiana University Purdue University Indianapolis, Indianapolis, IN, USA; ^2^Rutgers University, Newark, NJ, USA; ^3^Thresholds, Inc., Georgetown, DE, USA

##### **Correspondence:** Alan McGuire (abmcguir@iupui.edu)

**Background**

The current study piloted a mixed-method intervention to increase fidelity to Illness Management and Recovery (IMR), an evidence-based self-management program for people with severe mental illness [1]. The intervention included audit-and-feedback [2] and four sessions of collaborative goal-setting supervision [3].

**Materials and Methods**

A convenience sample of IMR providers submitted audio-recordings during two, three-month periods (intervention and observation). Fidelity of these sessions was rated using the IMR Treatment Integrity Scale (IT-IS) [4]. Participants completed self-report measures of IMR fidelity importance and confidence as well as demographics. Qualitative data was collected from providers to better understand the reception of the intervention.

**Results**

Hypothesis 1, that fidelity importance and confidence would be positively associated with baseline fidelity, was not supported. Hypothesis 2, that fidelity would increase across the intervention phase, was not supported. Exploratory analysis indicated that baseline fidelity importance was negatively associated with fidelity improvement (r = -.61., p < .001). A repeated-measures ANOVA indicated a main effect for time (F = 6.1, d.f. = 3, p = .001) and a time by importance interaction (F = 6.1, d.f. = 3, p = .001). A plot illustrated that IT-IS for participants with low baseline importance improved more than other participants. A mediation model was tested, in which importance at follow-up mediated the relationship between importance at baseline and change in IT-IS. This model was not supported. Qualitative analyses indicated a positive reception of the intervention and its tolerability. The majority of interviewees indicated they preferred the intervention supervision to their regular supervision. Suggested improvements included increasing the frequency and number of supervisions sessions and decreasing time between recording an IMR session and the corresponding supervision.

**Conclusions**

Results did not support the effectiveness of audit-and-feedback and collaborative goal-setting supervision in improving IMR fidelity. Several possibilities for these results exist. Participants had high baseline fidelity scores and, were likely more motivated to focus on fidelity than the average provider; therefore, participant selection bias may have limited room for improvement. Also, many participants came from settings with preexisting, robust IMR-focused supervision. The emergent interaction between baseline fidelity importance and improvement in fidelity requires further exploration. Analyses did not support the notion that the intervention increased perceived importance, which in turn increased fidelity. Moreover, fidelity improvements were not sustained following the intervention period. It appears likely that improvements were driven by controlled motivation (e.g., worry of embarrassment for low fidelity scores) rather than autonomous motivation (viewing fidelity as good clinical practice) [5].

**References**

1. McGuire AB, Kukla M, Green A, Gilbride D, Mueser KT, Salyers MP. Illness management and recovery: A review of the literature. Psychiatr Serv. 2014;65(2):171-9.

2. Ivers NM Grimshaw JM, Jamtvedt G, Flottorp S, O'Brien MA, French SD, Young J, Odgaard-Jensen J. Growing literature, stagnant science? Systematic review, meta-regression and cumulative analysis of audit and feedback interventions in health care. J Gen Intern Med. 2014; 29(11):534-41.

3. Milne D. The systematic review as an empirical approach to improving CBT supervision. Int J Cogn Therapy, 2010;3:278-94.

4. McGuire AB, Stull LG, Mueser KT, Santos M, Mook A, Rose N, Tunze C, White LM, Salyers MP. Development and reliability of a measure of clinician competence in providing illness management and recovery. Psychiatr Serv. 2012;63(8):772-8.

5. Sheldon, K.M. and A.J. Elliot, Not all personal goals are personal: comparing autonomous and controlled reasons for goals as predictors of effort and attainment*.* J Pers Soc Psychol, 1998. 24(5): 546.

## A123 Development and validation of a fidelity measure for cognitive-behavioral therapy with youth: The TPOCS-self-reported therapist intervention fidelity for youth

### Emily Becker-Haimes^1^, Bryce McLeod^2^, Sonja Schoenwald^3^, Shannon Dorsey^4^, Aaron Hogue^5^, Adina Lieberman^1^, Courtney Gregor^1^, Kelly Zentgraf^1^, Steven Marcus^1^, David Mandell^1^, Judy Shea^1^, Rinad Beidas^1^

#### ^**1**^University of Pennsylvania, Philadelphia, PA, USA; ^2^Virginia Commonwealth University, Richmond, VA, USA; ^3^Medical University of South Carolina, Charleston, SC, USA; ^4^University of Washington, Seattle, WA, USA; ^5^The National Center on Addiction and Substance Abuse, New York, NY, USA

##### **Correspondence:** Emily Becker-Haimes (embecker@upenn.edu)

**Background**

Accurate and feasible methods to assess clinician fidelity to cognitive behavioral therapy (CBT) are needed to monitor CBT implementation efficacy across settings. Self-report, in which therapists self-rate their fidelity to CBT via brief questionnaire [1], is a low burden assessment method, making it attractive for use in community mental health. However, current self-report measures face several challenges: 1) existing self-report measures are lengthy and/or contain, technical language that interferes with ability to accurately self-rate, and 2) no scale maps directly on to existing observational coding systems of therapist behavior. To address this, we developed a self-report measure of therapist fidelity to CBT for youth [2] that parallels the CBT intervention items on the Therapy Process Observation Coding Scale-Revised Strategies (TPOCS-RS), a gold standard observational coding system for therapist behavior [3].

**Materials and Methods**

The TPOCS-Self-Reported Therapist Intervention Fidelity for Youth (TPOCS-SeRTIFY) underwent an iterative, rigorous development process that included review by 4 experts in fidelity measurement and cognitive interviews with 8 community mental health clinicians. To circumvent two challenges of self-report, difficulty understanding items and lack of training in how to judge behavior, we (a) provided an operational definition for each item on the TPOCS-SeRTIFY, and (b) developed a brief training session and companion manual that includes sample vignettes of behaviors and information about how those vignettes should be rated. Two independent experts in CBT reviewed the finalized measure and rated how similar items on the TPOCS-SERTIFY were to those outlined in the TPOCS-RS coding manual. Initial psychometric data is being collected in a large sample of community mental health clinicians (data collection underway, n = 33 to date, 200 anticipated; anticipated completion: June, 2017). Participants will complete the TPOCS-SeRTIFY as part of a larger assessment battery, including the Therapy Procedures Checklist (TPC) [4], a commonly used and validated measure of clinician use of therapeutic strategies.

**Results**

The final measure consisted of 12 CBT interventions that map on to the TPOCS-RS CBT items and 4 additional items assessing therapist competence. CBT experts rated TPOCS-SeRTIFY as highly concordant with the TPOCS-RS coding manual (average similarity ratings across items was 6.5 out of 7). We will present results of exploratory factor analysis and initial validity by examining correlations with CBT items on the TPC.

**Conclusions**

The TPOCS-SeRTIFY has the potential to fill an important measurement gap in youth mental health. Implications for fidelity measurement and the TPOCS-SeRTIFY’s potential for widespread use will be discussed.

**References**

1. Schoenwald SK, Garland AF. A review of treatment adherence measurement methods. Psychol Assess. 2013;25:146-56.

2. Beidas RS, Maclean JC, Fishman J, Dorsey S, Schoenwald SK, Mandell DS, et al. A randomized trial to identify accurate and cost-effective fidelity measurement methods for cognitive-behavioral therapy: project FACTS study protocol. BMC Psychiatry. 2016;16(1):323.

3. McLeod BD, Smith MM, Southam-Gerow MA, Weisz JR, Kendall PC. Measuring treatment differentiation for implementation research: the Therapy Process Observational Coding System for Child Psychotherapy Revised Strategies Scale. Psychol Assess. 2015;*27*(1):314.

4. Weersing VR, Weisz JR, Donenberg GR. Development of the Therapy Procedures Checklist: a therapist-report measure of technique use in child and adolescent treatment. J Clin Child Adolesc Psychol. 2002;31(2):168-80.

## A124 Testing un-learning and substitution strategies to de-implement antipsychotics in nursing homes

### Christian Helfrich, Megan McCullough

#### Department of Veterans Affairs, Seattle, WA, USA

##### **Correspondence:** Christian Helfrich (megan.mccullough@va.gov)

**Background**

Medical overuse (i.e., treatment that provides no benefit and/or harm) represents 10%-46% of care depending on setting and practice. Use of antipsychotic medications to manage behavioral and psychological symptoms of dementia (BPSD) in nursing homes is an example of overuse. Despite limited evidence of efficacy and significant evidence of risks including mortality, 1 in 4 residents living with dementia in the Veterans Health Administration (VHA) Community Living Centers (CLCs—i.e., nursing homes) is prescribed antipsychotics. We developed a planned action model founded on the utility of two distinct, synergistic processes: 1) unlearning; and 2) substitution. Building on prior work, our objective is to tailor and operationalize unlearning and substitution strategies in the de- implementation of antipsychotic use in 6 VHA CLCs.

**Materials and Methods**

Via a stepped-wedge design, this project tests unlearning and substitution strategies. Academic detailing (unlearning) promotes change in prescribing habits through educational outreach on limited effectiveness and adverse effects of antipsychotics. The WeCareAdvisor™ is an on-line tool for use by frontline CLC staff that contains the DICE (Describe, Investigate, Create, Evaluate) approach for assessment and management of BPSD via an ecobiopsychosocial model (substitution). The tool guides staff through assessing CLC residents’ symptoms/ context and prompts them with behavioral and environmental interventions to address BPSD. This project involves a mixed-methods evaluation of the simultaneous implementation of these two strategies, including an interrupted time series analysis of changes in prescribing and a quantitative and qualitative evaluation of the process of tailoring and operationalizing these strategies to determine the impact of tailoring on outcomes.

**Results**

Barriers to reducing antipsychotic use will be identified as will the complicated roles various staff play in prescribing behavior. Concrete guidance on operationalizing and measuring unlearning and substitution strategies in nursing home settings will be provided. Methods for identifying how unlearning and substitution strategies are tailored and implemented will be described. Additionally, unintended consequences of the strategies will be catalogued and evaluated for their impact on overuse.

**Conclusions**

We propose concrete ideas on operationalizing and testing unlearning and substitution strategies. Lessons about the unintended consequences of implementing these strategies to lessen overuse will also add to the practical and conceptual knowledge about these types of implementation techniques.

## A125 Two-tiered external facilitation: An implementation strategy for successful uptake of a new teleSleep program

### Nicholas Rattray^1^, Teresa Damush^1^, Edward Miech^1^, Barbara Homoya^1^, Jennifer Myers^1^, Jared Ferguson^1^, Dawn Bravata^1^

#### ^**1**^Roudebush VA Medical Center, Indianapolis, IN, USA

##### **Correspondence:** Nicholas Rattray (nrattray@iupui.edu)

**Background**

While facilitation is a widely recognized implementation strategy in quality improvement projects, less is known about how multiple facilitators work together in combination to implement programs that span services and disciplines [1]. We applied the iPARIHS framework [2,3] to a prospective, in-depth case evaluation of two external facilitators that worked together as a dyad to implement a new, complex TeleSleep program at a VA Medical Center.

**Materials and Methods**

Data were collected prospectively from multiple sources including brief interviews with key informants; tracking spreadsheets completed by external facilitators that documented tasks completed and stakeholder communications; and program meeting notes. A trained team coded and analyzed the data for emergent themes related to facilitation.

**Results**

A two-tiered external facilitation strategy was crucial to the implementation success of the new TeleSleep program. At the executive level, an external facilitator sought endorsement from key stakeholders including: local leadership by securing resources, service chiefs for staff participation and work flow redesign; and the vendor for modification to the remote monitoring devices. The facilitator also planned and designed the program components in collaboration with executive level stakeholders. At the coordinator level, a second external facilitator provided guidance to front line stakeholders including: boundary spanning activities to bridge boundaries between staff and services; training staff on the program elements; creating tools for program implementation; serving as a neutral expert to answer questions and assist with problem-solving. Both levels of external facilitators were involved in monitoring implementation progress and feeding back to the front line and executive level stakeholders. The external facilitators were critical for patching the networks and communications during this complex innovation as two services had to collaborate and provide hand offs for the first time. Their role fluctuated between a holistic-orientated during the pre- and post-implementation phases to a more task-oriented role during active implementation. To sustain this innovation, key informants across the organization further adopted the program to implement through only one of the clinical services and leadership invested into permanent program adoption.

**Conclusions**

Two-tiered external facilitation can be an implementation strategy for the successful implementation of innovative and novel complex programs. External facilitators at the senior and coordinator levels can assist local stakeholders to overcome barriers by providing neutral expertise to guide the organizational changes during initial implementation. When an organization makes further adaptations to sustain the program, external facilitators can serve in a consultant manner to local champions.

**References**

1. Rycroft-Malone J, Seers K, Chandler J, Hawkes CA, Crichton N, Allen C, et al. The role of evidence, context, and facilitation in an implementation trial: implications for the development of the PARIHS framework. Implement Sci. 2013;8(1):28.

2. Stetler C, Damshroder L, Helfrich C, Hagedorn H. A Guide for applying a revised version of the PARIHS frame work for implementation. Implement Sci. 2011;6(1):99.

3. Harvey G, Kitson A. PARIHS revisited: from heuristic to integrated framework for the successful implementation of knowledge into practice. Implement Sci. 2016;11(1):33.

## A126 Enhancing evidence-based rehabilitation through communication and colocalization: Implementation experience at the Shirley Ryan AbilityLab (formerly Rehabilitation Institute of Chicago)

### Miriam R. Rafferty^1,2^, Justin D. Smith^3^, Mariah K. Meachum^3^, Melissa Briody^2^, Carmen E. Capo-Lugo^1^, Juan A. Villamar^3^, Piper Hansen^2^, Jamie L. O’Connor^1^, Allen W. Heinemann^2^, Richard L. Lieber^2^, C. Hendricks Brown^3^

#### ^**1**^Northwestern University Center for Education in Health Science, Chicago, IL, USA; ^2^Shirley Ryan AbilityLab, Chicago, IL, USA; ^3^Northwestern University Feinberg School of Medicine Center for Prevention Implementation Methodology (ce-PIM), Chicago, IL, USA

##### **Correspondence:** Miriam R. Rafferty (miriamrafferty@northwestern.edu)

**Background**

Fostering a research culture in an interdisciplinary rehabilitation setting presents many challenges related to collaboration and communication [1,2]. When the new Shirley Ryan AbilityLab facility was opened in 2017, it was designed to increase collaboration and communication between clinicians and researchers by integrating, or colocalizing, research labs in clinical space. The purpose of this study was to document the implementation of this novel AbilityLab Model of Care in domains of organizational culture, leadership, evidence-based practice, and communication.

**Materials and Methods**

A survey was emailed to 1205 clinicians (physicians, nurses, allied health professionals), researchers, support staff, and leadership two months prior to the transition to the new facility using the Research Electronic Data Capture secure survey platform [3]. The survey included domains adapted from the Organizational Change Recipients’ Beliefs Scale [4], the Implementation Leadership Scale [5], Evidence Based Practice Attitudes Scale [6], and the Evidence-Based Practice Questionnaire [7]. Several questions regarding communication attitudes and behaviors were added. The survey will be administered again following the transition to assess changes in employees’ attitudes and behaviors related to implementation.

**Results**

There was an overall 65% response rate to the baseline survey. Response rates by participant categories were: 63% clinicians, 57% researchers, 64% support staff, and 92% leaders. 5% of respondents identified dual clinical and research roles. At baseline, self-identified leaders reported the most familiarity with the AbilityLab Model of Care. Organizational change data indicated that leaders were most confident with the transition, while researchers were least likely to embrace the change. Researchers were also least likely to report that their leaders removed implementation obstacles. Eagerness to try new techniques and to research new clinical questions were similarly strong for clinicians and researchers. However, prior to the transition clinicians and researchers never or rarely communicated with each other, and clinicians expressed less confidence in their ability to communicate with researchers. Clinicians also endorsed the importance of two-way communication to a lesser extent than researchers and leaders.

**Conclusions**

Prior to the transition, areas conducive to implementation included high levels of clinician and researcher eagerness to engage with each other to influence practice and research. Potential implementation strategies include engaging more research champions to assist with the transition and providing enhanced opportunities for communication between clinicians and researchers. Quality improvement efforts and changes over time in employee attitudes and behaviors will be tracked to document implementation of the novel AbilityLab Model of Care.

**References**

1. Jones ML, Cifu DX, Backus D, Sisto SA. Instilling a research culture in an applied clinical setting. Arch Phys Med Rehabil. 2013;94(1 Suppl):S49-54.

2. Blevins D, Farmer MS, Edlund C, Sullivan G, Kirchner JE. Collaborative research between clinicians and researchers: a multiple case study of implementation. Implement Sci. 2010;5:76.

3. Harris PA, Taylor R, Thielke R, Payne J, Gonzalez N, Conde JG. Research electronic data capture (REDCap)--a metadata-driven methodology and workflow process for providing translational research informatics support. J Biomed Inform. 2009;42(2):377-81.

4. Armenakis AA, Bernerth JB, Pitts JP, Walker HJ. Organizational Change Recipients Beliefs Scale: development of an assessment instrument. J Appl Behav Sci. 2007;42:481-505.

5. Aarons GA, Ehrhart MG, Farahnak LR. The Implementation Leadership Scale (ILS): development of a brief measure of unit level implementation leadership. Implement Sci. 2014;9(1):45.

6. Aarons GA. Mental health provider attitudes toward adoption of evidence-based practice: the Evidence-Based Practice Attitude Scale (EBPAS). Ment Health Serv Res. 2004;6(2):61-74.

7. Upton D, Upton P. Development of an evidence-based practice questionnaire for nurses. J Adv Nurs. 2006;53(4):454-8.

## A127 Attitudes towards evidence-based practice: Evaluating the impact of provider attitudes on implementing a comprehensive CBT rollout training for frontline clinicians

### Hollie Granato (hollie.granato@gmail.com)

#### Harbor-UCLA Medical Center, Harbor City, CA, USA

**Background**

Mental health service providers implement frontline treatment for adults and children in the United States, yet much of the services being offered in clinics across the country are not based in current evidence for best practices [1]. Subsequently, growing efforts are being made to improve provider adoption of evidence- based practice (EBP), an approach to treatment that is characterized by the explicit and judicious use of the best available evidence for making clinical decisions (Sackett, 2000). However, numerous barriers remain to training providers in EBPs – first and foremost being provider attitudes towards using EBPs. Therefore, the goal of this study was to evaluate provider attitudes towards EBPs within a multi-year rollout of a widespread and well- documented EBP – Cognitive Behavioral Therapy (CBT) - to approximately 1500 mental health front line service providers living in the greater Los Angeles area.

**Materials and Methods**

All participants in this study were mental health providers who opted into the training, completing a previously validated measure of EBP attitudes, the Evidence Based Practice Attitudes Scale (EBPAS; Aarons, 2004) at both baseline and follow-up. The training required that participants attended a 3-day intensive and interactive training in CBT, engage in 16 weeks of one hour consultation calls with an expert in CBT, as well as attend a final “booster” training day at the end of the 16 weeks. In order to successfully complete the training, participants needed to score within a specific range on the Cognitive Therapy Rating Scale (CTRS; Young & Beck, 1980) on two of three audio taped sessions.

**Results**

The hypothesis that attitudes towards EBPs would significantly increase from pre- to post- test was tested using paired sample t-tests and fully supported. For all further hypotheses, data is still currently being aggregated and analyzed. We will evaluate the hypothesis that more negative attitudes towards EBPs at pre-training will predict drop out from the training. Finally, we will evaluate the hypothesis that a higher number of previous years in the mental health provider field prior to engaging in the training will predict more negative EBP attitudes at pre-training as well as moderate adherence to the treatment based on Cognitive Therapy Rating Scores. All data will be analyzed using SPSS.

**Conclusions**

This research has important implications for the systematic implementation of EBP training and informs how addressing attitudes towards EBPs could impact training success among providers.

**Reference**

1. Hoagwood K, Olin SS. The NIMH blueprint for change report: research priorities in child and adolescent mental health. J Am Acad Child Adolesc Psychiatr. 2002;41(7):760-7.

## A128 Feeling the pinch in community mental health: How perceived financial strain in clinicians affects turnover

### Danielle Adams^1,2^, Nathaniel Williams^3^, Emily Becker Haimes^1^, Laura Skriner^4^, Lauren Shaffer^1^, Kathryn Dewitt^1^, Arthur Evans^5^, Rinad Beidas^1^

#### ^1^Center for Mental Health Policy and Services Research, University of Pennsylvania, Philadelphia, PA, USA; ^2^School of Social Service Administration, University of Chicago, Chicago, IL, USA; ^3^School of Social Work, Boise State University, Boise, ID, USA; ^4^Weill Cornell Medicine, New York-Presbyterian Hospital—Westchester Division, White Plains, NY, USA; ^5^Department of Behavioral Health and Intellectual disAbility Services, Philadelphia, PA, USA

##### **Correspondence:** Danielle Adams (daniadams@uchicago.edu)

**Background**

Clinician turnover is a major barrier to evidence-based practice (EBP) implementation efforts in community mental health given that 30-60% of clinicians leave their organization annually [1]. Identifying predictors of clinician turnover in the context of EBP implementation efforts is critical to developing effective interventions to reduce clinician turnover and facilitate implementation. One predictor that has been unexplored to date is financial strain, or when an individual’s real expenses exceed their income, and when one is unable to meet his/her financial responsibilities. This is extremely relevant given the low wages that clinicians earn and the poor fiscal climate in community mental health centers (CMHCs) [2].

**Materials and Methods**

This study is the first to quantitatively explore the relationship between financial strain, EBP initiative participation, and turnover. Our sample included 247 therapists nested within 28 community mental health clinics. CMHCs were situated within a system implementing evidence-based practices (EBPs); 23 organizations were actively implementing EBPs. To assess financial strain, participants completed the InCharge Financial Distress/Financial Well-Being Scale [3], a measure evaluating an individual’s financial state on a continuum ranging from overwhelming financial distress/lowest level of financial well-being to no financial distress/highest level of financial well-being. EBP initiative participation was assessed by self-report. Turnover was assessed one year following initial data collection. Mixed effects logistic regression models examined the impact of financial strain on turnover and whether EBP initiative participation moderated this relationship, controlling for covariates (agency size, clinician race, employment status).

**Results**

Clinicians who perceived greater financial strain were significantly more likely to leave their agency (p < .01). EBP initiative participation moderated this relationship between financial strain and turnover, such that the probability of turnover (denoted as $\widehat{\mathrm{y}}$) was comparable among clinicians who had participated in an EBP initiative regardless of whether they experienced low or high financial strain ($\widehat{\mathrm{y}}$ = .35 vs. $\widehat{\mathrm{y}}$ = .36, respectively). In contrast, among clinicians who did not participate in an EBP initiative, expected probability of turnover was higher among those who experienced high financial strain ($\widehat{\mathrm{y}}$ = .51) compared to those who experienced low financial strain ($\widehat{\mathrm{y}}$ = .23); non- participating, highly-strained clinicians were 2.2 times more likely to turnover.

**Conclusions**

Participation in an EBP initiative may exert a protective effect on the likelihood of turnover among clinicians who are financially strained. As such, reducing financial strain and/or promoting EBP trainings may be both possible avenues of intervention to reduce turnover in CMHC settings.

**References**

1. Mor Barak ME, Nissly JA, Levin A. Antecedents to retention and turnover among child welfare, social work, and other human service employees: what can we learn from past research? A review and metanalysis. Soc Serv Rev. 2001;75(4):625-61.

2. Stewart RE, Adams DR, Mandell DS, Hadley TR, Evans AC, Rubin R, et al. The perfect storm: Collision of the business of mental health and the implementation of evidence-based practices. Psych Serv. 2016;67(2):159-61.

3. Prawitz AD, Garman ET, Sorhaindo B, O’Neill B, Kim J, Drentea P. InCharge financial distress/financial well- being scale: development, administration, and score interpretation. J Financ Counsel Plan. 2006;17(1):1-17.

## A129 The role of organizational factors in implementation outcomes from Project HEAL: A preliminary investigation

### Cheryl Holt^1^, Laundetta Jones^1^, Hongjie Liu^1^, Sherie Lou Santos^1^, Janice Bowie^2^, Jimmie Slade^3^

#### ^**1**^University of Maryland, College Park, MD, USA; ^2^Johns Hopkins University, Baltimore, MD, USA; ^3^Community Ministry of Prince George’s County, Capitol Heights, MD, USA

##### **Correspondence:** Cheryl Holt (cholt14@umd.edu)

**Background**

Project HEAL (Health through Early Awareness and Learning) is an implementation trial that compared two methods of training lay community health advisors (CHAs), Traditional in-person vs. web-based (“Technology”), to conduct evidence-based cancer educational group workshops in African American churches [1]. Organizational factors vary from setting to setting (e.g., between the churches) and may play a role in helping explain implementation outcomes, including why some churches had greater success than others. We report a descriptive analysis of the relationship between organizational/contextual factors and Project HEAL outcomes along the RE- AIM Framework [2].

**Materials and Methods**

Project HEAL CHAs in 14 African American churches delivered a 3-workshop cancer educational series to their church members age 40-75 (N=375). Using multi-level data from CHAs, participants, and study records, we described three aspects of organizational capacity in each church (staffing/space; health promotion experience; external collaborations) and the relationship between these capacity ratings and RE-AIM Framework outcomes. Due to the small sample of churches, Cohen’s d is used to report effect sizes for mean comparisons and correlation coefficient (r) for correlations.

**Results**

Baseline staffing/space scores were negatively associated with intervention reach, calculated as the number of eligible persons enrolled in Project HEAL / total pool of potential eligible individuals in the 14 churches (correlation [r] = -.62, p =0.02). Correlations between capacity scores and implementation outcomes (e.g., time to complete workshop series) varied from -.23 to .33. For intervention efficacy, men from churches with higher staffing/space scores (M = 51.03) were marginally more likely than those from churches with lower staffing/space (M = 48.68) to report having had a prostate specific antigen exam at 24 months (d = .45, p = .08). Capacity scores were not associated with participants’ reports of colonoscopy (d ranged from .06 to .16). Capacity scores were in some cases associated with sustainability outcomes (e.g., ongoing health promotion activities) with effect sizes ranging from d = .09 to 94.

**Conclusions**

Though limited by a sample size of 14 organizations, this descriptive data illustrates how context can be evaluated and may be associated with outcomes along the implementation continuum. Findings suggest that implementation outcomes are not a direct function of church size. Future development of capacity assessment in faith-based organizations and replication with larger samples are next steps. Methodological advances/applications are needed to account for modest sample sizes when the organization is the unit of analysis. Implications for implementation science are discussed.

**References**

1. Glasgow RE, Vogt TM, Boles SM. Evaluating the public health impact of health promotion interventions: the RE-AIM Framework. Am J Public Health, 1999;89(9):1322-7.

2. Holt CL, Tagai E, Scheirer MA, Santos SLZ, Bowie J, Haider M, Slade JL, Wang MQ, Whitehead T. Translating evidence-based interventions for implementation: experiences from Project HEAL in African American churches. Implement Sci. 2014;9:66. doi: 10.1186/1748-5908-9-66.

## A130 Implementation of Dialectical Behavior Therapy in a residential setting: Dissemination and evaluation

### Amber Holbrook^1^, Susan Hunt^2^, Jehan Morsi^1^

#### ^**1**^West Chester University, West Chester, PA, USA; ^2^Resources for Human Development, Inc, Philadelphia, PA, USA

##### **Correspondence:** Amber Holbrook (aholbrook@wcupa.edu)

**Background**

The use of evidence-based practices (EBPs) in social services is gaining momentum as the standard of care. However, many residential settings employ individuals without advanced formal education and training as Direct Service Professionals (DSPs). Typically, these lower-level workers provide the majority of daily care to the more challenged clients, often leading to poorer quality of client care, staff burnout, and high staff turnover rates [1]. The use of EBPs has the potential to mitigate poor client and staff outcomes in such settings when appropriate training can support fidelity to the intervention model. Dialectical Behavior Therapy (DBT) is demonstrated to be an effective intervention for a growing number of behavioral health disorders [2]. However, training is required to successfully implement DBT, and maintenance relies on reinforcement by the program milieu in which it is delivered [3]. Provision of training on EBPs, such as DBT, is important for both quality of client care and workforce development. This paper presents a DBT training delivery model and a process evaluation designed to provide feedback on the implementation of the model. The DBT training initiative sought to create a “DBT-informed program culture” through staff training and program-level consultation in four phases. Results from the first phase of staff training are presented.

**Materials and Methods**

Five residential programs participated in the training initiative from 2013-2015. Staff knowledge retention was measured post training through administration of an 18-question assessment of principles and skills associated with the four DBT modules: emotional regulation, distress tolerance, interpersonal skills, and mindfulness.

**Results**

Fifty-eight staff completed a knowledge retention quiz. Staff were predominantly female (82.8%), African-American (72.7%), and with a mean age of 34.8 (SD=8.4). Many had completed some college (40.4%), with a total of 57.9% of the sample attaining less than a four year degree at the time of training. Mean score was 83.2% with 77.6% of the sample scoring 83.2% or higher.

**Conclusions**

Results of the first phase of the training initiative suggest that it is feasible to train DSPs in the principles of DBT in a cost-effective manner, but attention is required to uneven knowledge retention.

**References**

1. Connor DF, Mcintyre EK, Miller K, Brown C, Bluestone H, Daunais S, et al. Staff retention and turnover in a residential treatment center. Resid Treat Child Youth. 2003;20(3):43-53. doi:10.1300/j007v20n03_04

2. Burroughs T, Somerville J. Utilization of evidenced based Dialectical Behavioral Therapy in assertive community treatment: examining feasibility and challenges. Community Ment Health J. 2012;49(1):25-32. doi:10.1007/s10597-012-9485-2

3. Swales MA, Taylor B, Hibbs RAB. Implementing Dialectical Behaviour Therapy: programme survival in routine healthcare settings. J Ment Health. 2012;21(6):548-55. doi:10.3109/09638237.2012.689435.

## A131 Applying the Plan-Do-Study-Act (PDSA) approach in pragmatic research with safety net health centers

### Amanda Petrik^1^, Jennifer Coury^2^, Jennifer Schneider^1^, Jennifer Rivelli^1^, Beverly Green^3^, Gloria Coronado^1^

#### ^**1**^Kaiser Permanente Center for Health Research, Portland, OR, USA; ^2^Care Oregon, Portland, OR, USA; ^3^Kaiser Permanente Washington Health Research Institute, Seattle, WA, USA

##### **Correspondence:** Amanda Petrik (amanda.f.petrik@kpchr.org)

**Background**

The Plan-Do-Study-Act (PDSA) cycle is a commonly used improvement process in health care settings, although its documented use in pragmatic clinical research is rare. A recent pragmatic clinical research study, called the Strategies and Opportunities to STOP Colon Cancer in Priority Populations (STOP CRC), used this process to optimize the research implementation of an automated colon cancer screening outreach program in intervention clinics. We describe the process of using this PDSA approach, the selection of PDSA topics by clinic leaders, and project leaders’ reactions to using PDSA in pragmatic research.

**Materials and Methods**

STOP CRC is a cluster-randomized pragmatic study that aims to test the effectiveness of a direct-mail fecal immunochemical testing (FIT) program involving eight Federally Qualified Health Centers in Oregon and California. We and a practice improvement specialist trained in the PDSA process delivered structured presentations to leaders of these centers; the presentations addressed how to apply the PDSA process to improve implementation of a mailed outreach program offering colorectal cancer screening through FIT tests. Center leaders submitted PDSA plans and delivered reports via webinar at quarterly meetings of the project’s advisory board. Project staff conducted one-on-one, 45-minute interviews with project leads from each health center to assess the reaction to and value of the PDSA process in supporting the implementation of STOP CRC.

**Results**

Clinic-selected PDSA activities included refining the intervention staffing model, improving outreach materials, and changing workflow steps. Common benefits of using PDSA cycles in pragmatic research were that it provided a structure for staff to focus on improving the program and it allowed staff to test the change they wanted to see.

A commonly reported challenge was measuring the success of the PDSA process with the available electronic medical record tools.

**Conclusion**

Understanding how the PDSA process can be applied to pragmatic trials and the reaction of clinic staff to their use may help clinics integrate evidence-based interventions into their everyday care processes.

## A132 Two models for improving colorectal cancer screening rates in health plan populations

### Jennifer K. Coury^1^, Jennifer Schneider^2^, Beverly Green^3^, Gloria Coronado^2^, Laura Mae Baldwin^4^, Amanda Petrik^2^, Keshia Bigler^5^, Malaika Schwartz^4^

#### ^**1**^Care Oregon, Portland, OR, USA; ^2^The Kaiser Permanente Center for Health Research, Portland, OR, USA; ^3^Kaiser Permanente Washington Health Research Institute, Seattle, WA, USA; ^4^University of Washington, Seattle, WA, USA; ^5^PacificSource, Portland, OR, USA

##### **Correspondence:** Amanda Petrik (amanda.f.petrik@kpchr.org)

**Background**

Screening decreases colorectal cancer (CRC) incidence and mortality by 30-60%, however, CRC screening rates remain low among minorities and low-income individuals. No available data shows the effectiveness of a direct-mail program initiated by health insurance plans that serve these populations.

**Materials and Methods**

The Pilot Program of Mailed Fecal Immunochemical Tests to Increase Colorectal Cancer Screening Rates: BeneFIT is a 4-year descriptive study that supports two health plans implementing a program that mails fecal immunochemical tests (FIT) to patients’ homes. In-depth qualitative interviews were conducted with health plan leaders before implementation. One health plan is in a single state with ~250,000 enrollees, the other is in multiple states with several million enrollees.

**Results**

These health plans are using two distinct models to implement BeneFIT. One health plan is using a Collaborative model. A vendor centrally mails the FIT kits and reminder letters; completed FITs are returned to the clinic, where labs are ordered. This model reduces staff burden while still enabling clinics to use their standard lab, follow-up, and referral processes. Early implementation challenges have been logistical issues for the smaller clinics and data in patient-clinic assignment lists. The other health plan is using a Centralized model. A vendor orders and mails the FITs, and conducts reminder calls; a central lab receives completed FITs and sends results to the vendor, which notifies the patient-assigned clinic. The plan uses its care coordinators to follow-up positive FITs. The model has economics of scale for administration and plan-based follow-up of FIT results. Challenges to implementation have been incomplete prior CRC screening data and possible redundancy of screening.

Baseline qualitative interviews with the health plans identified motivations to participate including increasing patient education, the possibility to improve screening rates and health outcomes, and the opportunity to translate a promising approach to an underserved population and formally evaluate the results. Factors that could affect future health plan decisions to maintain the direct mail approach include return rates, staff and resource requirements, and provider/patient satisfaction with the BeneFIT program.

**Conclusions**

Weighing the successes and challenges in these two plans will help decision makers choose between outreach strategies for CRC screening..

## A133 Mind the gap: Distinguishing implementation agents from implementation strategies to advance implementation science

### Melinda Davis^1,2^, Ross Brownson^3^

#### ^1^Family Medicine, Oregon Health & Science University, Portland, OR, USA; ^2^Oregon Rural Practice-based Research Network, Oregon Health & Science University Portland, OR, USA; ^3^George Warren Brown School of Social Work, Washington University in St. Louis, MO, USA

##### **Correspondence:** Melinda Davis (davismel@ohsu.edu)

**Background**

Understanding how, when, and why interventions are successfully implemented into routine practice is a core challenge for implementation scientists. Current conceptual models depict the flow of implementation research from intervention to implementation strategy to outcomes at three levels (i.e., implementation, service, and client) [1]. Implementation strategies are the ‘how to’ component of changing clinic or community practice; the specific means or methods by which interventions are adopted or implemented [2]. Experts recently identified 73 discrete implementation strategies that were later clustered in 9 domains [3,4]. However, our current models fail to adequately distinguish between the agents who are supporting the implementation process, and the implementation strategies that they use.

**Materials and Methods**

Author commentary and methodologic reflection.

**Results**

We extend current conceptual models to distinguish the following:Interventions (e.g., the evidence-based practice/behavior desired), Implementation agents (e.g., practice facilitators, quality improvement specialists, clinician champions who working within the local context to implement the intervention into practice), Implementation strategies (e.g., audit and feedback, academic detailing – the tools that are used by the implementation agent to achieve the target goal), Context (e.g., characteristics of the proximal and distal environment in which implementation occurs), and Outcomes (e.g., the impact of the intervention on implementation, service, and client outcomes).

We identify four key dimensions of the implementation agent that are likely to influence their effectiveness in selecting implementation strategies that are tailored to suit the local context and the intervention of interest. These include: individual characteristics (e.g., training, personal knowledge), prior experience with target setting (e.g., first project and relationships developing, prior relationships built on trust and mutual understanding), organizational affiliation (e.g., internal or external to the clinic or health system), and motivation for change (e.g., improvement target focused on the intervention, capacity development, or a mix).

**Conclusions**

Our goal is to provide an expanded conceptual model of implementation research which will allow researchers, policy makers, and practitioners to refine the study and understanding of implementation agents, implementation strategies, and contextual factors as well as the interactions between these factors. Separating the implementation agent from the implementation strategies that they use provides a pragmatic model that is congruent with our lived experience in translating research into routine practice.

**References**

1. Proctor E, Silmere H, Raghavan R, Hovmand P, Aarons G, Bunger A, et al. Outcomes for implementation research: conceptual distinctions, measurement challenges, and research agenda. Adm Policy Ment Health. 2011;38(2):65-76.

2. Proctor EK, Powell BJ, McMillen JC. Implementation strategies: recommendations for specifying and reporting. Implement Sci. 2013;8(1):139

3. Powell BJ, Waltz TJ, Chinman MJ, Damschroder LJ, Smith JL, Matthieu MM, Proctor EK, Kirchner JE. A refined compilation of implementation strategies: results from the Expert Recommendations for Implementing Change (ERIC) project. Implement Sci. 2015;10:21.4.

4. Waltz TJ, Powell BJ, Matthieu MM, Damschroder LJ, Chinman MJ, Smith JL, Proctor EK, Kirchner JE. Use of concept mapping to characterize relationships among implementation strategies and assess their feasibility and importance: results from the Expert Recommendations for Implementing Change (ERIC) study. Implement Sci. 2015;10:109.

## A134 Demonstration project of an adapted parenting program implemented with high-risk Latino immigrant families

### Jasney Cogua-Lopez^1^, W. Alex Mason^2^, Mariana Santa-Maria^3^

#### ^1^University of Nebraska Omaha, Office of Latino/Latin American Studies (OLLAS), Omaha, NE, USA; ^2^Boys Town National Research Institute for Child and Family Studies, Boys Town, NE, USA; ^3^Common Sense Parenting at Boys Town, Boys Town, NE, USA

##### **Correspondence:** Jasney Cogua-Lopez (Jasney.Cogua@boystown.org)

**Background**

Common Sense Parenting (CSP) [1] is a six-session, workshop-based parenting program that teaches practical child management skills to parents. CSP has shown evidence of effectiveness in small-scale, nonexperimental studies and is currently listed as a promising program. CSP is fully manualized in English and translated into Spanish. The program has been implemented nationally in both languages but tested primarily in mainstream (middle-income, white, English speaking) families. This is the first study conducted on its adaptation for low-income, Latino Spanish speaking immigrant parents. We compare engagement, outcome, and parent satisfaction data of the adapted CSP in a Latino immigrant community located in a mid-sized city in the Midwestern US with existing summary data of CSP English-speaking parents at a mainstream community in the same or similar cities.

**Materials and Methods**

We use data from CSP classes administered in English (93 participants) and Spanish (165 participants) on sociodemographic characteristics and psychosocial factors collected routinely and link it to data on program participation. This allows an examination and comparison of factors that predict a higher degree of engagement and retention among families (e.g., attending more sessions, completing the program) using regression analysis. Also, routine pretest and posttest scores on outcome assessments are examined and compared using repeated measures analysis of variance to test for improvements in parenting skills and reductions in child problem behaviors. Analyses of the program evaluations (e.g., participant satisfaction) collected at the last day of the workshop are also being conducted.

**Results**

Results from preliminary engagement analyses showed higher CSP participation and completion rates among Latino families (73%) compared to mainstream families (64%). Preliminary analyses of outcome data showed that effect sizes in the adapted, high-need community are medium-large and comparable to the mainstream implementation of the program. Participant evaluations suggest that Latino participants feel the workshops improved their parenting skills and reduced stress and children’s problem behaviors at comparable rates to mainstream families.

**Conclusions**

This is a first step in evaluation of an adapted version of CSP already being implemented by a large service provider. Further studies on the specific adaptation mechanism of CSP from its original model in English to Spanish and within a specific immigrant population are warranted.

**Reference**

1. Burke R, Herron R, Barnes BA Common Sense Parenting: Using your head as well as your heart to raise school-age children (Fourth ed.). Boys Town, NE: Boys Town Press; 2015.

## A135 Online presence of a research center as a tool for dissemination and implementation: Reach and content

### Katherine Seavey^1^, Sarah Lord^1^

#### ^**1**^Center for Technology and Behavioral Health, Geisel School of Medicine at Dartmouth College, Hanover, NH, USA

##### **Correspondence:** Katherine Seavey (sarah.e.lord@dartmouth.edu)

**Background**

The research-to-practice gap is a long-standing issue in behavioral health care that dissemination and implementation science seeks to address [1-3]. Within dissemination and implementation science, there is increased emphasis on understanding factors affecting adoption and implementation of evidence-based practices (EBPs) [4,5]. Across implementation models, individual and organization characteristics related to knowledge and awareness about EBP and access to implementation resources can affect implementation [4-6]. There is strong and growing evidence for the impact of digital behavioral health interventions (i.e. web-based, mobile apps) for substance use and mental health care [7]. There is a need for resources to improve dissemination of these approaches. The Center for Technology and Behavioral Health (CTBH) is a P30 Center of Excellence funded by the National Institute on Drug Abuse that supports research of digital interventions for substance use disorders and related conditions. The CTBH website is a resource for practitioners, researchers, and consumers interested in evidence-based digital behavioral health solutions. In this presentation, we describe features of the CTBH website and how they address the need for knowledge about technology-delivered EBPs and promote implementation of digital behavioral health technologies.

**Materials and Methods**

CTBH has regularly updated Facebook and Twitter accounts and a website. Key features of the website include reviews of programs and related published empirical literature, a blog feed of empirical literature and news stories concerning the state of the field, a growing compendium of resources for research, and a newsletter about Center activities. Google Analytics tracks website usage and follower counts portray the reach of CTBH social media accounts.

**Results**

Since 2013 the CTBH website has had a growing user base. Current usage statistics indicate that a total of 43,371 users have initiated 61,149 sessions on the CTBH website. The CTBH website includes reviews 66 programs, including 284 articles. There have been 860 posts to CTBH’s blog feed, including 100 summaries of scholarly articles. The CTBH Twitter and Facebook accounts have 399 and 672 followers, respectively.

**Conclusions**

CTBH disseminates information about evidence-based digital behavioral health interventions to a wide audience through its website and social media accounts. The CTBH website provides a model for how web-based technology and social media can promote dissemination and implementation science.

**References**

1. Condon TP, Miner LL, Balmer CW, Pintello D. Blending addiction research and practice: strategies for technology transfer. J Subst Abuse Treat. 2008;35(2):156-60. doi: 10.1016/j.jsat.2007.09.004

2. Marinelli-Casey P, Domier CP, Rawson RA. The gap between research and practice in substance abuse treatment. Psychiatr Serv. 2002;53(8):984-7. doi: 10.1176/appi.ps.53.8.984

3. Wandersman A, Duffy J, Flaspohler P, et al. Bridging the gap between prevention research and practice: The interactive systems framework for dissemination and implementation. Am J Community Psychol. 2008;41(3):171-81. doi: 10.1007/s10464-008-9174-z

4. Aarons GA, Wells RS, Zagursky K, Fettes DL, Palinkas LA. Implementing evidence-based practice in community mental health agencies: A multiple stakeholder analysis. Am J Public Health. 2009;99(11):2087-95. doi: 10.2105/AJPH.2009.161711

5. Damschroder LJ, Aron DC, Keith RE, Kirsh SR, Alexander JA, Lowery JC. Fostering implementation of health services research findings into practice: a consolidated framework for advancing implementation science. Implement Sci. 2009;4(50). doi: 10.1186/1748-5908-4-50

6. Tabak RG, Padek MM, Kerner JF, et al. Dissemination and implementation science training needs: Insights from practitioners and researchers. Am J Prev Med. 2017;52(3s3):S322-9. doi: 10.1016/j.amepre.2016.10.005

7. Lord S. Models for effective dissemination and implementation of technology-based approaches to behavioral health care. In: Marsch L, Lord S, Dallery J, editors. Behavioral health care and technology: using science-based innovations to transform practice. New York, NY: Oxford University Press; 2014.

## A136 Planning your implementation right to avoid a restart: Identification of barriers to chlorhexidine bathing implementation in a non-ICU setting

### Jackson Musuuza^1,2^, Linda McKinley^1^, Mary Jo Knobloch^1,2^, Jennifer Dhein^1^, Lobsang Tenzing^1^, Svetlana Bondar^1^, Nasia Safdar^1,2^

#### ^**1**^William S. Middleton Memorial VA Hospital, Madison, WI, USA; ^2^Department of Medicine, University of Wisconsin School of Medicine and Public Health, Madison, WI, USA

##### **Correspondence:** Jackson Musuuza (jmusuuza@medicine.wisc.edu)

**Background**

Daily bathing with chlorhexidine gluconate (CHG) has been shown to reduce healthcare-associated infections [1,2]. Daily CHG bathing has been recommended for intensive care (ICU) patients and emerging evidence supports daily CHG bating for other inpatient populations [3]. CHG bathing in non-ICU settings has not been widely implemented. In this abstract, we describe our experience with the implementation of daily CHG bathing on a non-ICU unit and identify barriers to the implementation.

**Materials and Methods**

The setting was a 20-bed medical-surgical unit of a Veterans Hospital in Madison, WI. We used the Systems Engineering Initiative for Patient Safety (SEIPS) as the main conceptual framework to inform the implementation and evaluation of the intervention. The intervention started in the third quarter of Fiscal Year 2016. We held planning meetings with key stakeholders at the facility and unit level to address prioritization of work system elements needed to facilitate successful implementation. To assess compliance, one-month after starting the intervention, we started conducting direct observations of the process. We identified that unit staff were not using the CHG soap consistently. Therefore, we conducted a focus group with four frontline nursing staff to discuss any barriers and brainstorm possible solutions. We audio recorded and transcribed the discussions and conducted content analysis to summarize the data. We categorized the barriers into five SEIPS work-systems elements: person, organization, tools and technologies, tasks and environment. We also noted more data on barriers through regular interactions with the staff and unit leadership.

**Results**

Participants reported a number of barriers summarized under the following themes: 1) Inadequate training in conducting CHG bathing (organization); 2) inadequate supplies/ tools provided (tools); 3) concerns about the CHG bathing product (tools); 3) interrupted workflow during CHG bathing (organization); and 4) miscommunication between clinical staff and patients (organization). We also observed varying levels of readiness at all levels— facility level, unit level and individual staff level, with the following themes identified: 1) human and physical resources (e.g., staffing shortage, longer bathing time), 2) costs (e.g., concern about cost of CHG product), and 3) communication between staff about CHG bathing. With this feedback, we decided to “restart” the implementation process.

**Conclusions**

In order to minimize barriers, adequate preparation is needed prior to initiating an infection prevention intervention. Careful application of the SEIPS model may facilitate apriori identification of some barriers. Assessing readiness for change at all levels of an organization can be helpful in ensuring successful implementation.

**References**

1. Climo MW, Yokoe DS, Warren DK, Perl TM, Bolon M, Herwaldt LA, et al. Effect of daily chlorhexidine bathing on hospital-acquired infection. N Engl J Med. 2013;368(6):533-42.

2. Huang SS, Septimus E, Kleinman K, Moody J, Hickok H, Avery TR, et al. Targeted versus universal decolonization to prevent ICU infection. N Engl J Med. 2013;368(24):2255-65.

3. Yokoe DS, Anderson DJ, Berenholtz SM, Calfee DP, Dubber E, et al. A compendium of strategies to prevent healthcare-associated infections in acute care hospitals: 2014 updates. Am J Infect Control. 2014;42(8):820-8.

4. Carayon P, Schoofs Hundt A, Karsh BT, Gurses AP, Alvarado CJ, Smith M, Brennan PF. Work system design for patient safety: the SEIPS model. Qual Saf Health Care. 2006;15(Suppl 1):i50-8.

